# 26th Annual Computational Neuroscience Meeting (CNS*2017): Part 2

**DOI:** 10.1186/s12868-017-0371-2

**Published:** 2017-08-18

**Authors:** Leonid L. Rubchinsky, Sungwoo Ahn, Wouter Klijn, Ben Cumming, Stuart Yates, Vasileios Karakasis, Alexander Peyser, Marmaduke Woodman, Sandra Diaz-Pier, James Deraeve, Eliana Vassena, William Alexander, David Beeman, Pawel Kudela, Dana Boatman-Reich, William S. Anderson, Niceto R. Luque, Francisco Naveros, Richard R. Carrillo, Eduardo Ros, Angelo Arleo, Jacob Huth, Koki Ichinose, Jihoon Park, Yuji Kawai, Junichi Suzuki, Hiroki Mori, Minoru Asada, Sorinel A. Oprisan, Austin I. Dave, Tahereh Babaie, Peter Robinson, Alejandro Tabas, Martin Andermann, André Rupp, Emili Balaguer-Ballester, Henrik Lindén, Rasmus K. Christensen, Mari Nakamura, Tania R. Barkat, Zach Tosi, John Beggs, Davide Lonardoni, Fabio Boi, Stefano Di Marco, Alessandro Maccione, Luca Berdondini, Joanna Jędrzejewska-Szmek, Daniel B. Dorman, Kim T. Blackwell, Christoph Bauermeister, Hanna Keren, Jochen Braun, João V. Dornas, Eirini Mavritsaki, Silvio Aldrovandi, Emma Bridger, Sukbin Lim, Nicolas Brunel, Anatoly Buchin, Clifford Charles Kerr, Anton Chizhov, Gilles Huberfeld, Richard Miles, Boris Gutkin, Martin J. Spencer, Hamish Meffin, David B. Grayden, Anthony N. Burkitt, Catherine E. Davey, Liangyu Tao, Vineet Tiruvadi, Rehman Ali, Helen Mayberg, Robert Butera, Cengiz Gunay, Damon Lamb, Ronald L. Calabrese, Anca Doloc-Mihu, Víctor J. López-Madrona, Fernanda S. Matias, Ernesto Pereda, Claudio R. Mirasso, Santiago Canals, Alice Geminiani, Alessandra Pedrocchi, Egidio D’Angelo, Claudia Casellato, Ankur Chauhan, Karthik Soman, V. Srinivasa Chakravarthy, Vignayanandam R. Muddapu, Chao-Chun Chuang, Nan-yow Chen, Mehdi Bayati, Jan Melchior, Laurenz Wiskott, Amir Hossein Azizi, Kamran Diba, Sen Cheng, Elena Y. Smirnova, Elena G. Yakimova, Anton V. Chizhov, Nan-Yow Chen, Chi-Tin Shih, Dorian Florescu, Daniel Coca, Julie Courtiol, Viktor K. Jirsa, Roberto J. M. Covolan, Bartosz Teleńczuk, Richard Kempter, Gabriel Curio, Alain Destexhe, Jessica Parker, Alexander N. Klishko, Boris I. Prilutsky, Gennady Cymbalyuk, Felix Franke, Andreas Hierlemann, Rava Azeredo da Silveira, Stefano Casali, Stefano Masoli, Martina Rizza, Martina Francesca Rizza, Stefano Masoli, Yinming Sun, Willy Wong, Faranak Farzan, Daniel M. Blumberger, Zafiris J. Daskalakis, Svitlana Popovych, Shivakumar Viswanathan, Nils Rosjat, Christian Grefkes, Silvia Daun, Damiano Gentiletti, Piotr Suffczynski, Vadym Gnatkovski, Marco De Curtis, Hyeonsu Lee, Se-Bum Paik, Woochul Choi, Jaeson Jang, Youngjin Park, Jun Ho Song, Min Song, Vicente Pallarés, Matthieu Gilson, Simone Kühn, Andrea Insabato, Gustavo Deco, Katharina Glomb, Adrián Ponce-Alvarez, Petra Ritter, Matthieu Gilson, Adria Tauste Campo, Alexander Thiele, Farah Deeba, P. A. Robinson, Sacha J. van Albada, Andrew Rowley, Michael Hopkins, Maximilian Schmidt, Alan B. Stokes, David R. Lester, Steve Furber, Markus Diesmann, Alessandro Barri, Martin T. Wiechert, David A. DiGregorio, Alexander G. Dimitrov, Catalina Vich, Rune W. Berg, Antoni Guillamon, Susanne Ditlevsen, Romain D. Cazé, Benoît Girard, Stéphane Doncieux, Nicolas Doyon, Frank Boahen, Patrick Desrosiers, Edward Laurence, Nicolas Doyon, Louis J. Dubé, Russo Eleonora, Daniel Durstewitz, Dominik Schmidt, Tuomo Mäki-Marttunen, Florian Krull, Francesco Bettella, Christoph Metzner, Anna Devor, Srdjan Djurovic, Anders M. Dale, Ole A. Andreassen, Gaute T. Einevoll, Solveig Næss, Torbjørn V. Ness, Geir Halnes, Eric Halgren, Geir Halnes, Tuomo Mäki-Marttunen, Klas H. Pettersen, Ole A. Andreassen, Marte J. Sætra, Espen Hagen, Alina Schiffer, Axel Grzymisch, Malte Persike, Udo Ernst, Daniel Harnack, Udo A. Ernst, Nergis Tomen, Stefano Zucca, Valentina Pasquale, Giuseppe Pica, Manuel Molano-Mazón, Michela Chiappalone, Stefano Panzeri, Tommaso Fellin, Kelvin S. Oie, David L. Boothe, Joshua C. Crone, Alfred B. Yu, Melvin A. Felton, Isma Zulfiqar, Michelle Moerel, Peter De Weerd, Elia Formisano, David L. Boothe, Joshua C. Crone, Melvin A. Felton, Kelvin Oie, Piotr Franaszczuk, Roland Diggelmann, Michele Fiscella, Andreas Hierlemann, Felix Franke, Domenico Guarino, Jan Antolík, Andrew P. Davison, Yves Frègnac, Benjamin Xavier Etienne, Flavio Frohlich, Jérémie Lefebvre, Encarni Marcos, Maurizio Mattia, Aldo Genovesio, Leonid A. Fedorov, Tjeerd M.H. Dijkstra, Louisa Sting, Howard Hock, Martin A. Giese, Laure Buhry, Clément Langlet, Francesco Giovannini, Christophe Verbist, Stefano Salvadé, Michele Giugliano, James A. Henderson, Hendrik Wernecke, Bulcsú Sándor, Claudius Gros, Nicole Voges, Paulina Dabrovska, Alexa Riehle, Thomas Brochier, Sonja Grün, Yifan Gu, Pulin Gong, Grégory Dumont, Nikita A. Novikov, Boris S. Gutkin, Parul Tewatia, Olivia Eriksson, Andrei Kramer, Joao Santos, Alexandra Jauhiainen, Jeanette H. Kotaleski, Jovana J. Belić, Arvind Kumar, Jeanette Hellgren Kotaleski, Masanori Shimono, Naomichi Hatano, Subutai Ahmad, Yuwei Cui, Jeff Hawkins, Johanna Senk, Karolína Korvasová, Tom Tetzlaff, Moritz Helias, Tobias Kühn, Michael Denker, PierGianLuca Mana, Sonja Grün, David Dahmen, Jannis Schuecker, Sven Goedeke, Christian Keup, Sven Goedeke, Katja Heuer, Rembrandt Bakker, Paul Tiesinga, Roberto Toro, Wei Qin, Alex Hadjinicolaou, David B. Grayden, Michael R. Ibbotson, Tatiana Kameneva, William W. Lytton, Lealem Mulugeta, Andrew Drach, Jerry G. Myers, Marc Horner, Rajanikanth Vadigepalli, Tina Morrison, Marlei Walton, Martin Steele, C. Anthony Hunt, Nicoladie Tam, Rodrigo Amaducci, Carlos Muñiz, Manuel Reyes-Sánchez, Francisco B. Rodríguez, Pablo Varona, Joseph T. Cronin, Matthias H. Hennig, Elisabetta Iavarone, Jane Yi, Ying Shi, Bas-Jan Zandt, Werner Van Geit, Christian Rössert, Henry Markram, Sean Hill, Christian O’Reilly, Elisabetta Iavarone, Ying Shi, Rodrigo Perin, Huanxiang Lu, Bas-Jan Zandt, Alexander Bryson, Christian Rössert, Michal Hadrava, Jaroslav Hlinka, Ryosuke Hosaka, Mark Olenik, Conor Houghton, Nicolangelo Iannella, Thomas Launey, Tatiana Kameneva, Rebecca Kotsakidis, Hamish Meffin, Jaymar Soriano, Takatomi Kubo, Takao Inoue, Hiroyuki Kida, Toshitaka Yamakawa, Michiyasu Suzuki, Kazushi Ikeda, Samira Abbasi, Amber E. Hudson, Detlef H. Heck, Dieter Jaeger, Joel Lee, Samira Abbasi, Skirmantas Janušonis, Maria Luisa Saggio, Andreas Spiegler, William C. Stacey, Christophe Bernard, Davide Lillo, Christophe Bernard, Spase Petkoski, Andreas Spiegler, Mark Drakesmith, Derek K. Jones, Ali Sadegh Zadeh, Chandra Kambhampati, Jan Karbowski, Zeynep Gokcen Kaya, Yair Lakretz, Alessandro Treves, Lily W. Li, Joseph Lizier, Cliff C. Kerr, Timothée Masquelier, Saeed Reza Kheradpisheh, Hojeong Kim, Chang Sub Kim, Julia A. Marakshina, Alexander V. Vartanov, Anastasia A. Neklyudova, Stanislav A. Kozlovskiy, Andrey A. Kiselnikov, Kanako Taniguchi, Katsunori Kitano, Oliver Schmitt, Felix Lessmann, Sebastian Schwanke, Peter Eipert, Jennifer Meinhardt, Julia Beier, Kanar Kadir, Adrian Karnitzki, Linda Sellner, Ann-Christin Klünker, Lena Kuch, Frauke Ruß, Jörg Jenssen, Andreas Wree, Paula Sanz-Leon, Stuart A. Knock, Shih-Cheng Chien, Burkhard Maess, Thomas R. Knösche, Charles C. Cohen, Marko A. Popovic, Jan Klooster, Maarten H.P. Kole, Erik A. Roberts, Nancy J. Kopell, Daniel Kepple, Hamza Giaffar, Dima Rinberg, Alex Koulakov, Caroline Garcia Forlim, Leonie Klock, Johanna Bächle, Laura Stoll, Patrick Giemsa, Marie Fuchs, Nikola Schoofs, Christiane Montag, Jürgen Gallinat, Ray X. Lee, Greg J. Stephens, Bernd Kuhn, Luiz Tauffer, Philippe Isope, Katsuma Inoue, Yoshiyuki Ohmura, Shogo Yonekura, Yasuo Kuniyoshi, Hyun Jae Jang, Jeehyun Kwag, Marc de Kamps, Yi Ming Lai, Filipa dos Santos, K. P. Lam, Peter Andras, Julia Imperatore, Jessica Helms, Tamas Tompa, Antonieta Lavin, Felicity H. Inkpen, Michael C. Ashby, Nathan F. Lepora, Aaron R. Shifman, John E. Lewis, Zhong Zhang, Yeqian Feng, Christian Tetzlaff, Tomas Kulvicius, Yinyun Li, Rodrigo F. O. Pena, Davide Bernardi, Antonio C. Roque, Benjamin Lindner, Davide Bernardi, Sebastian Vellmer, Ausra Saudargiene, Tiina Maninen, Riikka Havela, Marja-Leena Linne, Arthur Powanwe, Andre Longtin, Francisco Naveros, Jesús A. Garrido, Joe W. Graham, Salvador Dura-Bernal, Sergio L. Angulo, Samuel A. Neymotin, Srdjan D. Antic

**Affiliations:** 1 Indiana University Purdue University Indianapolis, Indianapolis, IN 46032 USA; 2 Stark Neurosciences Research Institute, Indiana University School of Medicine, Indianapolis, IN 46032 USA; 3 Department of Mathematics, East Carolina University, Greenville, NC 27858 USA; 4 Jülich Supercomputing Centre, Forschungszentrum Jülich, 52425 Jülich, Germany; 5 Future Systems, Swiss National Supercomputing Centre, 8092 Zurich, Switzerland; 6 User Engagement and Support, Swiss National Supercomputing Centre, 6900 Lugano, Switzerland; 7 Institut de Neurosciences des Systèmes, Aix Marseille Univ, 13005 Marseille, France; 8 Simulation Lab Neuroscience, Forschungszentrum Jülich, Jülich, Germany; 9 Department of Experimental Psychology, Ghent University, 9000 Ghent, Belgium; 10 Donders Center for Cognitive Neuroimaging, Radboud University, 6525HR Nijmegen, The Netherlands; 11 Department of Electrical, Computer, and Energy Engineering, University of Colorado, Boulder, CO 80309 USA; 12 Department of Neurosurgery, Johns Hopkins School of Medicine, Baltimore, MD 21287 USA; 13 Department of Neurology, Johns Hopkins School of Medicine, Baltimore, MD 21287 USA; 14 Department of Otolaryngology, Johns Hopkins School of Medicine, Baltimore, MD 21287 USA; 15 INSERM, U968, Paris, France; 16 Sorbonne Universités, UPMC University Paris 06, UMR_S 968, Institut de la Vision, Paris, France; 17 CNRS, UMR_7210, Paris, France; 18 Department of Computer Architecture and Technology, University of Granada (CITIC), Granada, Spain; 19 Sorbonne Universités, UPMC Univ Paris 06, INSERM, CNRS, Institut de la Vision, Paris, France; 20 Department of Adaptive Machine Systems, Osaka University, Osaka, Japan; 21 Department of Computer Science, University of Cergy-Pontoise, Cergy-Pontoise, France; 22 Department of Physics and Astronomy, College of Charleston, Charleston, SC 29424 USA; 23 School of Physics, Faculty of Science, University of Sydney, Sydney, NSW 2006 Australia; 24 Center of Excellence for Integrative Brain Function, Australian Research Council, Sydney, Australia; 25 Max Planck Institute for Human Cognitive and Brain Sciences, Saxony, Leipzig, Germany; 26 Department of Computing and Informatics. Faculty of Science and Technology, Bournemouth University, Bournemouth, England, UK; 27 Biomagnetism Section, Heidelberg University, Baden-Württemberg, Heidelberg, Germany; 28 Bernstein Centre for Computational Neuroscience Heidelberg-Mannheim, Heidelberg University, Heidelberg, Germany; 29 Center for Neuroscience, University of Copenhagen, 2200 Copenhagen, Denmark; 30 Brain and Sound Lab, Department of Biomedicine, Basel University, 4056 Basel, Switzerland; 31 Cognitive Science, Indiana University, Bloomington, IN 47405 USA; 32 Physics, Indiana University, Bloomington, IN 47405 USA; 33 Neuroscience and Brain Technology Department, Fondazione Istituto Italiano di Tecnologia, 16163 Genoa, Italy; 34 Scienze cliniche applicate e biotecnologiche, Università dell’Aquila, 67100 L’Aquila, Italy; 35 Krasnow Institute, George Mason University, Fairfax, VA 22030 USA; 36 Bioengineering Department, George Mason University, Fairfax, VA 22030 USA; 37 Institute of Biology, Otto-von-Guericke University, 39120 Magdeburg, Germany; 38 Center for Behavioral Brain Sciences, 39120 Magdeburg, Germany; 39 Network Biology Research Laboratory, Technion - Israel Institute of Technology, 3200003 Haifa, Israel; 40 Department of Physiology, Technion - Israel Institute of Technology, 32000 Haifa, Israel; 41 Institute of Biology, Otto von Guericke University, 39120 Magdeburg, Saxony-Anhalt Germany; 42 Department of Psychology, Birmingham City University, Birmingham, UK; 43 School of Psychology, University of Birmingham, Birmingham, UK; 44 Neural and Cognitive Sciences, NYU Shanghai, Shanghai, 200122 China; 45 Department of Neurobiology, University of Chicago, Chicago, Illinois 60637 USA; 46 Department of Statistics, University of Chicago, Chicago, Illinois 60637 USA; 47 Department of Physiology and Biophysics, University of Washington, Seattle, WA 98195 USA; 48 Allen Institute for Brain Science, Seattle, WA 98109 USA; 49 SUNY Downstate Medical Center, New York City, NY 11228 USA; 50 Computational Physics Laboratory, Ioffe Institute, St Petersburg, Russian Federation 194021; 51 Sechenov Institute of Evolutionary Physiology and Biochemistry, St Petersburg, Russian Federation 194223; 52 Pitié-Salpêtrière Hospital, University Pierre and Marie Curie, 75013 Paris, France; 53 Inserm U1129 Infantile Epilepsies and Brain Plasticity, Paris Descartes University, 75013 Paris, France; 54 Cortex and Epilepsy Group, Brain and Spine Institute, 75013 Paris, France; 55 Department of Cognitive Neuroscience Group for Neural Theory, École Normale Supérieure, 29, rue d’Ulm, 75005 Paris, France; 56 Center for Cognition and Decision Making, NRU Higher School of Economics, Moscow, Russian Federation 109316; 57 Department of Biomedical Engineering, University of Melbourne, Melbourne, Victoria 3010 Australia; 58 National Vision Research Institute, Australian College of Optometry, Melbourne, Victoria 3053 Australia; 59 NVRI, Department of Optometry and Vision Sciences, University of Melbourne, Melbourne, Victoria 3010 Australia; 60 Centre for Neural Engineering, University of Melbourne, Melbourne, Victoria 3053 Australia; 61 Department of Biomedical Engineering, Georgia Institute of Technology, Atlanta, GA 30322 USA; 62 Department of Biomedical Engineering, Emory University, Atlanta, GA 30322 USA; 63 Department of Electrical Engineering, Stanford University, Stanford, CA 94305 USA; 64 Department of Psychiatry and Behavioral Sciences, Emory University, Atlanta, GA 30322 USA; 65 Department of Biology, Emory University, Atlanta, GA 30322 USA; 66 School of Science and Technology, Gerogia Gwinnett College, Lawrenceville, GA 30043 USA; 67 Department of Neurology, University of Florida, Gainesville, FL USA; 68 Instituto de Neurociencias, Consejo Superior de Investigaciones Científicas, Universidad Miguel Hernández, 03550 Sant Joan d’Alacant, Spain; 69 Instituto de Física, Universidade Federal de Alagoas, Maceió, Alagoas 57072-970 Brazil; 70 Departamento de Ingeniería Industrial, Escuela Superior de Ingeniería y Tecnología, Universidad de La Laguna Avda. Astrofísico Fco. Sanchez, s/n, La Laguna, 38205 Tenerife, Spain; 71 Instituto de Física Interdisciplinar y Sistemas Complejos, CSIC-UIB, Campus Universitat de les Illes Balears E, 07122 Palma De Mallorca, Spain; 72 NEARLab, Department of Electronics, Information and Bioengineering, Politecnico di Milano, 20133 Milan, Italy; 73 Brain Connectivity Center, C. Mondino National Neurological Institute, 27100 Pavia, Italy; 74 Department of Brain and Behavioral Sciences, University of Pavia, Via Forlanini 6, 27100 Pavia, Italy; 75 Department of Biotechnology, Indian Institute of Technology Madras, Chennai, Tamilnadu India; 76 Bhupat and Jyoti Mehta School of Biosciences, Department of Biotechnology, Indian Institute of Technology Madras, Chennai, Tamilnadu India; 77 Bhupat and Jyoti Mehta School of Biosciences, Department of Biotechnology, IIT-Madras, Chennai, TN India; 78 High Performance Computing Division, National Center for High-Performance Computing, Hsinchu, Taiwan; 79 National Center for High-Performance Computing, Hsinchu, Taiwan; 80 Institut für Neuroinformatik, Ruhr University Bochum (RUB), 44801 Bochum, Germany; 81 Mercator Research Group ‘Structure of Memory’, Ruhr-University Bochum, Bochum, Germany; 82 Psychology Faculty, University of Wisconsin-Milwaukee, Milwaukee, WI 53201 USA; 83 Ioffe Institute, St.-Petersburg, Russian Federation 194021; 84 Sechenov Institute of Evolutionary Physiology and Biochemistry of RAS, St.-Petersburg, Russian Federation 194223; 85 Pavlov Institute of Physiology, St.-Petersburg, Russian Federation 199034; 86 Department of Applied Physics, Tunghai University, Taichung, Taiwan; 87 Department of Automatic Control and Systems Engineering, University of Sheffield, Sheffield, South Yorkshire S1 3JD UK; 88 Aix Marseille Univ, Inserm, INS, Institut de Neurosciences des Systèmes, Marseille, France; 89 Institut de la Santé et de la Recherche Médical, Institut de Neurosciences des Systèmes, UMR_S 1106, Aix Marseille Université, 13005 Marseille, France; 90 Department of Neurology, State University of Campinas, Campinas, SP 13083-887 Brazil; 91 Unité de Neurosciences, Information et Complexité, CNRS, 91198 Gif-Sur-Yvette, France; 92 Institute for Theoretical Biology, Humboldt-Universität zu Berlin, Berlin, Germany; 93 Department of Neurology, Universitätsmedizin Charité, Berlin, Germany; 94 Neuroscience Institute, Georgia Institute of Technology, Atlanta, GA 30332 USA; 95 School of Biological Sciences, Georgia Institute of Technology, Atlanta, GA 30332 USA; 96 Department of Biosystems Science and Engineering, ETH Zürich, Basel, Switzerland; 97 Ecole Normale Supérieure, Paris, France; 98 Centre National de la Recherche Scientifique, Paris, France; 99 Department of Brain and Behavioral Sciences, University of Pavia, Pavia, Italy; 100 Dipartimento di Informatica, Sistemistica e Comunicazione, Università degli Studi di Milano-Bicocca, Viale Sarca, Milan, Italy; 101 Department of Brain and Behavioral Sciences, University of Pavia, Via Forlanini 6, Pavia, Italy; 102 Dipartimento di Informatica, Sistemistica e Comunicazione, Università degli Studi di Milano-Bicocca, Viale Sarca 336, 20100 Milan, Italy; 103 Institute of Biomaterials and Biomedical Engineering, University of Toronto, Toronto, ON M5S3G9 Canada; 104 Centre for Addiction and Mental Health, Toronto, ON M5T1R8 Canada; 105 Department of Electrical and Computer Engineering, University of Toronto, Toronto, ON M5S3G4 Canada; 106 Department of Psychiatry, University of Toronto, Toronto, ON M5S3G4 Canada; 107 Heisenberg Research Group of Computational Neuroscience - Modeling Neural Network Function, Department of Animal Physiology, Institute of Zoology, University of Cologne, 50674 Cologne, Germany; 108 Cognitive Neuroscience, Institute of Neuroscience and Medicine (INM-3), Research Center Juelich, 52425 Juelich, Germany; 109 Department of Neurology, University Clinic Cologne, 50937 Cologne, Germany; 110 Department of Experimental Physics, University of Warsaw, 02-093 Warsaw, Poland; 111 Istituto Neurologico Carlo Besta, 20133 Milan, Italy; 112 Department of Bio and Brain Engineering, Korea Advanced Institute of Science and Technology, Daejeon, 34141 Republic of Korea; 113 Program of Brain and Cognitive Engineering, Korea Advanced Institute of Science and Technology, Daejeon, 34141 Republic of Korea; 114 Information and Electronics Research Institute, Korea Advanced Institute of Science and Technology, Daejeon, 34141 Republic of Korea; 115 Department of Information and Communication Technologies, Universitat Pompeu Fabra, Barcelona, Spain; 116 Max Plank Institute for Human Development, Berlin, Germany; 117 Clinic and Policlinic for Psychiatry and Psychotherapy, University Medical Center Hamburg-Eppendorf, 20246 Hamburg, Germany; 118 Center for Brain and Cognition, Department of Technology and Information, Universitat Pompeu Fabra, Carrer Ramon Trias Fargas, 25-27, 08005 Barcelona, Spain; 119 Institució Catalana de la Recerca i Estudis Avançats, Universitat Barcelona, Passeig Lluís Companys 23, 08010 Barcelona, Spain; 120 Department of Neurology, Charité - University Medicine, Charitéplatz 1, 10117 Berlin, Germany; 121 Computational Neuroscience Group, Center for Brain and Cognition, Dept. de Tecnologies de la Informació i les Comunicacions, Universitat Pompeu Fabra, Barcelona, Spain; 122 Computational Neuroscience Group, Dept. de Tecnologies de la Informació i les Comunicacions, Universitat Pompeu Fabra, Barcelona, Spain; 123 Epilepsy Monitoring Unit, Department of Neurology, Hospital del Mar Medical Research Institute, Barcelona, Spain; 124 Institute of Neuroscience, Newcastle University, Newcastle upon Tyne, UK; 125 School of Physics, University of Sydney, Sydney, NSW 2006 Australia; 126 Center for Integrative Brain Function, University of Sydney, Sydney, Australia; 127 Institute of Neuroscience and Medicine (INM-6) and Institute for Advanced Simulation (IAS-6), Jülich Research Centre and JARA BRAIN Institute I, 52425 Jülich, Germany; 128 School of Computer Science, University of Manchester, Manchester, M13 9PL UK; 129 Laboratory for Neural Circuit Theory, RIKEN Brain Science Institute, Wako, 351-0106 Japan; 130 Department of Psychiatry, Psychotherapy and Psychosomatics, Medical Faculty, RWTH Aachen University, 52062 Aachen, Germany; 131 Department of Physics, Faculty 1, RWTH Aachen University, 52062 Aachen, Germany; 132 Unite d’Imagerie Dynamique du Neurone, Institut Pasteur, Paris, France; 133 Department of Physiology, Universität Bern, Bern, Switzerland; 134 Department of Mathematics and Statistics, Washington State University Vancouver, Vancouver, WA 98686 USA; 135 Department of Mathematics and Computer Science, Universitat de les Illes Balears, 07122 Palma, Spain; 136 Department of Neuroscience and Pharmacology, University of Copenhagen, 2100 Copenhagen, Denmark; 137 Department of Applied Mathematics I, EPSEB, Universitat Politècnica de Catalunya, 08028 Barcelona, Spain; 138 Department of Mathematical Science, University of Copenhagen, 2100 Copenhagen, Denmark; 139 ISIR, Université Pierre et Marie Curie, 75005 Paris, France; 140 Department of Mathematics and Statistics, Laval University, Quebec, G1V 0A6 Canada; 141 Centre de recherche de l’Institut universitaire en santé mentale de Québec, Québec, Québec G1J 2G3 Canada; 142 Département de physique, de génie physique et d’optique, Université Laval, Québec, Québec G1V 0A6 Canada; 143 Département de mathématiques et de statistique, Université Laval, Québec, Québec G1V 0A6 Canada; 144 Department of Theoretical Neuroscience, ZI - Central Institute for Mental Health, 68159 Mannheim, Germany; 145 Department of Theoretical Neuroscience, Bernstein Center for Computational Neuroscience, Central Institute of Mental Health, Medical Faculty Mannheim, Heidelberg University, Heidelberg, Germany; 146 NORMENT, Institute of Clinical Medicine, University of Oslo, Oslo, Norway; 147 Centre for Computer Science and Informatics Research, University of Hertfordshire, Hatfield, UK; 148 Department of Neurosciences, University of California San Diego, La Jolla, CA USA; 149 Department of Radiology, University of California San Diego, La Jolla, CA 92093-0021 USA; 150 Department of Medical Genetics, Oslo University Hospital, Oslo, Norway; 151 Department of Neurosciences, University of California San Diego, La Jolla, CA 92093-0021 USA; 152 Department of Radiology, University of California San Diego, La Jolla, CA USA; 153 Faculty of Science and Technology, Norwegian University of Life Sciences, 1433 Ås, Norway; 154 Department of Physics, University of Oslo, 0316 Oslo, Norway; 155 Department of Informatics, University of Oslo, 0316 Oslo, Norway; 156 Simula-UiO-UCSD Research and PhD (SUURPh) Training Program, Oslo, Norway; 157 Faculty of Science and Technology, Norwegian University of Life Sciences, Ås, Norway; 158 Department of Neuroscience and Radiology, School of Medicine, UC San Diego, CA USA; 159 Letten Centre and Glialab, Department of Molecular Medicine, Institute of Basic Medical Sciences, University of Oslo, Oslo, Norway; 160 Centre for Molecular Medicine Norway, University of Oslo, Oslo, Norway; 161 Institute of Neuroscience and Medicine (INM-6) and Institute for Advanced Simulation (IAS-6), JARA BRAIN Institute I and Forschungszentrum Jülich, 52425 Jülich, Germany; 162 Computational Neuroscience Lab, Institute for Theoretical Physics, University of Bremen, 28359 Bremen, Germany; 163 Department of Psychology, Methods Section, Johannes Gutenberg University Mainz, 55122 Mainz, Germany; 164 Computational Neuroscience Lab, Institute for Theoretical Physics, University of Bremen, Bremen, Germany; 165 Optical Approaches to Brain Function Laboratory, Department of Neuroscience and Brain Technologies, Istituto Italiano di Tecnologia, Genoa, Italy; 166 Neural Coding Laboratory, Istituto Italiano di Tecnologia, Genova and Rovereto, Italy; 167 Department of Neuroscience and Brain Technologies, Istituto Italiano di Tecnologia, Genoa, Italy; 168 Neural Computation Laboratory, Center for Neuroscience and Cognitive Systems @UniTn, Istituto Italiano di Tecnologia, Rovereto, Italy; 169 US Army Research Laboratory, Aberdeen Proving Ground, Maryland 21005 USA; 170 U.S. Army Research Laboratory, Adelphi, MD 20783 USA; 171 Maastricht Centre for Systems Biology, Maastricht University, 6229 ER Maastricht, The Netherlands; 172 Department of Cognitive Neuroscience, Maastricht University, 6229 ER Maastricht, The Netherlands; 173 US Army Research Laboratory, Aberdeen, Maryland 21005 USA; 174 Department of Neurology, The Johns Hopkins University School of Medicine, Baltimore, Maryland 21287 USA; 175 Department of Biosystems Science and Engineering, ETH Zurich, Basel, Switzerland; 176 Neural Circuit Laboratories, Friedrich Miescher Institute for Biomedical Research, Basel, Switzerland; 177 UNIC, CNRS-FRE3693, 91190 Gif-Sur-Yvette, France; 178 Institut de la Vision, 75012 Paris, France; 179 Krembil Research Institute, University Health Network, Toronto, Ontario M5T 2S8 Canada; 180 Department of Psychiatry and Cell Biology, University of North Carolina at Chapel Hill, Chapel Hill, North Carolina USA; 181 Department of Mathematics, University of Toronto, Toronto, Ontario M5S 3G3 Canada; 182 Department of Physiology and Pharmacology, Sapienza University of Rome, Rome, Italy; 183 Istituto Superiore di Sanità, Rome, Italy; 184 Section for Computational Sensomotorics, Department of Cognitive Neurology, CIN&HIH, Univeristy of Tübingen, Tübingen, Germany; 185 GTC, International Max Planck Research School, University of Tübingen, Tübingen, Germany; 186 Department of Cognitive Science, University of Tübingen, Tübingen, Germany; 187 Department of Psychology, Center for Complex Systems and the Brain Sciences, Florida Atlantic University, Boca Raton, FL USA; 188 Neurosys Team, LORIA, CNRS, INRIA CR Nancy Grand Est, 54500 Villers-Lès-Nancy, France; 189 Université de Lorraine, 54506 Vandoeuvre-Lès-Nancy, France; 190 TNB, Department of biomedical sciences, University of Antwerp, 2610 Wilrijk, Belgium; 191 DIBRIS, University of Genova, 16145 Genoa, Italy; 192 Institute for Theoretical Physics, Goethe University, 60438 Frankfurt Am Main, Germany; 193 Institute of Neuroscience and Medicine (INM-6) and Institute of Advanced Simulation (IAS-6) and JARA Brain Institute I, Jülich Research Centre, 52425 Jülich, Germany; 194 Institut de Neurosciences de la Timone (INT), CNRS - Aix Marseille Universitée, Marseille, France; 195 Theoretical Systems Neurobiology, RWTH Aachen University, Aachen, Germany; 196 School of Physics and Australian Research Council Centre of Excellence for Integrative Brain Function, University of Sydney, Sydney, NSW 2006 Australia; 197 Group for Neural Theory, Ecole Normale Supérieure, 29, rue d’Ulm, 75005 Paris, France; 198 National Research University Higher School of Economics, Centre for Cognition and Decision Making, Moscow, Russia 101000; 199 Department of Cognitive Studies, Ecole Normale Superieure PSL* Research University, 75005 Paris, France; 200 School of Computer Science and Communication, KTH Royal Institute of Technology, Stockholm, Sweden; 201 Department of Numerical Analysis and Computer Science, Stockholm University, Stockholm, Sweden; 202 Department of Neuroscience, Karolinska Institute, Solna, Sweden; 203 Early Clinical Biometrics, AstraZeneca AB R&D, Gothenburg, Sweden; 204 Computational and Experimental Neuroplasticity Laboratory, Krasnow Institute for Advanced Study, George Mason University, George Mason, VA USA; 205 Science for Life Laboratory, Royal Institute of Technology, 17165 Solna, Sweden; 206 Department of Computational Science and Technology, Royal Institute of Technology, 11428 Stockholm, Sweden; 207 Bernstein Center Freiburg, University of Freiburg, 79104 Freiburg, Germany; 208 Department of Computational Science and Technology, KTH Royal Institute of Technology, 11428 Stockholm, Sweden; 209 Department of Neuroscience, Karolinska Institute, 17177 Solna, Sweden; 210 Osaka University, Toyonaka, Osaka Japan; 211 Riken Brain Science Institute, Saitama, Japan; 212 University of Tokyo, Bunkyo, Tokyo Japan; 213 Numenta, Redwood City, CA 94063 USA; 214 Department of Physics, Faculty 1, RWTH Aachen University, 52074 Aachen, Germany; 215 Department of Physics, Faculty I, RWTH Aachen University, 52062 Aachen, Germany; 216 Theoretical Systems Neurobiology, Faculty I, RWTH Aachen University, 52062 Aachen, Germany; 217 Institute of Neuroscience and Medicine (INM-6) and Institute of Advanced Simulation (IAS-6), JARA Brain Institute I, Jülich Research Centre, 52425 Jülich, Germany; 218 Department of Neuropsychology, Max Planck Institute for Human Cognitive and Brain Sciences, Leipzig, Germany; 219 Neuroinformatics Department, Donders Institute for Brain, Cognition and Behaviour, Radboud University Nijmegen, Nijmegen, The Netherlands; 220 Applied and Theoretical neuroanatomy group, Institut Pasteur, Paris, France; 221 Department of Optometry and Vision Sciences, University of Melbourne, Melbourne, Victoria 3010 Australia; 222 Department of Physiology and Pharmacology, SUNY Downstate Medical Center, Brooklyn, NY 11203 USA; 223 Department of Neurology, Kings County Hospital, Brooklyn, NY 11203 USA; 224 InSilico Labs LLC, Houston, TX USA; 225 Institute for Computational Engineering and Sciences, University of Texas at Austin, Austin, TX 78712 USA; 226 John H Glenn Research Center, NASA, Houston, TX USA; 227 ANSYS, Inc., Canonsburg, PA 15317 USA; 228 Thomas Jefferson University, Philadelphia, PA USA; 229 U.S. Food and Drug Administration, Washington, DC USA; 230 KBRWyle, El Segundo, CA 90245 USA; 231 Kennedy Space Center, NASA, Houston, TX USA; 232 Bioengineering and Therapeutic Sciences, University of California, San Francisco, CA USA; 233 Department of Biological Sciences, University of North Texas, Denton, TX 76203 USA; 234 Grupo de Neurocomputación Biológica, Dpto. de Ingeniería Informática, Escuela Politécnica Superior, Universidad Autónoma de Madrid, Madrid, Spain; 235 Institute for Adaptive and Neural Computation, School of Informatics, The University of Edinburgh, Edinburgh, EH8 9AB UK; 236 Laboratory for the Neural Basis of Brain States, Blue Brain Project, EPFL, 1202 Geneva, GE Switzerland; 237 Laboratory of Neural Microcircuity, Brain Mind Institute, EPFL, 1202 Lausanne, GE Switzerland; 238 Blue Brain Project, École Polytechnique Fédérale de Lausanne, 1202 Geneva, GE Switzerland; 239 Florey Institute of Neuroscience and Mental Health, University of Melbourne, Melbourne, VIC 3000 Australia; 240 Department of Cybernetics, Faculty of Electrical Engineering, Czech Technical University in Prague, Prague, 166 27 Czech Republic; 241 Department of Nonlinear Dynamics and Complex Systems, Institute of Computer Science, The Czech Academy of Sciences, Prague, 182 07 Czech Republic; 242 National Institute of Mental Health, Klecany, 250 67 Czech Republic; 243 Department of Applied Mathematics, Fukuoka University, Fukuoka, 814-0180 Japan; 244 School of Biological Sciences, University of Bristol, Bristol, BS81TQ UK; 245 Department of Computer Science, University of Bristol, Bristol, BS81UB UK; 246 School of Mathematical Sciences, University of Nottingham, Nottingham, NG7 2RD UK; 247 Lab for Synaptic Molecules of Memory Persistence, RIKEN, Brain Science Institute, 2-1 Hirosawa Wakoshi, Saitama, 351-0198 Japan; 248 Nara Institute of Science and Technology, Nara, Japan; 249 University of the Philippines - Diliman, Quezon City, Philippines; 250 Yamaguchi University, Ube, Japan; 251 Kumamoto University, Kumamoto, Japan; 252 Department of Biomedical Engineering, Hamedan University of Technology, Hamedan, 65169-13733 Iran; 253 Department Biology, Emory University, Atlanta, GA 30033 USA; 254 Department of Anatomy and Neurobiology, University of Tennessee Health Science Center, Memphis, TN 38163 USA; 255 Department of Biology, Emory University, Atlanta, GA USA; 256 Department of Psychological and Brain Sciences, University of California, Santa Barbara, CA 93106-9660 USA; 257 INSERM UMR 1106 Institut de Neurosciences des Systèmes - Aix-Marseille Université, 13005 Marseille, France; 258 Department of Neurology, Department of Biomedical Engineering, University of Michigan, Ann Arbor, MI 48109 USA; 259 Cardiff University Brain Research Imaging Centre, Cardiff University, Cardiff, UK; 260 Neuroscience and Mental Health Research Institute, Cardiff University, Cardiff, UK; 261 Department of Computer Science, University of Hull, Hull, HU6 7RX UK; 262 University of Warsaw, 02-097 Warsaw, Poland; 263 Cognitive Neuroscience Sector, SISSA, 34136 Trieste, Italy; 264 Sagol School of Neuroscience, Tel Aviv University, 6997801 Tel Aviv, Israel; 265 Kavli Institute, Norwegian University of Science and Technology, 7491 Trondheim, Norway; 266 School of Civil Engineering, University of Sydney, Sydney, NSW 2006 Australia; 267 Centre for Integrative Brain Function, University of Sydney, Sydney, NSW 2006 Australia; 268 CERCO, UMR 5549, CNRS – Université de Toulouse 3, 31300 Toulouse, France; 269 Department of Computer Science, School of Mathematics, Statistics, and Computer Science, University of Tehran, Tehran, Iran; 270 Convergence Research Institute, DGIST, Daegu, 42988 Korea; 271 Department of Physics, Chonnam National University, Gwangju, 61186 Republic of Korea; 272 Lomonosov Moscow State University, Moscow, Russia; 273 Psychological Institute of Russian Academy of Education, Moscow, Russia; 274 Graduate School of Information Science and Engineering, Ritsumeikan University, Kusatsu, Shiga 5258577 Japan; 275 Department of Human and Computer Intelligence, Ritsumeikan University, Kusatsu, Shiga 5258577 Japan; 276 Department of Anatomy, University of Rostock, 18055 Rostock, Germany; 277 Center for Integrative Brain Function, University of Sydney, Sydney, New South Wales 2006 Australia; 278 Center for Integrative Brain Function, University of Sydney, Sydney, New South Wales Australia; 279 Max Planck Institute for Human Cognitive and Brain Sciences, 04103 Leipzig, Germany; 280 Axonal Signalling Group, Netherlands Institute for Neuroscience, Royal Netherlands Academy for Arts and Sciences (KNAW), Meibergdreef 47, 1105 BA Amsterdam, The Netherlands; 281 Cell Biology, Faculty of Science, Utrecht University, Padualaan 8, 3584 CH Utrecht, The Netherlands; 282 Department of Biomedical Engineering, Boston University, Boston, MA 02215 USA; 283 Department of Mathematics and Statistics, Boston University, Boston, MA 02215 USA; 284 Cold Spring Harbor Laboratory, Cold Spring Harbor, NY 11724 USA; 285 New York University, New York, NY 10012 USA; 286 Department of Psychiatry and Psychotherapy, Charité University Medicine and St. Hedwig-Krankenhaus, 10115 Berlin, Germany; 287 Optical Neuroimaging Unit, Okinawa Institute of Science and Technology Graduate University (OIST), Okinawa, 904-0495 Japan; 288 Biological Physics Theory Unit, Okinawa Institute of Science and Technology Graduate University (OIST), Okinawa, 904-0495 Japan; 289 Department of Physics and Astronomy, Vrije Universiteit Amsterdam, 1081 HV Amsterdam, The Netherlands; 290 School of Computer Science and Communication, KTH Royal Institute of Technology, 11428 Stockholm, Sweden; 291 CNRS, University of Strasbourg, Strasbourg, France; 292 Department of Mechano-Informatics, University of Tokyo, Bunkyo, Tokyo 113-8656 Japan; 293 Department of Brain and Cognitive Engineering, Korea University, Seoul, Korea; 294 School of Computing, University of Leeds, Leeds, LS2 9JT UK; 295 School of Computing and Mathematics, Keele University, Newcastle-Under-Lyme, ST5 5BG UK; 296 Department of Neuroscience, Medical University of South Carolina, Charleston, SC 29425 USA; 297 School of Physiology, Pharmacology and Neuroscience, University of Bristol, Bristol, BS8 1TD UK; 298 Department of Engineering Mathematics, University of Bristol, Bristol, BS8 1UB UK; 299 Department of Biology, University of Ottawa, Ottawa, Ontario K1N 6N5 Canada; 300 Center for Neural Dynamics, University of Ottawa, Ottawa, Ontario K1N 6N5 Canada; 301 Ottawa Brain and Mind Research Institute, University of Ottawa, Ottawa, Ontario K1N 6N5 Canada; 302 Department of Management Science, Beijing Normal University, Beijing, 100875 China; 303 Institute of Physics-Biophysics, Georg-August-University, 37077 Goettingen, Germany; 304 Maersk Mc-Kinney Moller Institute, University of Southern Denmark, 5230 Odense, Denmark; 305 School of Systems Science, Beijing Normal University, Beijing, 100875 China; 306 Department of Physics, University of Sao Paulo, Ribeirao Preto, Sao Paulo Brazil; 307 Theory of Complex Systems and Neurophysics, Bernstein Center for Computational Neuroscience, 10115 Berlin, Germany; 308 Department of Physics, Humboldt University of Berlin, 12489 Berlin, Germany; 309 Bernstein Center for Computational Neuroscience - Berlin, 10115 Berlin, Germany; 310 Neuroscience Institute, Lithuanian University of Health Sciences, 50161 Kaunas, Lithuania; 311 Department of Informatics, Vytautas Magnus University, 44404 Kaunas, Lithuania; 312 Computational Neuroscience Group, BioMediTech Institute and Faculty of Biomedical Sciences and Engineering, Tampere University of Technology, Tampere, Finland; 313 Department of Physics and Centre for Neural Dynamics, University of Ottawa, Ottawa, Canada; 314 Department of Neuroscience, Brown University, Providence, RI 02912 USA; 315 Institute for Systems Genomics, University of Connecticut Health Center, Farmington, CT 06030 USA; 316 European Institute for Theoretical Neuroscience, CNRS, Paris, France

## P1 Potential functions of different temporal patterns of intermittent neural synchronization

### Leonid L. Rubchinsky^1,2^, Sungwoo Ahn^3^

#### ^1^Indiana University Purdue University Indianapolis, Indianapolis, IN 46032, USA; ^2^Stark Neurosciences Research Institute, Indiana University School of Medicine, Indianapolis, IN 46032, USA; ^3^Department of Mathematics, East Carolina University, Greenville, NC 27858, USA

##### **Correspondence:** Leonid L. Rubchinsky (lrubchin@iupui.edu)

*BMC Neuroscience* 2017, **18(Suppl 1)**:P1

Synchronization of neural activity has been associated with several neural functions and abnormalities of neural synchrony are associated with different neurological and neuropsychiatric diseases. Neural synchrony in the brain is usually intermittent rather than perfect, even on a very short time-scales. Temporal patterning of synchrony may impact neural function even if the average synchrony strength is fixed (few long intervals of desynchronized dynamics may be functionally different from many short asynchronous intervals even if the average synchrony is the same). Thus, it is of interest to explore network effects of different temporal patterns of neural synchrony.

Detection and quantification of the temporal patterning of synchronization is possible on the very short time-scales (up to one cycle of oscillations, provided that the data episode under analysis possesses some statistically significant synchrony level on the average [1,2]). These techniques allowed for exploration of the fine temporal structure of synchronization of neural oscillations. Experimental studies of neural synchrony in different neural systems report a feature, which appears to be universal: the intervals of desynchronized activity are predominantly very short (although they may be more or less numerous, which affects average synchrony). These observations have been found in different brain areas (cortical and subcortical), different species (humans and rodents), different brain rhythms (alpha, beta, theta), and different disease and behavioral status [3–5].

These observations may suggest that these quick numerous desynchronization events may potentially facilitate creation and break-up of functional synchronized neural assemblies, because both synchronized and desynchronized states are already present in the neural activity. This in turn may promote adaptability and quick reaction of neural systems. Other highly adaptable physiological systems may express short desynchronization dynamics too [6].

We use a minimal network of simple conductance-based model neurons to study how different patterning of intermittent neural synchrony affects formation of synchronized states in response to the common synaptic input to the network. We found that the networks with short desynchronization dynamics are easier to synchronize with the input signal and consider this phenomenon in the context of the experimental observations of neural synchrony patterning.

**References**

1. Ahn S, Park C, Rubchinsky LL: Detecting the temporal structure of intermittent phase locking. *Physical Review E* 2011, **84:** 016201.

2. Rubchinsky LL, Ahn S, Park C: Dynamics of synchronization-desynchronization transitions in intermittent synchronization. *Frontiers in Computational Physics* 2014, **2:**38.

3. Ahn S, Rubchinsky LL: Short desynchronization episodes prevail in the synchronous dynamics of human brain rhythms. *Chaos* 2013, **23:** 013138.

4. Ahn S, Rubchinsky LL, Lapish CC: Dynamical reorganization of synchronous activity patterns in prefrontal cortex - hippocampus networks during behavioral sensitization. *Cerebral Cortex* 2014, **24:** 2553–2561.

5. Ratnadurai-Giridharan S, Zauber SE, Worth RM, Witt T, Ahn S, Rubchinsky LL: Temporal patterning of neural synchrony in the basal ganglia in Parkinson’s disease. *Clinical Neurophysiology 2016*, **127:**1743–1745.

6. Ahn S, Solfest J, Rubchinsky LL: Fine temporal structure of cardiorespiratory synchronization. *American Journal of Physiology* - *Heart and Circulatory Physiology* 2014, **306:** H755–H763.

## P2 NestMC: A morphologically detailed neural network simulator for modern high performance computer architectures

### Wouter Klijn^1^, Ben Cumming^2^, Stuart Yates^2^, Vasileios Karakasis^3^, Alexander Peyser^1^

#### ^1^Jülich Supercomputing Centre, Forschungszentrum Jülich, Jülich, 52425, Germany; ^2^Future Systems, Swiss National Supercomputing Centre, Zürich, 8092, Switzerland; ^3^User Engagement & Support, Swiss National Supercomputing Centre, Lugano, 6900, Switzerland

##### **Correspondence:** Wouter Klijn (w.klijn@fz-juelich.de)

*BMC Neuroscience* 2017, **18(Suppl 1)**:P2

NestMC is a new multicompartment neural network simulator currently under development as a collaboration between the Simulation Lab Neuroscience at the Forschungszentrum Jülich, the Barcelona Supercomputing Center and the Swiss National Supercomputing Center. NestMC will enable new scales and classes of morphologically detailed neuronal network simulations on current and future supercomputing architectures.

A number of “many-core” architectures such as GPU and Intel Xeon Phi based systems are currently available. To optimally use these emerging architecture new approaches in software development and algorithm design are needed. NestMC is being written specifically with performance for this hardware in mind (Figure 1); it aims to be a flexible platform for neural network simulation while keeping interoperability with models and workflows developed for NEST and NEURON.

The improvements in performance and flexibility in themselves will enable a variety of novel experiments, but the design is not yet finalized, and is driven by the requirements of the neuroscientific community. The prototype is open source (https://github.com/eth-cscs/nestmc-proto, https://eth-cscs.github.io/nestmc/) and we invite you to have a look. We are interested in your ideas for features which will make new science possible: we ask you to think outside of the box and build this next generation neurosimulator together with us.

Which directions do you want us to go in?

- Simulate large morphological detailed networks for longer time scales: Study slow developing phenomena.
- Reduce the time to solution: Perform more repeat experiments for increased statistical power.
- Create high performance interfaces with other software: Perform online statistical analysis and visualization of your running models, study the brain at multiple scales with specialized tools, or embed detailed networks in physically modeled animals.
- Optimize dynamic structures for models with time-varying number of neurons, synapses and compartments: simulate neuronal development, healing after injury and age related neuronal degeneration.

Do you have other great ideas? Let us know!
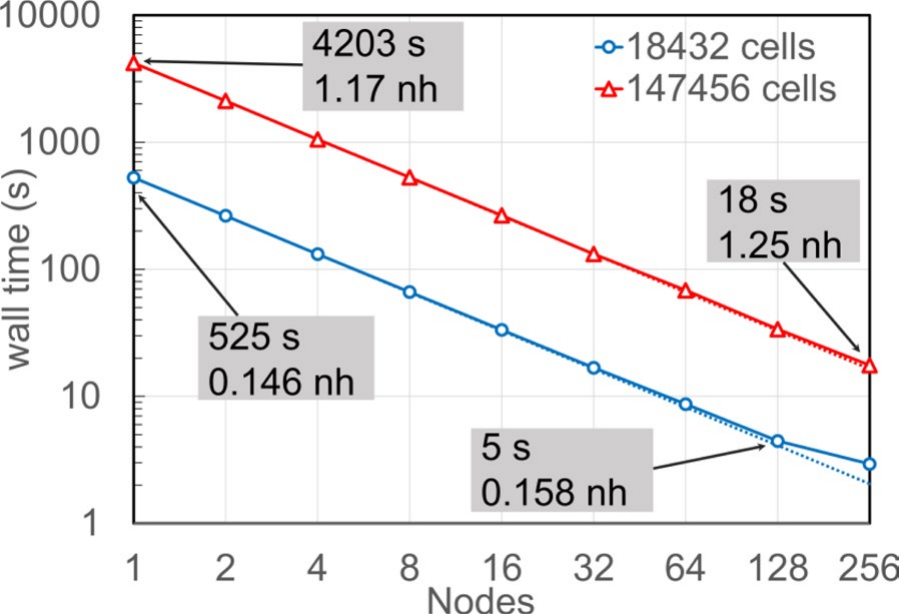


**Figure 1.** Strong Scaling for NestMC. Time to solution for two models of fixed size as a function of the number of compute nodes. Increasing the number of processors reduces the time to solution with little increase in the compute resources required measured in node hours (nh)

## P3 Automatically generating HPC-optimized code for simulations using neural mass models

### Marmaduke Woodman^1^, Sandra Diaz-Pier^2^, Alexander Peyser^2^

#### ^1^Institut de Neurosciences des Systèmes, Aix Marseille Université, Marseille, France; ^2^Simulation Lab Neuroscience, Forschungszentrum Jülich, Jülich, Germany

##### **Correspondence:** Sandra Diaz-Pier (s.diaz@fz-juelich.de)

*BMC Neuroscience* 2017, **18(Suppl 1)**:P3

High performance computing is becoming every day a more accessible and desirable concept for researchers in neuroscience. Simulations of brain networks and analysis of medical data can now be performed on larger scales and with higher resolution. However, many software tools which are currently available to neuroscientists are not yet capable of utilizing the full power of supercomputers, GPGPUs and other computational accelerators.

The Virtual Brain (TVB) [1] software is a validated and popular choice for the simulation of whole brain activity. With TVB the user can create simulations using neural mass models which can have outputs on different experimental modalities (EEG, MEG, fMRI, etc.). TVB allows the scientists to explore and analyze simulated and experimental signals and contains tools to evaluate relevant scientific parameters over both types of data [2]. Internally, the TVB simulator contains several models for the generation of neural activity at the region scale. Most of these neural mass models can be efficiently described with groups of coupled differential equations which are numerically solved for large spans of simulation time. Currently, the models simulated in TVB are written in Python and have not been optimized for parallel execution or deployment on High Performance Computing architectures. Moreover, several elements of these models can be abstracted, generalized and re-utilized, but the design for the right abstract description for the models is still missing.

In this work, we want to present the first results of porting several workflows from The Virtual Brain into High Performance Computing accelerators. In order to reduce the effort required by neuroscientist to utilize different HPC platforms, we have developed an automatic code generation tool which can be used to define abstract models at all stages of a simulation. These models are then translated into hardware specific code. Our simulation workflows involve different neural mass models (Kuramoto [3], Reduced Wong Wang [4], etc.), pre-processing and post-processing kernels (balloon model [5], correlation metrics, etc.). We discuss the strategies used to keep the code portable through several architectures but optimized to each platform. We also point out the benefits and limitations of this approach. Finally, we show initial performance comparisons and give the user an idea of what can be achieved with the new code in terms of scalability and simulation times.

**Acknowledgements**

 We would like to thank our collaborators Lia Domide, Mihai Andrei, Vlad Prunar for their work on the integration of the new software with the already existing TVB platform as well as Petra Ritter and Michael Schirner for providing an initial use case for our tests. The authors would also like to acknowledge the support by the Excellence Initiative of the German federal and state governments, the Jülich Aachen Research Alliance CRCNS grant and the Helmholtz Association through the portfolio theme SMHB and the Initiative and Networking Fund. In addition, this project has received funding from the European Union’s Horizon 2020 research and innovation programme under grant agreement No 720270 (HBP SGA1).

**References**

1. Sanz Leon P, Knock SA, Woodman MM, Domide L, Mersmann J, McIntosh AR, Jirsa V: The Virtual Brain: a simulator of primate brain network dynamics. *Front. Neuroinform.* 2013, **7:** 10.

2. Sanz-Leon, Paula et al.: Mathematical framework for large-scale brain network modeling in The Virtual Brain. *Neuroimage* 2015, **111:** 385–430.

3. Kuramoto Y: Phase-and center-manifold reductions for large populations of coupled oscillators with application to non-locally coupled systems. *Int. J. Bifurcat. Chaos* 1997, **7:** 789–806.

4. Wong, Kong-Fatt, and Xiao-Jing Wang. A recurrent network mechanism of time integration in perceptual decisions. *Journal of Neuroscience* 2006, **26.4:**1314–1328.

5. Buxton, Richard B, Eric C Wong, and Lawrence R Frank: Dynamics of blood flow and oxygenation changes during brain activation: the balloon model. *Magnetic resonance in medicine* 1998, **39.6:** 855–864

## P4 Conjunction or co-activation? A multi-level MVPA approach to task set representations

### James Deraeve^1^, Eliana Vassena^2^, William Alexander^1^

#### ^1^Department of Experimental Psychology, Ghent University, Ghent, 9000, Belgium; ^2^Donders Center for Cognitive Neuroimaging, Radboud University, Nijmegen, 6525HR, Netherlands

##### **Correspondence:** James Deraeve (james.deraeve@ugent.be)

*BMC Neuroscience* 2017, **18(Suppl 1)**:P4

In cognitive experiments, participants are often required to perform tasks where they must apply simple rules, such as “if target is a square, press left”. In everyday life, however, behavior is more complex and may be governed by collections of rules - task sets - that need to be selectively applied in order to achieve a goal. While previous research has demonstrated the involvement of dorsolateral prefrontal cortex (dlPFC) in representation and maintenance of relevant task sets, the nature of this representation remains an open question. One possibility is that task sets are represented as the co-activation of multiple neurons, each of which codes for a single rule. An alternative possibility is that the activity of individual neurons encodes the conjunction of simple rules. In order to answer this question, subjects performed a delayed match-to-sample task while undergoing fMRI. On each trial, subjects were shown a cue indicating one of three possible task sets. Each task set was composed of two out of three possible rules: color/orientation, orientation/shape or shape/color. Following a maintenance period, subjects were presented with a sample stimulus and were asked to memorize the cued task set dimensions. Subsequently, a target stimulus was shown and the subjects had to respond how many cued dimensions of the sample stimulus matched the target stimulus. A control condition was also included in which subjects indicated whether the direction of an arrow (left/right) matched a cued direction. Critically, each task set had one rule in common with another task set and the other rule in common with the remaining task set, allowing us to ascertain through feature selection and multivariate decoding the nature of the underlying neural representations. Under the co-activation hypothesis, voxels important in classifying between task set A and task set B should be those coding for the rules these task sets do not have in common. Since these are the rules that constitute the remaining task set C, classifying task set A and B against C and control using these important voxels as input features should yield classification accuracies at chance. Under the conjunction hypothesis, important voxels code for a specific conjunction of rules and classification of task sets A and B against C and control is possible. A whole brain searchlight analysis reveals a distributed network of regions, including dlPFC, ventrolateral PFC, and parietal cortex with maintenance period activity consistent with the co-activation hypothesis. Conversely, activity in visual cortex during maintenance appears to be consistent with the conjunction hypothesis.

**Acknowledgements**

This research was supported by FWO-Flanders Odysseus II award #G.OC44.13N to WHA.

## P5 Understanding Adaptation in Human Auditory Cortex with Modeling

### David Beeman^1^, Pawel Kudela^2^, Dana Boatman-Reich^3,4^, William S. Anderson^2^

#### ^1^Department of Electrical, Computer, and Energy Engineering^1^, University of Colorado, Boulder, CO 80309, USA; ^2^Department of Neurosurgery, Johns Hopkins School of Medicine, Baltimore, MD 21287, USA; ^3^Department of Neurology, Johns Hopkins School of Medicine, Baltimore, MD 21287, USA; ^4^Department of Otolaryngology, Johns Hopkins School of Medicine, Baltimore, MD 21287, USA

##### **Correspondence:** David Beeman (dbeeman@colorado.edu)

*BMC Neuroscience* 2017, **18(Suppl 1)**:P5

Neural responses in sensory cortex decrease with stimulus repetition, known as adaptation or repetition suppression. In auditory cortex, adaptation is thought to facilitate detection of novel sounds and improve perception in noisy environments. However, the neural mechanisms of adaptation in the human brain remain poorly understood. Here, we combine computational modeling with intracranial electrocorticographic (ECoG) recordings acquired directly from human auditory cortex of epilepsy patients undergoing pre-surgical evaluation.

The model was based on a large layer IV model of primary auditory cortex [1] with multi-compartmental pyramidal cells and fast spiking inhibitory basket cells, implemented in GENESIS 2.4. Thalamic inputs target rows along a tonotopic axis. We extended the model to include short term depression (STD) on excitatory synapses within and between multi-compartmental pyramidal cells and inhibitory basket cells. Model simulations were then compared with human ECoG recordings. Figure 1A shows population auditory evoked potentials (AEP) derived from ECoG recordings using an established 300-trial passive oddball paradigm to measure adaptation [2]. The repetitive stimulus was a 1000 Hz tone (200 ms duration; 82% trials); the non-adapting stimulus was an infrequently presented 1200 Hz tone. Stimuli were presented binaurally at 1.4 s intervals. All patients had electrodes covering posterior temporal cortex auditory areas. AEP results show adaptation to the frequent (repetitive) stimulus for the N1-P2 peaks at about 100–200 ms post-stimulus. Results from model simulations performed using stimulus parameters from the ECoG recordings are shown in Figure 1B and are consistent with patterns of adaptation observed in ECoG recordings.

In the model, as well as cortex, the spatial separation between the location of thalamic afferents for the two tones along the tonotopic axis is large enough that they excite distinct groups of neurons. On average, frequent tone stimuli will follow each other at 1.4 s intervals. The probability of two sequential infrequent tones is low, and the average interval between these stimuli is much larger. Fits of the STD model parameters to measurements in mouse auditory and somatosensory cortex reveal multiple time scales of adaptation. A significant time constant, on the order of one second, governs the time that it takes for the depressed synaptic weight to recover to its original value. Consequently, repeated presentation of the same tone would maintain some synaptic depression between pulses, but responses to infrequent stimuli will have recovered from depression. This is shown in Figure 1B for both the N1 peak, which arises from excitatory synaptic currents in pyramidal cells, and the P2 peak from the subsequent inhibitory currents. These results are consistent with primary auditory depth recordings from one patient. We have also observed similar results across three variations of our model: the single layer IV model with pyramidal and basket cells, a single layer version augmented with facilitating Martinotti cells, and most recently a detailed multilayer model that includes layers II/III and receives depressing synaptic input from layer IV.
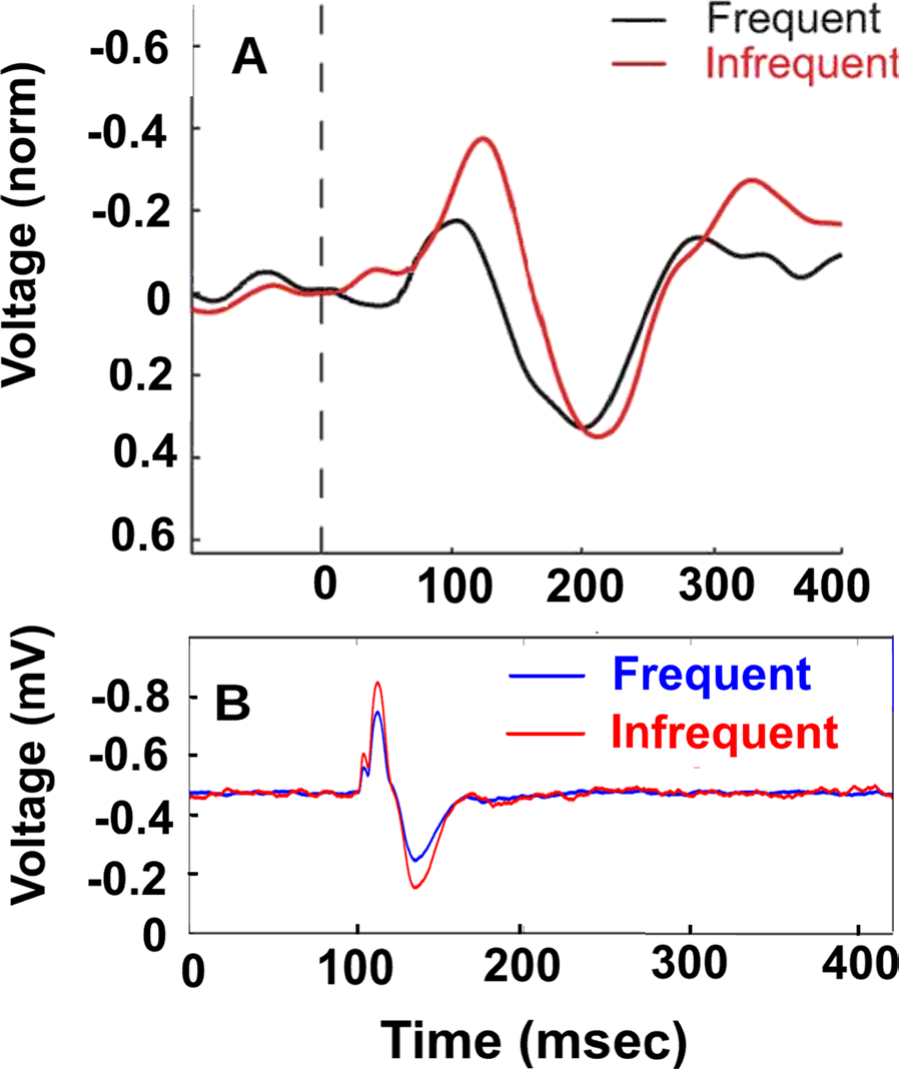


**Figure 1. A.** Average AEPs from [2]. **B.** Results from the model

**Acknowledgements**

Supported by U.S. Army Research Grants W911NF-14-1-0491 and W911NF-10-2-0022

**References**

1. Beeman D: A modeling study of cortical waves in primary auditory cortex. *BMC Neurosci* 2013, **14**(Suppl 1):23.

2. Eliades SJ, Crone NE, Anderson WS, Ramadoss D, Lenz FA, Boatman-Reich D: Adaptation of high-gamma responses in human auditory association cortex. *J Neurophysiol,* 2014 **112**:2147–2163

## P6 Silent and bursting states of Purkinje cell activity modulate VOR adaptation

### Niceto R. Luque^1,2,3^, Francisco Naveros^4^, Richard R. Carrillo^4^, Eduardo Ros^4^, Angelo Arleo^1,2,3^

#### ^1^INSERM, U968, Paris, France; ^2^Sorbonne Universités, UPMC University Paris 06, UMR_S 968, Institut de la Vision, Paris, France; ^3^CNRS, UMR_7210, Paris, France; ^4^Department of Computer Architecture and Technology, University of Granada (CITIC), Granada, Spain

##### **Correspondence:** Niceto R. Luque (niceto.luque@inserm.fr), Angelo Arleo (angelo.arleo@inserm.fr)

*BMC Neuroscience* 2017, **18(Suppl 1)**:P6

Within the cerebellar cortex, the inhibitory projections of Purkinje cells to deep cerebellar nuclei mediate fine motor coordination. Understanding the dynamics of Purkinje cell discharges can thus provide insights into sensorimotor adaptation. It is known that Purkinje cells exhibit three firing modes, namely tonic, silent, and bursting. However, the relation between these firing patterns and cerebellar-dependent behavioural adaptation remains poorly understood. Here, we present a spiking cerebellar model that explores the putative role of the multiple Purkinje operating modes in vestibular ocular reflex (VOR) adaptation. VOR stabilizes images on the retina during head ipsilateral rotations using contralateral eye-movement compensations. The model captures the main cerebellar microcircuit properties and incorporates multiple synaptic plasticity mechanisms at different cerebellar sites (parallel fibres - Purkinje cells, mossy fibres - deep cerebellar nuclei, and Purkinje cells - deep cerebellar nuclei). An analytically reduced version of a detailed Purkinje cell model is at the core of the overall cerebellar function. This allows us to examine the impact of different Purkinje firing modes on VOR adaptation performance. We show that the Purkinje silent mode, through transient disinhibition of targeted cells, gates the neural signals conveyed by mossy fibres to deep cerebellar nuclei. This gating mechanism accounts for early and coarse VOR, prior to the late consolidation of the reflex. In turn, properly timed and sized Purkinje bursts can finely shape the balance between long-term depression and potentiation (LTD/LTP) at mossy fibre - deep cerebellar nuclei synapses. This fine tuning of LTD/LTP balance increases the rate of VOR consolidation. Finally, the silent mode can facilitate the VOR reversal phase by reshaping previously learned synaptic weight distributions. Altogether, these results predict that the interburst dynamics of Purkinje cell activity is instrumental to VOR learning and reversal phase adaptation.

**Acknowledgements**

This study was supported by the European Union NR (658479-Spike Control), the Spanish National Grant NEUROPACT (TIN2013-47069-P) and by the Spanish National Grant PhD scholarship (AP2012-0906).

## P7 The “convis” framework: Population Simulation of the Visual System with Automatic Differentiation using theano

### Jacob Huth^1^, Timothée Masquelier^2^, Angelo Arleo^1^

#### ^1^Sorbonne Universités, UPMC Univ Paris 06, INSERM, CNRS, Institut de la Vision, Paris, France; ^2^CERCO UMR5549, CNRS, University Toulouse 3, Toulouse, France

##### **Correspondence:** Jacob Huth (jahuth@uos.de)

*BMC Neuroscience* 2017, **18(Suppl 1)**:P7

In our effort to extend a mechanistic retina model [1] to use arbitrary non-separable spatio-temporal filters, we created a Python toolbox that is able to formulate a variety of visual models which can then be efficiently computed on a graphics card and even efficiently fitted to electrophysiological recordings using automatic differentiation.

The model for which we were developing this framework is a linear-nonlinear cascade model with contrast gain control and LIF spiking neurons. Previous implementations run very efficiently even on single core systems due to recursive filtering, which restricts temporal filters to a difference of exponentials and spatial filters to 2d Gaussians which are either circular, or have to be aligned with the x and y axis of the simulation. For our research, we required the shape of receptive fields to be arbitrary in space and time and explored the possibilities of using 3d convolution to model finite impulse response linear filters, while still being able to simulate large numbers of cells in reasonable time.

We chose theano [2], a python package developed for deep learning, to construct a computational graph of mathematical operations, including 3d convolutions and alternatively recursive filtering, which can then be compiled via C/C++, CUDA or OpenCL language bindings depending on availability on the specific machine. The graph is optimized before compile time, removing redundancy, choosing appropriate implementation details and replacing some numerically unstable expressions with stable algorithms.

By using this package, we get automatic differentiation, and thus much more efficient optimization, for free. The retina model we are using, while being biologically plausible through its local contrast gain control mechanism, has notoriously many parameters, which are additionally not all independent. But since we implemented the model as an abstract computational graph, rather than the specific simulation code, the model can be analyzed with Computer Algebra System techniques, and gradients of an output with respect to a specific input can be derived automatically. This allows for a range of efficient optimization algorithms to be used, such as the nonlinear conjugate gradient method, Newton method, or Hessian-free optimization.

We examined the error function of the retina model during fitting with respect to different parameters and found that “almost-linear” input parameters, which have essentially a linear effect on the output, but occur before a non-linearity, keep their convex shape and can be sufficiently fitted assuming a quadratic error function. Independent parameters, which each have quadratic error functions, can be optimized with minimal exploration of the parameter space, leading to very fast convergence. In the case of interdependent parameters, the model can be reformulated to eliminate identical solutions. We distinguished areas of convex and concave error functions and found that for non-linear parameters descent to the global minimum is much faster if information of a gradient can be used.

The package is available via Pypi or github [3].

**Acknowledgements**

This research was supported by ANR – Essilor SilverSight Chair ANR-14-CHIN-0001.

**References**

1. Wohrer, A., & Kornprobst, P. Virtual Retina: A biological retina model and simulator, with contrast gain control. *Journal of Computational Neuroscience* 2009, **26(2):** 219–249. http://doi.org/10.1007/s10827-008-0108-4

2. Bastien, F., Lamblin, P., Pascanu, R., Bergstra, J., Goodfellow, I., Bergeron, A., Bouchard, N., Warde-Farley, D., Bengio, Y. Theano: new features and speed improvements. 2012 Retrieved from http://arxiv.org/abs/1211.5590

3. Convis github repository [https://github.com/jahuth/convis/]

## P8 Why does neural activity in ASD have low complexity: from a perspective of a small-world network model

### Koki Ichinose^1^, Jihoon Park^1^, Yuji Kawai^1^, Junichi Suzuki^1^, Hiroki Mori^2^, Minoru Asada^1^

#### ^1^Department of Adaptive Machine Systems, Osaka University, Osaka, Japan; ^2^Department of Computer Science, University of Cergy-Pontoise, Cergy-Pontoise, France

##### **Correspondence:** Koki Ichinose (koki.ichinose@ams.eng.osaka-u.ac.jp)

*BMC Neuroscience* 2017, **18(Suppl 1)**:P8

Autistic spectrum disorder (ASD) is a neurobiological developmental disorder, and many studies have shown abnormality of connectivity structures or neural activities in the brain of ASD. The most typical example of the abnormality is local over-connectivity characterized by increased short range connectivity [1]. Furthermore, it was reported that neural activity in ASD measured by electroencephalography (EEG) have low complexity (multiscale entropy: MSE) [2] and enhanced high frequency oscillation [3]. However, mechanism of the abnormal connectivity and neural activity is not well understood. We aim to comprehend the relation between connectivity and neural activity in the brain of ASD from a perspective of a small-world network model. Our network model consisted of 100 neuron groups, and each neuron group has 1000 spiking neurons. The connectivity of the neuron groups was determined according to the Watts and Strogatz method. The degree of local over-connectivity was modified by the rewiring probability. In our model, the regular and small-world networks denote ASD and typical brains, respectively. We analyzed the complexity and frequency spectrum of the neural activities. Figure 1 shows the relation between graph-theoretical properties (clustering coefficient and degree centrality) and complexity. The regular network has local over-connectivity (high clustering coefficient and high degree centrality) corresponding to the connectivity of ASD. These local over-connected neuron groups of the regular network exhibited higher frequency oscillation and lower complexity than those of other networks.
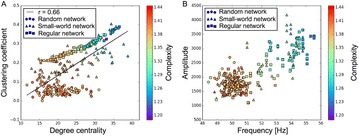


**Figure 1.** Each dot corresponds to each neuron group in the network and its color indicates the complexity (MSE) of the activity in the neuron group. Panel **A** shows the graph-theoretical properties of neuron groups. Clustering coefficient indicates how many closed triangle connections each neuron group has. Degree centrality is the sum of the connection strengths into each neuron group. Panel **B** shows the frequency components of the activities in the neuron groups. The x-axis indicates the peak frequency of neural activity and the y-axis indicates the amplitude of the peak frequency

**Conclusion:** Our results show that ASD brain model which has local over-connectivity (high clustering coefficient and high degree centrality) enhances high frequency oscillation and decreases complexity in neural activity. This implies that local over-connectivity induces the abnormality of neural activity in ASD.

**References**

1. Courchesne, E., and Karen P.: Why the frontal cortex in autism might be talking only to itself: local over-connectivity but long-distance disconnection. *Current Opinion in Neurobiology* 15.2 (2005): 225–230.

2. Bosl, W., et al.: EEG complexity as a biomarker for autism spectrum disorder risk. *BMC Medicine* 9.1 (2011): 18.

3. Cornew, L., et al.: Resting-state oscillatory activity in autism spectrum disorders. *Journal of Autism and Developmental Disorders* 42.9 (2012): 1884–1894.

## P9 Phase-locked mode prediction with generalized phase response curve

### Sorinel A. Oprisan^1^ and Austin I. Dave^1^

#### ^1^Department of Physics and Astronomy, College of Charleston, Charleston, SC 29424, USA

##### **Correspondence:** Sorinel A. Oprisan (oprisans@cofc.edu)

*BMC Neuroscience* 2017, **18(Suppl 1)**:P9

**Introduction:** The simplest possible synchronization mechanism among neurons is based on unidirectional coupling in which a driving neuron drives the activity of postsynaptic neurons. Phase response curve (PRC) method assumes that the only effect of a presynaptic input is a transient change in the firing phase of the postsynaptic neuron(s) and it was successfully used to predict phase-locked modes and synchrony in neural networks setting methodology has been successfully used for predicting one-to-one entrainment in networks where the receiving population always follows the driving population [1]. It has been analytically proven and numerically verified that time-delayed feedback can force coupled dynamical systems onto a synchronization manifold that involves the future state of the drive system, i.e. ``anticipating synchronization’’ [2]. Such a result is counterintuitive since the future evolution of the drive system is anticipated by the response system despite the unidirectional coupling.

**Method:** The phase response curve (PRC) tabulates the transient change in the firing period P_i_ (Fig. 1A) of a neural oscillator in response to one external stimulus at time *t*_*sa*_ per cycle of oscillation [3]. The term PRC has been used almost exclusively in regard to a single stimulus per cycle of neural oscillators. Recently, we suggested a generalization of the PRC that allowed us to account for the overall resetting when two (*t*_*sa*_, *t*_*sb*_) or more inputs are delivered during the same cycle [4]. We previously investigated the existence and stability of phase-locked modes in a neural network with a fixed delay feedback [1]. The novelty of this manuscript is the application of the generalized PRC to a realistic neural network with a dynamic feedback loop (Fig. 1B).

**Results:** Based on generalized PRC, we predicted a stable phase-locked pattern with t_2sa_* = 18.0 ms and t_2sb_* = 44.1 ms. The actual phase-locked values for the fully coupled network were t_2sa_* = 15.2 ms and t_2sb_* = 40.2 ms (Fig. 1C).
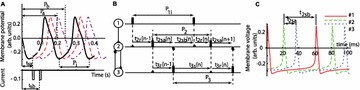


**Figure 1. A.** The first stimulus at phase φ_a_ = t_sa_/P_i_ modifies the intrinsic firing period P_i_ to P_a_ = P_i_(1 + F(φ_a_)), while the second stimulus at phase φ_b_ = t_sb_/P_a_ further resets the firing period to P_b_ = P_a_(1 + F(φ_b_)). ({\bf B}) A typical two stimuli phase response surface for a class I excitable cell. **B.** Neural network with driver (1), driven (2), and feedback look (3), The coupling between the neurons is excitatory (empty triangle) or inhibitory (solid circle). **C.** A typical stable phase-locked mode of a fully coupled network. The network’s firing period was P_1i_ = 60 ms

**Acknowledgements**

SAO acknowledges support for this research from NSF-CAREER award IOS 1054914.

**References**

1. Oprisan SA, Canavier CC: Stability analysis of entrainment by two periodic inputs with a fixed delay. *Neurocomputing* 2003, **52–54**:59–63.

2. Voss HU: Anticipating chaotic synchronization. *Physical Review E* 2000, **61**(5):5115–5119.

3. Perkel DH, Schulman JH, Bullock TH, Moore GP, Segundo JP: Pacemaker neurons: Effects of regularly spaced synaptic input. *Science* 1964, **145**(3627):61–63.

4. Vollmer MK, Vanderweyen CD, Tuck DR, Oprisan SA: Predicting phase resetting due to multiple stimuli. *Journal of the South Carolina Academy of Science* 2015, **13**(2):5–10.

## P10 Neural Field Theory of Corticothalamic Prediction and Attention

### Tahereh Babaie^1,2^, Peter Robinson^1,2^

#### ^1^School of Physics, Faculty of Science, University of Sydney, Sydney, 2006, NSW, Australia; ^2^Center of Excellence for Integrative Brain Function, Australian Research Council, Sydney, Australia

##### **Correspondence:** Tahereh Babaie (tahereh.babaie@sydney.edu.au)

*BMC Neuroscience* 2017, **18(Suppl 1)**:P10

In order to react to the world and achieve survival-relevant outcomes, the brain must attend to those stimuli that are salient, predict their future course, and make use of the results in its responses. In part, this involves combining multiple sensory streams, each of which has a different variance. Experimental evidence shows that the fusion of sensory information is approximately Bayesian. Many theoretical proposals have been made as to how this fusion is achieved, some highly abstract, and some partly based on brain architecture – notable examples include Kalman filters and various predictive coding schemes. However, a common feature is that all proposals to date invoke mathematical operations that the brain must perform at some point, without demonstrating explicitly how neural tissue can accomplish all these tasks, some of which are as complex as matrix inversion and integration over multidimensional probability distributions.

Instead of deciding on a favored mathematical formulation and assuming that it works in the brain, the present work takes the reverse approach of first analyzing realistic corticothalamic responses to simple visual stimuli using neural field theory. This yields system transfer functions that are found to embody key features in common with those of engineering control systems. The finding of analogous quantities in the corticothalamic system enables interpretation of its dynamics in data fusion terms, and assists in localizing the structures in which gain control is possible (see Fig. 1). In particular, these features assist in finding signals within the system, which represent input stimuli and their changes, and these are exactly the types of quantities that are used in control systems to enable prediction of future states, and adjustment of gains to implement attention. The response properties can then be used to drive attention, prediction, decision, and control.
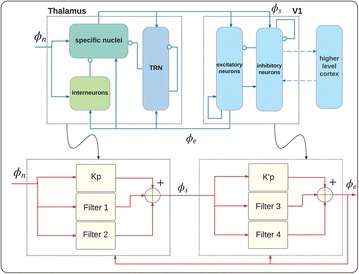


**Figure 1.** The physiologically based corticothalamic model (top) and its analogous control system interpretation (bottom). φ_n_ represents the external stimuli while φ_s_ and φ_e_ are the feedforward and feedback projections, respectively. The arrows in the model represent excitatory effect while the circles depict inhibitory effect

## P11 Top-down dynamics of cortical pitch processing explain the emergence of consonance and dissonance in dyads

### Alejandro Tabas^1,2^, Martin Andermann^3^, André Rupp^3†^, Emili Balaguer-Ballester^2,4†^

#### ^1^Max Planck Institute for Human Cognitive and Brain Sciences, Saxony, Leipzig, Germany; ^2^Department of Computing and Informatics. Faculty of Science and Technology, Bournemouth University, Bournemouth, England, UK; ^3^Biomagnetism Section, Heidelberg University, Baden-Württemberg, Heidelberg, Germany; ^4^Bernstein Centre for Computational Neuroscience Heidelberg-Mannheim, Heidelberg University, Heidelberg, Germany

##### **Correspondence:** Emili Balaguer-Ballester (eb-ballester@bournemouth.ac.uk)

^†^Join last authorship

*BMC Neuroscience* 2017, **18(Suppl 1)**:P11

Pitch is the perceptual correlate of sound’s periodicity and a fundamental attribute of auditory sensation. A dyad consists of a combination of two simultaneous harmonic complex tones that elicit different pitch percepts. Periodicity interactions in dyads give rise to an emergent sensation, described as some degree of consonance or dissonance, strongly correlated to the ratio between the fundamental periods of the involved tones. Simple ratios result in consonant sensations, that become more and more dissonant as the ratio complexity increases.

Consonance and dissonance play a fundamental role in music processing; however, the underlying neural mechanisms associated with the emergence of these percepts are yet poorly understood. In this work, we describe a general mechanism for pitch processing that explains, for the first time to our knowledge, the mechanistic relationship between cortical pitch processing and the sensations of consonance and dissonance. The N100 m is a transient neuromagnetic response of the auditory evoked fields observed in antero-lateral Heschl’s gyrus during MEG recordings. The N100 m latency is strongly correlated to perceived pitch [1]. In our study, we first studied the connection between pitch processing and consonance by measuring the dynamics of the N100 m elicited by six different dyads built from two iterated rippled noise (IRN) [2]. We found a strong and significant correlation between the dissonance percept reported by human listeners and the latency of the N100 m.

Next, we used a hierarchical ensemble model of cortical pitch processing in order to understand the observed correlation. The model receives inputs from a realistic model of the auditory periphery [3], followed by an idealised model of subcortical processing based on the autocorrelation models of pitch [4]. Subcortical input is further processed by a cascade of two networks comprising balanced excitatory and inhibitory ensembles endowing realistic neural and synaptic parameters [5], that effectively transforms the input patterns into a receptive-field-like pitch representation in cortex. Pitch is extracted in the first network due to harmonic connectivity structures inspired by recent findings on the organisation of mammal auditory cortex [6]. The second network further processes extracted pitch, modulating the dynamics of the first network through top-down efferents. The aggregated activity along pyramidal excitatory cells on the first network was predictive for the morphology of the N100 m, and accurately explained the N100 m latency dependence with pitch of iterated rippled noises [1]. Moreover, the model is able to resolve individual pitch values of tones comprised in dyads, quantitatively explain the observed N100 m dependence with consonance, and extends this correlation to further dyads not included in the experiments. Our results introduce a mechanistic explanation of consonance perception based on harmonic facilitation. Subcortical harmonic patterns associated with tones comprised in consonant dyads present a large number of common lower harmonics that, by means of the connectivity structure on the cortical model, facilitate pitch extraction during processing, reducing the processing time for consonant tone combinations. We suggest that these differences in processing time which are reflected in MEG responses are responsible for the differential percept elicited by dissonant and consonant dyads.

**References**

1. Krumbholz, K., Patterson, R. D., Seither-Preisler, A., Lammertmann, C., & Lütkenhöner, B: Neuromagnetic evidence for a pitch processing center in Heschl’s gyrus. *Cereb. Cortex* 2003, **13**(7):765–772.

2. Bidelman, G. M., & Grall, J: Functional organization for musical consonance and tonal pitch hierarchy in human auditory cortex. *NeuroImage* 2014, **101**:204–214.

3. Zilany, M. S. A., Bruce, I. C., & Carney, L. H: Updated parameters and expanded simulation options for a model of the auditory periphery. *JASA* 2014, **135**(1), 283–286.

4. Meddis R, O’Mard LP: Virtual pitch in a computational physiological model. *JASA* 2006, **6**:3861–3869.

5. Wong, K-F, Wang, X-J: A recurrent network mechanism of time integration in perceptual decisions. *J Neurosci* 2006, 26(4):1314–1328.

6. Wang, X: The harmonic organization of auditory cortex. *Front Syst Neurosci* 2013, **7**:114.

## P12 Modeling sensory cortical population responses in the presence of background noise

### Henrik Lindén^1^, Rasmus K. Christensen^1^, Mari Nakamura^2^, Tania R. Barkat^2^

#### ^1^Center for Neuroscience, University of Copenhagen, Copenhagen, 2200, Denmark; ^2^Brain and Sound Lab, Department of Biomedicine, Basel University, Basel, 4056, Switzerland

##### **Correspondence:** Henrik Lindén (hlinden@sund.ku.dk)

*BMC Neuroscience* 2017, **18(Suppl 1)**:P12

The brain faces the difficult task of maintaining a stable representation of key features of the outside world in highly variable sensory surroundings. How does sensory representation in the cortex change in the presence of background ‘noise’ and how does the brain make sense of it?

Here we address this question in the context of the auditory cortex where cells are known to respond in a tuned fashion to the frequency of auditory pure-tone stimuli. We first measured population spike responses using multi-channel extracellular electrodes in awake mice in response to tones with varying frequency, while adding a background noise. Interestingly, we found that the tuning properties of cells changed in the presence of background noise so that they responded more narrowly around their preferred frequency. How does that influence the ability to discriminate between sound stimuli that are close in frequency?

We consider a simple model of the cortical population response profile and assume that the brain compares the weighted read-out of the spike responses in populations corresponding to different pure-tone stimuli in order to discriminate between their frequency. Assuming a fixed width of the read-out profile we vary the width of the cortical activity in the presence of noise activity. Somewhat counter-intuitive, our model predicts that when making the cortical activations narrower (as we experimentally found) the discriminability actually improves with background noise for tones with small frequency differences at the expense of a somewhat degraded performance for tones with larger intervals. Preliminary analysis of behaving mice trained in a go/nogo tone discrimination task largely confirms our theoretical predictions.

Taken together, our results indicate that the sensory representation in auditory cortex varies with background noise in such a way that discriminability between sound stimuli is maintained, and the circuitry may even be optimized for somewhat noisy conditions.

## P13 Cortical circuits from scratch: A metaplastic rule for inducing lognormal firing rates in a cortical model

### Zach Tosi^1^, John Beggs^2^

#### ^1^Cognitive Science, Indiana University, Bloomington, IN 47405, USA; ^2^Physics, Indiana University, Bloomington, IN 47405, USA

##### **Correspondence:** Zach Tosi (ztosi@indiana.edu)

*BMC Neuroscience* 2017, **18(Suppl 1)**:P13

In science one key way of demonstrating the validity of our theories is by simulating a model using the relevant mathematical constructs and seeing if that model produces results consistent with the real-world phenomena in question. If the model matches the data (usually on a phenomenological level in the case of complex systems) then it can be said that our theories are complete with respect to what’s being modeled or more importantly that we understand or have gained insight into the system in question. However, if in such models we must hand tune certain variables *relevant to the subject of the simulation* it is generally taken as a sign that our theories are incomplete.

Previous work on models like the SORN (self-organizing recurrent network) have made significant headway in demonstrating that the key to a model capable of replicating the distinct nonrandom features of cortical behavior and topology through self-organization is the combination of multiple mechanisms of plasticity [1]. However, the SORN and other models like it have had to hand-set one or more relevant properties of the model to do so [1]. We introduce a novel model which like the SORN combines spike-timing dependent plasticity and firing rate homeostasis mechanisms, but which unlike the SORN includes a metaplastic mechanism for self-organizing the target firing rates of neurons to obtain a lognormal distribution. We further add to this work by using inhibitory STDP and incorporating inhibitory plasticity into the network’s homeostatic mechanisms. Collectively this allows us to begin a simulation with a network of excitatory and inhibitory neurons with *no synaptic connections and uniform target firing rates* and end the simulation with a network with *highly complex, biologically plausible synaptic structure and lognormally distributed target firing rates.* This metaplastic artificial neural architecture (MANA) not only reproduces key known features of synaptic topology, but also replicates the known relationships between high and low firing rate neurons in cortex (Fig. 1). The result of this self-organization appears to refine the network’s internal representations of its inputs, and when the resulting graph is subjected to community detection algorithms produce modules with distinct dynamical regimes. In sum, we introduce what could be called the first complete generic cortical network model in that we provide a means of broadly replicating known network level features through mechanism alone.
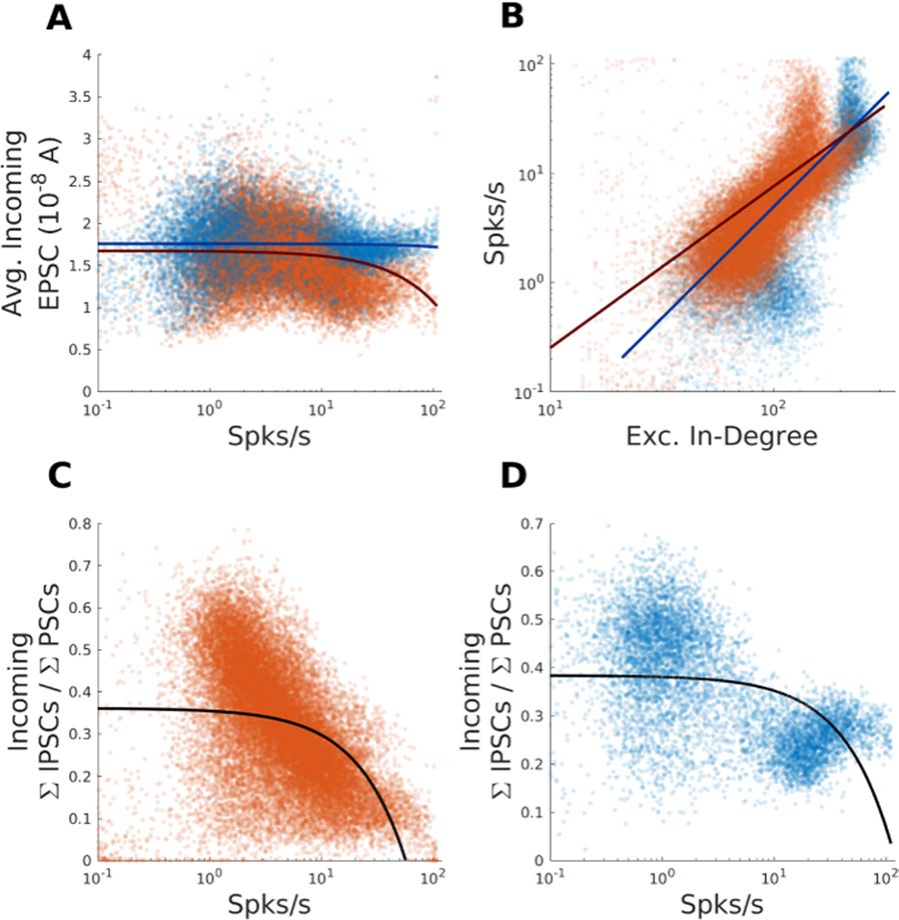


**Figure 1.** Relationships between high and low firing rate neurons in MANA where excitatory (inhibitory) neurons are in orange (blue). Consistent with [2] After self organization high firing rate neurons did not receive stronger connections on average **(A)** however they did receive more excitatory connections **(B)** and less inhibition (**C &D**)

**References**

1. Miner D, Triesch J: Plasticity-Driven Self-Organization under Topological Constraints Accounts for Non-random Features of Cortical Synaptic Wiring. *PLOS Computational Biology* 2016, **12(2)**: e1004759. doi: 10.1371/journal.pcbi.1004759

2. Benedetti BL, Takashima Y, Wen JA, Urban-Ciecko J, Barth AL: Differential Wiring of Layer 2/3 Neurons Drives Sparse and Reliable Firing During Neocortical Development. *Cerebral Cortex (New York, NY)* 2013, **23(11)**, 2690–2699. http://doi.org/10.1093/cercor/bhs257

## P14 Investigating the effects of horizontal interactions on RGCs responses in the mice retina with high resolution pan-retinal recordings

### Davide Lonardoni^1^, Fabio Boi^1^, Stefano Di Marco^2^, Alessandro Maccione^1†^, Luca Berdondini^1†^

#### ^1^Neuroscience and Brain Technology Department, Fondazione Istituto Italiano di Tecnologia, Genova, Italy, 16163; ^2^Scienze cliniche applicate e biotecnologiche, Università dell’Aquila, L’Aquila, Italy, 67100

##### **Correspondence:** Davide Lonardoni (davide.lonardoni@iit.it)

^†^Co-senior authors

*BMC Neuroscience* 2017, **18(Suppl 1)**:P14

Processing of visual information in the cortex relies on a cascade of complex neuronal circuits that receive spike-trains conveyed by the axons of retinal ganglion cells (RGCs). The RGCs are the output neurons of the retinal circuit and are organized in different sub-types that output distinct features of the visual sensory input. Visual information processing in the retina occurs through a mosaic of vertical microcircuits (photoreceptors-bipolar-ganglion cell chain) that are additionally modulated by local and long range lateral connections (e.g. horizontal cells and amacrine cells in the outer and inner plexiform layer, respectively) carrying the contribution of spatially distinct areas of the visual scene [1,2]. However, the study of these horizontal interactions at pan-retinal scale was so far hampered by the lack of large-scale recording neurotechnologies.

To investigate how RGCs encode visual information in such a distributed and parallel manner we took advantage of high-density CMOS multielectrode array sensors [3] and of a visual stimulator developed to provide sub-millisecond and micrometric spatiotemporal precision. This offers the possibility of simultaneously record spontaneous and light-evoked spiking activity from thousands of single RGCs (4096 electrodes, 7.12 mm^2^ active area, 42 µm electrode pitch, 7 kHz sampling rate/electrode). Further, in the mice explanted retina it allows to sample RGCs activity at pan-retinal scale.

Here, we used this platform to investigate whether, and to which extent, horizontal interactions may contribute in shaping RGCs responses in local regions. To do so, we compared the population responses of ON and OFF RGCs in regions of the retina (about a quarter of the active area of the MEA), when the retina was subjected to whole retina stimuli (full-field condition, FF) and when the stimuli were confined to the region in which the recorded RGCs were located (masked condition, M). The visual stimuli consisted in white and black flashes and in moving bars of different spatial gratings (spatial frequency range: 0.026–0.75 cycle/deg). Additionally, we presented stimuli at four different levels of contrast. The recorded signals were spike-sorted and single-units (about 400 in each of the considered region, n = 4 retina) were classified into the main ON and OFF functional categories. Data were analyzed with the aim of identifying differences in the responses of RGCs located in regions that were always subjected to the same local visual stimulus under the two conditions.

Our results reveal that ON-/OFF-cells responses were significantly delayed (~30 ms) when the stimulus was masked in comparison with full-field evoked response. This holds for all the single-units within the considered regions, even for units located far apart from the border of the stimulation mask. We also found that the masked condition mostly affects ON-cell responses, while OFF-cell responses are significantly affected only at low contras conditions. Under pharmacological manipulation (bicuculline), we observed for both ON- and OFF-cells a recovery of the response delay. Overall, our results reveal that horizontal long-range interactions can contribute in shaping the response dynamics of ON and OFF-cells in the retina, thus highlighting the importance of studying the contribution of these interactions.

**Acknowledgements**

This study received financial support from the 7th Framework Program for Research of the European Commission (Grant agreement no 600847: RENVISION, project of the Future and Emerging Technologies (FET) program Neuro-bio-inspired systems (NBIS) FET-Proactive Initiative)).

**References**

1. Masland RH. The neuronal organization of the retina. *Neuron.* 2012, Oct

2. Marre O, Botella-Soler V, Simmons KD, Mora T, Tkačik G, Berry MJ 2nd. High Accuracy Decoding of Dynamical Motion from a Large Retinal Population. *PLoS Comput Biol*. 2015 Jul

3. Maccione A, Hennig MH, Gandolfo M, Muthmann O, van Coppenhagen J, Eglen SJ, Berdondini L, Sernagor E. Following the ontogeny of retinal waves: pan-retinal recordings of population dynamics in the neonatal mouse. *J Physiol.* 2014, Apr

## P15 Calcium base plasticity rule can predict plasticity direction for a variety of stimulation paradigms

### Joanna Jędrzejewska-Szmek^1^, Daniel B. Dorman^1,2^, Kim T. Blackwell^1,2^

#### ^1^Krasnow Institute, George Mason University, Fairfax, VA 22030, USA; ^2^Bioengineering Department, George Mason University, Fairfax, VA 22030, USA

##### **Correspondence:** Joanna Jędrzejewska-Szmek (jjedrzej@gmu.edu)

*BMC Neuroscience* 2017, **18(Suppl 1)**:P15

The striatum is a major site of learning and memory formation for both sequence learning and habit formation. Synaptic plasticity – the long-lasting, activity dependent change in synaptic strength – is one of the mechanisms utilized by the brain for memory storage. Elevation in intracellular calcium is required in all forms of synaptic plasticity. It is widely believed that the amplitude and duration of calcium transients can determine direction of plasticity. It is not certain, however, if this hypothesis can be utilized in the striatum, partly because dopamine is required in potentiation of synaptic responses and partly because the diversity in stimulation paradigms is likely to produce a wide variety of calcium concentrations. To evaluate whether the direction of synaptic plasticity in the striatum can be predicted based on calcium dynamics, we used a model spiny projection neuron (SPN) and a calcium-based plasticity-rule. The SPN model utilized sophisticated calcium dynamics, which included calcium diffusion, buffering and pump extrusion both in the dendritic tree and spines, and also included synaptic AMPAR desensitization to more accurately model frequency dependent plasticity paradigms. The calcium based plasticity rule has been successfully used before [1] to predict plasticity direction of three spike-timing dependent plasticity (STDP) induction paradigms. To further test the rule, we utilized two frequency-dependent plasticity paradigms, one that elicits long-term depression (LTD) and one that elicits long-term potentiation (LTP). Our simulations show that, despite the variation in calcium for different protocols, a single, calcium-based weight change rule (plasticity rule) can explain the change in synaptic weights for two frequency dependent plasticity paradigms. Furthermore, using calcium-based weight change rule we tested whether excitability and possible changes in dopamine depletion on excitability of direct (dSPN) and indirect pathway spiny projection neurons (iSPN) can account for different outcome of STDP paradigm presented in [2] and [3]. Elucidating the mechanisms underlying synaptic plasticity, especially the role and interplay of calcium and dopamine, will allow for better understanding mechanisms of memory storage in health and disease.

**Acknowledgements**

The joint NIH-NSF CRCNS program through NIDA grant R01DA033390

**References**

1. Jędrzejewska-Szmek J, Damodaran S, Dorman DB, Blackwell KT: Calcium dynamics predict direction of synaptic plasticity in striatal spiny projection neurons. *Eur J Neurosci* 2016, doi:10.1111/ejn.13287

2. Shen W, Flajolet M, Greengard P, Surmeier D J: Dichotomous dopaminergic control of striatal synaptic plasticity. *Science* 2008, **321**:848–51. doi: 10.1126/science.1160575.

3. Wu Y-W, Kim J-I, Tawfik VL, Lalchandani RR, Scherrer G, Ding JB: Input- and cell-type-specific endocannabinoid-dependent LTD in the striatum. *Cell Reports* 2015, **10**: 75–87. http://dx.doi.org/10.1016/j.celrep.2014.12.005.

## P16 Unstructured network topology begets privileged neurons and rank-order representation

### Christoph Bauermeister^1,2^, Hanna Keren^3,4^, Jochen Braun^1,2^

#### ^1^Institute of Biology, Otto-von-Guericke University, Magdeburg, 39120, Germany; ^2^Center for Behavioral Brain Sciences, Magdeburg, 39120, Germany; ^3^Network Biology Research Laboratory, Technion - Israel Institute of Technology, Haifa, 3200003, Israel; ^4^Department of Physiology, Technion - Israel Institute of Technology, Haifa, 32000, Israel

##### **Correspondence:** Christoph Bauermeister (chrbauermeister@googlemail.com)

*BMC Neuroscience* 2017, **18(Suppl 1)**:P16

A perennial question in computational neuroscience is the ‘neural code’ employed by spiking assemblies. A convenient model system are assemblies with self-organized instability expressed as all-or-none synchronization events (‘network spikes’). We simulated and analyzed assemblies with random (unstructured) connectivity, synapses with short-term plasticity, with and without external stimulation. Here we show that unstructured connectivity begets a class of privileged ‘pioneer’ neurons that herald network spikes (i.e., by discharging reliably during the incipient phase) and that, by means of the rank-order of their firing, encode the site of any external stimulation. We also demonstrate that existence of pioneers is strongly enhanced by a topological heterogeneity.

Firstly, we show how pioneers arise from an interaction between sensitivity and influentialness, in a manner reminiscent of an amplifier. This clarifies the mechanisms that produce pioneers and their distinctive behavior. Secondly, the rank-order of pioneer discharge reliably encodes the site of any external stimulation, in stark contrast to rate-based encoding schemes. We demonstrate this by stimulating the network at one of five alternative locations and by seeking to decode the stimulated location from different measures of activity (both rate- and time-based). Thirdly, by mapping the number of ‘pioneers’ as a function of recurrent excitation, inhibition, and type of topology, we show that an unstructured and broadly heterogeneous connectivity begets more pioneers than scale-free or homogeneously random connectivity (Figure 1). (Analysis based on interval from neuron discharge to peak population activity. Pioneer neurons exhibit mean larger than standard deviation.) Thus, a robust fraction of pioneers requires more than mere presence of ‘hubs’ (e.g., scale-free topology).

We conclude that random assemblies with self-organized instability offer valuable insights bearing on the issue of ‘neural coding’. Finally, we propose such assemblies as a minimal model for the privileged ‘pioneer neurons’ that reliably predict network spikes in mature cortical neuron assemblies in vitro [1,2].
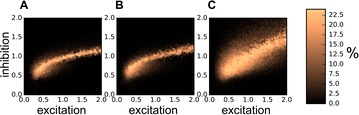


**Figure 1.** Fraction of pioneer neurons and E/I balance, for various unstructured connection topologies: (**A**) homogeneous random, (**B**) scale-free random, (**C**) heterogeneous random. In **A** and **B**, pioneers are restricted to a comparatively narrow regime. In **C**, the domain of pioneers is greatly enlarged

**References**

1. Eytan D, Marom S: Dynamics and effective topology underlying synchronization in networks of cortical neurons. *J Neurosci* 2006, **26(33)**:8465–8476.

2. Shahaf G, Eytan D, Gal A, Kermany E, Lyakhov V, Zrenner C, Marom S. Order-based representation in random networks of cortical neurons. *PLoS Comput* Biol 2008, **4(11)**:e1000228. doi:10.1371/journal.pcbi.1000228

## P17 Finer parcellation reveals intricate correlational structure of resting-state fMRI signals

### João V. Dornas^1^, Jochen Braun^1^

#### ^1^Institute of Biology, Otto von Guericke University, Magdeburg, Saxony-Anhalt 39120, Germany

##### **Correspondence:** João V. Dornas (joaodornas@gmail.com)

*BMC Neuroscience* 2017, **18(Suppl 1)**:P17

The correlation structure of resting-state BOLD signals in the human brain is highly complex. Suitable parcellations of the brain may render this structure simpler and more interpretable. Commonly used parcellations rely on anatomical and/or functional criteria, such as similarity of correlation profiles [1]. Seeking to integrate dependent (and segregate independent) sources of temporal variance, we formed ‘functional clusters’ distinguished by similar short-range correlation profiles inside their anatomically defined regions (‘AAL90’, [2]). Targeting an average size of 200 voxels per cluster, we obtained a parcellation into 758 functional clusters (termed ‘M758’), which proved to be largely contiguous in space. Whereas correlational structure was dense and simple for 90 AAL regions (62% of pairwise correlations are consistently significant, multivariate mutual information 82 bytes), it proved sparse and complex for ‘M758’ (26% of pairs, mutual information 883 bytes). To validate this approach, we examined and compared long-range functional correlations and long-range anatomical connectivity (established by fibre tracking) between cluster pairs in different anatomical regions. The correlational structure of ‘M758’ mirrored anatomical connectivity both overall and in detail. For purposes of comparison, we also established the correlational structure for the published parcellations ‘C400’ ([3]; 30% of pairs, mutual information 490 bytes) and ‘HCP360’ ([4]; 20% of pairs, mutual information 450 bytes), as well as for a parcellation of the same resolution (‘S758’) based on purely spatial criteria (45% of pairs, mutual information 837 bytes). This comparison showed ‘M758’ to be more successful than other parcellations at ‘lumping together’ redundant short-range correlations and ‘separating out’ independent long-range correlations, thereby facilitating the analysis and interpretation of correlational structure. We conclude that a finer parcellation of the human brain, based on a combination of functional and anatomical criteria, reveals a more intricate correlational structure in resting-state BOLD signals.

**Acknowledgements**

This work was supported by a Marie Curie Initial Training Network grant (n° 606901) under the European Union’s Seventh Framework Programme.

**References**

1. Cohen, A. L. and Fair, D. A. and Dosenbach, N. U. and Miezin, F. M. and Dierker, D. and Van Essen, D. C. and Schlaggar, B. L. and Petersen, S. E.: Defining functional areas in individual human brains using resting functional connectivity MRI. Neuroimage 2008, **41(1):** 45–57.

2. Tzourio-Mazoyer, N. and Landeau, B. and Papathanassiou, D. and Crivello, F. and Etard, O. and Delcroix, N. and Mazoyer, B. and Joliot, M.: Automated anatomical labeling of activations in SPM using a macroscopic anatomical parcellation of the MNI MRI single-subject brain. Neuroimage 2002, **15(1):** 273–289.

3. Craddock, R. C. and James, G. A. and Holtzheimer, P. E. and Hu, X. P. and Mayberg, H. S.: A whole brain fMRI atlas generated via spatially constrained spectral clustering. Hum Brain Mapp 2008, **33:** 1914–1928.

4. Glasser, M. F. and Coalson, T. S. and Robinson, E. C. and Hacker, C. D. and Harwell, J. and Yacoub, E. and Ugurbil, K. and Andersson, J. and Beckmann, C. F. and Jenkinson, M. and Smith, S. M. and Van Essen, D. C.: A multi-modal parcellation of human cerebral cortex. Nature 2016, **536:** 171–178.

## P18 Modelling human choices: MADeM and decision-making

### Eirini Mavritsaki^1,2^, Silvio Aldrovandi^1^, Emma Bridger^1^

#### ^1^Department of Psychology, Birmingham City University, Birmingham, UK; ^2^School of Psychology, University of Birmingham, Birmingham, UK

##### **Correspondence:** Eirini Mavritsaki (eirini.mavritsaki@bcu.ac.uk)

*BMC Neuroscience* 2017, **18(Suppl 1)**:P18

In this work, we present a novel approach, to our knowledge, to investigating the underlying brain processes involved in decision making using a computational model for decision making that is based on the Multiple Attribute Decision Making (MADeM) model. In decision making humans need to first evaluate the options available in the decision-making context and the way in which such evaluations are made is subject to debate. A growing body of recent literature [1] has suggested that people evaluate options in relative terms – that is, people are highly sensitive to the context in which an evaluation (and/or a choice) is made. For example, an individual product (e.g., a ready meal or a holiday) is evaluated with reference to other products (e.g., other ready meals, other holidays) available in the decision-making context [2].

The presented work is comprised of behavioral experiments and computational modelling work. The experiments we developed allow us in combination with MADeM, to further investigate the decision-making mechanisms at the cognitive and neuronal level. In the behavioral work participants chose between pairs of items, sampled across different choice domains (e.g., flats to rent and monetary gambles), which differed in terms of two features (e.g., rent cost and distance from a station for flats). Difficulty of choice was manipulated across trials by varying the distance in quality between the two features; for example, in a dominated trial an item was higher in quality on both attributes, whilst in a difficult trial participants were required to perform trade-offs between the two features.

MADeM is based on previous modelling work on visual attention using the spiking Search over Time and Space model (sSoTS). [3]. MADeM is separated into three layers; two layers for the two attributes simulated in the above experiment and one layer that gives us the outcome of the decision-making process. All layers are comprised by pools of excitatory and inhibitory neurons with properties as shown in Mavritsaki et al. [3]. Based on the levels of excitation and competition one of the choices will be higher activated in the Outcome pool and therefore this will be the selected choice. The preliminary results of this study show that the model successfully simulate the results from the behavioral studies using the organization presented above.

**References**

1. Aldrovandi S, Wood AM, Brown GDA: Sentencing, severity, and social norms: A rank-based model of contextual influence on judgments of crimes and punishments. *Acta Psychologica 2013,* **144**: 538–547.

2. Aldrocandi S, Brown GDA, Wood AM: Social norms and ranked-based nudging: Changing willingness to pay for healthy food. *Journal of Experimental Psychology*-*Applied 2015,* **21**: 242–254.

3. Mavritsaki E, Heinke D, Allen HA, Deco G, Humphreys GW: Bridging the gap between physiology and behavior: Evidence from the sSoTS model of human visual attention. *Psychological Review,* **118**: 3–41.

## P19 The interplay between synaptic plasticity and firing rate adaptation sharpens response dynamics with visual learning

### Sukbin Lim^1^, Nicolas Brunel^2,3^

#### ^1^Neural and Cognitive Sciences, NYU Shanghai, Shanghai, China, 200122; ^2^Department of Neurobiology, University of Chicago, Chicago, Illinois, 60637, USA; ^3^Department of Statistics, University of Chicago, Chicago, Illinois, 60637, USA

##### **Correspondence:** Sukbin Lim (sukbin.lim@nyu.edu)

*BMC Neuroscience* 2017, **18(Suppl 1)**:P19

Experience-dependent modifications of synaptic connections are thought to be one of the basic mechanisms for learning and memory. Changes of synaptic strengths lead to changes in inputs to neurons, which should in turn lead to changes in patterns of network activity with learning. In monkey inferotemporal cortex (ITC), changes in activity associated with familiarization with visual images include a reduction of average responses, as well as a broadening of the distribution of time-averaged visual responses [1–3]. Recently, it has been shown that not only the time-averaged responses, but also the dynamics of these visual responses changes with learning. Under conditions of rapid successive presentation of either learned or unlearned stimuli, it was found that familiar images, but not novel images, elicit strong periodic responses, which may underlie an enhancement of dynamic tracking ability with learning [3].

In this work, we investigated the mechanisms of such changes of response dynamics with learning using the time course data obtained in ITC neurons of monkeys during visual learning tasks [1–3]. Previously, we investigated how synaptic plasticity in recurrently connected circuits affects network activity, and derived a synaptic plasticity rule that reproduces changes of the distribution of time-averaged visual responses observed experimentally [4]. Here, we extended this framework to understand how the interaction between synaptic plasticity and various negative feedback mechanisms shapes response dynamics with learning.

We found that a fatigue mechanism analogous to firing rate adaptation, together with depression-dominant synaptic plasticity in recurrent circuits can explain the changes of response dynamics observed experimentally. When novel stimuli are shown repeatedly, the peak response to the second stimuli is smaller than the response to the first, due to slow recovery from the adaptation current. In contrast, for serial presentation of familiar stimuli, depression-dominant changes of synaptic strengths lead to a sharp truncation of the response to the first familiar stimulus, and consequently the response dynamics to the second stimulus is less affected by the adaptation current, and the peak response can be as strong as the first one.

We further demonstrated that such strong periodic response to rapid alternation of learned stimuli is a consequence of enhanced resonance properties with learning. Using firing rate models of recurrent circuits and mathematical analysis of mean-field dynamics, we showed that a long exposure of a single familiar image leads to a damped oscillatory response in contrast to an overdamped response to a novel image, consistent with experimental data [1, 2]. Thus, this work provides a mechanistic understanding of how interactions between depression-dominant synaptic plasticity and a negative feedback mechanism implementing firing rate adaptation shape network response dynamics, and accounts for experimental observations about the effects of visual experience on visual response dynamics of ITC neurons.

**References**

1. Freedman DJ, Riesenhuber M, Poggio T, Miller EK: Experience-dependent sharpening of visual shape selectivity in inferior temporal cortex. *Cerebral cortex* 2006, **16**(11):1631–1644.

2. Woloszyn L, Sheinberg DL: Effects of long-term visual experience on responses of distinct classes of single units in inferior temporal cortex. *Neuron* 2012, **74**(1):193–205.

3. Meyer T, Walker C, Cho RY, Olson CR: Image familiarization sharpens response dynamics of neurons in inferotemporal cortex. *Nature neuroscience* 2014, **17**(10):1388–1394.

4. Lim S, McKee JL, Woloszyn L, Amit Y, Freedman DJ, Sheinberg DL, Brunel N: Inferring learning rules from distributions of firing rates in cortical neurons. *Nature neuroscience* 2015, **18**(12):1804–1810.

## P20 Adaptation and inhibition control the pathologic synchronization in the model of a focal epileptic seizure

### Anatoly Buchin^1,2^, Clifford Charles Kerr^3^, Anton Chizhov^4,5^, Gilles Huberfeld^6,7^, Richard Miles^8^, Boris Gutkin^9,10^

#### ^1^Department of Physiology and Biophysics, University of Washington, Seattle, WA 98195, USA; ^2^Allen Institute for Brain Science, Seattle, WA 98109, USA; ^3^SUNY Downstate Medical Center, New York City, NY 11228, USA; ^4^Computational Physics Laboratory, Ioffe Institute, St Petersburg, 194021, Russian Federation; ^5^Sechenov Institute of Evolutionary Physiology and Biochemistry, St Petersburg, 194223, Russian Federation; ^6^Pitié-Salpêtrière Hospital, University Pierre and Marie Curie, Paris, 75013, France; ^7^Inserm U1129 Infantile Epilepsies and Brain Plasticity, Paris Descartes University, Paris, 75013, France; ^8^Cortex and Epilepsy Group, Brain and Spine Institute, Paris, 75013, France; ^9^Department of Cognitive Neuroscience Group for Neural Theory, École Normale Supérieure, Paris, 75005, France; ^10^Center for Cognition and Decision Making, NRU Higher School of Economics, Moscow, 109316, Russian Federation

##### **Correspondence:** Anatoly Buchin (anat.buchin@gmail.com)

*BMC Neuroscience* 2017, **18(Suppl 1)**:P20

Pharmacoresistant epilepsy is a common neurological disorder in which the basic mechanisms of neuronal excitability and connection processes lead to pathologically synchronous behavior in the brain [1]. In the majority of experimental and theoretical epilepsy models, epilepsy is associated with reduced inhibition in the pathological neural circuits, but intrinsic excitability is usually neglected. Here we developed a novel neural mass model that includes synaptic and intrinsic excitability in the form of spike-frequency adaptation in the excitatory population [2]. We validated our model using local field potential data [3] recorded from human subiculum slices obtained from surgery of temporal lobe epilepsy with hippocampal sclerosis, Fig. 1. We found that synaptic excitability, slow adaptation in the excitatory population, and synaptic noise all play essential roles for generating seizures and disinhibition-induced oscillations. Using bifurcation analysis, we found that transitions towards seizure and back to the resting state take place via Hopf bifurcations. These simulations therefore suggest that single neuron adaptation as well as inhibition are responsible for orchestrating seizure dynamics and transition towards the epileptic state.
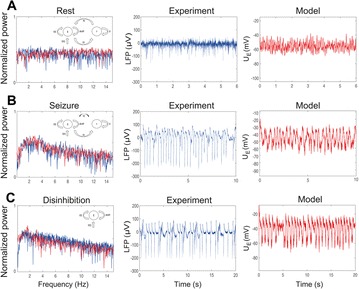


**Figure 1.** Population model in various excitatory regimes. **A.** Activity of a neural population in the resting state. **B.** Seizure state. **C.** Disinhibited state. Each plot contains the model scheme, power spectrum and time traces provided by the excitatory population as well as experimental LFP

**Acknowledgements**

Swartz Foundation, FRM FDT20140930942, ANR-10- LABX-0087 IEC, ANR-10-IDEX-0001-02 PSL, Contract No. 14.608.21.0001 unique ID project RFMEFI60815X0001

**References**

1. Chin JH, Vora N: The global burden of neurologic diseases *Neurology* 2014, **83:**349–351.

2. Buchin AY, Chizhov A: Firing-rate model of a population of adaptive neurons *Biophysics* 2010, **55:**592–599.

3. Huberfeld G, de La Prida LM, Pallud J, Cohen I, Le Van Quyen M, Clemenceau S, Baulac M, Miles R: Glutamatergic pre-ictal discharges emerge at the transition to seizure in human epilepsy *Nature Neuroscience 2011*, **5:**627–634.

## P21 Efficient and Effective Neural Activity Shaping for a Retinal Implant

### Martin J. Spencer^1^, Hamish Meffin^1,2^, Tatiana Kameneva^1^, David B. Grayden^1^, Anthony N. Burkitt^1^

#### ^1^Department of Biomedical Engineering, University of Melbourne, Melbourne, Australia; ^2^ NVRI, Department of Optometry & Vision Sciences, University of Melbourne, Melbourne, Australia

##### **Correspondence:** Martin J. Spencer (mspencer2@unimelb.edu.au)

*BMC Neuroscience* 2017, **18(Suppl 1)**:P21

Electrodes in a retinal implant can be activated in either positive or negative electrical polarity (cathodic or anodic). Either choice leads to activity in retinal ganglion cells (RGCs), and so can be used interchangeably. If every electrode is set to be positive (or negative) then this sets an upper limit on the perceived spatial contrast that can result from stimulation. In this case, the highest spatial gradient is limited by the spread of RGC activation associated with a single electrode. If positive and negative electrode activations are used simultaneously then this leads to higher perceived spatial contrast; electrodes of negative polarity can limit the spread of activity from a positive electrode, or vice versa [1]. The aim of the current investigation is to develop an algorithm that can be used to calculate an electrode activation pattern that takes advantage of this neural activity shaping effect to create a desired pattern of RGC activation.

It was assumed that the neural activity associated with each electrode activation could be summed to predict the total neural activity in the retina. A simple linear model of RGC activation was used: **R** = |**W.E|**, where **R** is a vector of *N*_*R*_ RGC activations, **E** is a vector of *N*_*E*_ electrode levels, and **W** is an array of dimension *N*_*R*_ × *N*_*E*,_ which maps electrode levels to neural activity. The values of the elements of **W** were calculated by assuming that the retinal activation created by each electrode is R_i_ = exp(−*d*_*ij*_^2^/*d*_*0*_^2^), where *d*_*ij*_ is the distance between electrode *E*_*j*_ and RGC *R*_*i*_, and *d*_*0*_ is a scaling factor.

To calculate the electrode pattern required to induce a particular set of retinal activations, it was assumed that the model could be simplified to **R** = **W.E**. This now allows for the manipulation **W** ^**−1**^.**R**_**desired**_ = **E**_**SVD**_ with **W** ^**−1**^ calculated as a pseudoinverse of **W** using singular value decomposition. This assumption may lead to errors in the calculation in cases where the resulting retinal activation **R**_**SVD**_ is negative. However, in simulations, it was not found to produce substantial errors because **R**_**desired**_ is always positive, so **R**_**SVD**_ was only ever marginally negative. Figure 1 shows an example desired image, calculated and simple electrical stimulation patterns, and resulting modeled neural activation results. **R**_**SVD**_ produces high-contrast neural activity, but with some artifacts. **R**_**naive**_, derived by simply mapping the neural activity pattern directly to the electrodes, has lower contrast.
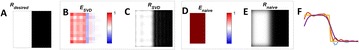


**Figure 1. A.** 2D pattern of desired high (white) and low (black) RGC activity. **B.** Electrode activations of 100 electrodes calculated using **W** ^**−1**^.**R**_**desired**_. Red: positive current, Blue: negative current. **C.** Simulated neural activity resulting from the calculated electrode activation pattern. **D.** Simple electrode pattern. **E.** Neural activity resulting from use of the simple electrode pattern. **F.** Cross secion comparisons between **R**_**desired**_ (yellow), **R**_**SVD**_ before (blue) and after (red) rectification, and **R**_**naive**_ (purple)

It might be anticipated that the complex mapping resulting from the system **R** = |**W.E|** would require sophisticated nonlinear or machine learning techniques to calculate desired electrode activations. However, we found that an efficient linear algebra approach was sufficient to see improved, high contrast, patterns of retinal activation. This approach is feasible for implementation in a retinal implant system.

**Acknowledgements**

Australian Research Council Discovery Project DP140104533

**Reference**

1. van den Honert C, Kesall DC: Focussed intracochlear electrical stimulation with phased array channels. *J Acoust Soc Am* 2007; **121**:3703–3716.

## P22 Application of control theory to neural learning in the brain

### Catherine E Davey^1^, David B. Grayden^1,2^, Anthony N. Burkitt^1^

#### ^1^Department of Biomedical Engineering, University of Melbourne, Melbourne, Victoria, 3010, Australia; ^2^Centre for Neural Engineering, University of Melbourne, Melbourne, Victoria, 3053, Australia

##### **Correspondence:** Catherine E Davey (cedavey@unimelb.edu.au)

*BMC Neuroscience* 2017, **18(Suppl 1)**:P22

Neural plasticity describes the process by which the brain learns, primarily in response to environmental inputs. Supervised learning is a subset of plasticity that describes how one sensory system trains a second sensory modality to achieve a specific goal. This sensory integration requires multimodal neurons, and is often performed in higher cortical layers. Consequently, while there are several small scale examples of supervised learning, more general cases require a complex system of interconnected neurons from multiple brain regions [1]. Supervised learning has historically been modelled using iterative gradient evaluation techniques [2]. Gradient methods typically back-propagate the error through the network to enable local updating of synaptic connection strengths. In a neural context, this assumes that the network can propagate the error backwards, which is a significant assumption that is not biologically plausible at the level of individual synapses [3].

In this work, we pose supervised learning in a control framework, with the primary objective to capitalise on the success of control theory in managing large scale, complex systems [4], by building a biologically plausible system that is scalable. Control theory has played a fundamental role in modern technological systems, with feedback control having many desirable properties, such as the ability to converge to a desired output, stable performance in a noisy environment, and a framework for modelling complex systems [5]. We develop a proof-of-concept and demonstrate equivalent performance to existing techniques. Our prototype system models the supervised learning of target direction from auditory information. More specifically, we model synaptic learning of how to transform interaural time difference (ITD), measuring delay between the arrival of a sound to the left and right ears [6], into an estimate of angle to source. The auditory feature map generated from the ITD is transformed to a source angle feature map in the superior colliculus, though exactly how this is achieved is the subject of ongoing research. The visual system provides the supervisor signal for learning this transformation.

We demonstrate the application of control theory analysis tools by describing the conditions under which the system is robust, stabilisable and controllable. Control parameters are optimised to regulate neural learning and balance the system’s ability to respond to new inputs, while exhibiting robustness to noise. Furthermore, the model is implemented without requiring backwards propagation of signals through synapses. Application of control theory will augment synaptic plasticity research with the advanced methodology and tools of the mature control theory discipline, and has the potential to resolve complexity limitations inherent in current approaches, in addition to addressing the biological plausibility issues associated with current techniques.

**Acknowledgements**

This research was funded by a University of Melbourne fellowship.

**References**

1. Knudsen E, Kokotovic PV, Morse AS. Supervised learning in the brain. *J Neurosci*, 1994, **14(7)**:3985–3997.

2. Hassoun M, *Fundamentals of Artificial Neural Networks*, The MIT Press, 1995.

3. Kasinski A, Ponulak K. Comparison of supervised learning methods for spike time coding in spiking neural networks. *Int. J. Appl. Math. Comput. Sci*, 2006, **16(1)**: 101–113.

4. Drouin M, Abou-Kandil H, Mariton M, *Control of Complex Systems: Methods and Technology*, Springer Science + Business, New York, 1991.

5. Goodwin GC, Graebe SF, Salgado ME, *Control System Design*, Prentice Hall, 2001.

6. Jeffress LA. A place theory of sound localization. *J Comp Physiol Psychol*, 1948, **41**:35–39.

## P23 Modeling dynamic oscillations: a method of inferring neural behavior through mean field network models

### Liangyu Tao^1^, Vineet Tiruvadi^1,2^, Rehman Ali^4^, Helen Mayberg^3^, Robert Butera^1^

#### ^1^Department of Biomedical Engineering, Georgia Institute of Technology, Atlanta, GA, 30332, USA; ^2^Department of Biomedical Engineering, Emory University, Atlanta, GA, 30322, USA; ^3^Department of Psychiatry and Behavioral Sciences, Emory University, Atlanta, GA, 30322, USA; ^4^Department of Electrical Engineering, Stanford University, Stanford, CA, 94305, USA

##### **Correspondence:** Liangyu Tao (ltao31@gatech.edu)

*BMC Neuroscience* 2017, **18(Suppl 1)**:P23

Deep brain stimulation (DBS) is a promising investigational treatment for patients with treatment resistant depression (TRD). Previous studies using diffusion tensor imaging (DTI) have identified key white matter tracts, passing through the subcallosal cingulate (SCC), associated with TRD recovery in patients receiving DBS [1]. However, the mechanism by which stimulation modulates network level pathological activity in the SCC network has not been clearly established. Local field potential recordings in the SCC have shown the emergence of transient, nonlinear, decreases in higher frequency power over 30–60 s following specific stimulation conditions in a subset of implanted patients. We provisionally define these electrophysiological signatures as a transient down-chirps. These transient down-chirps, when present, are a reproducible SCC and potential biometric of neural circuit interactions seen with initial exposure of the SCC to high frequency stimulation. Understanding why and how stimulation causes this electrophysiological behavior in the SCC network is an important step in increasing the efficiency and success rate of treatment for patients with TRD.

One mechanism of transient down-chirp generation was hypothesized to be the excitatory/inhibitory balance of neural regions following stimulation. Mean field network models can be used to understand the dynamics of groups of neurons that would cause the observed signals in LFP recordings. Each neural region was modeled based on a Wilson Cowan population with GABA and Glutamate dominated signaling [2]. White matter tracts connecting neural regions were modeled assuming Glutamate dominant signaling.
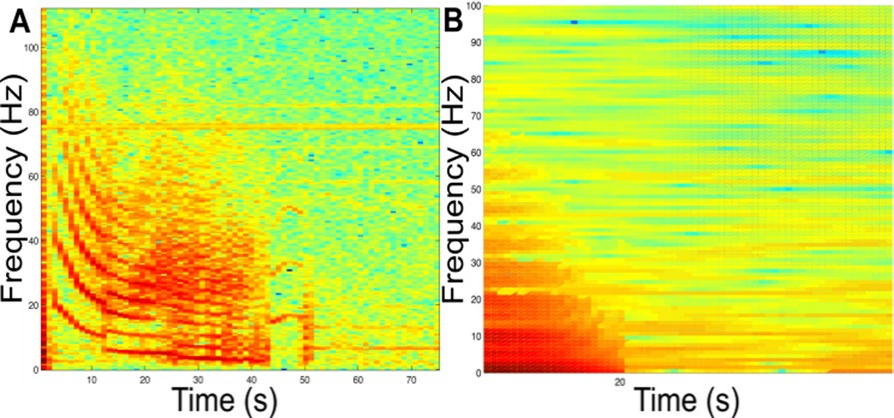


**Figure 1. A**. This is a spectrogram of LFP recordings showing transient down-chirp. **B**. This is a spectrogram of model generated transient down chirp

We show how a simple network, based on the topological layout of the SCC network, of mean field neural populations models could be used to generate qualitatively the down-chirps seen in the local field potentials (see Figure 1). We then characterize these modeled down-chirps by the excitatory/inhibitory balance associated with each neural region. Using these metrics, we classified the maximum likelihood of excitatory and inhibitory responses of neural regions following stimulation in generating these down-chirps.

Our results highlight the role of utilizing a network of population models informed by biology as a method of predicting neural activity in neural regions that are expensive and difficult to measure. Importantly, these models serve as a possible method of informing future treatment strategies in DBS.

**Acknowledgements**

Liangyu Tao is supported by NIH training grant 5R90DA033462 and the President’s Undergraduate Research Award (Fall 2016). DBS support: Hope for Depression Research Foundation, FDA IDE G130107 (HM).

**References**

1. Riva-Posse P, Choi KS, Holtzheimer PE, McIntyre CC, Gross RE, Chaturvedi A, Crowell AL, Garlow SJ, Rajendra JK, Mayberg HS: Defining Critical White Matter Pathways Mediating Successful Subcallosal Cingulate Deep Brain Stimulation for Treatment-Resistant Depression. *Biological Psychiatry* 2014, **76**(12):963–969.

2. Wilson HR, Cowan JD: Excitatory and Inhibitory Interactions in Localized Populations of Model Neurons. *Biophysical Journal* 1972, **12**(1):1–24.

## P24 Synaptic strengths dominate phasing of motor neurons by a central pattern generator

### Cengiz Gunay^1,2^, Anca Doloc-Mihu^1^, Damon Lamb^1,3^, Ronald L Calabrese^1^

#### ^1^Department of Biology, Emory University, Atlanta, GA 30322, USA; ^2^School of Science and Technology, Gerogia Gwinnett College, Lawrenceville, GA 30043, USA; ^3^Department of Neurology, Univ. Florida, Gainesville, FL, USA

##### **Correspondence:** Cengiz Gunay (cgunay@ggc.edu)

*BMC Neuroscience* 2017, **18(Suppl 1)**:P24

Rhythmic motor output is driven by upstream central pattern generator (CPG) phasing, whose synapses therefore play an important role in shaping motor patterns. Individual animals show large variability in motor circuits, not only in circuit synaptic parameters, but also in intrinsic neuronal parameters [1]. It is not known how this observed intrinsic variability influences motor circuit function across animals. Previous computer model parameter searches revealed a large landscape of intrinsic and circuit parameter combinations that can produce functional output in a general model population [2]. However, asking specific questions about individual animals requires experimental data. A leech heartbeat motor neuron model was previously tuned to individual animal circuit data using a multi-objective evolutionary algorithm (MOEA) approach [3]. Relative synaptic weights were measured experimentally [4], and model parameters were optimized to estimate intrinsic conductance parameters that produce a functional motor pattern output within the physiological ranges of the individual preparation. The method did not scale to find intrinsic conductance combinations that can match the outputs measured from five other preparations, even though the method’s convergence was improved with a fuzzy fitness criterion [5]. MOEA parameter search succeeded only when synaptic weights were allowed to vary from measured averages. Unique solutions found for each of the preparations showed the criticality of the relative weights of different synaptic inputs in determining motor output patterns as opposed to intrinsic parameters. To investigate whether the newly found synaptic weights are within experimental variability, we have calculated standard deviations of synaptic conductances from spike-triggered averages (STA) of voltage-clamped synaptic current traces. Variable baseline contributed to increased noise and variation of STA current traces. We found that a bandpass filtering method reduced baseline variability and therefore variability of estimated weights. Despite reduced variability, new synaptic weights found by the MOEA search were still within one standard deviation of experimentally measured values in each of the six preparations. Furthermore, we showed that a neuron model with same intrinsic conductances is able to produce functional outputs in all six of our preparations, as long as new synaptic weights were used. In summary, we used MOEA parameter search as a tool and improved spike-triggered average estimation of synaptic weights and their variability to find that CPG networks in individual animals require precise relative synaptic weights irreplaceable by adjusting intrinsic properties. Furthermore, we conclude that measured synaptic weights should be used with caution in computer models because any experimental noise may break functional output.

**Acknowledgements**

Angela Wenning and Brian Norris provided experimental data for synaptic weights. Supported by NIH NINDS 1 R01 NS085006.

**References**

1. Bucher D, Prinz AA, Marder E: Animal-to-animal variability in motor production in adults and during growth. *J Neurosci* 2005 **25(7):**1611–19.

2. Prinz AA, Bucher D, Marder E: Similar network activity from disparate circuit parameters. *Nat Neurosci* 2004 **7(12):**1345–52.

3. Lamb D, Calabrese RL: Correlated conductance parameters in leech heart motor neurons contribute to motor pattern formation. *PLoS ONE* 2013 **8(11):** e79267

4. Norris BJ, Weaver AL, Wenning A, García PS, Calabrese RL: A Central Pattern Generator Producing Alternative Outputs: Pattern, Strength, and Dynamics of Premotor Synaptic Input to Leech Heart Motor Neurons. *J Neurophysiol* 2007 **98:**2992–3005.

5. Smolinski TG, Prinz AA, Zurada, JM: Hybridization of rough sets and multi-objective evolutionary algorithms for classificatory signal decomposition. In Slazek and Lingras: *Rough Computing: Theories, Technologies, and Applications* 2007 204–27.

## P25 PumpHCO-db: A database of half-center oscillator computational models for analyzing the influence of Na^+^/K^+^ pump on the bursting activity

### Anca Doloc-Mihu, Ronald L. Calabrese

#### Department of Biology, Emory University, Atlanta, GA, 30322, USA

##### **Correspondence:** Anca Doloc-Mihu (adolocm@emory.edu)

*BMC Neuroscience* 2017, **18(Suppl 1)**:P25

Rhythmic behaviors such as walking or breathing are controlled by networks of neurons that produce rhythmic bursting activity, called central pattern generator (CPG). These CPG neurons depend upon a Na^+^/K^+^ pump to maintain the ionic gradients that establish the resting potential and thus support other ionic currents. However, how the Na^+^/K^+^ pump, which produces an outward net current proportional to its activity, directly influences bursting activity is not yet fully understood. Here, we use a half-center oscillator (HCO) (two mutually inhibitory neurons) mathematical model [1] that includes a Na^+^/K^+^ pump to replicate the electrical activity (rhythmic alternating bursting of mutually inhibitory interneurons) of the leech heartbeat CPG under a variety of experimental conditions. Our study here is preliminary to a full investigation of the role of the Na^+^/K^+^ pump in the robust maintenance of functional bursting activity. For this study, we used the mathematical model of Kueh et al. [1] of a HCO, which consists of a pair of reciprocally inhibitory model neurons, with each individual leech heart interneuron being represented as a single isopotential electrical compartment with Hodgkin and Huxley type intrinsic membrane and synaptic conductances. In this study, the HCO model has eight currents with voltage-dependent conductances including two types of inhibitory synaptic currents, spike mediated and graded. This HCO model also includes a Na^+^/K^+^ pump current that tracks changes in intracellular Na^+^ concentrations that occur as a result of the Na^+^ fluxes carried by ionic currents. The Na^+^/K^+^ pump exchanges two K^+^ ions for three Na^+^ ions, its activity and hence its current has a sigmoidal dependence on intracellular Na^+^ concentrations. Na^+^ currents include the fast spiking current (I_Na_) and a persistent Na^+^ current (I_P_). All model equations are given in Kueh et al. [1]. The 8–9 order Prince-Dormand method from the GNU Scientific Library (www.gnu.org/software/gsl) was used to integrate the model’s differential equations. To explore systematically the parameter space of this HCO and corresponding isolated neuron models, we used the brute-force approach. We varied selected parameters in both neurons in all combinations possible: the maximal conductances of the persistent Na^+^ (I_P_), slow Ca^2+^, leak, hyperpolarization-activated (I_h_), and persistent K^+^ currents, across of 50, 75, 100, 125, and 150 percent of their canonical values (see [1]), the leak reversal potential across −66.25, −62.5, −58.75, −55, and −51.25 mV, the half-activation of the Na^+^/K^+^ pump across −2, −1, 0, 1, and 2 mV, the maximum Na^+^/K^+^ pump current across 0.38, 0.41, 0.44, 0.47, and 0.5 nA, and the slope coefficient across 90, 95, 100, 105, and 110 percent of its canonical value. The resulting parameter space includes 100 million simulated models. After changing a parameter, a model was run for 200 s to allow the system to establish stable activity, and then, it was run for another 40 s, from which the data were recorded and analyzed. We then classified these HCO and isolated (synaptic currents equal zero) neuron model simulations by their activity characteristics, so that models showing the same electrical activity are segregated to the same group. Of particular interest to us, is the group of bursting simulated models, which was further split into realistic and non-realistic HCOs [3]. We built a relational database PumpHCO-db with the resulting model characteristics similar to our previous work [2]. Our ongoing studies use this database to ask fundamental questions about how realistic HCO [3] activity is influenced by the Na^+^/K^+^ pump. We will be particularly interested in parameter changes that correspond to known neuromodulations such as the modulation of I_h_ and maximal Na^+^/K^+^ pump current by myomodulin [4].

**Acknowledgements**

Work supported by the National Institute Health Grant R01 NS085006 to R.L.Calabrese.

**References**

1. Kueh D, Barnett WH, Cymbalyuk GS, Calabrese RL: Na^+^/K^+^ pump interacts with the *h*-current to control bursting activity in central pattern generator neurons of leeches. *eLife*: 2016; **5:**e19322.

2. Doloc-Mihu A, Calabrese RL: A database of computational models of a half-center oscillator for analyzing how neuronal parameters influence network activity. *J Biol Physics* 2011, **37**:263–283.

3. Doloc-Mihu A, Calabrese RL: Analysis of family structures reveals robustness or sensitivity of bursting activity to parameter variations in a half-center oscillator (HCO) model. *eNeuro* 2016, **3(4):** ENEURO.0015-16.2016.

4. Tobin AE, Calabrese RL: Myomodulin increases I_h_ and inhibits the Na/K pump to modulate bursting in leech heart interneurons. *J Neurophysiol.* 2005, **94:**3938–3950.

## P26 Encoding of memories: effective connectivity on the hippocampus and the role of inhibition in the information flow

### Víctor J. López-Madrona^1^, Fernanda S. Matias^2^, Ernesto Pereda^3^, Claudio R. Mirasso^4^, and Santiago Canals^1^

#### ^1^Instituto de Neurociencias, Consejo Superior de Investigaciones Científicas, Universidad Miguel Hernández, Sant Joan d’Alacant 03550, Spain; ^2^Instituto de Física, Universidade Federal de Alagoas, Maceió, Alagoas 57072-970, Brazil; ^3^Departamento de Ingeniería Industrial, Escuela Superior de Ingeniería y Tecnología, Universidad de La Laguna Avda. Astrofísico Fco. Sanchez, s/n, La Laguna, Tenerife 38205, Spain; ^4^Instituto de Física Interdisciplinar y Sistemas Complejos, CSIC-UIB, Campus Universitat de les Illes Balears E, 07122 Palma de Mallorca, Spain

##### **Correspondence:** Víctor J. López-Madrona (v.lopez@umh.es)

*BMC Neuroscience* 2017, **18(Suppl 1)**:P26

Networks containing a huge number of neurons and synapses confer the brain an immense computational capability. Learning how activity propagates in these intricate networks would help us understand how information is globally integrated. Only then we could try to understand, how perception and its unitary nature emerges from a multisensory experience or how complex memories are formed. Activity propagation in the system is determine by the structural connections (wiring diagram) linking the different nodes in the network and, importantly, by the functional interactions between the different nodes. These interactions are highly dynamic processes that mostly relay on changes in synaptic efficacy and the differential recruitment of excitatory and inhibitory elements (the excitation/inhibition balance). The combination of both factors, the wiring diagram and the dynamic functional properties of the connections, determine the effective connectivity of the system in a particular moment or state. Here we have used a computational model and causality measurements to study activity propagation in the hippocampal formation, a brain region critical for the formation of episodic memories. It is composed by the hippocampus proper (areas CA1 and CA3), the dentate gyrus (DG) and the entorhinal cortex (EC). While extensive literature on the connectivity of the first regions exist, the connectivity of the EC remains poorly investigated.

To better understand how the internal structure of EC affects the causality of information flow in the hippocampal formation, we implemented a model containing all the above areas. We assumed the EC was formed by 3 layers (II, III and V). We fixed all connections in the model between DG, CA3 and CA1, while the EC connectivity was systematically varied. The effective connectivity was estimated using Granger Causality (GC) and Partial Transfer Entropy (PTE). For these measurements, we assumed that only information from DG, CA3 and CA1 was available, as commonly happens in experiments. We also introduced interneurons in our circuit, considering inhibitory projections from CA1 to CA3. With this new ingredient, we addressed different “causality” measures, such as information flow and synchronization between populations for excitatory and inhibitory effective connections, respectively.

**Conclusions:** Our procedure revealed that different EC internal connectivity patterns give rise to very distinct causality results in the hippocampus, despite its fixed connectivity. Moreover, different results were obtained for the two methods (GC, PTE), highlighting the importance of the analysis and revealing potential misinterpretations when only partial information is available. Our method allowed us to analyze the differences of causality when excitatory and inhibitory projections are considered and identified the most probable EC configuration to explain the known connectivity between the DG, CA3 and CA1.

## P27 Extended generalized leaky integrate and fire neuron for cerebellum modeling

### Alice Geminiani^1^, Alessandra Pedrocchi^1^, Egidio D’Angelo^2^, Claudia Casellato^1^

#### ^1^NEARLab, Department of Electronics, Information and Bioengineering, Politecnico di Milano, Milan 20133, Italy; ^2^Department of Brain and Behavioral Sciences, University of Pavia, Pavia 27100, Italy

##### **Correspondence:** Alice Geminiani (alice.geminiani@polimi.it)

*BMC Neuroscience* 2017, **18(Suppl 1)**:P27

Simplified but realistic neuron models are useful for investigating the emergent properties of neural circuits in large-scale simulations and the role of specific neuron dynamics in efficient signal transmission, for behavior generation. Here we extend a generalized leaky integrate-and-fire (GLIF) model [1] so to produce an enriched variety of spiking responses. We developed the GLIF point-neuron model in PyNEST using NESTML [www.nest-initiative.org], adding in the state and update equations: i) spike generation stochasticity, ii) threshold dynamics depending on membrane voltage dynamics, iii) spike-triggered hyperpolarizing current with update constant depending on the input current (I_in_). The model couples time-dependent and event-driven algorithmic components; it can be tuned to generate autorhythm, specific slope between response frequency and input current (f-I_in_), spike-frequency adaptation increasing with I_in_, AfterHyperPolarization (AHP) duration increasing with I_in_, firing irregularity (CV of inter-spike-intervals), phase reset, and post-inhibitory rebound bursting. In particular, we focus on the specific electrophysiological properties of cerebellar cells (Golgi - GoC, Granular, Purkinje, Inferior Olive and Deep Nuclei neurons). After fixing some parameters with direct biological values, we tune the others to reproduce cell-specific behaviors. We implement a protocol injecting a sequence of I_in_ steps (excitatory and inhibitory) of different amplitudes and durations (Figure 1). For the GoC, we get f-I_in_ = 0.24 Hz/pA, and CV = 0.034 (on the long excitation step, exc_3_). The tuned model reproduces the typical electroresponsiveness of a GoC in vitro [2] (Figure 1).
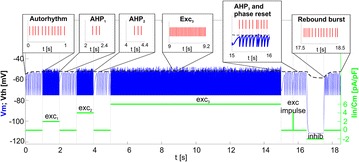


**Figure 1.** Membrane voltage (V_m_, in blue) of GoC model, threshold voltage (V_th_, black dashed line), along the 18.5 s of protocol (green steps of I_in_ on membrane Capacitance, C_m_). Insets show the produced spikes (red vertical lines) and associated properties

After automatic parameter optimization, we will create an in vivo cerebellum microcircuit by connecting the differentiated cell populations, through plastic synapses (this GLIF model includes also presynaptic spikes with voltage-dependent conductances). Motor learning skills will be tested by closed-loop sensorimotor tasks. Therefore, the tool allows to reliably reproduce specific alterations of neuron mechanisms and the consequent misbehaviors.

**Acknowledgements**

This work was supported by EU grant Human Brain Project (HBP 604102).

**References**

1. Mihalaş S and Niebur E.: A Generalized Linear Integrate-and-Fire Neural Model Produces Diverse Spiking Behaviors. *Neural Comput*. 2009; **21(3):** 704–718

2. D’Angelo E et al.: Modeling the Cerebellar Microcircuit: New Strategies for a Long-Standing Issue. *Front. Cell. Neurosci.* 2016; **10**:176.

## P28 Saccade Velocity Driven Oscillatory Network Model of Grid Cells

### Ankur Chauhan^1^,KarthikSoman^1^, V. Srinivasa Chakravarthy^1^

#### ^1^Department of Biotechnology, Indian Institute of Technology Madras, Chennai, Tamilnadu, India

##### **Correspondence:** V. Srinivasa Chakravarthy (schakra@iitm.ac.in)

*BMC Neuroscience* 2017, **18(Suppl 1)**:P28

Grid cells in the EntorhinalCortex (EC), one of the key neural correlates for spatial navigation in rodents and primates[1], have been recently reported to have a role in saccadic movement encoding also [2]. Experimental studies corroborated this by analyzing the characteristic hexagonal firing fields of neurons in EC of head fixed monkeys, as the animals scanned natural images displayed in front of them. Here, we present Saccade Velocity Driven Oscillatory Network (SVDON) model that captures the responses of grid cells to saccadic trajectories. SVDON is an extension of VDON model which was previously used for modeling the grid cell in actual spatial navigation [3].

SVDON has basically four stages viz: Saccade Generation (SG), Saccade Direction (SD) encoding, Path Integration (PI), and Spatial Cell (SC) stages respectively (Fig. A). SG was implemented using a saliency map based bottom-up attention model that selectively attends to the salient locations of an image depicting a natural scene [4]. Once the saccade trajectory was built on the image, velocity vectors at each point were computed. These velocity vectors were forward passed to the SD layer, an array of neurons, where each neuron had its own preferred direction. EC was experimentally reported to have neurons with tuned responses to saccade direction and called as SD cells [5]. SD layer response was passed on to the PI layer which had a one to one connection with the SD layer. Each neuron in the PI layer was a phase oscillator such that the SD response modulated the frequency of it and hence the saccade position information along that direction component was encoded as the phase of the respective oscillator. Final SC layer was an unsupervised neural layer that extracted the principal components of the oscillatory responses. Remapping the SC neuron activity on the saccade trajectory exhibited grid cell like periodicities including hexagonal firing fields (Fig. B). Further computation of the hexagonal gridness score (HGS) confirmed this result.
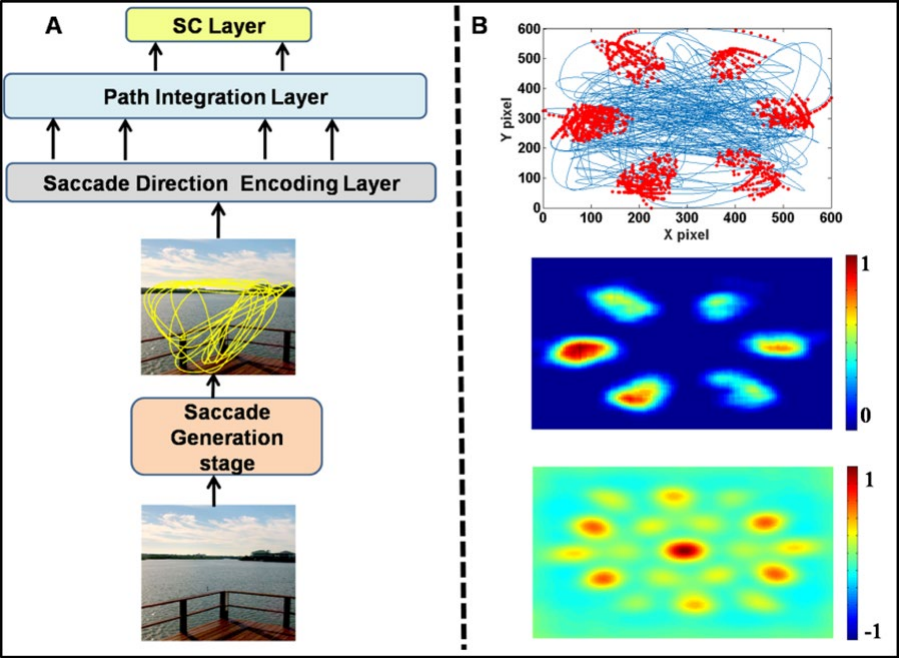


**Figure 1. A.** SVDON model architecture **B.** Firing field (Top), Firing Rate map (Middle) and Autocorrelation map (Bottom) of grid neuron in the SC layer. HGS score is 0.6971 (If HGS > 0 it qualifies as hexagonal grid cell)

**References**

1. Hafting T, Fyhn M, Molden S, Moser M-B, Moser EI: Microstructure of a spatial map in the entorhinal cortex. *Nature* 2005, **436**(7052):801–806.

2. Killian NJ, Jutras MJ, Buffalo EA: A map of visual space in the primate entorhinal cortex. *Nature* 2012, **491**(7426):761–764.

3. Soman K, Muralidharan V, Chakravarthy S: An oscillatory network model of head direction, spatially periodic cells and place cells using locomotor inputs. *bioRxiv* 2016:080267.

4. Walther D, Koch C: Modeling attention to salient proto-objects. *Neural networks* 2006, **19**(9):1395–1407.

5. Killian NJ, Potter SM, Buffalo EA: Saccade direction encoding in the primate entorhinal cortex during visual exploration. *Proceedings of the National Academy of Sciences* 2015, **112**(51):15743–15748.

## P29 Programmed cell death in substantia nigra due to subthalamic nucleus-mediated excitotoxicity: a computational model of Parkinsonian neurodegeneration

### Vignayanandam R Muddapu^1^, Srinivasa V. Chakravarthy^1^

#### ^1^Bhupat and Jyoti Mehta School of Biosciences, Department of Biotechnology, IIT-Madras, Chennai, TN, India

##### **Correspondence:** Srinivasa V. Chakravarthy (schakra@iitm.ac.in)

*BMC Neuroscience* 2017, **18(Suppl 1)**:P29

Parkinson’s disease (PD) is a neurodegenerative disease with an estimated 6 million people are affected worldwide. It is caused by the loss of dopaminergic neurons in the substantia nigra pars compacta (SNc), though the exact cause of the cell death is not clear. One hypothesis about the cause of SNc death, known as the “subthalamic nucleus-mediated excitotoxicity theory” [1] states that the dopamine deficiency in SNc leads to disinhibition and overactivity of the subthalamic nucleus (STN) which, in turn causes excitotoxic damage to their target structures, including the SNc itself. In order to investigate this hypothesis, we built a computational spiking network model of SNc-STN loop along with STN-GPe loop. The model aims to capture the underlying dynamics during the overactivity of STN and study excitotoxicity caused by it in SNc. All the nuclei are modeled as Izhikevich 2D neurons (Figure 1A). The model was tuned and simulated for normal and PD conditions characterized by loss of SNc cells. We incorporate a mechanism of programmed cell death, whereby a SNc cell under high stress (compared to an apoptotic threshold) kills itself. Stress on a given SNc cell was calculated based on mean firing history of the cell – higher firing activity leads to higher stress. Under normal conditions, the loop interactions between SNc and STN are such that, the stress levels in SNc do not exceed the apoptotic threshold, and therefore the SNc cells survive. But if a critical number of SNc cells die for some reason, the reduced SNc size leads to disinhibition of STN, which becomes overactive, due to which some of the SNc cells become overactive and die by programmed cell death. Thus, the initial loss of SNc cells leads to a runaway effect, leading to an uncontrolled loss of cells in the SNc, characterizing the underlying neurodegeneration of PD.

The simulation results obtained from the proposed model in normal and PD conditions provided important insights regarding excitotoxicity in SNc. Firstly, when the connection from SNc to STN were introduced (at t = 0 s), synchrony in the STN network decreased (Figure 1Bb, 0 to 10 s) which was observed in normal physiological condition [2]. A cell in SNc is “killed” whenever its stress variable crosses a “stress threshold”. When the stress threshold = 11.5, all the SNc neurons survive. To emulate PD condition in our model, stress threshold was lowered from 11.5 to 11.3 at t = 10 s, which triggers a steady and uncontrolled loss of SNc cells (Figure 1Bb roughly 17 s onwards). The synchrony in STN network begins to increase only when more than 50% of the SNc cells are lost (Figure 1Bb, roughly 40 s onwards). The proposed model was able to exhibit STN-mediated excitotoxicity in SNc. The connection strength from GPe to STN can be used as a parameter to delay or to hasten the rate of cell loss. In future work, we will investigate if deep brain stimulation to STN can slow down the progression of cell loss in PD condition.
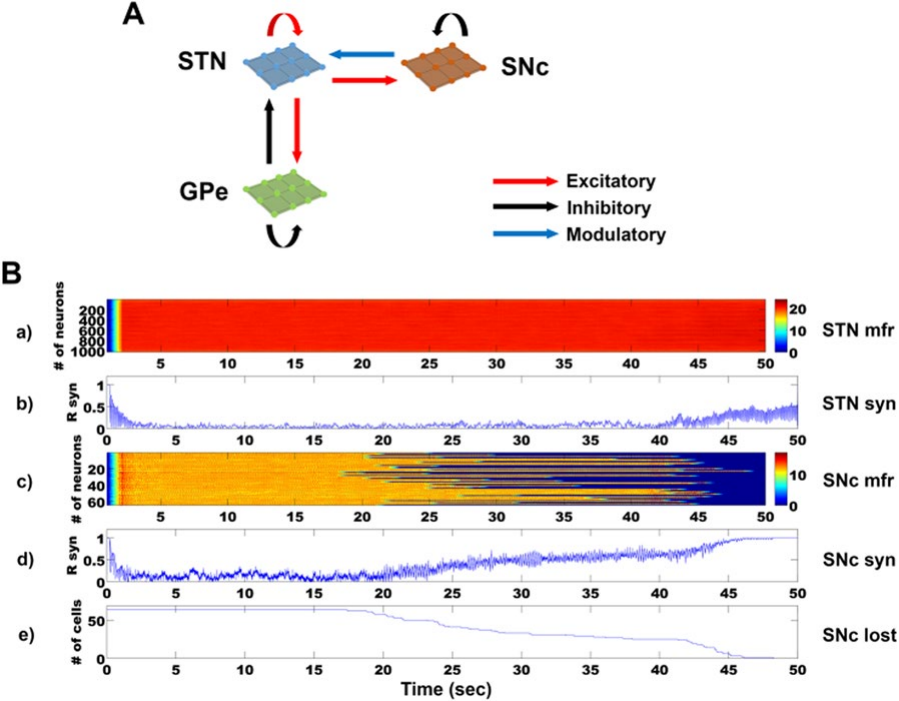


**Figure 1. A.** The model architecture. **B.** Simulation plot (50 s) shows **(a, d)** mean firing rate (mfr) and **(b, d)** synchrony (syn) measure for STN and SNc population. **(e)** subplot shows number of SNc cell lost during the simulation

**References**

1. Rodriguez MC, Obeso JA, Olanow CW: Subthalamic nucleus-mediated excitotoxicity in Parkinson’s disease: a target for neuroprotection. *Ann Neurol* 1998,**44(Suppl 1)**:S175–S188.

2. Cragg SJ, Baufreton J, Xue Y, Bolam JP, Bevan MD: Synaptic release of dopamine in the subthalamic nucleus. *Eur J Neurosci* 2004,**20**:1788–1802.

## P30 A novel approach for determining how many distinct types of neurons are in the Drosophila brain by sequencing neural structure

### Chao-Chun Chuang, Nan-yow Chen

#### National center for high-performance computing, Hsinchu, Taiwan

##### **Correspondence:** Chao-Chun Chuang (summerhill001@gmail.com)

*BMC Neuroscience* 2017, **18(Suppl 1)**:P30

The brain can be divided into two parts, “hardware” and “software”. Hardware refers to the constructed between nerve cells networks, and software refers to the neuronal connectomes by gene expression in nerve cells. For hardware parts of brain, we have constructed a three-dimensional single-cell database of Drosophila brain (FlyCircuit), about 30,000 neurons now. For software parts of brain, we will focus on mapping the neural connections and pathway in the Drosophila brain. To define how many distinct types of neurons are in the Drosophila brain will provide us a useful way to solve this problem. There are many way to define the different types of neurons. Obvious categories include structural differences in shape and positioning of dendrites and axons. However, they are too difficult and complicated. In the current study, we will analyze about 30,000 neurons in Flycircuit. For high-speed connectomics analysis of neuron morphology, algorithms adapted from those used in protein structure studies were developed to represent 3D neuron morphology as 1D sequence. We applied this method on Drosophila neuron 3D structure to show a sequential neuropilar pathway (global neurite structure sequence), and a voxel distribution within neuropilar subdomains for neurites (local neurite structure sequence). Then in a constructed relative framework, each neuron will be defined with a specialized digital code according to its class (Figure 1A), family with global neurite structure sequence (Figure 1B), and type with local neurite structure sequence (Figure 1C). Then we can use these codes to classify how many specialized types of neurons are in the Drosophila brain. Finally, the standardization of neurite structure sequence can handle massive 3D neuronal image data collected in experiments from different research groups as well as manage bio-images with deeper neurological insight.
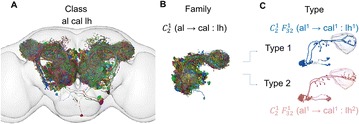


**Figure 1.** We applied this method on *Drosophila* neuron 3D structure to show a sequential neuropilar pathway (global neurite structure sequence), and a voxel distribution within neuropilar subdomains for neurites (local neurite structure sequence)

**References**

1. Lin, Chih-Yung, et al.: A comprehensive wiring diagram of the protocerebral bridge for visual information processing in the *Drosophila* brain.” *Cell reports* 2013, **3.5**: 1739–1753.

2. Chiang, Ann-Shyn, et al. Three-dimensional reconstruction of brain-wide wiring networks in *Drosophila* at single-cell resolution. *Current Biology* 2011, **21.1**: 1–11.

3. Jefferis, G.S. et al. Comprehensive maps of *Drosophila* higher olfactory centers: spatially segregated fruit and pheromone representation. *Cell* 2007, **128**: 1187–1203.

## P31 Generating sequences in recurrent neural networks for storing and retrieving episodic memories

### Mehdi Bayati^1,2^, Jan Melchior^1^, Laurenz Wiskott^1^, Sen Cheng^1,2^

#### ^1^Institut für Neuroinformatik, Ruhr-Universität Bochum, D-44801 Bochum, Germany; ^2^Mercator Research Group ‘Structure of Memory’, Ruhr-University Bochum, Bochum, Germany

##### **Correspondence:** Mehdi Bayati (bmehdi5@gmail.com)

*BMC Neuroscience* 2017, **18(Suppl 1)**:P31

It has been suggested that the reliable propagation and transformation of neural activity within and between different brain regions is crucial for neural information processing. Furthermore, temporal sequences of neural activation have recently been proposed to play an important role in the explanation of the function of hippocampal neural circuits in episodic memory, our memory of experienced events in our lives [1]. One central feature of CRISP is that hippocampal area CA3, because of its abundant recurrent connections, intrinsically produces temporal sequential activities. In this project, first we review the possible mechanisms by which a relatively fixed recurrent network structure (as a model of CA3) can generate neural activity sequences intrinsically. Next, we implement the CA3 models in a complete framework of cortico-hippocampal circuits (We use EC-CA3-CA1-EA network), each subregion has certain function based on CRISP theory. During memory encoding, intrinsic CA3 sequences are hetero-associated with sequences that are driven by sensory inputs. Later on, sequences in CA3 are hetero-associated with the sequence in CA1 and finally, the CA1 activities are hetero-associated with sensory inputs in the EC. During memory retrieval, intrinsic CA3 sequences have to be reactivated based on partial, noisy cues which is provided to EC. Finally, the retrieved sequences in CA3 reactivate the initial input sequences in EC via CA1 layer. Memory performance is determined by the network’s ability to perform sequence completion. If the network’s output is more similar to the original sequence, then the network has done some amount of sequence recall. As a measure for similarity we use the Pearson correlation coefficient between the corresponding patterns of the originally stored and retrieved sequences in different layers. Overall, we find that the neural network mechanism in CA3 generating the sequences has to be robust to noise in the triggering cue. On the other hand, less temporal-correlated patterns in CA3 give rise to more confidence in retrieving the sequence in a complete framework. To conclude, we find that using the right model in CA3, CRISP model surprisingly retrieves almost correctly the stored sequences up to moderate noise levels.

**Acknowledgements**

This work was supported by the grants (SFB 874, projects B2 and B3) from the German Research

Foundation (Deutsche Forschungsgemeinschaft, DFG) and a grant from the Stiftung Mercator.

**Reference**

1. Cheng S, The CRISP theory of hippocampal function in episodic memory. Frontiers in Neural Circuits, 2013, **7:**88.

## P32 Modeling replay and theta sequences in a 2-d recurrent neural network with plastic synapses

### Amir Hossein Azizi^1^, Kamran Diba^2^, Sen Cheng^1^

#### ^1^Institut für Neuroinformatik, Ruhr University Bochum (RUB), Bochum, 44801, Germany; ^2^Psychology faculty, university of Wisconsin-Milwaukee, Milwaukee, WI 53201, USA

##### **Correspondence:** Amir Hossein Azizi (amir.azizi@rub.de)

*BMC Neuroscience* 2017, **18(Suppl 1)**:P32

During immobility awake states or when rats are asleep, place cells are reactivated in a sequential order [1]. This reactivation co-occurs with sharp wave/ripples (SWR) in the local field potential (LFP) in the hippocampus. These *replay* sequences reflect the sequence of the animal’s prior spatial behaviour or the upcoming trajectory to a goal location. During running, the LFP shows characteristic theta oscillations, whose phase modulates the activity of place cells in addition to the location of the animal. This joint modulation, called *phase precession*, results in the activity of place cells occurring in a sequential order within a theta cycle. The causal relationship between phase precession and theta sequences remains unclear. One possibility is that phase precession leads to sequential ordering within theta cycles. Alternatively, phase precession might be the result of the directional activation of a group of cells with overlapping place fields. For instance, Romani and Tsodyks recently modelled phase precession using an unstable moving bump of activity in a 1-d continuous attractor neuronal network [2]. The driving force of the sequential activity is the short-term plasticity in the synaptic connections. This model also generates offline replay activity in a different operating mode. Since no long-term plasticity was included in the model, the resulting replay and theta sequences only reflected the recent behavior of the animal within the last few seconds and the associated span of phase precession was limited. Recent studies, however, point to a separation of phase precession and theta sequences. Although phase precession can be found immediately in novel environment, the development of theta sequences requires experience [3] and the goal location, rather than the extent of phase precession, appears to determine the length of theta look-ahead sequences [4]. Furthermore, a recent study suggests a dissociation between replay and theta sequences [5]. Only SWR-associated replay activity included portions of an environment that the animal had learned to avoid, while theta sequences did not penetrate into the avoided region. Here we study phase precession, theta sequences, replay activity, and the relationship between these phenomena in a 2-d continuous attractor network model. The units in the network exhibit spike-frequency-adaptation that destabilizes the bump attractor and synapses with long-term plasticity. This model can generate enhanced replay after exposure, theta sequences, and phase precession. The spatial extent of theta sequences is controlled by the running speed of the virtual animal (Figure 1) as hypothesized by Wu et al. [5]. Our preliminary findings suggest that replay and theta sequences can be accounted for within a single model.
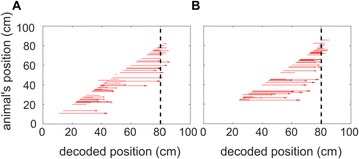


**Figure 1.** Theta sequences reflect the goal location. The decoded location of the animal in each theta cycle is indicated by arrows starting from the current location of the animal. The arrowheads show the direction of the sequential activity. **A**. When the animal runs slowly, decoded sequences do not reach the avoided region (80–100 cm). **B**. When the animal runs faster, decoded theta sequences reflect trajectories into the avoided region

**References**

1. Diba K, Buzsáki G. Forward and reverse hippocampal place-cell sequences during ripples. *Nat. Neurosci.* 2007; **10:**1241–2.

2. Romani S, Tsodyks M. Short-term plasticity based network model of place cells dynamics. *Hippocampus* 2015; **25:**94–105.

3. Feng T, Silva D, Foster DJ. Dissociation between the Experience-Dependent Development of Hippocampal Theta Sequences and Single-Trial Phase Precession. *J. Neurosci.* 2015; **35:**4890–902.

4. Wikenheiser AM, Redish AD. Hippocampal theta sequences reflect current goals. *Nat. Neurosci.* 2015; **18:**289–94.

5. Wu C-T, Haggerty D, Kemere C, Ji D. Hippocampal awake replay in fear memory retrieval. *Nat. Neurosci*. 2017, in press

## P33 Biophysically detailed model of cortical activity in response to moving gratings

### Elena Y. Smirnova^1,2^, Elena G Yakimova^3^, Anton V. Chizhov^1,2^

#### ^1^Ioffe Institute, St.-Petersburg, 194021, Russian Federation; ^2^Sechenov Institute of Evolutionary Physiology and Biochemistry of RAS, St.-Petersburg, 194223, Russian Federation; ^3^Pavlov Institute of Physiology, St.-Petersburg, 199034, Russian Federation

##### **Correspondence:** Elena Y. Smirnova (elena.smirnova@mail.ioffe.ru)

*BMC Neuroscience* 2017, **18(Suppl 1)**:P33

Description of the mechanisms of visual feature selectivity of cortical neurons is still in development. We propose a model that implements a mechanism of direction selectivity (DS) of the primary visual cortex (V1) neurons into our previous model of 2-d distributed neuronal populations in V1, selective to orientation of stationary gratings [1]. The model is based on the conductance-based refractory density approach which provides both a biophysically detailed description of neuronal populations in terms of ionic channel conductances for one- or two-compartment neurons and good precision for statistically equilibrium and non-equilibrium regimes of ensemble activity. Coupled excitatory and inhibitory neurons interact via glutamatergic and GABAergic synapses. Here, we extend this model by supplying a filter-based description of retinothalamic visual signal processing [2]. The mechanism of DS is based on asymmetrical projections from lagged and non-lagged thalamic neurons to the cortex [3], such that V1 neurons preferring a certain direction receive a non-lagged input from one side of its thalamic footprint and a lagged input from the other side. Our model realistically reproduces membrane potential, firing rate, synaptic conductances etc. in response to moving gratings. Simulations shows that the implemented mechanism of DS provides only moderate direction tuning of the time-varying characteristics averaged over the population, however the DS is clearly observed in maps of time-averaged activity, similar to experimental evidences obtained by optical imaging. The time-averaged activity of inhibitory neurons is not selective to stimulus direction, as well as the time-averaged input to the cortex. The results demonstrate how DS maps can originate from the thalamic input that is transiently selective to direction but non-selective on average in time.

**Acknowledgements**

This work was supported by the Russian Foundation for Basic Research (project 15-04-06234).

**References**

1. Chizhov AV: Conductance-based refractory density model of primary visual cortex. *J Comput Neurosci* 2014, **36:** 297–319.

2. Dayan P, Abbott LF: Theoretical neuroscience: computational and mathematical modeling of neural systems. *The MIT Press* 2001.

3. Vigeland LE, Contreras D, Palmer LA: Synaptic mechanisms of temporal diversity in the lateral geniculate nucleus of the thalamus. *J Neurosci* 2013, **33(5)**: 1887–1896.

## P34 *NeuriteSLIM* – Shrink the Neuro Fibers for Visualization the Connectome

### Nan-Yow Chen^1^, Chi-Tin Shih^2^, Chao-Chun Chuang^1^

#### ^1^High Performance Computing Division, National Center for High‐Performance Computing, Hsinchu, Taiwan; ^2^Department of Applied Physics, Tunghai University, Taichung, Taiwan

##### **Correspondence:** Nan-Yow Chen (nanyow@nchc.narl.org.tw)

*BMC Neuroscience* 2017, **18(Suppl 1)**:P34

Connectome assembled through fluorescent imaging is regarded as an important step to understand how brains work [1]. However, due to the spreading feature of fluorescent images, the fibers of the neurons were larger than their actual sizes. Consequently, the number of neurons which could be simultaneously visualized in the standard brain was limited because signals from different neurons would mix together and the neurons became indistinguishable. For constructing visualization of connectome, we develop an algorithm called *NeuriteSLIM* which could shrink the neurite thickness with preserving their length, shape and radial intensity distribution. With this tool, we can reconstruct and visualize the *Drosophila* connectome at the single-cell resolution and provide a useful tool for connectome studying in the future.

The goal of *NeuroSLIM* was to shrink the fiber thickness with preserved cross-sectional shape and intensity distribution of the fibers. At first, voxels were divided into $n_{x} \times n_{y} \times n_{z}$ smaller, nearly cubic voxels with the same intensity as in the original voxel. The next step was to identify the nearest central point for each voxel. (As shown in Figure A and B). The intensity of each voxel was then moved from the original position to the shrunk position, which was the voxel in the cross section closest to the interpolated point according the desired shrink ratio between the original position and the central point. If more than one original voxels were moved to the same new voxel, the intensity of the new voxel would be the averaged intensity of the original voxels. Figure C showed the shrunken result of Figure A. Figure D showed the case with 120 neurons warped into the standard model brain [2] and provided a global connectomic view of the *Drosophila* brain. For such a large number of neurons packed in the same brain, the neurites were inevitably mixed together. *NeuroSLIM* shrunk the radii of the neurites and provided a clearer connectomic visualization in Figure E.
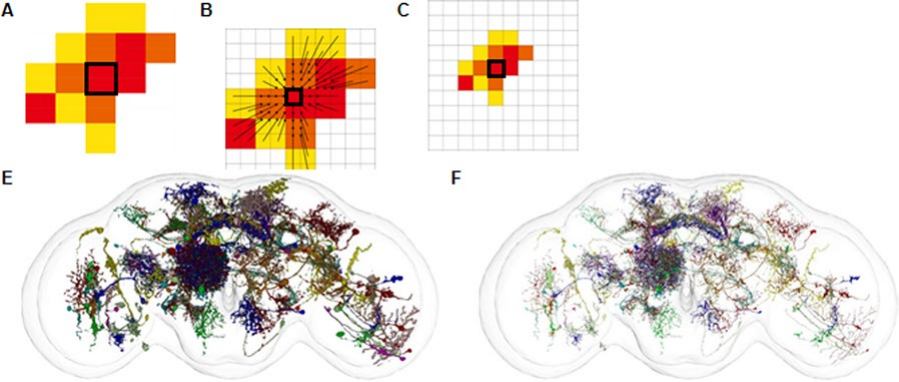


**Figure 1. A-C.** indicate the shrinking algorithm of *NeuriteSLIM*. **E-F.** show the results of 120 neurons before and after applying *NeuriteSLIM*

**References**

1. Alivisatos, A.P. et al.: The Brain Activity Map Project and the Challenge of Functional Connectomics. *Neuron* 2012, **74**: 970–974.

2. Ann-Shyn Chiang, et al.: Three-Dimensional Reconstruction of Brain-wide Wiring Networks in Drosophila at Single-Cell Resolution. *Current Biology* 2010, **21**: 1–11.

## P35 Identification of models of sensory neural circuits consisting of a nonlinear filter in series with a leaky integrate-and-fire neuron

### Dorian Florescu, Daniel Coca

#### Department of Automatic Control and Systems Engineering, University of Sheffield, Sheffield, South Yorkshire, S1 3JD, UK

##### **Correspondence:** Daniel Coca (d.coca@sheffield.ac.uk)

*BMC Neuroscience* 2017, **18(Suppl 1)**:P35

The early sensory processing circuits in the brain, incorporating sensory neurons and downstream spiking neurons, have often been represented as cascade models consisting of a linear or nonlinear filter [1,2] followed by model of spike generation such as a Poisson or integrate-and-fire model. The cascade model is usually inferred directly from experimental measurements using system identification methods.

Although the leaky integrate-and-fire (LIF) neuron model is much simpler than a biophysically realistic Hodgkin-Huxley model, the LIF model has been used successfully to predict experimentally recorded spike trains [3]. The identification of a linear filter in cascade with a Hodgkin-Huxley neuron has been considered in [1] by assuming prior knowledge of the spiking neuron parameters or assuming that measurements of the input to the spiking neuron input are available. A number of methods are available to estimate a cascade model consisting of a linear filter in series with a LIF neuron (LF-LIF). In [3] this involves maximizing the likelihood of observed spike responses to a stochastic visual stimulus, assuming that the threshold parameter is known, whilst in [4] the parameters of an input-output equivalent model are estimated by assuming that the LIF parameters are known a priori. Here we propose for the first time a method to identify a cascade model consisting of an arbitrary nonlinear filter in series with a leaky integrate-and-fire neuron model where both the parameters of the LIF neuron and the structure and parameters of the nonlinear filter are unknown. Furthermore, the input of the spiking neuron is assumed to be corrupted by Gaussian white noise and not available for measurement.

A new input-output equivalent representation of the circuit is proposed in which one of the parameters represents the minimum step amplitude required to trigger a response of the NF-LIF circuit. By analogy to the rheobase of a biological neuron we call this parameter the rheobase of the NF-LIF circuit. The identification of the NF-LIF circuit has two stages. The LIF model parameters are estimated first followed by the identification of the nonlinear filter. In order to estimate the LIF parameters, we derive the theoretical steady state firing rate output of the NF-LIF in response to a step input of a given amplitude. For this estimation stage only, the filter is approximated with a linear filter. Subsequently, we fit this theoretical output to noisy measurements of the circuit in response to repeated step inputs with different amplitudes, using the Levenberg-Marquardt algorithm. Using the experimentally observed rheobase as an initial parameter guess increases significantly the performance of the algorithm. Once the LIF parameters are estimated, an orthogonal forward selection algorithm is used to identify the NARMAX model of the scaled nonlinear filter based on the nonlinear filter input and the reconstructed filter output i.e. the reconstructed input to the LIF neuron.

**References**

1. Lazar AA: Population encoding with Hodgkin–Huxley neurons. *IEEE Transactions on Information Theory* 2010 **56(2)**:821–37.

2. Lazar AA, Slutskiy YB: Spiking neural circuits with dendritic stimulus processors. *Journal of computational neuroscience* 2015 **38(1)**:1–24.

3. Pillow JW, Paninski L, Uzzell VJ, Simoncelli EP, Chichilnisky EJ: Prediction and decoding of retinal ganglion cell responses with a probabilistic spiking model. *Journal of Neuroscience* 2005, **25(47)**:11003–13.

4. Lazar AA, Slutskiy Y: Identifying dendritic processing. *Advances in neural information processing systems* 2010:1261–1269.

## P36 Modelling fluctuations in resting-state functional connectivity in epilepsy

### Julie Courtiol^1^, Spase Petkoski^1^, Viktor K Jirsa^1^

#### ^1^Aix Marseille Univ, Inserm, INS, Institut de Neurosciences des Systèmes, Marseille, France

##### **Correspondence:** Julie Courtiol (julie.courtiol@univ-amu.fr)

*BMC Neuroscience* 2017, **18(Suppl 1)**:P36

Understanding the mechanisms behind epilepsy is one of the most challenging problems in neuroscience. Recent efforts provided valuable evidence that epileptic activity involves widespread brain networks rather than single sources [1] and that these networks contribute to epilepsy based interictal brain alterations [2]. In order to better understand the underlying alterations of the functional connectivity (FC), we propose a whole-brain computational modelling approach of resting-state using patient-specific structural connectivity, with clinically diagnosed bitemporal epilepsy, derived from diffusion tensor imaging (DTI), and generic 2D oscillator for the node intrinsic activity. From the model, we systematically alter the neural excitability of the nodes in healthy control subjects to progressively incorporate a propagation and epileptogenic zone (EZ) according to clinical criteria of the patient, and check the effects of this manipulation on simulated FC. This is then compared with the empirical FC from the patient and compared with healthy control group.

Our results reveal a significant increase of different FC-derived measures along the entire brain, with increasing of the epileptogenic strength of the node, in line with the divergence from the bifurcation point. In addition, this is shown to be enhanced for stronger connected nodes, according to the individual connectome, or for larger epileptogenic zone. These results support the view that perturbations in whole-brain dynamics, due to the epileptogenic activity of certain nodes, cause predictable individualized alterations in the FC [3].

**References**

1. Jirsa VK, Proix T, Perdikis D, Woodman MM, Wang H, Gonzalez-Martinez J, Bernard C, Bénar C, Guye M, Chauvel P, Bartolomei F.: The Virtual Epileptic Patient: Individualized whole-brain models of epilepsy spread. *Neuroimage* 2017, **145(Pt B):**377–388.

2. Wirsich J, Perry A, Ridley B, Proix T, Golos M, Bénar C, Ranjeva JP, Bartolomei F, Breakspear M, Jirsa V, Guye M.: Whole-brain analytic measures of network communication reveal increased structure-function correlation in right temporal lobe epilepsy. *Neuroimage: Clin.* 2016, **11:**707–718.

3. Courtiol J, Petkoski S, Jirsa VK.: in preparation.

## P37 Exact solutions to a Wilson-Cowan network of excitatory and inhibitory neurons whose dynamics is triggered by one single spike

### Roberto J. M. Covolan

#### Department of Neurology, State University of Campinas, Campinas, SP, 13083-887, Brazil

##### **Correspondence:** Roberto J. M. Covolan (covolan@ifi.unicamp.br)

*BMC Neuroscience* 2017, **18(Suppl 1)**:P37

Wilson-Cowan equations [1,2] is a widely used theoretical model by which a network of coupled populations of excitatory and inhibitory neurons is represented. This model is constituted by a set of integro-differential equations that describe the time evolution of the level of activity of excitatory and inhibitory neuronal populations by using a nonlinear sigmoidal function to represent the interactions between these populations. Typical applications require approximation methods and numerical solutions.

In this paper, an analytical method that allow one to obtain exact solutions to a particular setup of Wilson-Cowan equations is presented. This method is based on a spinorial representation and a Feynman-like procedure of ordered exponential operators [3], which was further developed by Fujiwara [4].

The obtained solutions depend on specific initial conditions in the form of delta function, which are interpreted as an action potential-like inputs, thus more general results can be readily generated by applying an impulse train function.

**Conclusion:** Feynman procedure of ordered exponential operators, posteriorly expressed by Fujiwara in terms of expansional operators, has been successfully applied to obtain closed solutions to a particular setup of the Wilson-Cowan equations.

**Acknowledgements**

This work has been supported by FAPESP, Grant Number 2013/07559-3).

**References**

1. Wilson HR, Cowan JD: Excitatory and inhibitory interactions in localized populations of model neurons. *Biophys J* 1972, **12:**1–24

2. Wilson HR, Cowan JD: A mathematical theory of the functional dynamics of nervous tissue. *Kybernetik* 1973, **13:**55–80

3. Feynman RP: An Operator Calculus Having Applications in Quantum Electrodynamics. *Phys. Rev.* 1951, **84**:108.

4. Fujiwara I: Operator Calculus of Quantized Operator. *Prog. Theor. Phys.* 1952, **7:**433

## P38 Encoding variable cortical states with short-term spike patterns

### Bartosz Teleńczuk^1^, Richard Kempter^2^, Gabriel Curio^3^, Alain Destexhe^1^

#### ^1^Unité de Neurosciences, Information et Complexité, CNRS, 91198 Gif-sur-Yvette, France; European Institute for Theoretical Neuroscience, CNRS, Paris, France; ^2^Institute for Theoretical Biology, Humboldt-Universität zu Berlin, Berlin, Germany; ^3^Department of Neurology, Universitätsmedizin Charité, Berlin, Germany

##### **Correspondence:** Bartosz Teleńczuk (telenczuk@unic.cnrs-gif.fr)

*BMC Neuroscience* 2017, **18(Suppl 1)**:P38

Neurons in the primary somatosensory cortex (S1) respond to peripheral stimulation with synchronised bursts of spikes, which lock to the macroscopic 600 Hz EEG wavelets [1,2]. The mechanism of burst generation and synchronisation in S1 is not yet understood. We fitted unit recordings from macaque monkeys with a Poisson-like model including the refractory period (spike-train probability model, STPM). The model combines high-amplitude synaptic inputs with absolute and relative refractoriness. We show that these two properties can reproduce synchronised bursts observed in S1 neurons. The probabilistic nature of the model introduces trial-to-trial response variability. Similar to the experimental data, the variability can be decomposed into stereotypical spike patterns consisting of short bursts of spikes with variable number of spikes and length of within-burst intervals. Next, we extend the model to a population of uncoupled neurons, which receive common inputs fluctuating in amplitude across trials. We demonstrate that these fluctuations introduce correlations between neurons and between the single-neuron spike patterns and population activity (high-frequency EEG wavelets) as observed experimentally [2].

To further study the biophysical mechanism behind S1 burst responses, we develop a single-compartment model (leaky integrate-and-fire, LIF) receiving intracortical and feedforward thalamic inputs. The intracortical inputs are assumed to be in a balanced state, where excitatory and inhibitory currents nearly cancel each other out yielding the neuron in the high-conductance state [3]. This enables the model neuron to respond quickly to a transient barrage of thalamocortical inputs and generate bursts of spikes tightly locked to the stimulus onset. The transient response decays quickly to the baseline and terminates the burst due to the activity-dependent depression of thalamocortical synapses. This model can reproduce many features of experimental data, in particular the burst statistics and the presence of spike patterns.

Our findings show that a simple feedforward processing of peripheral inputs could give rise to neuronal responses with non-trivial temporal and population statistics. We conclude that neural systems could use refractoriness to encode variable cortical states into stereotypical short-term spike patterns amenable to processing at neuronal time scales (tens of milliseconds). See [4] for more details.

**Acknowledgements**

This study was partially funded by the CNRS, European Commission (Human Brain Project, H2020-720270) and BMBF (grants BCCN-B1, 01GQ1001A and 01GQ0972).

**References**

1. Baker S, Curio G, Lemon R. EEG oscillations at 600 Hz are macroscopic markers for cortical spike bursts. J. *Physiol.* 2003; **550:**529–534.

2. Telenczuk B, Baker SN, Herz AVM, Curio G. High-frequency EEG covaries with spike burst patterns detected in cortical neurons. *J. Neurophysiol.* 2011; **105:**2951–9.

3. Destexhe A, Rudolph M, Paré D. The high-conductance state of neocortical neurons in vivo. Nat. Rev. *Neurosci.* 2003; **4:**739–51.

4. Telenczuk B, Kempter R, Curio G, Destexhe A. Encoding variable cortical states with short-term spike patterns. 2017; preprint http://biorxiv.org/content/early/2017/01/04/098210 bioRxiv doi:10.1101/098210

## P39 Cat Paw-shaking as a Transient Response to Sensory Input to Locomotion CPG

### Jessica Parker^1^, Alexander N. Klishko^2^, Boris I. Prilutsky^2^, Gennady Cymbalyuk^1^

#### ^1^Neuroscience Institute, Georgia State University, Atlanta, GA 30303, USA; ^2^School of Biological Sciences, Georgia Institute of Technology, Atlanta, GA 30332, USA

##### **Correspondence:** Jessica Parker (jgreen59@student.gsu.edu)

*BMC Neuroscience* 2017, **18(Suppl 1)**:P39

It has not yet been determined whether the same CPG can generate rhythmic activity of distinct behaviors with significantly different frequencies (1 vs 10 Hz), such as locomotion and paw-shake responses. We have previously published a model of a multistable CPG constructed as a half-center oscillator (HCO), which consists of two reciprocally inhibitory interneurons [1]. This HCO was able to produce the stable rhythms associated with both locomotion and paw-shake. We also used this HCO model to demonstrate that a multifunctional CPG controlling a neuromechanical model of a cat hind limb could reproduce the essential features of the rhythm, kinematics and muscle synergies of cat locomotion and paw-shake responses [1]. Here, we show that using a pulse of current, a transient paw-shake-like rhythm can be elicited either in the multistable HCO model or in a monostable version of the model. Our model predicts that the flexor burst duration and the extensor interburst interval will increase throughout a single paw-shake-like response. We tested these predictions by eliciting paw-shake responses in cats by attaching a piece of adhesive tape to the hind paw and allowing the cat to walk on a level walkway. Hind limb kinematics and EMG activity of various hind limb muscles were recorded [3]. The cats performed paw-shake responses intermittently while walking, and each paw-shake response consisted of 4 to 10 cycles. In accordance with previous studies [2], we found a progressive increase in EMG burst period throughout consecutive cycles of paw-shake responses. Furthermore, we found a progressive increase in EMG burst duration in consecutive paw-shake cycles for flexors and a progressive increase in EMG interburst interval for extensors. We conclude that a paw-shake response might be a transient response to sensory input to the locomotion CPG.

**Acknowledgements**

We acknowledge support by the NSF PHY-0750456 to Gennady Cymbalyuk and by NIH P01 HD32571, R01 EB012855, and R01 NS048844 and by the Center for Human Movement Studies at GA Tech to Boris I. Prilutsky.

**References**

1. Bondy B, Klishko AN, Edwards DH, Prilutsky BI, Cymbalyuk G: Control of cat walking and paw-shake by a multifunctional central pattern generator. In: *Neuromechanical Modeling of Posture and Locomotion.* edn. New York: Springer; 2016: 333–359.

2. Koshland GF, Smith JL: Mutable and immutable features of paw-shake responses after hindlimb deafferentation in the cat. *J Neurophysiol* 1989, **62**(1):162–173.

3. Hodson-Tole EF, Pantall AL, Maas H, Farrell BJ, Gregor RJ, Prilutsky BI: Task dependent activity of motor unit populations in feline ankle extensor muscles. *J Exp Biol* 2012, **215**:3711–3722.

## P40 Population Coding with Two-Dimensional Feature Maps in the Retina

### Felix Franke^1^, Andreas Hierlemann^1^, Rava Azeredo da Silveira^2,3^

#### ^1^Department of Biosystems Science and Engineering, ETH Zürich, Basel, Switzerland; ^2^Ecole Normale Supérieure, Paris, France; ^3^Centre National de la Recherche Scientifique, Paris, France

##### **Correspondence:** Felix Franke (felix.franke@bsse.ethz.ch)

*BMC Neuroscience* 2017, **18(Suppl 1)**:P40

A robust internal representation of relevant variables in the external world is necessary to guide animal behavior. The brain constructs this internal representation from sensory inputs. The nervous system has to rely on a two-dimensional sensor array for visual perception, the retina. Sensor arrays encode external variables with two-dimensional feature maps: the concerted activity of photoreceptors encodes information about brightness and color across the entire retina. These brightness levels also encode all information about more complex features of the visual stimulus, such as the location, velocity, and contrast of moving objects. But to access the more complex features the nervous system first needs to extract them from the photoreceptor activity. Retinal circuitry processes the photoreceptor activity and sends the processed information via the spiking activity of retinal ganglion cells to the brain. There are over 30 different types of retinal ganglion cells, each cell type tiling the entire retina with their receptive fields, and each cell type sending information about different features to the brain. The entire information the brain receives about the visual world is, thus, encoded in the concerted activity of those >30 cell types, each representing a two-dimensional map with its particular sensitivity, e.g., sensitivity the direction of local movement or to the presence of an edge.

Here, we analyze the encoding properties of direction-selective retinal ganglion cells for position, velocity, and direction of moving objects. To this end, we use tuning functions estimated from real recordings of mouse retinae and Fisher Information to calculate the precision of the neural code. We estimate the coding precision for different visual features (position, velocity, direction), and for a variety of stimuli. We then proceed as follows: First, we vary single-cell properties, i.e., the tuning functions, and quantify the impact of these changes on the coding precision. Second, we change population properties, e.g., the geometric arrangement of the receptive fields within the mosaic, and, again, quantify the consequences.

When varying single-cell properties, we concentrate on the feature-selectivity of the cells, i.e., direction-selectivity. In particular, we compare the encoding precision on the direction and position of a moving object in a mosaic of direction-selective cells vs. in a mosaic of non-direction-selective cells (with matched mean firing rates).

We find that non-direction-selective cells encode direction (and position) more faithfully than direction-selective cells if the moving object is larger than the receptive-field size and the distance of receptive field centers within the mosaic. At first, this is counter-intuitive, as feature selectivity for a given feature should increase the encoding precision for that feature. However, a feature can be encoded by both, feature-selective and non-selective cells, the question is with what ease it can be decoded. This yields a hypothesis for the function of direction-selective cells: either they are specialized to encode direction locally within their receptive fields or they satisfy a requirement to decode direction early in the visual pathway, presumably to allow for the decoding of more sophisticated visual features immediately downstream.

## P41 A detailed computational reconstruction of the cerebellum granular layer network predicts large scale spatiotemporal dynamics of neuronal activity

### Stefano Casali^1^, Stefano Masoli^1^, Martina Rizza^1,3^, Egidio D’Angelo^1,2^

#### ^1^Department of Brain and Behavioral Sciences, University of Pavia, Pavia, Italy 27100; ^2^Brain Connectivity Center, C. Mondino National Neurological Institute, Pavia, Italy 27100; ^3^Dipartimento di Informatica, Sistemistica e Comunicazione, Università degli Studi di Milano-Bicocca, Viale Sarca, Italy

##### **Correspondence:** Stefano Masoli (stefano.masoli@unipv.it)

*BMC Neuroscience* 2017, **18(Suppl 1)**:P41

The main aim of the present work was to demonstrate that high-level spatiotemporal dynamics taking place in the cerebellar granular layer can be understood as emergent phenomena, naturally determined by the complex interaction occurring among microscopic variables, like specific topology of intercellular connectivity and neurophysiological properties of single neurons. To this aim, we have developed an updated large-scale computational model of the cerebellar granular layer in Python-Neuron [1]. Our results show that (1) the center-surround profile describing the ratio between excitation and inhibition observed in slices of cerebellar tissue depends on spatial arrangement of Granule cells (*Grcs*) – Golgi cells (*Gocs*) connectivity; (2) spatial interaction between different spots of activation spontaneously leads to combinatorial responses, like combined excitation and inhibition; (3) the entire granular layer generates coherent oscillations in response to random background input when two conditions are met: the mossy fibers (*mfs*) input conveyed to *Gocs* and mutual inhibition among *Gocs* are weak or absent; (4) Spatial distribution of long term potentiation (LTP) and inhibition (LTD) at the *mfs*-*Grcs* synapses can be faithfully reproduced in our network model. The presented results are in strict agreement with observations from network-level in vitro experiments, like VSD and MEA recordings [2, 3], and from in vivo LFP studies [4]. The model has also been validated against highly precise experiments conducted in cerebellar slices using Two-Photon imaging microscopy [5], which allowed to test its precision to the level of single-spike activity.

**References**

1. M. L. Hines, A. P. Davison, and E. Muller. NEURON and Python. Front Neuroinform, 3, 2009.

2. Mapelli J, D’Angelo E: The spatial organization of long-term synaptic plasticity at the input stage of Cerebellum. *J Neurosci* 2007**, 27:** 1285–1296

3. Mapelli J, Gandolfi D, D’Angelo E: Combinatorial responses controlled by synaptic inhibition in the Cerebellum granular layer. *J Neurophysiol* 2010, **103:** 250–261

4. Diwakar S, Lombardo P, Solinas S, Naldi G, D’Angelo E: Local field potential modeling predicts dense activation in cerebellar granule cells clusters under LTP and LTD control. *PLoS One* 2011, **6(7)**

5. Gandolfi D, Pozzi P, Tognolina M, Chirico G, Mapelli J, D’Angelo E: The spatiotemporal organization of cerebellar network activity resolved by two-photon imaging of multiple single neurons. *Front Cell Neurosci* 2014, **8:**92 doi: 10.3389/fncel.2014.00092

## P42 A Biophysically Detailed Cerebellar Stellate Neuron Model Predicts Local Synaptic Interactions

### Martina Francesca Rizza^1,2^, Stefano Masoli^1^, Egidio D’Angelo^1,3^

#### ^1^Department of Brain and Behavioral Sciences, University of Pavia, Via Forlanini 6, I-27100, Pavia, Italy; ^2^Dipartimento di Informatica, Sistemistica e Comunicazione, Università degli Studi di Milano-Bicocca, Viale Sarca 336, I-20100, Milan, Italy; ^3^Brain Connectivity Center, Istituto Neurologico IRCCS C. Mondino, Via Mondino 2, Pavia, I-27100, Italy

##### **Correspondence:** Martina Francesca Rizza (martina.rizza@disco.unimib.it)

*BMC Neuroscience* 2017, **18(Suppl 1)**:P42

The cerebellar stellate cells (SC) are located in the molecular layer and play a critical role in modulating the activity of Purkinje cells (PC). Starting from a broad range of published experimental observations, we constructed a biophysically realistic SC model in Python-NEURON [1]. A human SC morphology (Neuromorpho.org) was comprised of highly branched dendritic tree, soma, axon initial segment (AIS) and axon with collaterals [2]. The membrane mechanism [3] were distributed according to the literature. Two distinct types of Na^2+^ channels were used: Nav1.1 (without resurgent current) in the soma and Nav1.6 (with resurgent current) in the AIS/axon. The K^+^ channels were Kv3.4 and Kv4.3, mainly in the soma. The Ca^2+^ and Ca^2+^-dependent K^+^ channels were KCa1.1 and KCa2.2, mainly in the dendrites. The model was endowed with an intracellular Ca^2+^ buffer that contributed to spike repolarization and firing pattern regulation. In the SC model, the set of maximum ionic conductances (G_i-max_) had to be tuned to match the firing pattern revealed by electrophysiological recordings. G_i-max_ tuning was performed by automatic parameter estimation using both the swarm intelligence algorithm (particle swarm optimization, PSO [4]) and the genetic algorithms MOEA [5] (in BluePyOpt [6]). The optimized models showed spontaneous firing with an average frequency of 14 Hz, appropriate spike shape and amplitude. The SC model was validated by running simulation demonstrating the impact of the gap junctions [7] in conjunction with glutamatergic synaptic inputs from parallel fibers (pf) and the GABAergic synapses between SCs. In addition, we evaluated the impact of SC activity on PCs. This model thus provides a valuable tool to further investigate the SC function in cerebellar network models.

**References**

1. Hines ML, Davison AP, Muller E: NEURON and Python. *Front Neuroinform* 2009, 3.

2. Jacobs B, Johnson NL, Wahl D, Schall M, Maseko BC, Lewandowski A, Manger PR: Comparative neuronal morphology of the cerebellar cortex in afrotherians, carnivores, cetartiodactyls, and primates. *Frontiers in Neuroanatomy* 2014, 8.

3. Masoli S, Solinas S, D’Angelo E: Action potential processing in a detailed Purkinje cell model reveals a critical role for axonal compartmentalization. *Front. Cell. Neurosci.* 2015; **9:**1–22.

4. Kennedy J, Eberhart R: Particle Swarm Optimization. *In Proc IEEE Int Conf Neural Networks* 1995, volume 4, pages 1942–1948.

5. Druckmann S: A novel multiple objective optimization framework for constraining conductance-based neuron models by experimental data. *Frontiers in Neuroscience* 2007, **1(1):** 7–18.

6. Van Geit W, Gevaert M, Chindemi G, Rössert C, Courcol J-D, Muller EB, Schürmann F, Segev I, Markram H: BluePyOpt: Leveraging Open Source Software and Cloud Infrastructure to Optimise Model Parameters in Neuroscience. *Front Neuroinform* 2016, **10:**1–30.

7. Alcami P, Marty A: Estimating functional connectivity in an electrically coupled interneuron network. *Proceedings of the National Academy of Sciences* 2013, **110(49):** E4798–E4807.

## P43 Neuromodulation of Subgenual Cingulate Activity Localizable from EEG

### Yinming Sun^1,2^, Willy Wong^1,3^, Faranak Farzan^2^, Daniel M. Blumberger^2,4^, Zafiris J. Daskalakis^2,4^

#### ^1^Institute of Biomaterials and Biomedical Engineering, University of Toronto, Toronto, ON, M5S3G9, Canada; ^2^Centre for Addiction and Mental Health, Toronto, ON, M5T1R8, Canada; ^3^Department of Electrical and Computer Engineering, University of Toronto, Toronto, ON, M5S3G4, Canada; ^4^Department of Psychiatry, University of Toronto, Toronto, ON, M5S3G4, Canada

##### **Correspondence:** Yinming Sun (yinming.sun@gmail.com)

*BMC Neuroscience* 2017, **18(Suppl 1)**:P43

Subgenual cingulate (SGC) activity is implicated in the pathophysiology of major depressive disorder (MDD) [1]. Neuromodulation treatments for MDD may work by modifying connections between the SGC and other brain regions. One prominent connection is between the SGC and dorsolateral prefrontal cortex (DLPFC). The present work explores SGC source activity in two studies: 1) Verifying source localization with EEG recorded from patients receiving deep brain stimulation (DBS) in the SGC, 2) Using source analysis to determine if magnetic seizure therapy (MST) for MDD works by affecting the connection between the DLPFC and SGC. In the first study, accuracy of source localization was quantified by the error in locating the source of DBS stimulus, which was extracted in sensor space from EEG recorded during active stimulation with matched filters based on previously published methods [2]. Since the magnitude of the DBS stimulus is much larger than typical brain activity, a threshold for detection was determined by examining source localization results from simulated data created by adding suppressed versions of the extracted DBS stimulus to data with the stimulator turned off. Results from this study is one of the first to empirically demonstrate the efficacy of EEG in detecting activity from a deep brain source. In the second study, source analysis was applied to EEG recorded during transcranial magnetic stimulation (TMS-EEG) before and after a course of MST treatment. MST uses a train of magnetic pulses delivered over the scalp to create a seizure and has shown efficacy for reducing suicidal ideations [3]. For TMS-EEG, magnetic pulses were delivered to the dorsolateral prefrontal cortex (DLPFC) and the EEG was collected with a 64-channel system. A TMS-EEG measure called the significant current scatter (SCS) [4] was calculated based on the computed source image. SCS quantifies the spread of activation from a stimulus location to other brain regions and has been effective in capturing changes in brain network connections during task performance and loss of consciousness. A standard atlas was used to identify dipoles belonging to the SGC region. Results show that SCS values were significantly decreased after MST based on the Wilcoxon signed-rank test (Z = −2.16, p = 0.03). For patients with baseline suicidal ideation, higher baseline SCS values were correlated with greater SSI reductions (Spearman Rho = 0.625, p = 0.004). Using baseline SCS values, suicidal ideation remission can also be predicted with 100% sensitivity and 70% specificity (AUC = 0.86, p = 0.01). Overall, this work provides both a methodological confirmation for the utility of EEG for studying SGC activity and a mechanistic explanation for MST’s therapeutic benefit for MDD patients. MST may exert its therapeutic effects on suicidal ideation via transsynaptic modulation of the SGC from the prefrontal cortex and reconfiguring pathological connections in the process of treatment. With carefully designed experiments, future EEG studies with source analysis will undoubtedly yield additional mechanistic insights for neuroscience.

**Acknowledgements**

This work was supported by the Canadian Institutes of Health Research (CIHR), the Brain and Behaviour Research Foundation (formerly NARSAD) and the Temerty Family and Grant Family and through the Centre for Addiction and Mental Health (CAMH) Foundation and the Campbell Institute.

**References**

1. Mayberg HS: Limbic-cortical dysregulation: a proposed model of depression. *J Neuropsychiatry Clin Neurosci* 1997, **9**(3):471–481.

2. Sun Y, Farzan F, Garcia Dominguez L, Barr MS, Giacobbe P, Lozano AM, Wong W, Daskalakis ZJ: A novel method for removal of deep brain stimulation artifact from electroencephalography. *J Neurosci Methods* 2014, **237C**:33–40.

3. Sun Y, Farzan F, Mulsant BH, Rajji TK, Fitzgerald PB, Barr MS, Downar J, Wong W, Blumberger DM, Daskalakis ZJ: Indicators for Remission of Suicidal Ideation Following Magnetic Seizure Therapy in Patients With Treatment-Resistant Depression. *JAMA Psychiatry* 2016.

4. Casali AG, Casarotto S, Rosanova M, Mariotti M, Massimini M: General indices to characterize the electrical response of the cerebral cortex to TMS. *Neuroimage* 2010, **49**(2):1459–1468.

## P44 Phase dynamics in a GO/NOGO finger tapping task

### Svitlana Popovych^1,2^, Shivakumar Viswanathan^2,3^, Nils Rosjat^1,2^, Christian Grefkes^2,3^, Gereon R. Fink^2,3^, Silvia Daun^1,2^

#### ^1^Heisenberg Research Group of Computational Neuroscience - Modeling Neural Network Function, Department of Animal Physiology, Institute of Zoology, University of Cologne, Cologne, 50674, Germany; ^2^Cognitive Neuroscience, Institute of Neuroscience and Medicine (INM-3), Research Center Juelich, Juelich, 52425, Germany; ^3^Department of Neurology, University Clinic Cologne, 50937, Cologne, Germany

**Correspondence:** Silvia Daun (Silvia.Daun@uni-koeln.de)

*BMC Neuroscience* 2017, **18(Suppl 1)**:P44

Motor actions arise as a result from a complex interplay between various brain regions. Since the same brain regions can form different functional networks depending on the action, it is, in general, a demanding task to identify the neural signals that are the constituting components of a motor action.

In a previous study, we found that voluntary and visually triggered movements exhibit significant phase locking in the delta-theta frequency band (2–7 Hz) starting already before movement onset in the motor regions contralateral to the moving hand both in younger [1] and older subjects [2]. This phase locking therefore seems to be an electrophysiological marker of movement execution, no matter how the movement has been initiated. We suggested that this synchrony helps the simultaneously active pathways of distinct cortical networks that initiate voluntary and stimulus-triggered movements, converge to a common motor output and activate the appropriate muscles to perform the movement.

In these previous studies, since a prepared movement was always executed, it is unclear whether the reported pre-movement phase locking in the low frequency bands is a necessary prerequisite for movement execution, or rather a correlate of movement preparation. To distinguish between these alternatives, we recorded EEG from young (18–35 years) right-handed healthy subjects as they performed a simple GO/NOGO finger tapping task where a prepared action was either executed (GO trials, 75%) or cancelled (NOGO, 25%).

Results of the data analysis revealed an increase in phase synchronization in the low frequency bands in both the GO and NOGO condition, hinting at a potential role of delta-theta phase synchronization in movement preparation.

**Acknowledgements**

This work was supported by the DFG grants to S. Daun (GR3690/2-1, GR3690/4-1 and UoC EG CONNECT).

**References**

1. Popovych S, et al.: Phase-locking in the delta-theta band is an EEG marker of movement execution. *Neuroimage* 2016, **139**:439–449.

2. Liu L et al.: Movement-related intra-regional phase locking in the delta-theta frequency band in young and elderly subjects. *Society for Neuroscience* 2016, Annual meeting.

## P45 Mechanisms of focal seizure generation in a realistic small-network model with ionic dynamics

### Damiano Gentiletti^1^, Piotr Suffczynski^1^, Vadym Gnatkovski^2^, Marco De Curtis^2^

#### ^1^Department of Experimental Physics, University of Warsaw, Warsaw, 02-093, Poland; ^2^Istituto Neurologico Carlo Besta, Milan, 20133, Italy

##### **Correspondence:** Damiano Gentiletti (Damiano.Gentiletti@fuw.edu.pl)

*BMC Neuroscience* 2017, **18(Suppl 1)**:P45

Epilepsy and seizures are traditionally associated with an imbalance between excitatory and inhibitory forces in the brain. This classic view is challenged by the in vitro isolated guinea pig brain model of focal seizures [1]. Based on experimental data recorded from the entorhinal cortex (EC), it appears that inhibitory neurons are active at the very beginning of a focal seizure, whereas excitatory cells are quiescent. This is accompanied by an increase of the extracellular potassium concentration. Within a few seconds from seizure onset, the principal cells display excessive firing associated with the seizure discharge. Neuronal firing of principal neurons subsequently decreases, and further evolves into rhythmic bursting activity that terminates the seizure.

In order to gain more understanding of the link between ionic dynamics and neuronal activity during seizures we developed a computational model of the entorhinal cortex circuit. The model consists of a small neuronal network made up of five hippocampal cells – an inhibitory interneuron and four pyramidal cells – each one surrounded by an extracellular space. Each extracellular environment incorporates realistic dynamics of Na^+^, K^+^, Cl^−^ and Ca^2+^ ions, the glial buffering system and diffusion mechanisms. Different extracellular spaces communicate with each other by diffusive exchange of K^+^ ions.

Simulations performed with our in silico model show that ion concentration changes have significant impact on the network behaviour and determine the different phases of a focal seizure. In particular, the model is able to reproduce the membrane potential and potassium concentration traces recorded experimentally, and the pathological sequence taking place in the pyramidal cells: quiescent period – seizure onset – excessive pyramidal firing – late bursting phase. Our simulations confirm the experimentally driven hypothesis that strong discharge of inhibitory interneurons may result in long lasting accumulation of extracellular K^+^, which in turn is responsible for seizure progression in principal cells.

Our study also shows that a reduced model with fixed ionic concentrations is not able to reproduce the seizure patterns observed experimentally, pointing to the importance of the role played by non-synaptic mechanisms in modeling focal epileptic activity.

Additionally, we exploited the model to suggest and test novel antiepileptic therapies. A potentially viable strategy reckons on the implementation of a nanoparticle system designed to buffer the excess of extracellular potassium ions. Simulations incorporating such an additional mechanism show feasibility of seizure control by artificial pharmacological agents, suggesting future avenues of controlling ictogenesis.

**References**

1. De Curtis M, Gnatkovsky V: Reevaluating the mechanisms of focal ictogenesis: the role of low-voltage fast activity. *Epilepsia* 2009, **50(12):**2514–2525.

## P46 Pre-allocation of working memory modulates memory performance

### Hyeonsu Lee^1^, Woochul Choi^1,2^, Se-Bum Paik^1,2^

#### ^1^Department of Bio and Brain Engineering, Korea Advanced Institute of Science and Technology, Daejeon 34141, Republic of Korea; ^2^Program of Brain and Cognitive Engineering, Korea Advanced Institute of Science and Technology, Daejeon 34141, Republic of Korea

##### **Correspondence:** Hyeonsu Lee (hslee9305@kaist.ac.kr)

*BMC Neuroscience* 2017, **18(Suppl 1)**:P46

Working memory capacity is known to be limited to a small number of items. Two controversial memory models–the slot model and the resource model–were proposed to describe limited capacity. While the slot model hypothesizes that a fixed number of discrete slots store the information for each item, the resource model proposes that continuous working memory resources can be allocated to each item and that memory precision will increase as more resources are allocated to the item [1]. It has recently been shown that the resource model appears to successfully describe the observation that memory precision for each item smoothly decreases as the total number of items increases [2]. However, it is still elusive how the resources are distributed and whether allocating resources before encoding–pre-allocation– actually affects memory performance. In this study, we suggest that memory pre-allocation is in effect and modulates memory performance. To examine the pre-allocation effect of working memory on memory performance, we performed a human psychophysics experiment in which subjects memorized the pattern of visual stimuli presented sequentially. To study the pre-allocation effect, the total number of items was either informed to the subject before the items were presented or not informed in the control case. In the pre-allocated condition, there were two schemes: whether the number given as a cue is the same as the actual number of items appeared (matched cue) or is less than the number of items (non-matched cue). The results showed that the performance for the pre-allocated case was higher than that for the control case where the number of items was not informed. This suggests that working memory resources may be pre-allocated based on cue and that allocated resources improve memory performance. In addition, in the non-matched cue case, memory precision was lower than the matched cue case. Whereas working memory resources were pre-allocated by the cue in both cases, an unexpected additional item was given to subject in the non-matched cue case. Thus, working memory resources were insufficient to store the information of that item. Our results imply that pre-allocation may allocate working memory resources efficiently. We propose that working memory resources can be pre-allocated prior to encoding the items, and allocating resources may modulate memory performance.

**References**

1. Ma WJ, Husain M, Bays PM, Ji Ma W, Husain M, Bays PM: Changing concepts of working memory. *Nat. Neurosci* 2014, **17**:347–356.

2. Gorgoraptis N, Catalao RFG, Bays PM, Husain M: Dynamic updating of working memory resources for visual objects. *J. Neurosci* 2011, **31**:8502–8511.

## P47 Temporal dynamics of bistable perception reveals individual time window for perceptual decision making

### Woochul Choi^1,2^, Se-Bum Paik^1,2^

#### ^1^Program of Brain and Cognitive Engineering, Korea Advanced Institute of Science and Technology, Daejeon 34141, Republic of Korea; ^2^Department of Bio and Brain Engineering, Korea Advanced Institute of Science and Technology, Daejeon 34141, Republic of Korea

##### **Correspondence:** Woochul Choi (choiwc1128@kaist.ac.kr)

*BMC Neuroscience* 2017, **18(Suppl 1)**:P47

When a sensory stimulus can be interpreted in two alternative ways, the perception of the stimulus often changes spontaneously and quasi-periodically between the two. This phenomenon, called bistable perception, may provide rich information about how the brain dynamically interprets the sensory stimulus. One of the most interesting characteristics of bistable perception is that the switching frequencies are fairly consistent within an individual but vary across individuals. However, it is still unclear what drives the periodic alternation and which parameter determines the individual switching frequency. To explain the origin of diverse perceptual alternation, we assumed that bistable perception results from the integration of sensory information, and that each individual has a specific time window for sensory information integration that might determine their own switching frequency.

To examine the hypothesis, we used randomly moving dots in an annulus (“racetrack” stimuli) [1]. During the stimulation, we controlled the portion of the rotating dots with a coherence parameter, c. When c = 0, all the dots would move in random directions, inducing illusory motion, and the participant would experience bistable perception. In contrast, when c > 0, the racetrack would generate noisy rotational motion, and the participant’s response would follow the actual motion (Figure 1B). To find the relationship between the switching frequency of bistable perception and the response dynamics during motion detection tasks, we examined individual phase duration, τ, of bistable perception and response time, and the accuracy in detection of ambiguous motion. Our result showed that the response time of motion detection was positively correlated with the τ of bistable perception (N = 49, R = 0.52, p < 0.001), while the accuracy was independent of τ (Figure 1C). Next, to investigate whether the time window for information integration is a crucial factor in determining the τ of bistable perception, we modified the racetrack to have time-varying motion with different frequencies. Our result shows that individuals with short τ has smaller integration time than individuals with long τ (Figure 1D). In addition, the simulation study shows that diverse time windows of stimulus integration can regenerate various τ of bistable perception. This result supports the idea that each individual has an intrinsic time window for information accumulation, and that the duration of the window may determine the τ of bistable perception.
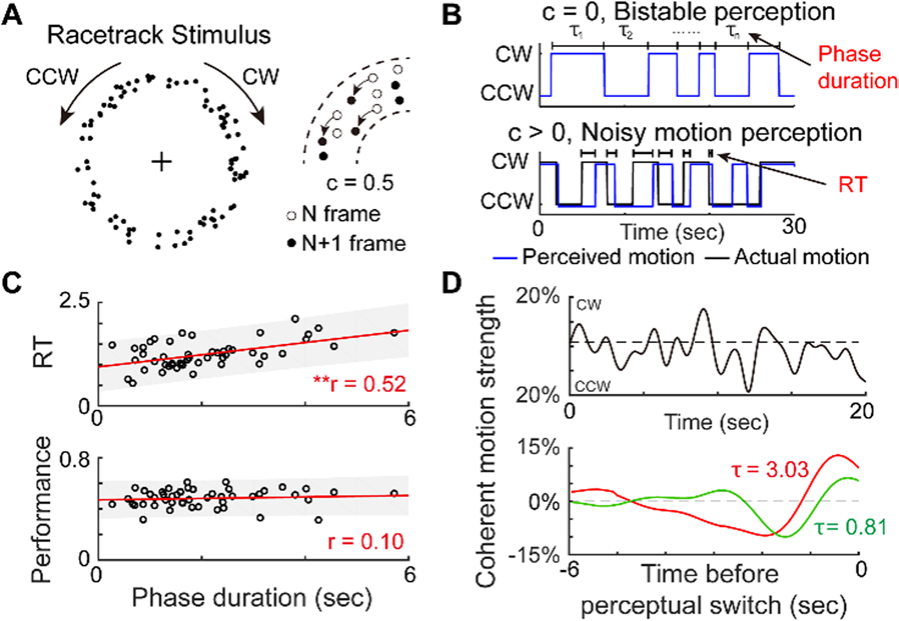


**Figure 1.** Temporal dynamics of bistable perception and behaviour characteristic of perceptual decision making **A.** Racetrack stimulus. Coherence level, c determines the portion of rotating dots **B.** In c = 0, perception is illusory bistable motion (top) and in c > 0, perception follows actual motion (bottom) **C.** Correlation between subjects’ phase duration and the response time (top) and motion detection accuracy (bottom). **D.** Time-varying coherence stimulus (top), and the reverse correlation analysis of perceptual switching (bottom). Individual with long τ has larger information integration time than individual with short τ

**Conclusions:** A series of psychophysics experiments shows that the phase duration of bistable perception is positively correlated with the sensory information accumulation time. This suggests that bistable perception may result from continuous decision making related to the accumulation of sensory information.

**Reference**

1. Jain S: Performance characterization of Watson Ahumada motion detector using random dot rotary motion stimuli. *PLoS One* 2009, **4**. e4536

## P48 Regularly structured retinal mosaics can induce structural correlation between orientation and spatial frequency maps in V1

### Jaeson Jang^1^, Se-Bum Paik^1,2^

#### ^1^Department of Bio and Brain Engineering, Korea Advanced Institute of Science and Technology, Daejeon 34141, Republic of Korea; ^2^Program of Brain and Cognitive Engineering, Korea Advanced Institute of Science and Technology, Daejeon 34141, Republic of Korea

##### **Correspondence:** Jaeson Jang (jaesonjang@kaist.ac.kr)

*BMC Neuroscience* 2017, **18(Suppl 1)**:P48

In higher mammals, the primary visual cortex (V1) is organized into functional maps that capture specific features of visual stimulus such as orientation or spatial frequency. In each functional map, the preferred features change continuously in a quasi-periodic manner. Moreover, it has been reported that the topographies of the functional maps on the same cortical surface are correlated. For example, the contour of an iso-orientation domain orthogonally intersect the contour of an iso-frequency domain [1]. This implies the systematic organization of the functional maps but leaves ambiguous how such correlated topography could be developed in V1. In this study, using computer simulation, we show that orientation and frequency maps are both seeded from the regularly structured retinal mosaics and that this common source can induce the observed correlated organization of functional maps. Previously, it was proposed for a theoretical model that the superposition between the hexagonal mosaics of ON and OFF retinal ganglion cells (RGC) generates a moiré interference pattern (Figure 1A) [2]. The key assumption of the model was that the orientation preference of a V1 neuron can be predicted by the relative location of ON and OFF RGCs, which is supported by recent observations that the structure of cortical functional maps is strongly correlated with the local organization of ON and OFF afferents [3, 4]. Thus, the model suggested that the repetition of similar orientation preference across the interference pattern can seed a quasi-periodic orientation map (Figure 1B). Here, we propose that the frequency preference of a cortical neuron depends on the distance between local ON and OFF RGCs, which is also repeated across the interference pattern. Our simulation reproduced the quasi-periodic orientation map and the frequency map both seeded from a common set of hexagonal ON and OFF RGC mosaics (Figure 1B,C). We found that the preferred orientation and frequency change in relation to each other in the orthogonal direction [1], because the distance between ON and OFF RGCs changes in the direction orthogonal to the change of orientation preference across the moiré interference. Our simulation also reconstructed the observed relationship in which a pinwheel in the orientation map overlaps high or low frequency domains in the frequency map [5]. Additionally, we found hexagonal structure in the observed frequency map, as our model predicted. Our results explain how the topographic correlation between cortical functional maps is developed from the identical sources of retinal mosaics. This may provide a blueprint explaining how the visual system develops the correlated structure of functional maps with a simple organization principle in retina.
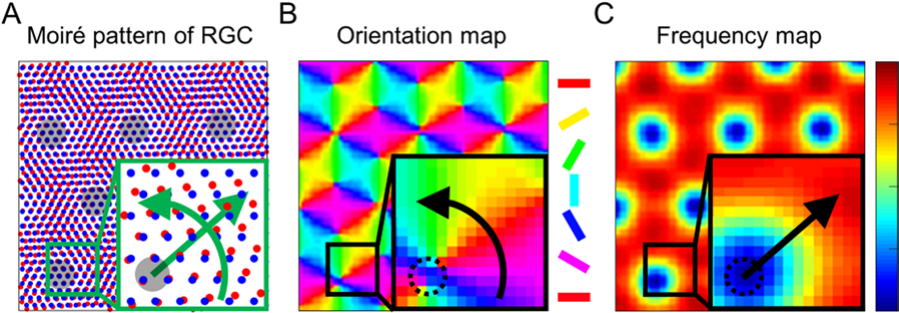


**Figure 1.** Regularly structured RGC mosaics can seed topographic correlation between cortical functional maps. **A.** Moiré interference pattern of ON- and OFF-center RGCs **B.** Simulated orientation map **C.** Simulated spatial frequency map; two features change in the orthogonal direction

**References**

1. Nauhaus I, Nielsen KJ, Disney A a, Callaway EM: Orthogonal micro-organization of orientation and spatial frequency in primate primary visual cortex. *Nat Neurosci* 2012, **15**:1683–1690.

2. Paik S-B, Ringach DL: Retinal origin of orientation maps in visual cortex. *Nat Neurosci* 2011, **14**:919–925.

3. Kremkow J, Jin J, Wang Y, Alonso JM: Principles underlying sensory map topography in primary visual cortex. *Nature* 2016, **533**:52–57.

4. Lee K-S, Huang X, Fitzpatrick D: Topology of ON and OFF inputs in visual cortex enables an invariant columnar architecture. *Nature* 2016, **533**:90–94.

5. Hübener M, Shoham D, Grinvald A, Bonhoeffer T: Spatial relationships among three columnar systems in cat area 17. *J Neurosci* 1997, **17**:9270–9284.

## P49 Distinct role of synaptic and nonsynaptic plasticity in memory ensemble formation, allocation, and linkage

### Youngjin Park^1^, Se-Bum Paik^1,2^

#### ^1^Department of Bio and Brain Engineering, Korea Advanced Institute of Science and Technology, Daejeon 34141, Republic of Korea; ^2^Program of Brain and Cognitive Engineering, Korea Advanced Institute of Science and Technology, Daejeon 34141, Republic of Korea

##### **Correspondence:** Youngjin Park (yodamaster@kaist.ac.kr)

*BMC Neuroscience* 2017, **18(Suppl 1)**:P49

Synaptic plasticity—the change of synaptic strength between pre- and postsynaptic neurons—is widely believed to be the basis of learning and memory. Yet, there is another type of plasticity observed in the brain: nonsynaptic plasticity [1]. Nonsynaptic plasticity, often referred to as intrinsic plasticity, induces changes in excitability of a neuron by modulating neuronal intrinsic properties. A growing number of studies report that changes in nonsynaptic properties, such as the action potential threshold or afterhyperpolarization level, are triggered by learning [1]. This indicates nonsynaptic plasticity may play crucial roles in memory formation, but the functional mechanisms still remain elusive. Here we hypothesize distinct roles for synaptic and nonsynaptic plasticity in learning and memory: activity-dependent synaptic plasticity is involved in memory ensemble formation, and nonsynaptic plasticity is involved in pre-allocation and linkage of memory. To validate our ideas, we constructed a spiking neural network model consisting of 100 excitatory and 30 inhibitory leaky integrate-and-fire neurons (Figure 1A). Output layer neurons were set to receive temporal patterns from input layer neurons, and receive lateral inhibition from nearby inhibitory neurons. As the synaptic learning rule, spike-timing-dependent plasticity was applied to sparse feedforward connection between input and output layers. Our simulation results show that the network model learned temporal patterns by repeated exposure and formed a neuronal ensemble, a set of output neurons that selectively responded to a trained pattern (Figure 1B). Remarkably, by applying nonsynaptic plasticity to the network, we could control the pre-allocation of memory. Neurons with higher excitability had a greater chance of being recruited into a memory ensemble than those in a control group, as reported from the experiment (Figure 1C) [2]. Moreover, the total size of the memory ensemble remained consistent throughout the simulation, due to the inhibitory feedback. We did further simulations to investigate the advantage of nonsynaptic modulation. First, it regulates the learning rate of neurons; we observed that neurons with greater excitability learned faster than normal neurons. Second, temporal change of excitability via nonsynaptic plasticity modulates the linkage of multiple memories. Last, pre-allocation of neurons boost the memory lifetime and capacity of the network.

Overall, our model shows that synaptic plasticity is required for information storage through ensemble formation, whereas nonsynaptic plasticity modulates neuronal allocation of memory.
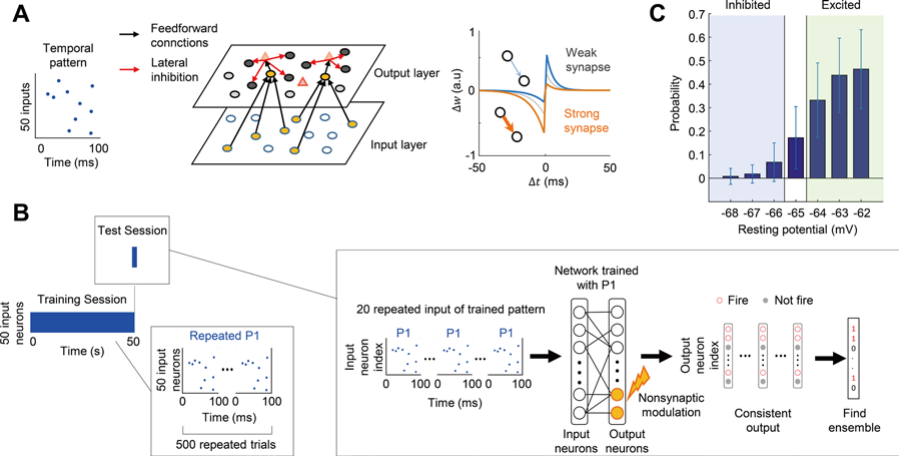


**Figure 1**. **A.** (Left) Temporal input pattern. (Middle) Spiking neural network model with lateral inhibition. (Right) Spike-timing-dependent plasticity. **B.** The network learned temporal patterns and formed a neuronal ensemble. **C.** Role of non-synaptic plasticity. Neurons excited just before learning have higher probability of being recruited into an engram

**References**

1. Mozzachiodi R, Byrne JH: More than synaptic plasticity: role of nonsynaptic plasticity in learning and memory. *Trends Neurosci* 2010, **33:**17–26.

2. Yiu AP, Mercaldo V, Yan C, Richards B, Rashid AJ, Hsiang HLL, et al.: Neurons are recruited to a memory trace based on relative neuronal excitability immediately before training. *Neuron* 2014, **83:**722–735.

## P50 Frequency- and Location-Dependence of Auditory Influence on Human Visual Perception

### Jun Ho Song^1^, Se-Bum Paik^2,3^

#### ^1^Information and Electronics Research Institute, Korea Advanced Institute of Science and Technology, Daejeon 34141, Republic of Korea; ^2^Department of Bio and Brain Engineering, Korea Advanced Institute of Science and Technology, Daejeon 34141, Republic of Korea; ^3^Program of Brain and Cognitive Engineering, Korea Advanced Institute of Science and Technology, Daejeon 34141, Republic of Korea

##### **Correspondence:** Jun Ho Song (jhs11@kaist.ac.kr)

*BMC Neuroscience* 2017, **18(Suppl 1)**:P50

The topography in primary sensory cortices fades in higher cortical regions, but a recent fMRI study reported that the activity induced by different stimuli of one sensory modality is discernable in non-pertinent sensory systems [1]. However, whether the influence of a sensory system on another sensory system is systematically organized in humans remains a question. In the present study, we hypothesized that the frequency map in human auditory perception systematically projects to the spatial map in visual perception. To test our hypothesis, we conducted a set of psychophysical experiments: Subjects were asked to perform orientation discrimination tasks with one eye closed. Visual stimuli were presented for 67 ms at various locations on the half side of the monitor screen ipsilateral to the open eye. Half a second before a visual stimulus appeared, either no acoustic stimulus or a sound with a frequency ranging from 200 Hz to 8 kHz was introduced to the ear ipsilateral to the open eye. We found that sounds within a certain frequency bandwidth significantly changed subjects’ performance compared to the no-sound condition, whereas those outside the bandwidth did not. These effects were not homogeneous across the visual space: visual perception at different visual locations had location-specific sound frequencies that substantially affected subjects’ performance, and these frequencies changed gradually across the visual space. Our results show that auditory influence on visual perception can modelled as a function of sound frequency and location in visual space. Because both the auditory and visual stimuli that we introduced began to be processed in the very early stages of the sensory systems—i.e. frequency discrimination in the primary auditory cortex and orientation discrimination in the primary visual cortex—the observations we made imply that the tonotopic organization of the auditory cortex may be matched to the retinotopic organization of the visual cortex.

**Reference**

1. Liang M, Mouraux A, Hu L, Iannetti GD. Primary sensory cortices contain distinguishable spatial patterns of activity for each sense. *Nat Commun* 2013, **4**:1979.

## P51 Developmental model for ocular dominance column seeded from retinal

### Min Song^1,2^, Se-Bum Paik^1,2^

#### ^1^Department of Bio and Brain Engineering, KAIST, Daejeon 34141, Republic of Korea; ^2^Program of Brain and Cognitive Engineering, KAIST, Daejeon 34141, Republic of Korea

##### **Correspondence:** Min Song (night@kaist.ac.kr)

*BMC Neuroscience* 2017, **18(Suppl 1)**:P51

It has been reported that an ocular dominance column and an orientation map in the primary visual cortex (V1) have a close relationship in which the ocular dominance peaks are located at the pinwheel centers in the orientation map [1]. One theoretical study suggested that the quasi-periodic hexagonal structure of the orientation map can be seeded by the moiré interference pattern between ON and OFF retinal ganglion cell (RGC) mosaics [2], but the origin of ocular dominance column structure has not been explained. Because similar hexagonal patterns were also observed in an ocular dominance column in our preliminary analysis of the experimental data, and these spatial structures are also thought to be formed before eye-opening [3], we hypothesized that the ocular dominance column, along with the orientation map, is seeded from the retinal mosaics. In this study, using computer simulation, we show that the hexagonal structure of the ocular dominance column can be developed by the moiré interference pattern of the RGC density. We designed a model in which a V1 layer is statistically wired with two different RGC layers. In the development period, the initial orientation map is first developed by contralateral wiring in the visual pathway; then, the ipsilateral wiring matches the initial orientation map during the critical period of development [4]. Because of this, we assumed that V1 cells were initially only connected with contralateral RGCs within a local convergence range and with ipsilateral RGCs within a wide convergence range (Figure 1A). By presenting drifting gratings to either the contralateral or ipsilateral RGC layers, we simulated and plotted the response of V1 (Figure 1B, C). Then, the ocular dominance map was calculated as a relative ratio between the contralateral and ipsilateral response maps of V1 (Figure 1D). We observed that the hexagonal pattern in the ocular dominance map matches the moiré interference pattern of RGC mosaics (Figure 1E). We compared this ocular dominance map with an orientation map seeded by a contralateral RGC mosaic. The results show that the ocular dominance peaks are located at pinwheel centers of the orientation map, as reported by previous experimental studies (Figure 1F). Our model shows that the initial ocular dominance map can be seeded from the periodicity of the contralateral RGC mosaic. Furthermore, we expect that the initial ocular dominance column can be sharpened during development.
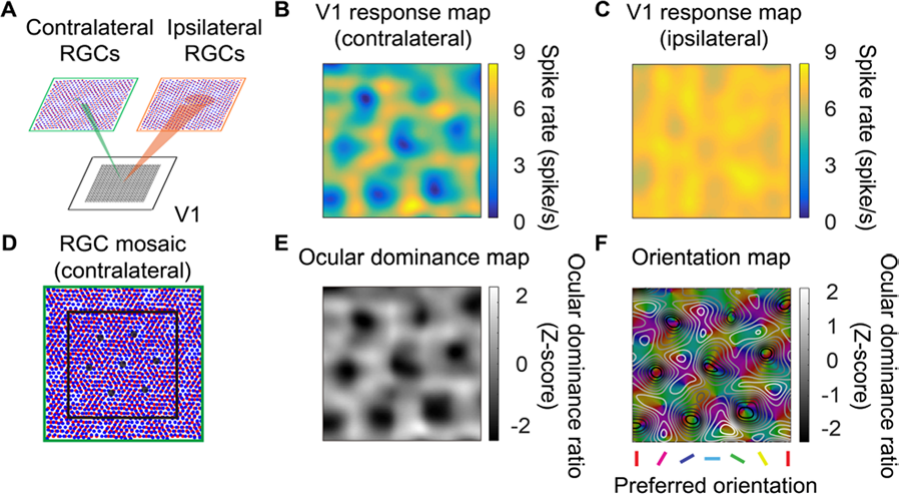


**Figure 1.** Simulation of ocular dominance column development. **A.** Schematics of RGC-cortex model. **B.** Response map of contralateral input **C.** Response map of ipsilateral input. **D.** Moiré interference periodicity of contralateral RGC. Black box indicates the net computed area of response maps to exclude boundary effects. **E.** Ocular dominance map. **F.** Relationship between ocular dominance map and orientation map seeded from contralateral RGC mosaic

**References**

1. Crair MC, Ruthazer ES, Gillespie DC, Stryker MP: Ocular dominance peaks at pinwheel center singularities of the orientation map in cat visual cortex. *Journal of Neurophysiology* **1997**, 77.6: 3381–3385.

2. Paik SB, Ringach DL: Retinal origin of orientation maps in visual cortex. *Nature neuroscience* 2011, **14.7**: 919–925.

3. Crowley JC, Lawrence CK: Early development of ocular dominance columns. *Science* 2000 **290.5495**: 1321–1324.

4. Crair MC, Gillespie DC, Stryker MP: The role of visual experience in the development of columns in cat visual cortex.” *Science* 1998 **279.5350**: 566–570.

## P52 Reliability of effective connectivity from fMRI resting-state data: discrimination between individuals

### Vicente Pallarés^1^, Matthieu Gilson^1^, Simone Kühn^2^, Andrea Insabato^1^, Gustavo Deco^1^

#### ^1^Department of Information and Communication Technologies, Universitat Pompeu Fabra, Barcelona, Spain; ^2^Max Plank Institute for Human Development, Berlin, Germany

##### **Correspondence:** Vicente Pallarés (vicente.pallares@upf.edu)

*BMC Neuroscience* 2017, **18(Suppl 1)**:P52

Neuroimaging studies traditionally analyze data at the group level, without considering individual characteristics. However, recent studies have stressed the relevance of subject-specific analysis. In particular, efforts have been made to assess the variability and reliability of brain connectivity based on fMRI data to characterize individuals [1]. Brain connectivity is typically calculated as the statistical dependence between the activity of brain regions - for example using Pearson correlation - giving matrices of functional connectivity (FC). To understand the causal interactions between regions that generate the observed FC patterns, the concept of effective connectivity (EC) has been developed [2,3]. EC reflects many biophysical mechanisms such as neurotransmitters, excitability, etc., and captures spatiotemporal information of fMRI signals.

In this work, we use fMRI resting-state data acquired from 6 subjects that underwent scanning for 50 sessions over 6 months, as well as data from 50 subjects that were scanned once. We calculate the whole-brain FC using a parcellation of 116 anatomical regions and estimate the EC for a dynamic model that reproduces the measured FC [3]. This unique dataset allows us to evaluate the variability and reliability of the EC, taken as a fingerprint of fMRI activity, and to establish a comparison with the FC [4]. Practically, we classify subjects from 1-6 sessions using their EC and FC. We train a linear classifier and are able to predict subject identity of remaining sessions. We achieve a very high identification accuracy (>90%) after training with 3 or 4 sessions with a duration of 5 min each. The better performance of the EC than the FC in discriminating between individuals demonstrates the importance of temporal information in fMRI signals and our model-based approach (Figure 1).

Beyond the theoretical understanding of brain dynamics, our results are a first step toward the clinical applicability of EC model. Our long-term goal is to provide a mechanistic explanation for neuropsychiatric disorders, allowing for the follow-up of subject-specific drug treatments or therapies based on EC measures from the non-invasive fMRI.
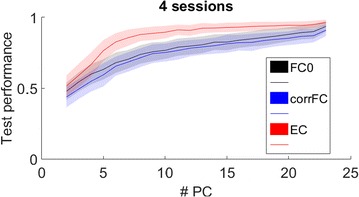


**Figure 1.** Accuracy of the classification for test sessions after training the classifier with 4 resting-state sessions. Results are shown for EC and two versions of FC: correlation (corrFC) and no-shift covariances (FC0)

**References**

1. Shehzad Z, Kelly AM, Reiss PT, Gee DG, Gotimer K, Uddin LQ, Lee SH, Margulies DS, Roy AK et al.: The resting brain: unconstrained yet reliable. *Cereb Cortex*. 2009, **19(10):**2209–2229.

2. Friston KJ: Functional and effective connectivity: a review. *Brain Connect*. 2011, **1(1):**13–36.

3. Gilson M, Moreno-Bote R, Ponce-Alvarez A, Ritter P, Deco G: Estimation of Directed Effective Connectivity from fMRI Functional Connectivity Hints at Asymmetries of Cortical Connectome. *PLoS Comput Biol*. 2016, 12(3):e1004762.

4. Finn ES, Shen X, Scheinost D, Rosenberg MD, Huang J, Chun MM, Papademetris X, Constable RT: Functional connectome fingerprinting: identifying individuals using patterns of brain connectivity. *Nat Neurosci.* 2015, **18(11):**1664–1671.

## P53 Temporal dynamics of resting state networks on a whole-brain level

### Katharina Glomb^1^, Adrián Ponce-Alvarez^1^, Matthieu Gilson^1^, Petra Ritter^2^, Gustavo Deco^1,3^

#### ^1^Center for Brain and Cognition, Department of Technology and Information, Universitat Pompeu Fabra, Carrer Ramon Trias Fargas, 25-27, 08005 Barcelona, Spain; ^2^Department of Neurology, Charité - University Medicine, Charitéplatz 1, 10117 Berlin, Germany; ^3^Institució Catalana de la Recerca i Estudis Avançats, Universitat Barcelona, Passeig Lluís Companys 23, 08010 Barcelona, Spain

##### **Correspondence:** Katharina Glomb (katharina.glomb@upf.edu)

*BMC Neuroscience* 2017, **18(Suppl 1)**:P53

FMRI BOLD signals recorded during resting state (RS) can be used to study the large-scale functional organization of the human brain [1]. This way, robust patterns of functional connectivity (FC) have been shown to exist and are termed resting state networks (RSNs) [2]. However, FC is not constant over time, and the properties and significance of its modulations are not yet understood and characterized, despite substantial interest in the topic over the last years [3]. While it seems clear that they are relevant to behavior and are at least to some extent related to underlying neural activity, there is ongoing debate as to whether they reflect nonstationarities (e.g. state switching) or not [4]. We analyzed fMRI RS data (22 min, TR = 2 s) recorded from 24 healthy controls, studying FC of 66 ROIs covering the entire cortex. With this whole-brain approach, we characterized dynamic FC (dFC) on a global level via a simple sliding-window technique. We extracted RSNs and their time courses with a dimensionality reduction technique known as tensor decomposition, which does not assume independence as ICA does [5]. We examined global dynamic modulations in the underlying BOLD signal to shed light on the mechanisms behind RSN dynamics apparent in the time courses extracted from dFC-based tensors. We show that the substantial modulations in the activity of RSNs are to a large extent explained by modulations in underlying BOLD variance and average correlation strength, establishing a tight relationship between the three measures (see Figure 1 for one subject’s example). We ask whether the modulations can be explained by stationary dynamics, using both surrogate data and a mean-field model. This way, we show that the *presence* and the *size* of modulations are explained by stationary dynamics. However, the dwell times at the peaks and troughs of the modulations are longer in the real data than expected. We conclude that in order to understand dFC, we should consider deviations from *expected* modulations rather than focusing primarily on their size, stressing the importance of appropriate null models.
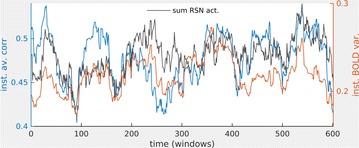


**Figure 1.** Traces of two measures of BOLD dynamics (blue: instantaneous average correlation, i.e. overall level of FC; orange: instantaneous BOLD variance, i.e. average over all brain regions’ variance) together with an RSN time course (grey)

**References**

1. Biswal B., Zerrin Yetkin F., Haughton VM., Hyde JS: Functional connectivity in the motor cortex of resting human brain using echo‐planar mri. *Magnetic resonance in medicine* 1995, **34(4):**537–541

2. Beckmann CF, DeLuca M, Devlin JT, Smith SM. Investigations into resting-state connectivity using independent component analysis. *Philosophical Transactions of the Royal Society of London B: Biological Sciences* 2005, **360(1457):**1001–1013

3. Preti MG, Bolton TAW, Van De Ville D: The dynamic functional connectome: State-of-the-art and perspectives. *NeuroImage* (in press)

4. Hindriks R, Adhikari MH, Murayama Y, Ganzetti M, Mantini D, Logothetis NK, Deco G: Can sliding-window correlations reveal dynamic functional connectivity in resting-state fMRI? *Neuroimage* 2016, **127**:242–256

5. Glomb K, Ponce-Alvarez A, Gilson M, Ritter P, Deco G: Robust extraction of spatio-temporal patterns from resting state fMRI. *bioRxiv* 2016:08951

## P54 Non-parametric estimation of network connectivity using MVAR processes in multiunit activity

### Matthieu Gilson^1^, Adria Tauste Campo^1,2^, Alexander Thiele^3^, Gustavo Deco^1,4^

#### ^1^Computational Neuroscience Group, Department de Tecnologies de la Informació i les Comunicacions, Universitat Pompeu Fabra, Barcelona, Spain; ^2^Epilepsy Monitoring Unit, Department of Neurology, Hospital del Mar Medical Research Institute, Barcelona, Spain; ^3^Institute of Neuroscience, Newcastle University, Newcastle upon Tyne, UK; ^4^Institució Catalana de Recerca i Estudis Avançats, Barcelona, Spain

##### **Correspondence:** Matthieu Gilson (matthieu.gilson@upf.edu)

*BMC Neuroscience* 2017, **18(Suppl 1)**:P54

Connectivity inference has become a cornerstone in neuroscience following the recent progress in recording techniques to characterize functional networks. New recording techniques using electrode arrays allow for the study of the simultaneous activity of distant neuronal populations. Based on our recently proposed non-parametric detection method for multivariate autoregressive (MVAR) process [1], we examine interactions between 26 electrode channels from a UTAH array implanted in a monkey performing a passive visual task. The multiunit activity envelope (MUAe) putatively reflects the spiking activity of neuronal population neighboring the electrodes, with a resolution of a few milliseconds. However, MUAe activity appears very noisy across trials – for example, it requires an averaging over many trials to exhibit differences in magnitude. Therefore, it is questionable whether MUAe conveys temporal information related to pairs of channels that could be decoded by MVAR. Our method estimates (correlated) noisy inputs received by the channels in addition to the directed connectivity between them. We find many significant interactions after the stimulus presentation, in contrast to the pre-stimulus period.

Meanwhile, we compare several types of surrogate techniques applied on the MUA time series time to build the null hypothesis of no connection in the channel network. In doing so, we also evaluate the importance of building a null distribution for each possible interaction, as compared to a single null distribution for the whole network (i.e., homogeneous test for all channel pairs). Last, we examine the stimulus-related directed interactions with the increase of MUAe activity of the source and target channels: we observe that outgoing weights are positively correlated with the channel’s activity, suggesting a gating of an underlying non-trivial connectivity by the local channel activity. The application of our method to MUAe (corresponding to high frequencies between 600 Hz and 4 kHz) complements existing techniques such as Granger causality applied to the local-field potential (1–300 Hz) for these electrode recordings.

**Acknowledgements**

MG acknowledges funding from the Marie Sklodowska-Curie Action (grant H2020-MSCA-656547). MG and GD were supported by the Human Brain Project (grant FP7-FET-ICT-604102 and H2020-720270 HBP SGA1). GD and ATC were supported by the European Research Council Advanced Grant DYSTRUCTURE 588 (Grant 295129). The authors are grateful to Robert Castelo and Inma Tur for constructive discussions.

**Reference**

1. http://biorxiv.org/content/early/2017/01/18/100669

## P55 Dependence of Absence Seizure Dynamics on Physiological Parameters

### Farah Deeba^1,2^, Paula Sanz-Leon^1,2^, P. A. Robinson^1,2^

#### ^1^School of Physics, University of Sydney, Sydney, Australia; ^2^Center for Integrative Brain Function, University of Sydney, Sydney, Australia

##### **Correspondence:** Farah Deeba (farah.deeba@sydney.edu.au)

*BMC Neuroscience* 2017, **18(Suppl 1)**:P55

A neural field model of the corticothalamic system is applied to investigate the temporal and spectral characteristics of absence seizures in the presence of a temporally varying connection strength between the cerebral cortex and thalamus. It has previously been found that increasing connection strength drives the system into seizure once a threshold is passed and a supercritical Hopf bifurcation occurs [1,2]. In this study, the dynamics and spectral characteristics of the resulting seizures are explored as functions of maximum connection strength, time above threshold, and ramp rate [3]. Figure 1 shows the outcomes of the variation of maximum connection strength. The results enable spectral and temporal characteristics of seizures to be related to underlying physiological variations via nonlinear dynamics and neural field theory. Spectral analysis reveals that the power of harmonics and duration of the oscillations increase as maximum connection strength and time above threshold increase. It is also found that the time to reach the stable limit-cycle seizure oscillation from the instability threshold decreases with the square root of the ramp rate.
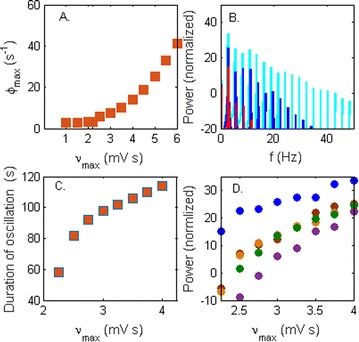


**Figure 1.** Effects of the variation of maximum connection strength. **A.** Maximum firing rate. **B.** Number of harmonics above dB during ictal state. **C.** Duration of oscillations. **D.** Power in harmonics

**Acknowledgements**

This work was supported by the Australian Research Council Center of Excellence for Integrative Brain Function Grant CE140100007, and by Australian Research Council Laureate Fellowship Grant FL140100025.

**References**

1. M. Breakspear, J. A. Roberts, J. R. Terry, S. Rodrigues, N. Mahant, P. A. Robinson: A unifying explanation of primary generalized seizures through nonlinear brain modeling and bifurcation analysis. *Cereb. Cortex* 2005, **16:** 1296–1313.

2. P. A. Robinson, C. J. Rennie, D. L. Rowe: Dynamics of large-scale brain activity in normal arousal states and epileptic seizures. *Phys. Rev. E* 2002, **64:** 041924.

3. F. Deeba, Paula Sanz-Leon, P. A. Robinson: Dependence of absence seizure dynamics on physiological parameters. *Phys. Rev. E,* submitted.

## P56 NEST-SpiNNaker comparison of large-scale network simulations

### Sacha J. van Albada^1^, Andrew Rowley^2^, Johanna Senk^1^, Michael Hopkins^2^, Maximilian Schmidt^1,3^, Alan B Stokes^2^, David R Lester^2^, Steve Furber^2^, Markus Diesmann^1,4,5^

#### ^1^Institute of Neuroscience and Medicine (INM-6) and Institute for Advanced Simulation (IAS-6), Jülich Research Centre and JARA BRAIN Institute I, Jülich, 52425, Germany; ^2^School of Computer Science, University of Manchester, Manchester, M13 9PL, UK; ^3^Laboratory for Neural Circuit Theory, RIKEN Brain Science Institute, Wako, 351-0106, Japan; ^4^Department of Psychiatry, Psychotherapy and Psychosomatics, Medical Faculty, RWTH Aachen University, Aachen, 52062, Germany; ^5^Department of Physics, Faculty 1, RWTH Aachen University, Aachen, 52062, Germany

##### **Correspondence:** Sacha J. van Albada (s.van.albada@fz-juelich.de)

*BMC Neuroscience* 2017, **18(Suppl 1)**:P56

We previously reported [1] the porting of a full-scale cortical microcircuit model [2] from the neural network simulation software NEST [3] to the digital neuromorphic hardware SpiNNaker [4] via the PyNN [5] meta-simulation language. The network contains around 80,000 leaky integrate-and-fire neurons and 0.3 billion synapses, and is thereby the network with the most connections simulated on SpiNNaker to date. The Poisson drive of the original model was replaced by a DC input. The NEST simulations were performed on a cluster using multithreading and MPI parallelism, at 0.1 ms resolution. The single-neuron and network dynamics were compared between the two simulators and with NEST simulations with precise spike timing [6] as a reference.

In this work, we further compare the performance of the two simulators in terms of speed, power, and energy consumption, controlling for accuracy. For the network simulations, achieving an accuracy comparable to that of NEST requires a slowdown of around 20 with respect to real time on the present SpiNNaker version to account for the 0.1 ms resolution and to avoid spike loss. NEST simulation speed saturates at one-third real time, but this speed is associated with an energy cost. The energy-to-solution of the NEST simulations is minimized around 96 virtual processes, for which it runs at about one-seventh real time and achieves a similar energy consumption per synaptic event to SpiNNaker for similar solution accuracy. The asynchronous update of SpiNNaker may yet confer an advantage in terms of power efficiency for even larger network simulations.

**Acknowledgements**

This project received funding from the European Union’s Horizon 2020 research and innovation programme under grant agreement No. 720270, and was previously supported by the European Union under grant agreement No. 269921 (BrainScaleS) and FP7-604102 (Human Brain Project). The design and construction of the SpiNNaker machine was supported by EPSRC (the UK Engineering and Physical Sciences Research Council) under grants EP/D07908X/1 and EP/G015740/1. Ongoing support comes from the European Research Council under the European Union’s Seventh Framework Programme (FP7/2007-2013)/ERC grant agreement 320689.

**References**

1. Van Albada SJ, Rowley AG, Hopkins M, Schmidt M, Senk J, Stokes AB, Galluppi F, Lester DR, Diesmann M, Furber SB: Full-scale simulation of a cortical microcircuit on SpiNNaker. *Front Neuroinform* Conference Abstract: Neuroinformatics 2016. doi: 10.3389/conf.fninf.2016.20.00029

2. Potjans TC, Diesmann M: The cell-type specific cortical microcircuit: relating structure and activity in a full-scale spiking network model. *Cereb Cortex* 2014, 24:785–806.

3. Eppler JM et al. NEST 2.8.0. *Zenodo* 2015, 10.5281/zenodo.32969

4. Furber SB, Lester DR, Plana LA, Garside JD, Painkras E, Temple S, Brown AD: Overview of the SpiNNaker system architecture*. IEEE Transactions on Computers* 2013, 62:2454–2467.

5. Davison A, Brüderle D, Kremkow J, Muller E, Pecevski D, Perrinet L, Yger P: PyNN: a common interface for neuronal network simulators. *Front Neuroinform* 2009, 2(11).

6. Hanuschkin A, Kunkel S, Helias M, Morrison A, Diesmann M: A general and efficient method for incorporating precise spike times in globally time-driven simulations. *Front Neuroinform* 2010, 4:113.

## P57 Temporal processing in the cerebellar cortex enabled by dynamical synapses

### Alessandro Barri^1^, Martin T. Wiechert^2^, David A. DiGregorio^1^

#### ^1^Unite d’Imagerie Dynamique du Neurone, Institut Pasteur, Paris, France; ^2^Department of Physiology, Universität Bern, Bern, Switzerland

##### **Correspondence:** Alessandro Barri (abarri@pasteur.fr)

*BMC Neuroscience* 2017, **18(Suppl 1)**:P57

The cerebellar cortex (CC) is considered to be essential for the learning of precisely timed tasks on the order of several tens of ms to a few seconds. Experimentally, this property of the cerebellum can be probed with the classical eye-blink paradigm [1] in which an animal learns to associate two stimuli that are separated by a temporal delay.

Since the classical work of Marr and Albus [2,3] the great majority of cerebellar models considers the CC as a three layered network where mossy fibres (MFs) and Purkinje cells (PCs) form the input and output layer, respectively, and granule cells (GCs) constitute a hidden layer. In this framework, temporal learning in the CC is thought to work as follows: an external input to the CC elicits temporally varying responses in GCs. PCs then weight these GC signals (by adjusting the GC-PC synapses) so as to produce the desired output [4]. This learning paradigm requires sufficiently diverse temporal signals across the GCs.

Various mechanism which generate diverse time varying signals in the GCs have been proposed [e.g. 5–7]. Recent findings have established that synaptic transmission between MFs and GCs exhibits various forms of synaptic short-term plasticity (STP) [8]. Here we show that these synaptic dynamics can provide a sufficiently rich temporal modulation of GC activity to enable temporal learning by PCs on behaviourally relevant timescales.

Our study consists of two parts. First, we re-analysed data from MF-GC dual-cell recordings from Ref. [8] with a model based inference method [9] and extracted parameters associated with pre-synaptic depression, facilitation and post-synaptic receptor desensitisation. This revealed the existence of a rich diversity of synaptic time-constants. We find that the longest of these time constants are associated with desensitisation.

In a second step, we used the experimentally obtained synaptic parameters to constrain a firing-rate-based model of the CC. In this model, GCs exhibit transient modulations of their firing rates in response to changes in MF activity. We show that these GC transients enable PCs to learn precisely timed modulations of their firing rates. The time-scales of the PC signals that can be learned are similar to those observed in behavioural responses during the eye-blink paradigm. Furthermore, when MF-GC synapses are dynamic, abrupt changes in MF activation cause model PCs to respond with sharp transient changes in their firing rates. We show that these PC responses can be interpreted as a signal of how much the sensory context provided by MFs has changed.

**References**

1. McCormick DA, Thompson RF: Cerebellum: essential involvement in the classically conditioned eyelid response. *Science,* 1984, **223**:296–299.

2. Marr D: A theory of cerebellar cortex. *The Journal of Physiology*, 1968, **202**:437–470.

3. Albus JS: A Theory of Cerebellar Function. *Mathematical Biosciences,* 1971, **10**:25–61.

4. Dean P, Porrill J, Ekerot C-F, Jörntell H: The cerebellar microcircuit as an adaptive filter: experimental and computational evidence. *Nature Reviews Neuroscience*. 2010, **11**:30–43.

5. Moore JW, Desmond JE, Berthier NE: Adaptively timed conditioned responses and the cerebellum: a neural network approach. *Biological cybernetics*, 1989, **62**:17–28.

6. Medina JF, Mauk MD: Computer simulation of cerebellar information processing. *Nature Neuroscience*, 2000, **3**:1205–1211.

7. Yamazaki T, Tanaka S. The cerebellum as a liquid state machine. *Neural Networks*, 2007, **20**:290–297.

8. Chabrol FP, Arenz A, Wiechert MT, Margrie TW, DiGregorio DA: Synaptic diversity enables temporal coding of coincident multisensory inputs in single neurons. *Nature Neuroscience*, 2015, **18**:718–727.

9. Barri A, Wang Y, Hansel D, Mongillo G: Quantifying Repetitive Transmission at Chemical Synapses: A Generative-Model Approach. *Eneuro*, 2016, **3**.

## P58 Emergence of perceptual invariances in biological sensory processing

### Alexander G. Dimitrov

#### Department of Mathematics and Statistics, Washington State University Vancouver, Vancouver, WA 98686, USA

##### **Correspondence:** Alexander G. Dimitrov (alex.dimitrov@wsu.edu)

*BMC Neuroscience* 2017, **18(Suppl 1)**:P58

A problem faced by all perceptual systems is natural variability in sensory stimuli. Some variability is irrelevant for perception, whereas other types of variability form the critical basis for distinguishing different objects. This is a common problem in sensory perception. Interpreting varied optical signals as originating from the same object requires a large degree of tolerance [1]. Understanding speech requires identifying phonemes, such as the consonant/g/, that constitute spoken words. A/g/is perceived as a/g/, despite tremendous variability in acoustic structure that depends on the surrounding vowels and consonants [2]. The main goal of an object recognition problem is the ability to identify individual objects while invariant to changes stemming from multiple transformations.

To model invariant representation in sensory systems, we model the represented probability of sensory stimuli as a distribution over stimulus features *w*, $p(w)$ jointly with a distribution $p(\tau ) = \prod_{i} p(\tau_{i} )$ of transformations *g*(*τ*) acting independently on the features. The ensemble of features {*s*_*j*_} is considered to have been drawn from the feature distribution $p(w)$, with transformations $g_{k} \equiv g_{k} (\tau_{i} )$ applied to the sound features, so that $s_{j} = g_{1} g_{2} \ldots g_{n} w_{j} .$

This probabilistic stimulus representation allows a straightforward expansion for the degree of invariance. Consider a population of locally invariant (transformation-tuned) feature detectors, each representing the probability $p\left( {w |w_{0} } \right) p(\tau |\mu )$ for a signal having a specific feature $w_{0}$, but a separate preferred transformation distribution $p\left( {\tau |\mu } \right)$. For example, $\mu$ can be the preferred scale or position of the feature. Invariance extension is natural in this formalism: we define a broad region of transformation parameters, $\varOmega$, and a distribution over preferred means, $p(\mu )$, which is essentially uniform over $\varOmega$. With this addition, a set of locally invariant units with $\mu \in \varOmega$ can be combined to a unit invariant for all transformations with $\tau \in \varOmega$ by the simple act of marginalization over preferences,

1 $$ p_{\varOmega } \left( \tau \right) = E_{p\left( \mu \right)} p\left( {\tau |\mu } \right) = \mathop \smallint \limits_{\mu \in \varOmega }^{{}} p\left( {\tau |\mu } \right)p\left( \mu \right) $$

When only one or a few transformations are marginalized, the system will be more invariant to those transformations, while retaining its degree of covariance to other transformations. This process realizes a mixture model of feature or template detectors with different preferred transformations.

The theoretical aspects of marginalization are deceptively simple: according to Eq. (1), a linear operation (weighted sum when discretized) leads to invariant stimulus representation. Instantiating the theory in the neural context is more involved. While we posit that neural activity represents probabilities of stimuli and transformations, what is available to other parts of the nervous system is a specific sample from that probability, the neural population vector response, $r = \left( {r_{1} ,r_{2} ,r_{3} , \ldots r_{n} } \right)$. The question that needs to be solved then is what operation should be performed on $r$, such that the resultant $R = f\left( r \right)$ has the desired distribution from Eq. (1)? In other words, how do neuronal populations actually achieve invariance through marginalization?

To address this question, we use probabilistic population coding (PPC, [3]), a model of neural population coding that has the capacity of performing the necessary marginalization. With PPC, it has been shown conceptually how marginalization of two distinct transformations can be realized through divisive normalization. We innovate in two aspects. First, we use this model approach as an explanatory tool for specific brain areas, rather than the conceptual example provided in [3]. Second, we generalize the results to multiple transformations, as required by Eq. 1.

**References**

1.Rust NC, DiCarlo JJ: Selectivity and tolerance (“invariance”) both increase as visual information propagates from cortical area V4 to IT. *Journal of Neuroscience* 2010, **30(39):**12978–12995.

2. Diehl RL, LottoAJ, Holt LL: Speech Perception *Annu. Rev. Psychol.* 2004, **55:**149–79.

3. Beck JM, Latham PE, Pouget A: Marginalization in neural circuits with divisive normalization. *J. Neurosci.* 2011, **31:**15310–15319.

## P59 A non-linear stochastic strategy to estimate synaptic conductances under the presence of subthreshold ionic currents

### Catalina Vich^1^, Rune W. Berg^2^, Antoni Guillamon^3^, Susanne Ditlevsen^4^

#### ^1^Department of Mathematics and Computer Science, Universitat de les Illes Balears, Palma, 07122, Spain; ^2^Department of Neuroscience and Pharmacology, University of Copenhagen, Copenhagen, 2100, Denmark; ^3^Department of Applied Mathematics I, EPSEB, Universitat Politècnica de Catalunya, 08028, Barcelona, Spain; ^4^Department of Mathematical Science, University of Copenhagen, Copenhagen, 2100, Denmark

##### **Correspondence:** Catalina Vich (catalina.vich@uib.es)

*BMC Neuroscience* 2017, **18(Suppl 1)**:P59

Unveiling the information that a neuron receives from other neurons and distinguishing between excitatory and inhibitory inputs is an important task in neuroscience as it provides valuable information on local connectivity and brain operating conditions. Experimentally, the synaptic conductances are difficult to estimate due to the diversity of synaptic inputs and their unattainable conductances. Different linear inverse methods have been proposed to solve this problem, such as [1–3].

It has been reported that linear models provide poor estimates in spiking regimes (see [4]), but they can also be poor if ionic currents are active in the subthreshold regime (see [5]). Thus, taking a linear model as a generic one to estimate conductances does not seem a valid strategy in all situations; even with some data treatment, such as filtering the observed trace, the transformed dynamics cannot be assumed to follow a linear model.

A deterministic strategy has been developed taking into account quadratic terms (see [5]), which seems to improve estimations under the presence of subthreshold fluctuations. However, the method does not incorporate noise and, moreover, it requires the use of two voltage traces from different trials, which can lead to some misestimations.

In this work, we propose a new strategy to estimate synaptic conductances, which has been tested using in silico data and applied to in vivo recordings. The model is constructed to capture the non-linearities caused by subthreshold activated currents, and the estimation procedure can discern between excitatory and inhibitory conductances using only one membrane potential trace. More precisely, we perform second order approximations of biophysical models to capture the subthreshold non-linearities, resulting in quadratic integrate-and-fire models, and apply approximate maximum likelihood estimation where we only suppose that conductances are stationary in a 50 ms time window. The results show good estimations when applied to different computational models of endowed with different subthreshold ionic currents. Moreover, we also obtain an improvement when we compare the proposed estimation procedure with a linear method with similar features and an oversampling method.

**References**

1. Bédard C., Béhuret S, Deleuze C, Bal T, Destexhe A: Oversampling method to extract excitatory and inhibitory conductances from single-trial membrane potential recordings. *Journal of neuroscience methods* 2011, **210** (1).

2. Berg RW, Ditlevsen S: Synaptic inhibition and excitation estimated via the time constant of membrane potential fluctuations. *Journal of Neurophysiology* 2013, 110 (4).

3. Lankarany M, Heiss JE, Lampl I, Toyoizumi T: Simultaneous Bayesian estimation of excitatory and inhibitory synaptic conductances by exploiting multiple trails. *Frontiers in Computational Neuroscience* 2016, **10:**110.

4. Guillamon A, McLaughlin DW, Rinzel J: Estimation of synaptic conductances. *Journal of Physiology*-*Paris* 2006, **100** (1–3)

5. Vich C, Guillamon A: Dissecting estimation of conductances in subthreshold regimes. *Journal of Computational Neuroscience* 2015, **39** (3).

## P60 Involvement of randomness in reinforcement learning

### Romain D. Cazé^1^, Benoît Girard^1^, Stéphane Doncieux^1^

#### ^1^ISIR, Université Pierre et Marie Curie, Paris, 75005, France

##### **Correspondence:** Romain D. Cazé (romain.caze@gmail.com)

*BMC Neuroscience* 2017, **18(Suppl 1)**:P60

Animals could differently respond when confronted two times to the exact same situation. Classic reinforcement learning agents implement this randomness using a constant parameter. Most of the time, the agent picks the action with the highest value to maximize its reward, and a fraction of the time, regulated by the previous parameter, it randomly picks an action. This randomness enables an agent to probe unexplored options and help to address the exploration-exploitation trade off. While this approach successfully explains how animals can behave randomly, it fails to replicate the non-uniform variation of performances observed from one day to another. For instance, performances at the end of the previous day could be higher than at the beginning of the next day. To reproduce this daily variation, we use here a parameter that varies periodically from session to session. First, we compare on a single session three types of agent performing the standard armed-bandit problem. For these three agents, the parameter setting randomness takes either: (1) a low (2) a high (3) or a decreasing value over a session and we show that this latter type of agent collects more reward than the others. We reset the parameter regulating our third agent randomness between every session to mimic a resting period. Second, we demonstrate that in a session where agents have already learned another armed-bandit problem different from the previous session. The third type of agent still performs best and remains unaffected by the variation that is not the case especially for the first type of agent. Our work paves the way for a new type of agent with periodic variations of its choices randomness.

## P61 Modelling the impact of dendritic spine geometry on electrical and calcic signalling with the Finite Element Method

### Nicolas Doyon, Frank Boahen

#### Department of Mathematics and Statistics, Laval University, Quebec, Canada, G1V 0A6

##### **Correspondence:** Nicolas Doyon (nicolas.doyon@mat.ulaval.ca)

*BMC Neuroscience* 2017, **18(Suppl 1)**:P61

The complex geometry of neural sub compartments such as dendritic spines and nodes of Ranvier play important roles in calcium and electrical signaling. The usual multi compartment approach fails to accurately describe electro-diffusion in such domains. A way to obtain more accurate results and to describe the spatial distribution of ionic concentrations and electrical potential up to a nanometric resolution is to solve the Poisson Nernst Planck equations with the Finite Element Method (FEM). Given that applying this technic on complex three dimensional geometries can rapidly lead to prohibitive computational cost, mathematical tools from the field of numerical analysis are required. We present how such a tool, automatic mesh adaptation, can improve solution accuracy in an electrodiffusion model of a node of Ranvier [1]. We then describe electrical and calcium signalling in a dendritic spine with the FEM. Spine geometry varies greatly from one spine to another as well as during synaptic potentiation but the functional roles of this geometry are still not fully understood [2]. We show how FEM based models provide an ideal tool to investigate this question [3]. Using models with different geometries, we finally obtain relationships between the geometry of the spine and properties of calcium as well as electrical signalling.

**Acknowledgements**

A The work here presented was supported by the National Science and Engineering Research Council of Canada (NSERC)

**References**

1. I Dione, J Deteix, T Briffard, E Chamberland, N Doyon: Improved Simulation of Electrodiffusion in the Node of Ranvier by Mesh Adaptation. *PloS one* 2016,11(8).

2. R Yuste: Electrical compartmenatalization in dendritic spines. *Annu Rev Neurosci.* 2013, **36:**429–49.

3. D Holcman, R Yuste: The new nanophysiology: regulation of ionic flow in neuronal subcompartments. *Nat Rev Neurosci.* 2015, **16(11)**:685–92.

## P62 Resilience in dynamical neural networks with synaptic adaptation

### Patrick Desrosiers^1,2^, Edward Laurence^2^, Nicolas Doyon^1,3^, Louis J. Dubé^2^

#### ^1^Centre de recherche de l’Institut universitaire en santé mentale de Québec, Québec, Québec, Canada, G1J 2G3; ^2^Département de physique, de génie physique et d’optique, Université Laval, Québec, Québec, Canada G1V 0A6; ^3^Département de mathématiques et de statistique, Université Laval, Québec, Québec, Canada G1V 0A6

##### **Correspondence:** Patrick Desrosiers (Patrick.desrosiers.1@ulaval.ca)

*BMC Neuroscience* 2017, **18(Suppl 1)**:P62

The brain is a notorious resilient system. After minor strokes, for example, parts of the brain reorganize their structural connectivity and essentially recover their original functions. Although some dynamical effects of brain network failures on their activity have been found [1], most studies about resilient neural systems have so far focused on purely topological properties of connectomes. This is due in part to the inherent high-dimensionality of dynamical neural systems. Recent progresses suggest however that the resilience analysis of many complex dynamical systems can be dramatically simplified by dimension reductions resulting from mean-field approximations [2,3]. We extend these previous works to study models of neural networks in which neurons and synaptic weights are dynamical variables. In our framework, the dynamics of a network with *N* neurons is described by *N*(*N* + 1) nonlinear coupled ODEs that govern the fast evolution of the neural activity (e.g., firing-rates) as well as the slow adaptation of the synaptic weights (e.g., Hebbian potentiation with saturation). Two global variables, the effective activity $x_{eff}$ and the effective synaptic weight $\beta_{eff}$, are used for predicting the global evolution of the whole system. We prove, both numerically and theoretically, that $x_{eff}$ captures more accurately the behavior of the network than the usual mean network activity. When the synaptic adaptation is neglected, the resilience analysis can be easily done with bifurcation diagrams as in Figure 1A. Structural perturbations, such as weak or strong attacks that respectively change weights or break synaptic connections, result in a modification of $\beta_{eff}$. If the latter reaches some critical value, $\beta_{c}$, the system undergoes a sudden transition and loses its resilience. This is numerically confirmed in Figure 1B. As illustrated in Fig 1C, the addition of synaptic adaptation leads to the emergence of new resilience patterns and often facilitates the recovery of the original network activity.
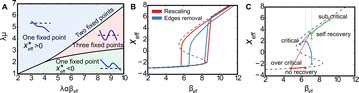


**Figure 1. A.** Typical bifurcation diagram for the effective model without synaptic adaptation, where $\varvec{\alpha},\varvec{\lambda},\varvec{\mu}$ are dynamical parameters regulating the neural dynamics while $\varvec{\beta}_{{\varvec{eff}}}$ is effective synaptic weight. **B.** Global effective activity at equilibrium after weak (red line) or strong (blue line) attacks on static synaptic connections compared to the theoretical hysteresis curve (dashed line) obtained from mean-field theory. **C.** Same as **B** but with synaptic adaptation. The square, stars, and triangles respectively denote the equilibria before an attack, just after an attack but before adaption, and after adaptation. Green line: resilience enabled by adaptation. The numerical solutions in **B** and **C** were produced from small random networks with 200 neurons and connectivity density $\varvec{p} = 0.2$

**Acknowledgements**

FRQNT, NSERC, and the Sentinel North program supported by the Canada First Research Excellence Fund.

**References**

1. Joyce KE, Hayasaka S, Laurienti PJ: The human functional brain network demonstrates structural and dynamical resilience to targeted attack. *PLoS Comput Biol* 2013, ***9*** **(1):** e1002885 1–11.

2. Majdandzic A, Podobnik B, Buldyrev SV, Kenett DY, Havlin S, Stanley HE: Spontaneous recovery in dynamical networks. *Nature Physics* 2014, **10(1):**34–38.

3. Gao J, Barzel B, Barabási AL: Universal resilience patterns in complex networks. *Nature* 2016, **530(7590):**307–312.

## P63 Cell assemblies: a computational challenge

### Russo Eleonora, Daniel Durstewitz

#### Department of Theoretical Neuroscience, ZI - Central Institute for Mental Health, Mannheim, 68159, Germany

##### **Correspondence:** Russo Eleonora (eleonora.russo@zi-mannheim.de)

*BMC Neuroscience* 2017, **18(Suppl 1)**:P63

More than half a century ago, Hebb proposed that neurons may organize into coherent spatio-temporal activity patterns (‘cell assemblies’) to represent mental entities. Only recently, with the advance of multiple single-unit recording techniques, this core concept of computational and cognitive neuroscience has become experimentally accessible. From a statistical perspective, however, detecting these patterns in data still remains a major challenge: the presence of non-stationarity, the combinatorial explosion of multi-unit pattern configurations and the resulting necessity of a fast statistical test are only some of the difficulties to be faced when detecting cell assemblies. Here we present a novel mathematical framework that captures assembly structure at different temporal scales, levels of precision, and with arbitrary internal organization. Applying this methodology to multi-cell recordings from various brain areas we found that there is no universal cortical coding scheme, but that assembly structure strongly differs with brain area recorded and current task demands.

## P64 Reconstructing neural dynamics from experimental data using radial basis function recurrent neural networks

### Dominik Schmidt, Daniel Durstewitz

#### Department of Theoretical Neuroscience, Bernstein Center for Computational Neuroscience, Central Institute of Mental Health, Medical Faculty Mannheim, Heidelberg University, Heidelberg, Germany

##### **Correspondence:** Dominik Schmidt (dominik.schmidt@zi-mannheim.de)

*BMC Neuroscience* 2017, **18(Suppl 1)**:P64

Neural recordings are often very complex, noisy and high-dimensional signals. Modern data acquisition techniques allow for simultaneous recordings from up to hundreds of units over many trials. To assess underlying network mechanics and dynamics, one has to analyze the population as a whole, for example, by reducing the dimensionality of the data [1]. In addition, neural responses are highly noisy and often fluctuate significantly between trials, even when experimental conditions are unchanged. These fluctuations may encode relevant behavioral information, such that simple averaging over trials could potentially smooth out and obscure behaviorally important aspects of neural dynamics [2]. A popular class of methods to reduce dimensionality while analyzing data on a trial by trial basis is the statistical framework of State Space Models (SSMs) [3]. The idea behind SSMs is that there is an underlying latent dynamical system generating the observations, with latent dynamics and observations having separate noise terms. While linear SSMs are widely used to recover hidden neural trajectories [4], they are only able to reproduce the linear aspects of the underlying neural dynamics. They are thus not powerful enough to capture the underlying dynamical system itself [5].

For that reason, we use a nonlinear SSM that includes radial basis functions (RBF) for the latent state dynamics, originally developed in [6]. With such a RBF expansion, arbitrary dynamical systems can be approximated [6], which potentially not only allows for dimensionality reduction and retrieving hidden neural trajectories, but also for reproducing the underlying dynamical system itself. To estimate parameters and hidden states of the model, an Expectation Maximization (EM) algorithm together with an Extended Kalman Filter-Smoother is used [6]. One advantage of this method is that all steps of the algorithm have a closed form analytical expression, resulting in computationally efficient parameter estimation that does not depend on computationally expensive numerical methods.

To assess the validity of the method and explore its capabilities, it is first applied to synthetically generated data from a number of different dynamical systems, including multistable, oscillatory and chaotic systems. In addition to this synthetic data, the method is probed on experimental data. This enables a detailed analysis of attractor dynamics within the observed regions and potentially yields not only a descriptive model, but also a predictive one.

**Acknowledgements**

The work was funded by the German Research Foundation within the CRC 1134 (D01) and by the Federal Ministry of Education and Research (BMBF; 01ZX1311A)

**References**

1. Cunningham JP and Yu B M: Dimensionality reduction for large-scale neural recordings. *Nature Neuroscience* 2014, **17(11)**, 1500–1509.

2. Latimer KW, Yates JL, Meister MLR, Huk AC, Pillow JW: Single-trial spike trains in parietal cortex reveal discrete steps during decision-making. *Science* 2013, **349(6244),** 184–187.

3. Durstewitz D, Koppe G, Toutounji H: Computational models as statistical tools. *Current Opinion in Behavioral Sciences* 2016, **11**, 93–99.

4. Yu BM, Cunningham JP, Santhanam G, Ryu SI, Shenoy KV, Sahani M: Gaussian-process factor analysis for low-dimensional single-trial analysis of neural population activity. *Journal of Neurophysiology* 2009, **102(1)**, 614–635.

5. Durstewitz D: A State Space Approach for Piecewise-Linear Recurrent Neural Networks for Reconstructing Nonlinear Dynamics from Neural Measurements 2016, arXiv:1612.07846 [q-bio.NC]

6. Roweis S, Ghahramani Z: Learning Nonlinear Dynamical Systems Using the Expectation–Maximization Algorithm, in *Kalman Filtering and Neural Networks* 2001 (ed S. Haykin), John Wiley & Sons, Inc., New York, USA.

## P65 Layer V pyramidal cells as mediators of delta oscillations: Insights from biophysically detailed modeling and connections with schizophrenia genetics

### Tuomo Mäki-Marttunen^1^, Florian Krull^1^, Francesco Bettella^1^, Christoph Metzner^2^, Anna Devor^3,4^, Srdjan Djurovic^5^, Anders M. Dale^3,4^, Ole A. Andreassen^1^, Gaute T. Einevoll^6,7^

#### ^1^NORMENT, Institute of Clinical Medicine, University of Oslo, Oslo, Norway; ^2^Centre for Computer Science and Informatics Research, University of Hertfordshire, Hatfield, UK; ^3^Department of Neurosciences, University of California San Diego, La Jolla, CA, USA; ^4^Department of Radiology, University of California San Diego, La Jolla, CA, USA; ^5^Department of Medical Genetics, Oslo University Hospital, Oslo, Norway; ^6^Faculty of Science and Technology, Norwegian University of Life Sciences, Ås, Norway; ^7^Department of Physics, University of Oslo, Oslo, Norway

##### **Correspondence:** Tuomo Mäki-Marttunen (tuomomm@uio.no)

*BMC Neuroscience* 2017, **18(Suppl 1)**:P65

Delta oscillations (0.5–4 Hz) are widely distributed brain oscillations that are observable with electroencephalogram (EEG) measurements during sleep and mental tasks. They seem to have two components, one thalamically generated and one originating from the neocortex [1]. The thalamically generated delta oscillation stems solely from the intrinsic properties of the thalamocortical neurons, while the cortically generated delta oscillations likely rely on the intrinsic properties of layer V pyramidal cells (L5PCs) [1]. Moreover, L5PCs integrate large numbers of inputs from thalamic nuclei [2] and could therefore play a crucial role in maintaining the thalamically generated delta as well.

Due to the pivotal role of L5PCs as hubs integrating information from nearby and distant brain areas, altered L5PC activity has been suggested as the reason behind faulty perceptions, such as hallucinations, in mental disease [2]. Importantly, schizophrenia (SCZ) patients show elevated power in delta oscillations, which may also be a sign of altered L5PC firing. The recent genome-wide association studies confirm the contribution of a large set of ion-channel (both synaptic and non-synaptic) and calcium-transporter-encoding genes to risk of SCZ [3].

In this work, we study the contributions of the intrinsic processes of L5PCs to the generation and maintenance of delta oscillations using biophysically detailed modeling. We employ models of single L5PCs and networks of coupled L5PCs [4]. The single-cell models are multi-compartmental models that include description of Ca^2+^ dynamics and Hodgkin-Huxley type of kinetics for many types of ion channels. The network model [4] includes the description of L5PC-to-L5PC glutamatergic synapses. We employ a reduced version of this model [5] to boost up the simulation speed. We modify the parameters of these models in a way that mimics the small effects that are expected  to be observed in common variants associated with SCZ [6]. We show that the L5PC network gain and the responses of the network to delta oscillations are altered by variants of many SCZ-associated ion-channel and Ca^2+^-transporter-encoding genes. In a similar fashion, we study the effects of differential gene expression by varying the conductances of the ion-channel species that correspond to genes whose expression in blood sample data of SCZ patients deviated from that of healthy controls. Our results deepen the understanding of altered delta power in SCZ patients and could ultimately aid the development of novel future treatments of the mental disease.

**References**

1. Neske GT. The Slow Oscillation in Cortical and Thalamic Networks: Mechanisms and Functions. *Front Neural Circ* 2015;9:88.

2. Larkum M. A cellular mechanism for cortical associations: an organizing principle for the cerebral cortex. *Trends Neurosci* 2013;36.3:141–51.

3. Ripke S, Sanders AR, Kendler KS, Levinson DF, Sklar P, Holmans PA, Lin DY, Duan J, Ophoff RA, Andreassen OA et al.: Genome-wide association study identifies five new schizophrenia loci. *Nat Gen* 2011, 43:969–976.

4. Hay E, Segev I. Dendritic Excitability and Gain Control in Recurrent Cortical Microcircuits. *Cereb Cortex* 2015;25.10:3561–71.

5. Mäki-Marttunen T, Halnes G, Devor A, Metzner C, Dale AM, Andreassen OA, Einevoll GT. Step-wise model fitting accounting for high-resolution spatial measurements: Construction of a layer V pyramidal cell model with reduced morphology. *BMC Neuroscience*, 17(Suppl 1):P165, 2016.

6. Mäki-Marttunen T, Halnes G, Devor A, Witoelar A, Bettella F, Djurovic S, Wang Y, Einevoll GT, Andreassen OA, Dale AM. Functional Effects of Schizophrenia-Linked Genetic Variants on Intrinsic Single-Neuron Excitability: A Modeling Study. *Biological Psychiatry: Cognitive Neuroscience and Neuroimaging*. 2016;1:49–59.

## P66 Biophysical modeling of single-neuron contributions to ECoG and EEG signals

### Solveig Næss^1,2^, Torbjørn V Ness^3^, Geir Halnes^3^, Eric Halgren^4^, Anders M Dale^4^ and Gaute T Einevoll^3,5^

#### ^1^Department of Informatics, University of Oslo, Oslo, Norway; ^2^Simula-UiO-UCSD Research and PhD (SUURPh) training program, Oslo, Norway; ^3^Faculty of Science and Technology, Norwegian University of Life Sciences, Ås, Norway; ^4^Department of Neuroscience and Radiology, School of Medicine, UC San Diego, CA, USA; ^5^Department of Physics, University of Oslo, Oslo, Norway

##### **Correspondence:** Solveig Næss (solvenae@ifi.uio.no)

*BMC Neuroscience* 2017, **18(Suppl 1)**:P66

Electroencephalography (EEG), i.e., recordings of electrical potentials at the scalp, and electrocorticography (ECoG), i.e., potentials recorded on the cortical surface, are two prominent techniques probing brain activity at the systems level. Despite their long history and widespread use, the proper interpretation of these brain signals in terms of the biophysical activity in underlying neurons (nerve cells) and neuronal networks is still lacking. Present-day analysis is predominantly statistical and limited to identification of phenomenological signal generators without a clear biophysical interpretation. New biophysics-based analysis methods are thus needed to take full advantage of these brain-imaging techniques [1].

Here we used biophysical modeling based on morphologically detailed multicompartmental neuron models to explore single-neuron contributions to ECoG and EEG signals and in particular the feasibility of using the so-called current-dipole approximation in predicting these signals [2]. Specifically, we used the open-source Python package LFPy [3] which builds on Neuron [4] and is based on well-established volume-conductor theory for numerical calculations of extracellular potentials. The LFPy package was supplemented with new Python tools for calculating the current-dipole moment of a neuron for use of the current-dipole approximation to predict ECoG and EEG signals. Current-dipole approximations were explored in the inhomogeneous four-concentric-spheres head model [5], and compared with results from using the Finite Element Method [6].

When comparing computed cortical-cell contributions to the EEG and ECoG signals from using the current-dipole approximation with results from the full model explicitly including all transmembrane currents, we find that the current-dipole approximation is applicable for modeling EEG signals. This allows for a drastic simplification of future biophysics-based computation of EEG signals from cortical cell populations. However, we find that the current-dipole approximation is not generally applicable for computing ECoG signals.

**References**

1. Einevoll GT, Kayser C, Logothetis NK, Panzeri S: Modelling and analysis of local field potentials for studying the function of cortical circuits. *Nat Rev Neurosci 2013*, **14:**770-785.

2. Hämäläinen M, Hari R, Ilmoniemi RJ, Knuutila J, Lounasmaa OV: Magnetoencephalography – theory, instrumentation, and applications to noninvasive studies of the working human brain. *Rev Mod Phys 1993*, **65:**413–505.

3. LFPy [lfpy.github.io]

4. NEURON [www.neuron.yale.edu]

5. Srinivasan R, Nunez PL, Silberstein RB: Spatial filtering and neocortical dynamics: estimates of EEG coherence. *IEEE Trans Biomed Eng 1998*, **45:** 814–826 (Srinivasan et al., IEEE Trans Biomed Eng, 1998)

6. Larson MG, Bengzon F: The Finite Element Method: theory, implementations and applications. Heidelberg: Springer; 2013.

## P67 Extracellular diffusion can introduce errors in current source density estimates

### Geir Halnes^1^, Tuomo Mäki-Marttunen^2^, Klas H Pettersen^3,4^,Ole A Andreassen^2^, Gaute T Einevoll^1,5^

#### ^1^Faculty of Science and Technology, Norwegian University of Life Sciences, Ås, Norway; ^2^NORMENT, Institute of Clinical Medicine, University of Oslo, Oslo, Norway; ^3^Letten Centre and Glialab, Department of Molecular Medicine, Inst. of Basic Medical Sciences, University of Oslo, Oslo, Norway; ^4^Centre for Molecular Medicine Norway, University of Oslo, Oslo, Norway; ^5^Department of Physics, University of Oslo, Oslo, Norway

##### **Correspondence:** Geir Halnes (geir.halnes@nmbu.no)

*BMC Neuroscience* 2017, **18(Suppl 1)**:P67

A standard way to study neuronal activity is to record the local field potential (LFP) in the extracellular space (ECS) surrounding active neurons. Theoretical methods, such as current source density (CSD) theory can then be used to infer the distribution of neuronal current sources from recorded potentials. When estimating the CSD, several assumptions are made. Typically, one assumes a spatially homogeneous neuronal activity level and a constant extracellular conductivity. Another important assumption is that ionic diffusion in the ECS has a negligible impact on the LFP, so that the recorded potentials exclusively reflect underlying cellular current sources [1].

As the charge carriers in brain tissue are ions, diffusion and electrical migration are in reality interdependent processes. The assumption that diffusion has a negligible impact on the LFP could therefore be challenged, especially under conditions when concentration gradients in the ECS become large. Large extracellular concentration gradients are symptomatic for many pathological conditions, but periods of intense neural signalling can evoke concentration shifts of several millimolars even in non-pathological cases [2].

By means of biophysical modelling, we here explore the error introduced in CSD estimates by neglecting effects from diffusion currents. We use a the previously developed electrodiffusive Kirchhoff-Nernst-Planck formalism [3], which allows us to simulate the dynamics of the electrical potential and of the ion concentrations in the ECS surrounding a neural population [4]. In this in silico scenario, the true CSD (i.e., the spatiotemporal distribution of neuronal transmembrane currents in the model) is known, and can be compared to the conventional CSD estimate (based only on the LFP), and an alternative CSD estimate which also accounts for diffusion dependent effects. We find that the electrodiffusive CSD estimate accurately predicts the true CSD, while the conventional CSD estimate dramatically deviates from the true CSD when extracellular concentration gradients become large, and can lead to the prediction of spurious current sources (Figure 1).
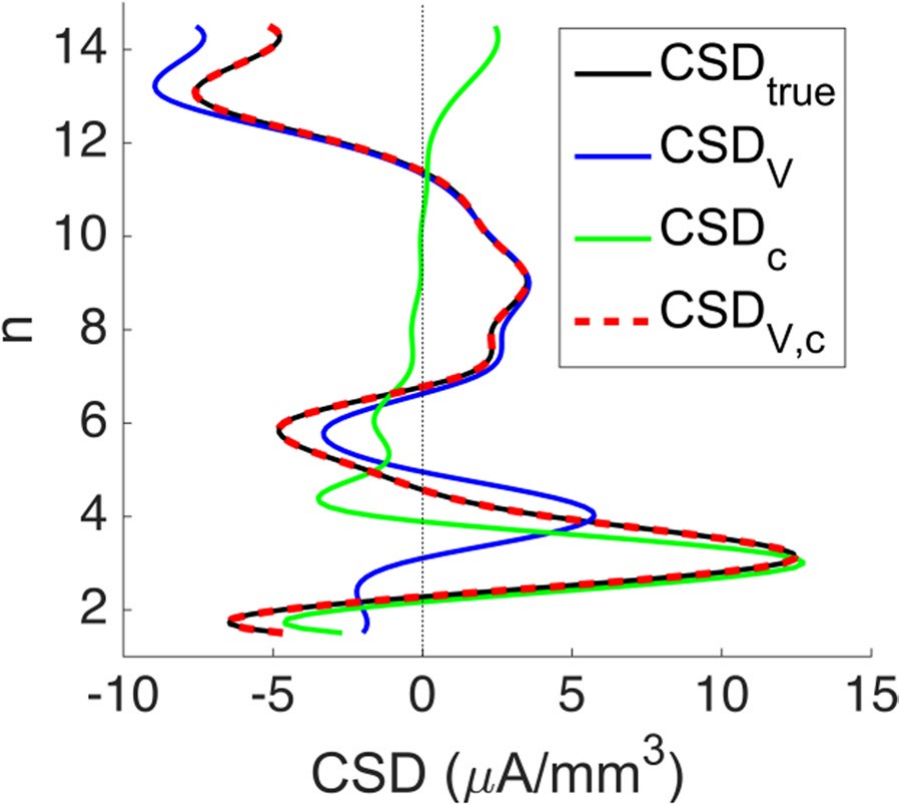


**Figure 1**. Temporally averaged CSD estimates at different locations (n = 1 is the bottom and n = 15 is the top of a cortical column). Black line: True CSD. Blue line: Conventional CSD estimate (double spatial derivative of the LFP). Red line: Improved, electrodiffusive CSD estimate. Green line: CSD correction imposed by diffusion

**References**

1. Gratiy SL, Halnes G, Koch C, Hawrylycz MJ, Einevoll GT, Anastassiou CA: The theory of current-source density analysis in brain tissue. *European Journal of Neuroscience* 2017. doi: 10.1111/ejn.13534.

2. Kofuji P, Newman EA: Potassium buffering in the central nervous system: *Neuroscience* 2004, **129.4**: 1043–1054.

3. Halnes G, Østby I, Pettersen KH, Omholt S & Einevoll GT: Electrodiffusive model for astrocytic and neuronal ion concentration dynamics. *PLoS Comput Biol.* 2013, **9(12):** e1003386.

4. Halnes G, Mäki-Marttunen T, Keller D, Pettersen KH, Andreassen OA, Einevoll GT: Effect of Ionic Diffusion on Extracellular Potentials in Neural Tissue. *PLoS Comput Biol.* 2016, **12(11):** e1005193.

## P68 Estimation of metabolic oxygen consumption from optical measurements in cortex

### Marte J. Sætra^1^, Anders M Dale^2^, Anna Devor^2^, Gaute T Einevoll^1,3^

#### ^1^Department of Physics, University of Oslo, Oslo, 0316, Norway; ^2^Department of Neurosciences, UC San Diego, La Jolla, California, 92093-0021, USA; ^3^Faculty of Science and Technology, Norwegian University of Life Sciences, Ås, 1433, Norway

##### **Correspondence:** Marte J. Sætra (m.j.satra@fys.uio.no)

*BMC Neuroscience* 2017, **18(Suppl 1)**:P68

The cerebral metabolic rate of oxygen (CMRO_2_) is an important parameter for understanding how the brain responds to changes in metabolism and oxygen delivery. Such changes are associated with clinical conditions like stroke and Alzheimer’s disease. An estimate of the oxygen consumption rate is further important for the interpretation of functional magnetic resonance imaging. Despite the obvious need of an O_2_ consumption measure, there happens to be no standardized way of measuring it. This is true for both the steady-state situation and for measuring dynamic CMRO_2_ changes.

Common practice varies and relies on measurements of both blood flow and oxygenation. The CMRO_2_ parameter is estimated by analysing the measurements within the context of an appropriate mathematical model. All methods of estimating CMRO_2_ are essentially “solving a mass balance equation where CMRO_2_ is equated to the difference of oxygen flowing into a region of interest and the oxygen flowing out” [1]. Estimating CMRO_2_ is therefore a complex task where inaccuracies of both experimental methods and mathematical models need to be evaluated.

Here, we present a more direct method for estimating CMRO_2_. It enables us to extract the CMRO_2_ parameter from a single quantity only, by fitting Poisson’s equation to measurements of O_2_ partial pressure (pO_2_) around vessels. Earlier attempts of doing the same has been limited by the inability to measure tissue pO_2_ with adequate resolution.

The development of two-photon phosphorescence lifetime microscopy has recently made us overcome this limitation [1].

Using pO_2_ measurements of this kind, we have studied the Krogh method for steady-state CMRO_2_ [2]. For the Krogh method we assume and axisymmetric, cylindrical geometry of the vessel-tissue region [3]. The assumption leads to a model describing pO_2_ as a function of the distance to vessel. The Krogh method, mostly used to study muscles earlier, shows disconcerting results when applied to data from brain tissue [2]. The results indicate that the method is not robust.

We introduce the Laplace method as an alternative way of estimating CMRO_2_. The method states that CMRO_2_ can be estimated by taking the second derivative of pO_2_ measurements [2]. In order to validate the method, we construct datasets with known ground truth. Estimates of CMRO_2_ from ground truth model data suggest that the Laplace method represents a more useful tool for measuring O_2_ consumption than the Krogh method [2].

**References**

1. Sakadzic S, Yaseen MA, Jaswal R, Roussakis E, Dale AM, Buxton RB, Vinogradov SA, Boas DA, Devor A: Two-photon microscopy measurement of cerebral metabolic rate of oxygen using periarteriolar oxygen concentration gradients. *Neurophotonics* 2016, **3(4):** 045005.

2. Sætra MJ: Estimation of metabolic oxygen consumption from optical measurements in cortex. *Master’s thesis, University of Oslo* 2016. [http://urn.nb.no/URN:NBN:no-54857]

3. Goldman D: Theoretical models of microvascular oxygen transport to tissue. *Microcirculation* 2008, **15(8):** 795–811.

## P69 Computing Brain Signals: Concurrent simulation of network activity, extracellular electric potentials and magnetic fields

### Espen Hagen^1^, Solveig Næss^2^, Torbjørn V. Ness^3^, Gaute T. Einevoll^1,3^

#### ^1^Department of Physics, University of Oslo, Oslo, 0316, Norway; ^2^Department of Informatics, University of Oslo, Oslo, 0316, Norway; ^3^Faculty of Science and Technology, Norwegian University of Life Sciences, Aas, 1433, Norway

##### **Correspondence:** Espen Hagen (espen.hagen@fys.uio.no)

*BMC Neuroscience* 2017, **18(Suppl 1)**:P69

Recordings of extracellular electrical, and later also magnetic, brain signals have been the dominant technique for measuring brain activity for almost a century. The interpretation of such signals is nontrivial [1–3], however, as the measured signals result of both local and remote neuronal activity. The recorded extracellular potentials in general stem from a complicated sum of contributions from transmembrane currents of neurons near the measurement site, while corresponding intra- and extracellular electric currents generate the brain’s magnetic field [4]. This calls for forward-models grounded in the biophysics of the different measurement modalities [3] while the underlying sources are faithfully represented. The initial release of the Python package LFPy ([5], LFPy.github.io) incorporated a now commonplace and well-established scheme for predicting extracellular potentials of individual neurons with arbitrary levels of biological detail. LFPy relies on the NEURON simulation environment ([6], neuron.yale.edu) to compute transmembrane currents of multicompartment neurons in conjunction with an electrostatic forward model [7]. We have now extended its functionality to populations and networks of multicompartment neurons with concurrent calculations of extracellular potentials and current-dipole moments [8]. The current-dipole moments are used to compute non-invasive measures of neuronal activity, e.g., electroencephalogram (EEG) scalp potentials when combined with an appropriate volume-conductor model. One such model is the 4-sphere model including the different electric conductivities of brain, cerebral spinal fluid, skull and scalp [9]. In addition, the current-dipole moments can be used for magnetoencephalography (MEG) signal prediction [4,9]. The version of LFPy presented here is thus a true multi-scale simulator, capable of simulating electric neuronal activity at the level of cell-membrane dynamics, individual synapses, neurons, networks, extracellular potentials within neuronal populations and macroscopic EEG and MEG signals. The present implementation is suitable for parallel execution on HPC facilities.

**Acknowledgements**

This work is supported by the Norwegian Ministry of Education and Research through the Research Council of Norway (NFR, through COBRA, CINPLA, NOTUR -NN4661 K) and SUURPh Programme, and EU Grant 604102 (HBP).

**References**

1. Pettersen, KH, Lindén, H, Dale, AM, Einevoll, GT: Extracellular spikes and CSD, *in Brette, R. and Destexhe, A. (eds.)* *Handbook of Neural Activity Measurement*. Cambridge: Cambridge University Press; 2012

2. Buzsaki G, Anastassiou CA, Koch K: The origin of extracellular fields and currents – EEG, ECoG, LFP and spikes*. Nat Rev Neurosci* 2012, **13:**407–419

3. Einevoll GT, Kayser C, Logothetis NK, Panzeri S: Modelling and analysis of local field potentials for studying the function of cortical functions. *Nat Rev Neurosci* 2013, **14:**770–785

4. Hämäläinen M, Hari R, Ilmoniemi RJ, Knuutila J, Lounasmaa OV: Magnetoencephalography—theory, instrumentation, and applications to noninvasive studies of the working human brain. Rev Mod Phys 1993, **65**:413–487

5. Lindén H, Hagen E, Leski S, Norheim E, Pettersen KH, Einevoll GT: LFPy: a tool for biophysical simulation of extracellular potentials generated by detailed model neurons. *Front Neuroinf* 2014, **7(41):**1–15

6. Hines ML, Davison AP, Muller E: NEURON and Python. *Front Neuroinf* 2009, **3(1):**1–12

7. Holt G, Koch C: Electrical Interactions via the Extracellular Potential Near Cell Bodies. *J Comp Neurosci* 1999, **6:**169–184

8. H Lindén H, Pettersen KH, Einevoll GT: Intrinsic dendritic filtering gives low-pass power spectra of local field potentials. *J Comp Neurosci* 2010, **29:**423–444

9. Nunez PL, Srinivasan R: *Electric Fields of the Brain: The neurophysics of EEG, 2* ^*nd*^ *edition*. Oxford: Oxford University Press; 2006

## P70 Integration of orientation and spatial frequency in a model of visual cortex

### Alina Schiffer^1^, Axel Grzymisch^1^, Malte Persike^2^, Udo Ernst^1^

#### ^1^Computational Neuroscience Lab, Institute for Theoretical Physics, Univ. of Bremen, Bremen, 28359, Germany; ^2^Department of Psychology, Methods Section, Johannes Gutenberg University Mainz, Mainz, 55122, Germany

##### **Correspondence:** Alina Schiffer (alina@neuro.uni-bremen.de)

*BMC Neuroscience* 2017, **18(Suppl 1)**:P70

In the visual system, complex scenes have to be integrated from simple local features into global and meaningful percepts. One basic process in feature integration that is needed to e.g. form the shape of objects is contour integration. Models studying this process usually focus on orientation alignment as the defining feature of a contour, however, experimental work has shown that also other features such as spatial frequency (SF) strongly shape contour integration. In our framework, we include SFs as a second cue to gain deeper insight into mechanisms of contour integration, by hypothesizing that similar SFs will be integrated more strongly than dissimilar ones.

We constructed a structurally simplistic cortical model with population dynamics described by simplified Wilson-Cowan equations. The model was presented with stimuli consisting of an ensemble of oriented Gabor patches with different orientations and spatial frequencies, into which contours of aligned and/or SF-homogeneous patches are embedded. Feature integration is performed by recurrent interactions between populations with receptive fields (RFs) tuned to the orientation and SF of localized stimulus patches. Interactions comprise excitatory and inhibitory couplings, with inhibition providing normalization and being independent on orientation preference. Excitatory connections realize an association field [1] specifying the linking strength for elements with different properties: In particular, we implement strong links between collinear and co-circularly aligned RFs, and we assume that interaction strength exponentially increases with decreasing SF difference (i.e., “what fires together wires together”).

By quantitatively reproducing the results of multiple psychophysical studies [2] we are able to provide a unifying account of contour integration in a variety of different stimulation paradigms. Our model suggests a novel mechanism involved in feature integration, namely spatial-frequency dependent interactions, which accounts for previously unexplained findings (see Figure 1), thus going beyond contour integration on orientation information only, and helping to create a more comprehensive understanding of computation in the visual system.
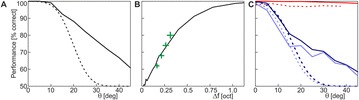


**Figure 1.** Comparison of model (solid lines) and experimental psychometric curves (dashed lines) for contour detection in a 2-AFC design. Since the model is not subject to noise, we expect its performance to be equal or higher than for human observers. **A**: Contour defined by orientation alignment only (same SF for all Gabors): performance decreases with increasing tilt angle deviating from perfect alignment. **B**: Contour defined by SF shift between contour and background elements (random orientations for all Gabors): performance increases with increasing SF shift (green crosses: experiment). **C**: Contour defined by orientation alignment, with SFs of contour and background subject to different levels of random jitter (2 octaves and 3 octaves width, light and dark blue, respectively): detection threshold decreases with increasing jitter. For jitter on the contour elements only (red), the target remains visible even for large tilt angles (prediction of model confirmed by new experiments, unpublished data)

**Acknowledgements**

This work was supported by the BMBF (Bernstein Award Udo Ernst, grant no. 01GQ1106). Alina Schiffer was supported by the SMART START 2 Program.

**References**

1. Field DJ, Hayes S, Hess RF: Contour integration by the human visual system: Evidence for a local association field. *Vision Res.* 1993, **33**:173–193.

2. Persike M, Meinhardt G: Cue combination anisotropies in contour integration: The role of lower spatial frequencies. *Journal of Vision* 2015a, **15(5)**:17; Persike M, Meinhardt G: Effects of spatial frequency similarity and dissimilarity on contour integration. *PLoS One* 2015b, **10(6):**1–19.

## P71 Performance-optimization guided distribution of attentional resources

### Daniel Harnack, Udo A Ernst^1^

#### Computational Neuroscience Lab, Institute for Theoretical Physics, University Bremen, Bremen, Germany

##### **Correspondence:** Daniel Harnack (daniel@neuro.uni-bremen.de)

*BMC Neuroscience* 2017, **18(Suppl 1)**:P71

In the visual system, attention improves information processing and is required to solve complex tasks such as shape detection and object recognition. On the neuronal level, it is found that different task demands, given by e.g. nature and specific combination of cues and cue validities, modulate response properties in many cortical areas in parallel [1]. Selective attention is assumed to be instrumental in orchestrating the flexible and efficient distribution of resources among brain areas to set up task-specific functional networks [2]. However, it is unclear how this process is organized on a functional level, and according to which principles computation is coordinated among different neuronal populations and visual areas. Here, we investigate task-specific attentional distribution in a simplified framework where a stimulus is processed by two or more neuronal populations (or visual areas) which are specialized in representing different features such as orientation or color. We assume the task is to detect a stimulus change in one of its features, while a cue is given that matches the changing feature with a certain probability (cue validity). Adhering to physiological constraints, attention is modeled as a bounded gain change on the populations’ outputs to a higher area decision population, whereas the whole input to the decision population is normalized [3] (**Fig. 1A**). Considering distributed attention as an optimization problem, we compute the gain factors minimizing error rates for the different populations engaged in the task by analytical gradient descent. We find that optimal gain factors depend on cue validity and change saliency, with attention also boosting the populations representing non-cued features if cue validity is below 100% and if change saliency is high. Furthermore, when attention spreads to non-cued features, we find that a multitude of attentional distributions exist that yield the same optimal performance (**Fig. 1B**). Our results have important implications for empirical studies: first, we provide a first-principle explanation in a minimal framework of attentional modulation spreading also to non-cued feature dimensions or attributes. Second, the dependence of optimal modulation strength on task parameters and the degenerative nature of solutions in part of the parameter space implies that attention-related gain changes observed in animal studies might not be constant, but will change over time if e.g. cue validity is manipulated and if perceptual learning takes place.
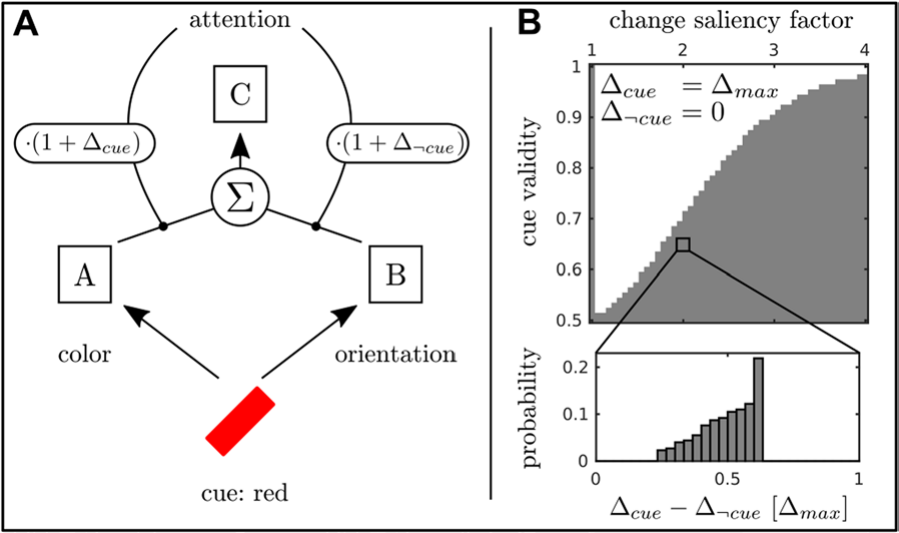


**Figure 1. A:** Schematic of the model setup with a two-feature stimulus. **B:** In the white region, optimal performance is achieved by directing maximal attention towards the cued feature and none to the non-cued one. In the gray region, it is optimal to also attend to the non-cued feature. Here, solutions are degenerate such that a multitude of attentional configurations leads to the same optimal performance. The probability distribution of modulation differences illustrates this for one exemplary parameter set

**Acknowledgements**

This research was funded by the BMBF (Bernstein Award Udo Ernst, Grant 01GQ1106).

**References**

1. Siegel M, Buschmank TJ, Miller EK: Cortical information flow during flexible sensorimotor decisions. *Science* 2015, **348:**1352–1355.

2. Harnack D, Ernst UA, Pawelzik KR: A model for attentional information routing through coherence predicts biased competition and multistable perception. *J Neurophysiol* 2015, **114:**1593–1605.

3. Reynolds JH, Heeger DJ: The normalization model of attention. *Neuron* 2009, **61:**168–185.

## P72 Feature integration with critical dynamics in cortical subnetworks

### Nergis Tomen, Udo Ernst

#### Computational Neuroscience Lab, Institute for Theoretical Physics, University of Bremen, 28359, Bremen, Germany

##### **Correspondence:** Nergis Tomen (nergis@neuro.uni-bremen.de)

*BMC Neuroscience* 2017, **18(Suppl 1)**:P72

Recent experimental and theoretical work increasingly suggests that cortical neurons operate close to a critical state which describes a phase transition from chaotic to ordered dynamics and optimizes multiple aspects of information processing (e.g. [1,2]). However, although critical dynamics have been demonstrated in recordings of spontaneously active cortical neurons [3], the link between criticality and active cortical computation remains largely unexplored. Establishing this link requires addressing major conceptual challenges, namely: making abstract complexity measures work in realistic computational settings and considering—instead of homogeneous, spontaneously active networks—strongly driven systems with high firing rates and networks with structured connectivity.

In our work, we focus on visual feature integration as a prototypical and prominent example for cortical computation. Visual feature integration refers to neural processes which link localized image information into more global representations such as contours, shapes, and objects. We study feature integration in a figure-ground segregation task, where cortical subnetworks operate close to the critical state when part of a visual stimulus matches a ‘figure’ which is to be detected by the visual system. Within the simple, but analytically well-described framework of the Ernst-Herrmann-Eurich (EHE) model, we embed a large number of figures into a recurrently coupled network. Out of *N* units representing each figure, we allow for *n* units to represent multiple figures at the same time and characterize the network dynamics for different stimuli.

We find that presenting a visual stimulus with a target figure dynamically organizes the network into two parts: one with critical dynamics, encoding the ensemble of features making up the figure, and one with subcritical dynamics, encoding the background elements. We show that figure representation in the oscillatory dynamics of the system as well as the task performance in a 2AFC-scenario is maximized near the critical point. Adding inhibitory interactions between neurons encoding different figures ensures that the coupling strength for which the network is critical is robust against changes in *n* (Figure 1), the network size and the number of figures in the network.

Our model extends the idea of criticality being optimal for computation to inhomogeneous systems, establishes links to spatial computation performed in the visual system and predicts that local subnetworks can display supercritical activity, contained by inhibition, while the cortex at large is poised at subcritical regimes.
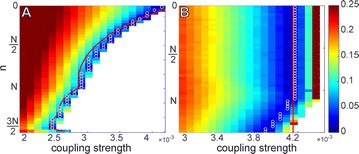


**Figure 1.** The Kolmogorov–Smirnov (KS) statistic, quantifying the distance of spike statistics from a power-law, for an excitatory network **(A)** and for a network with both excitation and inhibition **(B)**, as a function of the coupling strength and the overlap between figures, *n*. In the white regions, the network starts to exhibit infinite avalanches. White circles mark where the KS statistic is lowest and the red line shows our theoretical approximation for the critical point. We find that for the network with inhibition, the critical coupling strength does not change as *n* increases until *n* = *N*

**Acknowledgements**

This research project was funded by the BMBF (Bernstein Award Udo Ernst, grant no. 01GQ1106).

**References**

1. Shew WL, Yang H, Petermann T, Roy R, Plenz D: Neuronal avalanches imply maximum dynamic range in cortical networks at criticality. *J Neurosci* 2009, **29:**15595–15600.

2. Tomen N, Rotermund D, Ernst U: Marginally subcritical dynamics explain enhanced stimulus discriminability under attention. *Front Syst Neurosci* 2014, **8:**151.

3. Beggs JM, Plenz D: Neuronal avalanches in neocortical circuits. *J Neurosci* 2003, **23:**11167–11177.

## P73 Interneuronal contribution to state transition in the mouse neocortex

### Stefano Zucca^1,2^, Valentina Pasquale^3^, Giuseppe Pica^2,4^, Manuel Molano-Mazón^2,4^, Michela Chiappalone^3^, Stefano Panzeri^2,4^, Tommaso Fellin^1,2^

#### ^1^Optical Approaches to Brain Function Laboratory, Department of Neuroscience and Brain Technologies, Istituto Italiano di Tecnologia, Genova, Italy; ^2^Neural Coding Laboratory, Istituto Italiano di Tecnologia, Genova and Rovereto, Italy; ^3^Department of Neuroscience and Brain Technologies, Istituto Italiano di Tecnologia, Genova, Italy; ^4^Neural Computation Laboratory, Center for Neuroscience and Cognitive Systems @UniTn, Istituto Italiano di Tecnologia, Rovereto, Italy

##### **Correspondence:** Valentina Pasquale (valentina.pasquale@iit.it)

*BMC Neuroscience* 2017, **18(Suppl 1)**:P73

Large scale transitions between active (up) and silent (down) states during NREM sleep or quiet wakefulness regulate fundamental cortical functions and are known to involve both excitatory and inhibitory cells. However, the role of inhibition in regulating cortical state transitions is still unclear and controversial.

Using simultaneous local field potential (LFP) and two-photon-guided juxtasomal in vivo recordings of parvalbumin (PV) and somatostatin (SST) positive cells in anesthetized mice, we investigated the relationship between the firing activity of interneurons and the up-down state cycle. Up/down state time periods were detected from the LFP signal by means of advanced analytical methods, modified from the literature [1, 2] and calibrated for our experimental conditions based on previous simultaneous LFP and patch-clamp recordings on pyramidal neurons. We first measured the firing activity of PV and SST interneurons in the two different states. We confirmed that both cell types are mainly active during the up state, but we also found that they display significant firing activity during cortical silent state. To investigate a potential correlation between the occurrence of spikes in PV/SST interneurons and the LFP slow-oscillation phase (which in turn reflects cortical state [2]), we quantified the phase locking of interneuronal spikes for different relative time shifts of LFP and juxtasomal recordings [3, 4]. Phase locking was found to be maximal when shifting LFP backwards by ~100 ms (and up to ~200 ms) for both classes of interneurons, suggesting a causal contribution of interneurons for changes in phase dynamics, in particular in the latter part of the up state (including its end). This result was further confirmed by analyzing the mean changes in the LFP phase speed triggered by a spike prior to the end of an up/down state: we found that, on average, phase speed significantly increased after a PV spike and a SST spike near the end of an up state. In contrast, phase speed significantly decreased after a PV spike near the end of a down state, while no significant effect was found for SST neurons in the down state.

Together these findings support the hypothesis that the firing of both PV and SST interneurons during up states may causally contribute to their ending, whereas the firing of interneurons during down states, at least for PV cells, may delay the transition from the down state to the up state.

**Acknowledgements**

This work was supported by the ERC (NEURO-PATTERNS), NIH (1U01NS090576-01), FP7 (DESIRE), MIUR FIRB (RBAP11X42L) to TF, and, in part, from Flag-Era JTC Human Brain Project (SLOW-DYN) to TF and SP. MMM is supported by a Marie Skłodowska-Curie fellowship (ETIC, 6999829).

**References**

1. Mukovski M, Chauvette S, Timofeev I, Volgushev M: Detection of active and silent states in neocortical neurons from the field potential signal during slow-wave sleep. *Cerebral cortex* 2007, **17**(2):400–414.

2. Saleem AB, Chadderton P, Apergis-Schoute J, Harris KD, Schultz SR: Methods for predicting cortical UP and DOWN states from the phase of deep layer local field potentials. *Journal of computational neuroscience* 2010, **29**(1–2):49–62.

3. Siapas AG, Lubenov EV, Wilson MA: Prefrontal phase locking to hippocampal theta oscillations. *Neuron* 2005, **46**(1):141–151.

4. Eschenko O, Magri C, Panzeri S, Sara SJ: Noradrenergic neurons of the locus coeruleus are phase locked to cortical up-down states during sleep. *Cerebral cortex* 2012, **22**(2):426–435.

## P74 Embodiment, connectivity, and critical states in neural systems

### Kelvin S. Oie^1^, David L. Boothe^1^, Joshua C. Crone^1^, Alfred B. Yu^1^, Melvin A. Felton, Jr.^2^

#### ^1^U.S. Army Research Laboratory, Aberdeen Proving Ground, MD, 21005, USA; ^2^U.S. Army Research Laboratory, Adelphi, MD, 20783, USA

##### **Correspondence:** Kelvin S. Oie (kelvin.s.oie.civ@mail.mil)

*BMC Neuroscience* 2017, **18(Suppl 1)**:P74

The spontaneous emergence of collective dynamical behaviors in complex networks has been the focus of intensive study in physical and biological systems. Such collective dynamics in the form of neural oscillations are widely thought to underlie information processing in the brain. However, while many theoretical studies of collective dynamics have utilized homogenous networks with simple topologies (e.g., global couplings or regular lattices), biological neural systems are characterized by heterogeneity and complex patterns of connectivity. Specifically, the biological brain belongs to a class of large-N systems with non-uniform connectivity, where the connectivity between neurons is of degree $K \ll N$ (i.e., sparse). Recently, it has been demonstrated that a critical – though typically low – degree of connectivity is necessary for the emergence of collective dynamics in such “strongly diluted” systems [1]. Previously, we have obtained results consistent with this finding, showing that the spontaneous emergence of large-amplitude oscillatory dynamics in a large-scale simulation model [2], which is composed of multi-compartment model neurons representing 11 distinct cell types, is dependent upon the degree of connectivity.

Here, we integrate some of our recent simulation modeling results (see further, submissions by Boothe et al. and Felton et al., at this meeting) with theoretical insights that have reported the coexistence of qualitatively different dynamics – irregular and regular – in large randomly connected networks [3] and the occurrence of unstable attractors in networks of pulse-coupled oscillators [4]. In such systems, it can be suggested that switching between dynamic regimes may provide a basis for computation. However, a recognition of the embodied nature of real-world systems, wherein a system’s collective dynamics are dependent upon external inputs and feedback processes, allows a reinterpretation of the functional role of collective dynamical behaviors in complex networks. Such a reinterpretation leads us to a novel proposal on the relationship between connectivity and “critical states” (i.e., system states whose occurrence leads to global system instabilities) in large-scale neural systems.

**Acknowledgements**

The authors would like to thank Dr. Piotr Franaszczuk for his insight and guidance provided over many constructive discussions. The views and conclusions contained in this document are those of the authors and should not be interpreted as representing official policies, either expressed or implied, of the Army Research Laboratory or the U.S. Government. The U.S. Government is authorized to reproduce and distribute reprints for Government purposes notwithstanding any copyright notation herein.

**References**

1. S. Luccioli, S. Olmi, A. Politi and A. Torcini, Collective dynamics in sparse networks, *Phys Rev Lett* 2012, **109(13)**:138103-1–138103-5.

2. D. L. Boothe, A. B. Yu, J. C. Crone and K. S. Oie, The impact of differential local connectivity on rhythmogenesis in a large scale model of cerebral cortex, 38th Int. Conf. *IEEE Eng Med Biol Soc*, Orlando, FL, 2016.

3. M. Timme, F. Wolf and T. Geisel, Coexistence of regular and irregular dynamics in complex networks of pulse-coupled oscillators, *Phys Rev Lett* 2002, **89(25)**:258701-1–258701-4.

4. M. Timme, F. Wolf and T. Geisel, Prevalence of unstable attractors in networks of pulse-coupled oscillators, *Phys Rev Lett* 2002, **89(15)**:154105-1–154105-4.

## P75 A computational model of temporal processing in the human auditory cortex

### Isma Zulfiqar^1^, Michelle Moerel^1,2^, Peter De Weerd^1,2^, Elia Formisano^1,2^

#### ^1^Maastricht Centre for Systems Biology, Maastricht University, Maastricht, 6229 ER, The Netherlands; ^2^Department of Cognitive Neuroscience, Maastricht University, Maastricht, 6229 ER, The Netherlands

##### **Correspondence:** Isma Zulfiqar (isma.zulfiqar@maastrichtuniversity.nl)

*BMC Neuroscience* 2017, **18(Suppl 1)**:P75

Temporal information in sounds is relevant for many general purpose and domain specific auditory functions, including amplitude modulation (AM) detection, extraction of temporal pitch and decoding of speech. Each of these functions has been extensively studied with a variety of methods (electrophysiology, non-invasive neuroimaging and psychophysics). The common neural coding mechanisms underlying these observations, however, remain unclear. Here we propose a computational framework to integrate available empirical information to derive a unified view of temporal information processing in the human auditory cortex (AC).

We designed a simplified model of the AC consisting of four functional units. These units approximately correspond to two core areas (AI, R) and two belt “streams” (Fast, Slow). Each unit was simulated using the Wilson Cowan Cortical Model (WCCM) [1] of neural circuitry. The WCCM generates dynamic neural responses by interaction of excitatory (E) and inhibitory (I) populations. AI and R receive cochleotopic thalamic input, the belt streams receive cochleotopic input from AI (Fast) and R (Slow) respectively. We adjusted the two main unit parameters, temporal and spectral integration windows, based on physiological evidence from monkeys [2,3] and recent human fMRI [4].

The model was tested on various artificial sounds to evaluate its representation of three psychoacoustic phenomena. Model output was evaluated by comparing the simulation results to human behavioral (psychoacoustic) studies.

First, we explored pure tone frequency discrimination. We observed a dual code for frequency discrimination. Model performance, in line with psychoacoustic results [5], deteriorated for frequencies above 2 kHz. This deterioration in discrimination could not be solely accounted for by a tonotopic place code, and instead depended on the temporal response of the model (specifically in “Fast” region). This observation demonstrates how a dual code (place and time) may underlie human frequency discrimination performance [5]. Second, we explored AM detection for both noise and tones (0.125–8 kHz). In line with experimental findings, AM was coded with two mechanisms (synchronization and rate coding). We observed a switch from a temporal to a rate code for modulation rates above 50 Hz. The upper limit of the temporal (but not rate) code was unit dependent, in accordance with electrophysiology [2]. Interestingly, estimated modulation transfer functions followed psychophysical modulation detection thresholds [5]. Furthermore, we observed a dependence of AM coding on the carriers in agreement with psychophysics [5]. Finally, we tested the model’s representation of temporal pitch with missing fundamental (MF) stimuli and iterated ripple noise (IRN). We again observed a dual coding strategy, where AI and the Fast area temporally decoded low (<300 Hz) MF sounds, while the Slow area represented high frequencies as phase coherence across the network. IRN was coded temporally with a comparatively weaker synchrony strength, matching its weaker pitch percept.

In summary, using a simple network of E and I interactions and tuning of only two parameters, we modeled processing of temporal information in parallel cortical streams. The read-out of population responses are in agreement with human psychoacoustics. In future work, we plan to explore the temporal processing of speech by this model.

**References**

1. Wilson HR, Cowan JD: Excitatory and inhibitory interactions in localized populations of model neurons. *Biophys Journal* 1972, **12:** 1–24.

2. Steinschneider M, Arezzo J, Vaughan HG Jr: Phase-locked cortical responses to a human speech sound and low-frequency tones in the monkey. *Brain Research* 1980, **198:** 75–84.

3. Bendor D, Wang X: Neural response properties of primary, rostral, and rostrotemporal core fields in the auditory cortex of marmoset monkeys. *J. Neurophysiol.* **100:** 888–906

4. Santoro R, Moerel M, De Martino F, Goebel R, Ugurbil K, Yacoub E, Formisano E: Encoding of Natural Sounds at Multiple Spectral and Temporal Resolutions in the Human Auditory Cortex. *PLoS Comput Biol* 2014, **10(1)**: e1003412. doi:10.1371/journal.pcbi.1003412.

5. Moore BCJ: An Introduction to the Psychology of Hearing 2012, *Brill*, Sixth Edition

6. Bendor D, Osmanski MS, Wang X: Dual-pitch processing mechanisms in primate auditory cortex. *J Neurosci.* 2012, **32(46):** 16149–1616.

## P76 The dependence of simulated local field potential (LFP) frequency content on local and long range connectivity

### David L. Boothe^1^, Alfred B. Yu^1^, Joshua C. Crone^1^, Melvin A. Felton Jr.^1^, Kelvin Oie^1^, Piotr Franaszczuk^1,2^

#### ^1^US Army Research Laboratory, Aberdeen, Maryland, 21005, USA; ^2^Department of Neurology, The Johns Hopkins University School of Medicine, Baltimore, Maryland, 21287, USA

##### **Correspondence:** David L. Boothe (david.l.boothe7.civ@mail.mil)

*BMC Neuroscience* 2017, **18(Suppl 1)**:P76

Large scale cortical oscillations recorded using techniques, such as electroencephalogram (EEG) are thought to provide a biological substrate for computation [1]. The relationship between cortical connectivity features (distance, myelination, neuronal arborization, and synapse location) and cortical oscillatory frequency is at present poorly understood [2]. In order to explore the impact of the full range of cortical connectivity features on network activity one needs to use a model with sufficient biophysical detail. Here, we present a compartment-level model of cortex and observe connectivity-dependent changes in network activity in the power spectrum of a simulated local field potential (LFP). Our model consists of 5,376 neurons, with 11 types of cortical and thalamic neurons each with its own compartmental morphology, connectivity patterns, and internal dynamics [3]. Activity is generated by a combination of intrinsic voltage fluctuations and extrinsic Poisson distributed noise (10% of neurons, mean 5 Hz). Neurons were divided into four micro-columns, which were then either left unconnected (LFP-Local) or connected via long range connections emerging from pyramidal cells in layers 2/3 (P23) and synapsing onto neurons in adjacent micro-columns. The number of P23 synapses were scaled to be equal across simulations. Our results indicate that long range connections contribute to the generation of sharp peaks in the power spectrum of the LFP at frequency ranges between 10 Hz and 50 Hz (Fig. 1B, red arrows). Simulations with local connectivity only generate activity with no clear peaks in the 1 to 50 Hz frequency range (Fig. 1A). Activity in some neuron types is altered by network connectivity. For instance, the power spectrums of the summed membrane potentials of layer 5 pyramidal cells (P5) in simulations with only short range connections match the overall LFP (Fig. 1A and C), while in simulations with long range connections P5 neurons contribute to peaks in the 10 to 50 Hz range (Fig. 1D). Our results indicate that long range connections contribute to increased oscillatory activity in network activity, and that this increase occurs in specific LFP frequency ranges. Future work will include exploring asymmetric long range connectivity and comparing cortical regions in different network states.
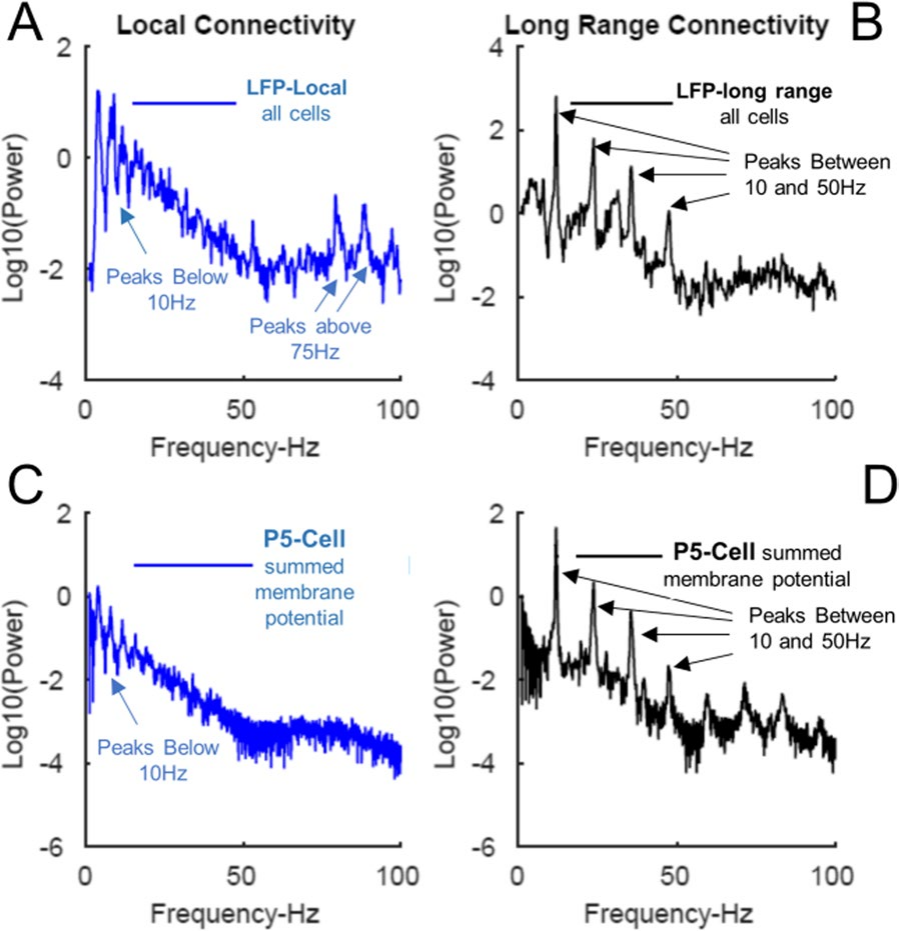


**Figure 1. A.** LFP power spectrum from model with no long range connections exhibits no strong peaks. **B.** LFP power spectrum of simulation with long range connections, exhibits clear peaks. **C** and **D**. contribution of pyramidal cells to LFP depend on long range connections

**References**

1. VanRullen R, & Koch C: Is perception discrete or continuous? *Trends in Cognitive Sciences* 2003, **7(5)**:207–213.

2. Passingham RE, Stephan KE, Kotter R: The anatomical basis of functional localization in the cortex. *Nature Reviews Neuroscience* 2002, **3**:606–616.

3. Traub RD, Contreras D, Cunningham MO, Murray H, LeBeau FE, Roopun A,Whittington MA: Single-column thalamocortical network model exhibiting gamma oscillations, sleep spindles, and epileptogenic bursts. *Journal of Neurophysiology*, 2005, ***93*** **(4)**: 2194–2232.

## P77 Pre-whitening as a means to improve dimensionality reduction and simplify clustering in spike-sorters for multi-electrode recordings

### Roland Diggelmann^1,2^, Michele Fiscella^1,2^, Andreas Hierlemann^1^, Felix Franke^1^

#### ^1^Department of Biosystems Science and Engineering, ETH Zurich, Basel, Switzerland; ^2^Neural Circuit Laboratories, Friedrich Miescher Institute for Biomedical Research, Basel, Switzerland

##### **Correspondence:** Roland Diggelmann (roland.diggelmann@bsse.ethz.ch)

*BMC Neuroscience* 2017, **18(Suppl 1)**:P77

Spike sorting is the process to extract single neuronal activity from extracellular recordings. It makes use of the fact that spikes from a single neuron feature highly similar waveforms, whereas spikes from different neurons have different waveforms. Clustering algorithms are used to find groups of similar spikes that putatively originated from the same neuron. However, since spike waveforms especially in multi-electrode recordings can have a high dimensionality, their dimensionality needs to be reduced before clustering. Principal component analysis (PCA) is one of the most commonly employed dimensionality reduction methods for this purpose [1]. It reduces the dimensions to those where the variance of the data was highest, presumably those along which the waveforms of separate neurons differ most strongly, However, if the noise is not uniform in all dimensions, high variability can also mean high noise, which would render a dimension useless for discrimination. We, therefore, propose an additional pre-whitening step before PCA and discuss two beneficial effects on the subsequent clustering. We illustrate these effects by using spikes from retinal ganglion cells recorded with high-density multi-electrode arrays (HD-MEA).

Pre-whitening is a method that is used to transform a set of data points that follow a multivariate Gaussian distribution with a given covariance matrix to make them follow a multivariate standard normal distribution after transformation. In the case of our HD-MEA recordings, the noise covariance is a strong contributor to non-spherical shapes of spike clusters, which means that pre-whitening significantly affects the cluster shapes.

The first beneficial effect of pre-whitening for spike sorting is that it leads to a better alignment of the first principal components with the directions that feature the largest between-cluster variance. The reason for this is that the noise is uniformly distributed over all dimensions after pre-whitening. Therefore, the cluster locations are now the main contributors to the variance in the data.

The second benefit of pre-whitening is that it simplifies the clustering problem. After pre-whitening, the clusters are roughly spherical with uniform standard deviation in all dimensions. (There is one caveat to this: Since the noise covariance matrix used for pre-whitening does not capture all the variability of the spike waveforms, the clusters are in fact not completely spherical. But we will show that the benefits of the procedure are still given). Knowing the approximate size and shape of the clusters, we can choose suitable clustering procedures, which offer the possibility to employ unsupervised learning techniques that do not require subsequent manual curation of the clusters.

In conclusion, pre-whitening is a simple method to improve PCA-based dimensionality reduction of multi-electrode array recordings and it simplifies the clustering problem for the spike-sorting algorithms. Thus, we believe pre-whitening before dimensionality reduction is an important tool towards the development of fully automatic spike-sorters.

**Acknowledgements**

Financial support through the ERC Advanced Grants 267351 “NeuroCMOS” (FP7) and 694829 “neuroXscales” (H2020) and the Swiss National Science Foundation Sinergia Project CRSII3_141801 are acknowledged, as well as individual support for R. Diggelmann through a Swiss SystemsX IPhD grant.

We thank Thomas L. Russell for sharing tissue material from his experiments.

**References**

1. Lewicki MS. A review of methods for spike sorting: the detection and classification of neural action potentials. *Netw. Comput. Neural Syst*. 1998; **9:**R53–78.

## P78 An integrative model explaining many functions of corticothalamic feedback

### Domenico Guarino^1^, Jan Antolík^1,2^, Andrew P. Davison^1^, Yves Frègnac^1^

#### ^1^UNIC, CNRS-FRE3693, Gif-sur-Yvette, 91190, France; ^2^Institut de la Vision, Paris, 75012, France

##### **Correspondence:** Domenico Guarino (domenico.guarino@unic.cnrs-gif.fr)

*BMC Neuroscience* 2017, **18(Suppl 1)**:P78

In the early visual system of the cat, the feedforward pathway going from the lateral geniculate nucleus (LGN) to the primary visual cortex (V1) is well characterized both anatomically and functionally. On the other hand, despite a large amount of experimental work, there is poor agreement about functional roles of the feedback pathway from V1 to LGN. The common experimental approach compares system responses taken in the *open*-*loop* condition - probing thalamus in isolation from cortex - and in the *closed*-*loop* condition - probing the intact system. In the literature to date this approach has yielded inconsistent results. Open- and closed-loop results for some stimuli differ only by an additive factor, while for other stimuli there is a marked qualitative difference in the results.

In this computational study, we investigated the thalamocortical loop with reference to an unprecedentedly broad set of experimental studies. We took published data for six types of experiments (with stimuli varying in luminance, contrast, spatial frequency, temporal frequency, size, and orientation) involving both open- and closed-loop conditions. We explored each condition with an integrative large-scale spiking model of the cat early visual system that includes LGN, peri-geniculate nucleus (PGN), and V1. The model is heavily constrained by the available literature at multiple levels: anatomical, functional, and statistical. The model is developed using the Mozaik workflow engine [1] with NEST [2] as the backend simulator.

The experimental constraints enabled us to find a single parameter set that qualitatively and quantitatively accounts for the published responses to all stimuli in both open- and closed-loop conditions. We report that in our model the feedback has additive effects on: spontaneous activity, in agreement with [3], contrast tuning response [4], and temporal frequency tuning [5]. On the other hand, in our model the feedback changes the shape of responses for: spatial frequency tuning, as in [6], size tuning response [7], and orientation tuning [8].

We identify two main mechanisms supporting our results: the reciprocal spatial distribution of cortical and thalamic receptive fields, as in [9], and the topological mirroring of cortical response profiles onto thalamus, as in [10].

**References**

1. Antolík J, Davison AP: Integrated workflows for spiking neuronal network simulations. *Front Neuroinform* 2013, **7**:34

2. Gewaltig MO, Diesmann M: NEST (Neural Simulation Tool). *Scholarpedia* 2007, **2**(4):1430.

3. Waleszczyk WJ, Bekisz M, Wróbel A: Cortical modulation of neuronal activity in the cat’s lateral geniculate and perigeniculate nuclei. *Exp Neurol* 2005, **196**:54–72.

4. Li G, Ye X, Song T, Yang Y, Zhou Y: Contrast adaptation in cat lateral geniculate nucleus and influence of corticothalamic feedback. *EJ Neurosci* 2011 **34**:622–631

5. Marrocco RT, McClurkin JW, Alkire MT: The influence of the visual cortex on the spatiotemporal response properties of lateral geniculate nucleus cells. *Brain Res* 1996, **737**:110–118.

6. Kimura A, Shimegi S, Hara SI, Okamoto M, Sato H: Role of GABAergic inhibition in shaping the spatial frequency tuning of neurons and its contrast dependency in the dorsal lateral geniculate nucleus of cat. *EJ Neurosci* 2013, **37**:1270–83.

7. Murphy PC, Sillito AM: Corticofugal feedback influences the generation of length tuning in the visual pathway. *Nature* 1987, **329**:727–729.

8. Vidyasagar TR, Urbas JV: Orientation sensitivity of cat LGN neurones with and without inputs from visual cortical areas 17 and 18. *Exp Brain Res* 1982, **46**:157–169.

9. Jones HE, Andolina IM, Ahmed B, Shipp SD, Clements JT, Grieve KL, Cudeiro J, Salt TE, Sillito AM: Differential feedback modulation of center and surround mechanisms in parvocellular cells in the visual thalamus. J Neurosci 2012 **32**:15946–15951.

10. Suga N, Yan J, Zhang Y: Cortical maps for hearing and egocentric selection for self-organization. *TICS* 1997, **1**:13–20.

## P79 State-Dependent Control of Oscillatory Brain Dynamics

### Benjamin Xavier Etienne^1^, Flavio Frohlich^2^, Jérémie Lefebvre^1,3^

#### ^1^Krembil Research Institute, University Health Network, Toronto, Ontario M5T 2S8, Canada; ^2^Department of Psychiatry and Cell Biology, University of North Carolina at Chapel Hill, Chapel Hill, North Carolina, USA; ^3^Department of Mathematics, University of Toronto, Toronto, Ontario, M5S 3G3, Canada

##### **Correspondence:** Jérémie Lefebvre (jeremie.lefebvre@uhnresearch.com)

*BMC Neuroscience* 2017, **18(Suppl 1)**:P79

Numerous studies have shown that periodic electrical stimulation can be used to not only to interfere with the activity of isolated neurons, but also to engage population-scale synchrony and collective rhythms. These findings have raised the fascinating prospect of manipulating emergent brain oscillations in a controlled manner, engaging neural circuits at a functional level to boost information processing, manipulate cognition and treat neurobiological disorders (“oscillopathies”) [1]. Capitalizing on this, it has been shown that brain stimulation can be tuned to alter perception and task performance [2]. Rhythmic brain stimulation forms the basis of a control paradigm in which one can manipulate the intrinsic oscillatory properties of cortical networks via a plurality of input-driven mechanisms such as resonance, entrainment and non-linear acceleration [3]. **But the brain is not a passive receiver**: outcomes of brain stimulation, either intracranial or non-invasive, are highly sensitive to ongoing brain dynamics, interfering and combining with internal fluctuations in non-trivial ways [4]. Exogenous control on brain dynamics has indeed been shown to be gated by neural excitability, where effects of brain stimulation are both state-dependent and highly sensitive to stimulation parameters [5].

To understand this phenomenon, we here used computational approach to study the role of ongoing dynamics on the entrainment of cortical neurons. We here considered the thalamo-cortical system as a non-linear oscillator and examined its resonant properties in different noise-induced activation states. Specifically, we examined whether state-dependent changes in thalamo-cortical oscillations – mediated by increased input to the thalamus - could implement a gain control mechanism regulating cortical susceptibility to stimulation. Our analysis shows that this increased sensory drive is sufficient to trigger the suppression of resting state oscillations throughout the system, resulting in a gradual transition from strong pairwise correlations towards asynchronous neural firing. We found that this increase in irregular fluctuations during task states enables a greater susceptibility of cortical neurons to entrainment, and that this phenomenon can explained by a passage through a bifurcation combined to stochastic resonance. These results suggest that the stability of resting state attractor dynamics limits the controllability of neural systems. To better understand the mechanism involved, we used a conceptual delayed oscillator model and computed its resonance curves both close and away from the a Hopf bifurcation in presence of noise [6]. The analysis reveals non-linear interactions between internal resonances, noise and externally applied periodic stimulation, deepening our understanding of the mechanism regulating the susceptibility of neural systems to entrainment.

Taken together, our results provide new insights about the state-dependent interaction between rhythmic stimulation and cortical oscillatory activity, accelerating the development of new paradigms to interrogate neural circuits and restore cognitive functions based on the selective manipulation of brain rhythms.

**References**

1. Romei et al.: Information-Based Approaches of Noninvasive Transcranial Brain Stimulation. *Trends Neurosci.* 2016, **39(11):**782–795.

2. Cecere et al.: Individual Differences in Alpha Frequency Drive Crossmodal Illusory Perception. *Curr Biol* 2015, **19(2):**231–235.

3. Herrmann et al.: Shaping Intrinsic Neural Oscillations with Periodic Stimulation*. J Neurosci.* 2016, **36:**5328–37.

4. Neuling et al.: Orchestrating neuronal networks: sustained after-effects of transcranial alternating current stimulation depend upon brain states. *Front. Hum. Neurosci* 2013, **7:**161.

5. Alagapan et al.: Modulation of cortical oscillations by low-frequency direct cortical stimulation is state-dependent. *PLOS Biology* 2016 **14**: e1002424.

6. Hutt et al.: Dynamic Control of Synchronous Activity in Networks of Spiking Neurons. *PLOS One* 2016, **11(9):** e0161488.

## P80 Switch of preference as a signature of heterogeneous excitability of neurons in the primate prefrontal cortex

### Encarni Marcos^1^, Maurizio Mattia^2^, Aldo Genovesio^1^

#### ^1^Department of Physiology and Pharmacology, Sapienza University of Rome, Rome, Italy; ^2^Istituto Superiore di Sanità, Rome, Italy

##### **Correspondence:** Encarni Marcos (encarni.marcos@uniroma1.it)

*BMC Neuroscience* 2017, **18(Suppl 1)**:P80

We continuously need to coordinate multiple processes to accomplish our current goals. By means of its connection with multiple other areas of the brain, the prefrontal cortex (PF) plays a pivotal role in this cognitive challenge [1]. Neurons in PF are known to represent task-relevant information and to memorize current goals until the proper action can be selected. However, how PF neurons transform the goals into specific actions is not completely understood yet. To address this question, we used an experimental task that resembles a situation in which an object needs to be maintained in memory and then perform a motor plan to reach it.

Two monkeys were trained to perform a distance discrimination task [2] while the activity of neurons in PF were recorded. All procedures followed the Guide for the Care and Use of Laboratory Animals (1996, SBN 0-309-05377-3) and were approved by the NIMH Animal Care and Use Committee. The monkeys had to decide which of two stimuli (blue or red) sequentially presented on a screen was farther from a reference point. A working memory period separated the end of the presentation of the second stimulus from the reappearance of both stimuli (goals) that served as a “goal” signal. The positions of the two goals were randomized so that, during the working memory period, the monkeys could not predict the future action to perform. Thus, during the delay period the monkeys had to remember the goal (blue or red) and then to select it by touching the corresponding switch below. We found that only a minority of neurons were involved in both the encoding of the goal in memory and the transformation of it into an action by representing the goal also in this phase. Moreover, in equal proportion, they switched or maintained their goal preference across such transition. Such high probability in the change of preference did not occur in other periods of the task and therefore we interpreted it as a signature of an activity reconfiguration of the PF network due to a transition between different collective states.

From a theoretical point of view, the active maintenance of goal information in memory requires some degree of stability whereas, by contrast, the same network needs to be susceptible and flexible enough to adapt to the external changes. To account for such dynamics, we propose that the PF network is composed of bistable cell assemblies with heterogeneous excitability [3] such that both dynamical stability and input susceptibility can be simultaneous expressed. Moreover, we show that, although the neurons that represent the goal both in memory and during the goal to action transformation process are only a minority of all neurons, they can play a fundamental role in the PF activity reconfiguration.

**References**

1. Desimone R, Duncan J. Neural mechanisms of selective visual attention. *Annu Rev Neurosci* 1995, **18:** 193–222.

2. Genovesio A, Tsujimoto S, Wise SP. Prefrontal cortex activity during the discrimination of relative distance. *J Neurosci* 2011, **31**:3968–3980.

3. Mattia M, Pani P, Mirabella G, Costa S, Del Giudice P, Ferraina S. Heterogeneous attractor cell assemblies for motor planning in premotor cortex. *J. Neurosci.* 2013, **33**: 11155–11168.

## P81 Modeling of the perceptual dynamics of the perception of body motion

### Leonid A. Fedorov^1,2^, Tjeerd M.H. Dijkstra^1^, Louisa Sting^1,3^, Howard Hock^4^, Martin A Giese^1,2^

#### ^1^Section for Comput. Sensomotorics, Department Cognitive Neurology, CIN&HIH, Univ. of Tübingen, Tübingen, Germany; ^2^GTC, International Max Planck Research School, University of Tübingen, Tübingen, Germany; ^3^Department of Cognitive Science, University of Tübingen, Tübingen, Germany; ^4^Center for Complex Systems and the Brain Sciences, Department of Psychology, Florida Atlantic University, Boca Raton, FL, USA

##### **Correspondence:** Leonid A. Fedorov (leonid.fedorov@uni-tuebingen.de)

*BMC Neuroscience* 2017, **18(Suppl 1)**:P81

Dynamic phenomena of perceptual organization and multi-stable perception have been studied extensively since the time of Gestalt psychology, typically with low-level vision. Recent work demonstrates multi-stability and adaptation for high-level body motion perception. We have developed a neurodynamical model that reproduces multi-stability and adaptation in body motion perception. Our model consists of hierarchies of neural detectors that analyze the silhouette and the shading features of body motion stimuli, which are encoded as temporal sequences of patterns by a dynamic neural field (Fig 1A). Its multi-stable dynamics accounts for spontaneous perceptual switching. In addition, its neurons are adaptive, accounting for high-level after-effects. Further details of the implementation are discussed in [2].We showed elsewhere that the model reproduces the perceptual multi-stability of body motion perception and its dependence on shading cues [2]. Here we show additional simulations reproducing the following experimental results: (i) High-level after-effects and the time course of adaptation (Figure 1B). For a bistable stimulus the probability of seeing the percept shown during adaptation decays with the duration of the adaptor, with a time constant that is similar to the one found in experiments [1]. (ii) Exploiting a novel stimulus (inset Figure 1A) that for which perception can be biased towards one of the two perceptual alternatives, we find the time for the first perceptual switch in the region where both percepts are equally stable. By adjusting 2 parameters, we could match the observed behavior to the psychophysically measured switching times (Figure 1C). A physiologically-inspired hierarchical (‘deep’) neural model for body motion perception reproduces a multitude of effects that characterize the dynamics of body motion perception. The model makes concrete predictions about the behavior of single cells in body motion-sensitive areas.
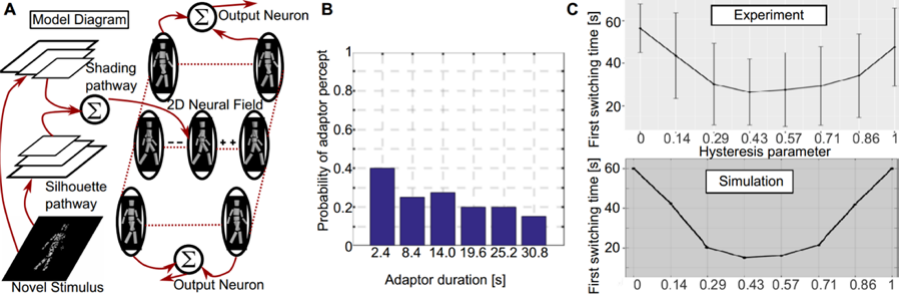


**Figure 1. A.** Model overview. **B.** Probability of perceiving bistable test stimulus as adaptor as function of adaptor duration. **C.** Time to first perceptual switch as function of biasing parameter that controls preference for the two alternative percepts. Top panel: experimental data. Bottom panel: model simulation

**Acknowledgements**

HFSP RGP0036/2016; HBP FP7-ICT-2013-FET-F/604102; DFG GZ: KA 1258/15-1.

**References**

1. Troje N., Sadr J., Geyer H., Nakayama K. Adaptation aftereffects in the perception of gender from biological motion. *J Vis* 2006; **6(8):**850–7.

2. Fedorov L., Vangeneugden J., Giese M. Neural model for the influence of shading on the multistability of the perception of body motion. *IJCCI/NCTA Neural Computation Theory and Applications 8* ^*th*^ *Conf. Proceedings.* 2016 69–76.

## P82 Modelling the effects of propofol on neuronal synchronization in network of interneurons

### Laure Buhry^1,2^, Clément Langlet^2^, Francesco Giovannini^1,2^

#### ^1^Neurosys Team, LORIA, CNRS, INRIA CR Nancy Grand Est, Villers-lès-Nancy, F-54500, France; ^2^Université de Lorraine, Vandoeuvre-lès-Nancy, F-54506, France

##### **Correspondence:** Laure Buhry (laure.buhry@inria.fr)

*BMC Neuroscience* 2017, **18(Suppl 1)**:P82

Propofol is a chemical agent commonly used as an intravenous general anesthetic. At the cellular level, this short-acting anaesthetic positively modulates GABAergic inhibitory activity by targeting GABA-A receptors [1]. This type of receptor is widespread in the brain and can be present both within synaptic clefts, as well as on extrasynaptic locations along the dendrites and neuron membrane where they are responsible for tonic inhibition. At the macroscopic level of SEEG (deep Stereographic-Electroencephalogram) or EEG (Electroencephalogram) recordings, one observes, with certain doses of propofol, a paradoxical excitation phenomenon [2] the generation mechanisms of which are not clearly understood. In this study, we suggest a potential mechanism for the appearance of paradoxical excitation occurring under propofol-induced general anaesthesia.

We show, with a model network of Hodgkin-Huxley neurons, that tonic inhibition – induced by the binding of propofol to extra-synaptic receptors – together with an increase of the synaptic time constant within a certain range [3] can account for the phenomenon of paradoxical excitation. However, changes in the gain (or conductance) of the synaptic inhibition do not correspond to a sudden increase in neuronal population firing rate nor synchrony as described in the experiments [3]. The action of propofol on extrasynaptic GABAergic receptors was modelled by varying the conductance *g* of a tonic current in the form *I*_*ton*_ = *g(V*-*E*_*ton*_ *)* as described in [4]. Figure 1 shows the evolution of the neuronal population firing rate and the coherence (or synchrony) of the network activity as the tonic inhibition and the synaptic conductance vary. The plots are given for different values of the synaptic time constants. The increase of these three variables, synaptic time constant and conductance, and tonic conductance reflect an increase in propofol doses.
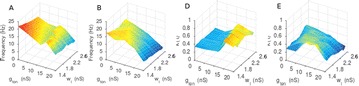


**Figure 1.** Results of the simulations. Neuronal population firing rate and coherence as the dose of propofol varies. **A.** LFP power spectrum from model with no long range connections exhibits no strong peaks. **B.** LFP power spectrum of simulation with long range connections, exhibits clear peaks. **C and D.** contribution of pyramidal cells to LFP depend on long range connections

**Acknowledgements**

Laure Buhry thanks the CNRS for the PEPS JCJC 2016.

**References**

1. C. Vanlersberghe and F. Camu: “Propofol,” in Modern Anesthetics: Handbook of Experimental Pharmacology. 2008. 182: 227–252.

2. Brown, Emery N.: General Anesthesia, Sleep, and Coma. New-England J. of Med. 2011., 363 (27):2638–2650.

3. McDougall, S.J., Bailey, T.W., Mendelowitz, D., Andresen, M.C.: Propofol enhances both tonic and phasic inhibitory currents in second-order neurons of the solitary tract nucleus. Neuropharmacology 2008, 54 (3): 552–563.

4. A. Hutt and L. Buhry: Study of GABAergic extra-synaptic tonic inhibition in single neurons and neural populations by traversing neural scales: application to propofol-induced anaesthesia. J. Comp. Neurosci. 2014, 37: 417–437,

## P83 The dynamical response properties of in silico neurons from the Blue Brain Project digitally reconstructed neocortical microcircuitry

### Christophe Verbist^1^, Stefano Salvadé^1,2^, Michele Giugliano^1^

#### ^1^TNB, Department of biomedical sciences, University of Antwerp, Wilrijk, 2610, Belgium; ^2^DIBRIS, University of Genova, Genova, 16145, Italy

##### **Correspondence:** Christophe Verbist (christophe.verbist@uantwerpen.be)

*BMC Neuroscience* 2017, **18(Suppl 1)**:P83

The onset of an action potential (AP) in cortical neurons has been measured experimentally and found to be much faster than what accounted by classic isopotential membrane theory. Such a sharp onset is more than a measuring artefact and it correlates to the dynamical input–output neuronal bandwidth [1]: it allows reliable transmission of temporal information with frequency content up to 200–300 cycles/s [1]. While this might be accounted for in effective models of excitability, such as the exponential Integrate-and-Fire, it cannot be replicated by conventional single-compartment Hodgkin-Huxley models. These experimental and theoretical evidences led to the three theories, proposed to explain this phenomenon. The first one invokes cooperativity of sodium voltage-gated ionic channels, and it states that when channels open they recruit neighbouring channels to open as well, giving rise to a cascade effect. The second theory invokes APs back propagation from the axon to the soma, and it states a kind of constructive interference occurs at the soma leading to a much sharper spike onset. The third theory is the critical resistive theory [2] and it states that smooth APs generated at the Axon Initial Segment (AIS) become a much sharper at the soma due an electrotonic mismatch between the axon and the soma. Therefore, the sharp AP onset also referred to as kink, is expected to depend on the distance between AIS and the soma.

Very recently, our experimental findings on L2/3 pyramidal human cortical neurons revealed that they relay time-varying inputs with even a broader band than in rodents, while their ensemble firing rate is ~10 Hz [3]. This can be expressed also in terms of an upper cut-off frequency: human cortical cells have a much higher cut-off frequency when an individual suprathreshold sinusoidal input current is injected at the soma.

In this work, we investigate several models of different complexity but the focus is on the biological detailed models of the Blue Brain Project (BBP) [4]. We do a systematic investigation of several of these models. A noisy, Ornstein-Uhlenbeck process, sinusoidal current is injected in the soma. The frequency of this sine is then increased in sequent runs so that a response magnitude curve from the neuron can be extracted, magnitude and phase is extracted from the spike time using circular statistics [5]. Then the axial resistance for a model can be changed, in principle this is the same as increasing the distance between soma and AIS. From this analysis, it can be seen that the cut-off frequency is indeed increasing with increasing axial resistance. These magnitude curves can also be fitted by a Bode function to estimate the cut-off frequency more accurately.

**Conclusion:** Increasing the axial resistance between soma and axon changes the shape of the action potential at the soma, this due to the critical resistive coupling between both. We see that this change in rapidness of onset has a direct consequence on the ability of the neuron to follow up to a particular frequency. In other words, increasing the axial resistance the cut-off frequency is also increasing.

**References**

1. Köndgen et al.: The dynamical response properties of neocortical neurons to temporally modulated noisy inputs in vitro. *Cerebral Cortex* 2008, **18(9):**2086–2097.

2. Brette R.: Sharpness of Spike Initiation in Neurons Explained by Compartmentalization. *PLoS Comput Biol* 2013, **9(12):** e1003338.

3. Testa-Silva et al.: High bandwidth synaptic communication and frequency tracking in human neocortex. *PLoS Biology* 2014, **12(11):**e1002007.

4. Yu Y. et al.: Cortical Action Potential Backpropagation Explains Spike Threshold Variability and Rapid-Onset Kinetics. *The Journal of Neuroscience* 2008, **28(29):**7260–7272

5. Ilin V. et al.: Fast Computations in Cortical Ensembles Require Rapid Initiation of Action Potentials. *The Journal of Neuroscience* 2013, **33(6):**2281–2292

## P84 Self-Organized Balanced Spiking Neural Networks To Encode Natural Stimuli

### James A. Henderson, Pulin Gong

#### School of Physics, The University of Sydney, Sydney, Australia

##### **Correspondence:** James A. Henderson (james.henderson@sydney.edu.au)

*BMC Neuroscience* 2017, **18(Suppl 1)**:P84

Experimental observations of plasticity report key features like Hebbian spike timing and homeostatic mechanisms; however, there is great variability within these mechanisms. While many models of plasticity use an ad hoc selection of experimentally motivated rules to produce other experimental observations like balanced excitatory and inhibitory currents or variable spiking, what is really needed are attempts at theoretical frameworks under which the large variety of observations of plasticity and neural dynamics can be explained and reduced.

One approach to developing such a theoretical framework is to consider neural coding as an outcome of an optimization process implemented in part with plasticity rules. However, studies of neural coding and plasticity are largely separate from each other even though the purpose of plasticity should be to produce a useful neural code. Thus, a theoretical framework of learning that connects neural coding to plasticity is needed to describe how a neural network can learn to perform tasks via optimizing a neural code using plasticity in a way that is biologically plausible and constrained by relevant experimental observations. By establishing this connection, it then becomes possible to explicitly link experimental observations to function and explain why for example, balance between excitatory and inhibitory currents aid the brain in its information processing.

Here we describe a learning scheme for spiking neural networks that learns so that the activity of an individual neuron can be inferred or decoded from the activity of other neurons. The theory begins by proposing three broad biological plausibility constraints that the learning scheme be spike driven, able to be implemented online and only require local information in the network. These constraints are then used to develop biologically plausible plasticity rules that implement learning. Two rules are derived with a precise relation between each other; a Hebbian rule with similarities to existing models of STDP that increases connection strengths, and a homeostatic rule that decreases connection strengths but does not attempt to set a fixed target firing rate as is common in many models of homeostatic plasticity. We use considerations of stability to constrain the parameters of the learning scheme and show that the learning scheme optimizes toward a tight balance of excitatory and inhibitory currents (Fig. 1A). We present a demonstration in which a complex real world stimulus obtained from a Dynamic Vision Sensor (DVS) camera is encoded in the population activity of a spatially extended, conductance based spiking network [2] in which all connections are learned using this scheme (Fig. 1B). The resulting distributions of connection weights are long tailed and approximately log-normal, as found experimentally (Fig. 1C).
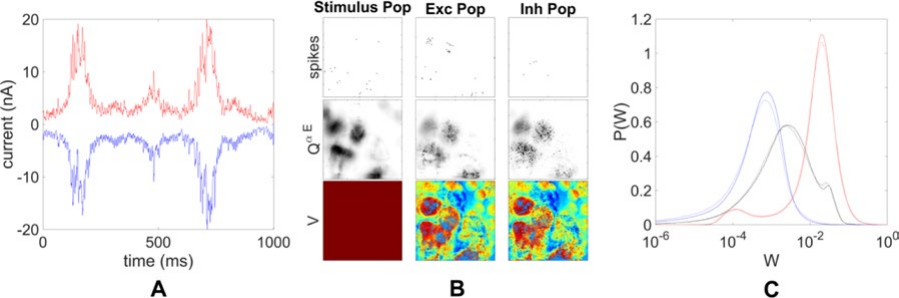


**Figure 1. A.** Example excitatory (red) and inhibitory (blue) currents. **B.** Spikes (row 1), inferred spiking activity (row 2) and membrane potential (row 3) of the 2D stimulus, excitatory and inhibitory populations. **C.** Distribution of connection weights after learning for excitatory (blue), inhibitory (red) and excitatory stimulus (black) connections

**Acknowledgements**

This work was supported by the Australian Research Council and the Centre for Integrative Brain Function.

**Reference**

1. Henderson JA, Gibson T, Wiles J: Spike event based learning in neural networks. *Arχiv*, 2015, 1502.05777.

## P85 Transiently attracting states in recurrent neural networks

### Hendrik Wernecke^1^, Bulcsú Sándor^1^, Claudius Gros^1^

#### ^1^Institute for Theoretical Physics, Goethe University, Frankfurt am Main, 60438, Germany

##### **Correspondence:** Hendrik Wernecke (wernecke@th.physik.uni-frankfurt.de)

*BMC Neuroscience* 2017, **18(Suppl 1)**:P85

For effective cognitive information processing the interplay of different time scales, from milliseconds to hours, is necessary. In neural networks with distinct time scales fixpoints of the fast subsystem are destroyed by the additional evolution of the slow subsystem but remain present as so-called attractor relicts [1]. These points of slow flow that form the slow manifold can still have large influence on the evolution of a dynamical state guiding it along the manifold (see Fig. 1A) and representing a *transiently attracting state*. However, for high dimensional systems it is computationally challenging to compute all transiently attracting states. On the other hand, for a given system it might not be obvious whether the dynamics is effectively shaped by the slow manifold and how strong this effect is.

In this contribution, we introduce the concept of transiently attracting states for general neural networks incorporating a fast and a slow subsystem denoting with 0 < ε<1 the difference in time scales. We define (un)stable *adiabatic fixpoints* (AFP) as the (un)stable fixpoints of the fast subsystem that are recovered for the full system in the adiabatic limit ε → 0. Considering only the one (stable) AFP that the system converges in the adiabatic limit we can define for every state exactly one transiently attracting state on the slow manifold as corresponding target *point* (see Fig. 1 B) and characterize its effect on the overall evolution of the system [2]. Therefore, we investigate the average distance of a trajectory to the corresponding target points and the distribution of the distance. By these measures, we are able to quantify the influence of transiently attracting states on the time evolution.

In particular, we examine the AFP and target points for a three-neuron system that consists of continuous-time point neurons with the intrinsic adaption being substantially slower than the primary neural activity [3]. For this system we find both, states that are highly influenced by the transiently attracting states and states that are protected by symmetry from settling close to the slow manifold. The transition between the different regimes is investigated by evaluating the average distance of the trajectory to the target points. Additionally, we describe a chaotic state in terms of the transiently attracting states gaining insight on the origin of chaos in this system.
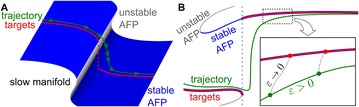


**Figure 1.** Trajectory and AFP. **A.** The slow manifold (surface) consists of all stable (blue) and unstable (gray) adiabatic fixed points (AFP). The target manifold (red line) consists of all target points corresponding to the trajectory (green line) and it is a subset of the set of stable AFP. The trajectory follows the target points slightly delayed. **B.** At every point on the trajectory (green bullets) the fast subsystem is attracted by the corresponding target point (red bullets). In the adiabatic limit ε → 0 the system converges (dashed lines) to the target point. For a finite flow in the slow subsystem, viz when ε > 0, the system evolves along the trajectory (green line). The entirety of target points corresponding to a trajectory forms the target manifold (red line) mapping the trajectory to the slow manifold

**Acknowledgements**

The authors acknowledge this research being supported by funds of the German Research Foundation DFG and Stiftung Polytechnische Gesellschaft Frankfurt am Main.

**References**

1. Linkerhand M, Gros C: Generating functionals for autonomous latching dynamics in attractor relict networks. *Sci Rep* 2013, **3**:2042(2013).

2. Wernecke H, Sándor B, Gros C: Attractor metadynamics in a recurrent neural network: adiabatic vs. symmetry protected flow. *arXiv preprint* 2016, arXiv:1611.00174.

3. Marković D, Gros C: Intrinsic adaptation in autonomous recurrent neural networks. *Neural Comput* 2012, **24(2)**:523–540.

## P86 Characterization of resting state dynamics in monkey motor cortex

### Nicole Voges^1^, Paulina Dabrovska^1^, Johanna Senk^1^, Espen Hagen^2^, Alexa Riehle^1,3^, Thomas Brochier^3^, Sonja Grün^1,4^

#### ^1^Institute of Neuroscience and Medicine (INM-6) & Institute for Advanced Simulations (IAS-6) & JARA Brain Institute I, Juelich Research Centre, Juelich, Germany; ^2^Department of Physics, University of Oslo, Oslo 0316, Norway; ^3^Institut de Neurosciences de la Timone (INT), CNRS - Aix Marseille Universitée, Marseille, France; ^4^Theoretical Systems Neurobiology, RWTH Aachen University, Aachen, Germany

##### **Correspondence:** Nicole Voges (n.voges@fz-juelich.de)

*BMC Neuroscience* 2017, **18(Suppl 1)**:P86

Nowadays, modeling studies of cortical network dynamics aim to include realistic assumptions on structural and functional properties of the corresponding neurons [1,2]. Such models often do not consider functional aspects but rather describe the “ground”, “idle”, or “resting state” of the cortical network, typically characterized as asynchronous irregular spiking [2]. However, for model validation, i.e., for a concrete comparison of experimental versus model data aiming at a more realistic model, one needs to compare this cortical state to the corresponding experimental data. Therefore, we performed a “resting state” experiment (this term is adapted from human fMRI studies where it is defined as brain activity observed when the subject is at rest [3]). We recorded the neuronal activity from macaque monkey motor cortex with a chronically implanted 4x4 mm^2^ 100 electrode Utah Array (Blackrock Microsystems) for 15 min, while the monkey was sitting in a chair without any task or given stimulus. This is in contrast to most neurophysiological studies that focus on a task- or stimulus-specific analysis [e.g. 4]. Based on a video recording of the monkey during the neuronal recording, we differentiate between “resting” intervals and intervals when the monkey spontaneously moved. The goal of this study is to thoroughly characterize the simultaneous spiking activity recorded from 146 single units during resting state. To enable a detailed comparison to simulated spiking data, we subdivide the single units into putative excitatory and inhibitory neurons based on their spike shapes [5]. We apply common statistical measures, e.g., firing rate, (local) coefficient of variation for single unit characterization, and we also compute the pairwise fine temporal correlation by correlation coefficients. These measures are calculated in two ways: averaged over time and single units, as well as averaged over time but separately for each single unit (except for the correlation coefficients). Comparing the distributions of these measures from the two behavioral states we do not find any difference – when averaging over single units. However, when focusing on non-averaged, single unit data we notice that some neurons increase their firing rates systematically when the monkey moves compared to rest, whereas others decrease or do not change their rates. Thus, there was seemingly no difference on the population level, but significant differences on the level of individual neurons. Moreover, we observe a strong correlation between a few neuronal units, independent of their cortical distance, while others show lower or no correlation. Our next steps are to characterize if such findings are particularly different for excitatory and inhibitory neurons. Further, we aim to study the underlying network mechanisms. One possibility would be to re-consider the balancing effects of inhibition and recurrence [5,6].

**Acknowledgements**

Supported by DFG GR 1753/4-1 Priority Program (SPP 1665), Helmholtz Portfolio Theme Supercomputing and Modeling for the Human Brain (SMHB), EU Grant 720270 (HBP).

**References**

1. Potjans TC, Diesmann M: The cell-type specific cortical microcircuit: relating structure and activity in a full-scale spiking network model. *Cereb Cortex* 2014, **24**:785–806.

2. Voges N, Perrinet L: Complex dynamics in recurrent cortical networks based on spatially realistic connectivities. *Front Comput Neurosci* 2012, **6** (41).

3. Biswai BB: Resting state fMRI: A personal history. *Neuroimage* 2012, **62**:938–944.

4. Torre E, Quaglio P, Denker M, Brochier T, Riehle A, Grün S: Synchronous Spike Patterns in Macaque Motor Cortex during an Instructed-Delay Reach-to-Grasp Task. *J Neurosci* 2016, **36**: 8329–8340.

5. Dehghani N, Peyrache A, Telenczuk B, Le Van Quyen M, Halgren E, Cash SS, Hatsopoulos NG, Destexhe A: Dynamic Balance of Excitation and Inhibition in Human and Monkey Neocortex. *Scientific Reports* 2016, **6**:23176.

6. Tetzlaff T, Helias M, Einevoll GT, Diesmann M: Decorrelation of neural-network activity by inhibitory feedback. *PLoS Comp Biol* 2012, **8(8):**e1002596.

## P87 Sharp wave ripples as propagating patterns emerging from spatially extended neural circuits

### Yifan Gu^1^, Pulin Gong^1^

#### ^1^School of Physics and Australian Research Council Centre of Excellence for Integrative Brain Function, University of Sydney, Sydney, NSW 2006, Australia

##### **Correspondence:** Pulin Gong (pulin.gong@sydney.edu.au)

*BMC Neuroscience* 2017, **18(Suppl 1)**:P87

Sharp wave ripples (SPW-Rs) are short episodes of increased activity with superimposed high-frequency oscillations (100–250 Hz). It has been found that SPW-Rs are crucial for memory consolidation, forward and reverse memory replay and constructive planning; however, there is a general lack of understanding of the dynamical nature and the circuit mechanism of these high frequency oscillations. In this study, we develop and investigate a spatially extended spiking neural circuit that incorporates the widely observed heterogeneous properties of neural connectivity, including the lognormal distribution of neural coupling strengths and heterogeneous in- and out-degrees of connections. We show that in this spatially extended circuit with heterogeneous connectivity, sharp wave ripples can emerge (Fig. 1A) when excitation and inhibition are tightly balanced. We analyze the condition of the existence of balanced excitation and inhibition in our spiking circuit model. We show that SPW-Rs propagate across the circuit as wave packets (Fig. 1B) with their propagation properties quantitatively consistent with those measured in [1]; these propagation properties, however, cannot be reproduced in existing modeling studies. In addition, we find that the dynamics of the wave packets can quantitatively account for a wide range of experimentally observed features of SPW-Rs. These features include: (a) strong positive correlations between sharp wave amplitude and ripple frequency, (b) log-normal distributions of population synchrony during ripples [2], (c) heavily tailed distributions of inter-event intervals, and (d) the exponential build-up of EPSP and IPSPs during ripple initiation [3]. Based on the dynamics of these wave packets, we also identify network mechanism for both SPW-R initiation and termination. These results thus provide new insights into understanding the circuit mechanism of SPW-Rs and suggest that their propagation properties may be essential for communicating information from the hippocampus to its cortical targets.
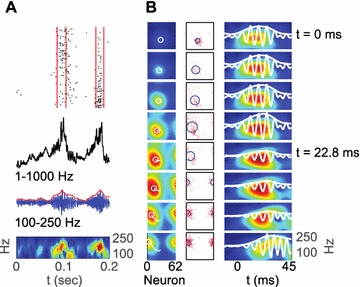


**Figure 1.A** Two detected SWP-R events in the model. B. Left column: snapshots of ripple power in the spatially extended network; middle column: snapshots of spikes; right column: wavelet maps recorded from the corresponding ripple power peak locations

**References**

1. Patel, J., Schomburg, E. W., Berényi, A., Fujisawa, S., & Buzsáki, G.: Local generation and propagation of ripples along the septotemporal axis of the hippocampus. *Journal of Neuroscience* 2013, **33**: 17029–17041.

2. Mizuseki, Kenji, and György Buzsáki: Preconfigured, skewed distribution of firing rates in the hippocampus and entorhinal cortex. *Cell reports* 2013, **4**: 1010–1021.

3. Schlingloff, D., Káli, S., Freund, T. F., Hájos, N., & Gulyás, A. I.: Mechanisms of sharp wave initiation and ripple generation. *Journal of Neuroscience* 2014, **34**:11385–11398

## P88 Macroscopic Phase-Resetting Curve of Spiking Neural Networks: Theory and Application

### Grégory Dumont, Boris Gutkin

#### Group for Neural Theory, Ecole Normale Supérieure, 29, rue d’Ulm, 75005 Paris, France

##### **Correspondence:** Grégory Dumont (gregory.dumont@ens.fr)

*BMC Neuroscience* 2017, **18(Suppl 1)**:P88

The study of brain rhythms is one of the most challenging subjects of interest in neuroscience. An understanding of their functional implications and computational roles could be facilitated by the use of phase resetting curve (PRC); a powerful analytical tool in use to study rhythms [2,9]. However, the topic of PRC for global oscillations observed at the macroscopic scale in neural circuits has received little attention so far. The reason is that macroscopic rhythms emerge from the synaptic interaction of thousands of spiking cells. Although we look at the network as an oscillator and define its phase cycle in term of ongoing self-sustained rhythmic activity, it is made up of individual units which are not oscillators.

In this study, we take advantage of a thermodynamic approach combined with the Ott-Antonsen theory. The thermodynamic framework produces an analytically tractable population models written in term of a partial differential equation (PDE), from which we extract the firing rate of the spiking network [5]. The Ott-Antonsen theory, see [10], allows further reduction and breaks down the PDE into a low dimensional system [6,8]. Bifurcation analysis of the reduced system enables us to reveal how synaptic interactions and inhibitory feedback permit the emergence of macroscopic rhythms. The usual adjoint method can then be applied and a semi-analytical expression of the macroscopic infinitesimal PRC is derived [3].

Our analytical computations allow us to make key predictions. First, we observed that only stimulus targeting the inhibitory cells can generate a biphasic PRC. Such PRCs are known to facilitate entrainment to periodic inputs at both higher and lower frequencies than the natural frequency of the network. Then we investigate the effect of coupling strength and transmission delay on the dynamical emergence of phase locking mode within two bidirectionally delayed-coupled spiking networks. Within the framework of weakly coupled oscillators, we clarify why macroscopic oscillations show phase relations that are persistent across time, and provide reasons for the reported diversity of phase lags between cortical regions [7]. For the first time, we bring theoretical support regarding the strong implication of inhibitory cells not only in the emergence of macroscopic rhythms, but also on phase locking modes between different neural circuits engaged in rhythmic oscillatory patterns. Our predictions are supported by extensive numerical simulations and are consistent with empirical data [1,4].

**Acknowledgements**

This study was funded by the Russian Science Foundation grant (contract number: 17-11-01273)

**References**

1. Akam T, Oren I, Mantoan L, Ferenczi E, Kullmann DM: Oscillatory dynamics in the hippocampus support dentate gyrus ca3 coupling. *Nat Neurosci* 2012, **15(5):** 763–768.

2. Ashwin P, Coombes S, Nicks R: Mathematical frameworks for oscillatory network dynamics in neuroscience. *The Journal of Mathematical Neuroscience* 2016, **6(1):** 2. DOI 10.1186/s13408-015-0033-6.

3. Brown E, Moehlis J, Holmes P: On the phase reduction and response dynamics of neural oscillator populations. *Neural Computation* 2004, **16(4):** 673–715. DOI 10.1162/089976604322860668.

4. Cardin JA, Carlen M, Meletis K, Knoblich U, Zhang F, Deisseroth K, Tsai LH, Moore CI: Driving fast-spiking cells induces gamma rhythm and controls sensory responses. *Nature* 2009, **459(7247):** 663–667.

5. Deco G, Jirsa VK, Robinson PA, Breakspear M, Friston K: The dynamic brain: From spiking neurons to neural masses and cortical fields. *PLoS Comput Biol* 2008, **4(8):**1–35. DOI 10.1371/journal.pcbi.1000092.

6. Luke TB, Barreto E, So P: Complete classification of the macroscopic behavior of a heterogeneous network of theta neurons. *Neural Computation* 2013, **25(12):** 3207–3234. DOI 10.1162/NECOfngafng00525.

7. Maris E, Fries P, van Ede F: Diverse phase relations among neuronal rhythms and their potential function. *Trends in Neurosciences* 2015, **39(2):** 86–99. DOI 10.1016/j.tins.2015.12.004.

8. Montbrio, E., Pazo, D., Roxin, A.: Macroscopic description for networks of spiking neurons. *Phys. Rev.* 2015, X **5:**021,028. DOI 10.1103/PhysRevX.5.021028.

9. Nakao, H.: Phase reduction approach to synchronisation of nonlinear oscillators. *Contemporary Physics* 2016, **57(2):**188–214. DOI 10.1080/00107514.2015.1094987.

10. Ott E, Antonsen TM: Low dimensional behavior of large systems of globally coupled oscillators. *Chaos* 2008, **18(3):** 037113. DOI http://dx.doi.org/10.1063/1.2930766.

## P89 Semi-numerical method for computationally effective analysis of working memory models

### Nikita A. Novikov^1^, Boris S Gutkin^1,2^

#### ^1^Centre for Cognition and Decision Making, National Research University Higher School of Economics, Moscow, 101000, Russia; ^2^Department of Cognitive Studies, Ecole Normale Superieure PSL* Research University, Paris, 75005, France

##### **Correspondence:** Nikita A. Novikov (nikknovikov@gmail.com)

*BMC Neuroscience* 2017, **18(Suppl 1)**:P89

Working memory is the ability to temporarily retain information about sensory stimuli that are no longer directly perceived. Retention of working memory trace is associated with elevated spiking activity in specific subsets of neurons [1]. From the computational perspective, it could be implemented by a bistable neural network, one stable state of which corresponds to the background firing, and the other one – to the elevated spiking activity during retention of the memory trace [2]. A major problem of classical WM models is an excessive regularity of spike trains in the active state. Recently, a solution for this issue was proposed, based on a balanced network with short-term synaptic potentiation [3,4]. In such a model, mean input current to the neurons stays near zero in the active state, while variation of the current increases due to increased synaptic weights. The exact mathematical solution of this model is complicated because of the need to account for the effect of varying level of noise on the firing rates. In this work, we propose a computationally effective method for predicting the behavior of the model based on nullcline analysis and low-dimensional population models. The method is based on a pre-calculation of neuronal gain functions under two Ornstein-Uhlenbeck current inputs (representing synaptic currents from excitatory and inhibitory subpopulations) in the nodes of 3-dimensional grid (coordinates: mean input, variances of both inputs) with subsequent numerical interpolation of the results. In turn, steady-state values of input means and variances can be found analytically as functions of instantaneous firing rates, which allows us to find self-consistent solutions numerically. The computationally expensive step in this method (pre-calculation) depends only on the parameters of the neurons and on the synaptic time constants (which could be determined from experiments), so the analysis can be easily performed for various combination of synaptic weights (which are usually unknown and should be tuned to yield an appropriate model behavior). Using the described method, we perform nullcline analysis and build a low-dimensional model of WM based on exponential convergence of firing rates, as well as means and variances of the synaptic currents, to the corresponding steady-states obtained using the method described above. We demonstrate that the behavior of this model is similar to the behavior of the corresponding spiking network (see Figure 1).
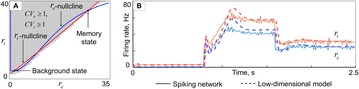


**Figure 1. A.** Nullclines of the WM model. **B.** Population firing rate dynamics

**Acknowledgements**

Supported by Russian Science Foundation grant (contract number: 17-11-01273)

**References**

1. Goldman-Rakic PS: Cellular basis of working memory. *Neuron* 1995, **14(3):**477–485.

2. Amit DJ, Brunel N: Model of global spontaneous activity and local structured activity during delay periods in the cerebral cortex. *Cereb Cortex* 1997, **7(3):**237–252.

3. Hansel D, Mato G: Short-term plasticity explains irregular persistent activity in working memory tasks. *J Neurosci* 2013, **33(1):**133–149.

4. Mongillo G, Hansel D, van Vreeswijk C: Bistability and spatiotemporal irregularity in neuronal networks with nonlinear synaptic transmission. *Phys Rev Lett* 2012, **108(15):**158101.

## P90 Workflow for model building, parameter estimation and uncertainty analysis applied to calcium- and G-protein dependent subcellular signaling underlying synaptic plasticity

### Parul Tewatia^1†^, Olivia Eriksson^1,2†^, Andrei Kramer^2†^, Joao Santos^3^, Alexandra Jauhiainen^4^, Kim T Blackwell^5^, Jeanette H Kotaleski^1,3^

#### ^1^KTH Royal Institute of Technology, School of Computer Science and Communication, Stockholm, Sweden; ^2^Stockholm University, Department of Numerical Analysis and Computer Science, Stockholm, Sweden; ^3^Karolinska Institute, Department of Neuroscience, Solna, Sweden; ^4^Early Clinical Biometrics, AstraZeneca AB R&D, Gothenburg, Sweden; ^5^Computational and Experimental Neuroplasticity Laboratory, Krasnow Institute for Advanced Study, George Mason University, George Mason, VA, USA

##### **Correspondence:** Parul Tewatia (ptewatia@kth.se), Olivia Eriksson (olivia@kth.se), Andrei Kramer (andreikr@kth.se)

^†^Contributed equally to this work

*BMC Neuroscience* 2017, **18(Suppl 1)**:P90

When modelling subcellular signalling pathways, experimental data are integrated into a precise and structured framework from which it is possible to make predictions that could be tested experimentally, thereby facilitating the understanding of the biological mechanisms involved. The quantitative experimental data that are used for building the models are often sparse as compared to the size and complexity of the modelled system, and the translation of these data into dynamical models therefore leaves numerous uncertainties in parameter values. An explicit description of this uncertainty is useful in order to precisely describe assumptions that are made during the modelling process concerning parameters as well as the data that used for parameter estimation.

We have earlier developed a workflow for building and testing intracellular signalling models and for the quantification of model parameter uncertainty, and its propagation to predictions [1]. This workflow was applied on a model describing calcium (Ca)-dependent activation of Calmodulin (CaM), Protein phosphatase 2B (PP2B) and Ca/CaM-dependent protein kinase II (CaMKII) [2]. We here develop this workflow further and also apply it on the G-protein coupled cascade underlying endocannabinoid production [3]. Both CaMKII and endocannabinoids (eCBs) are important for synaptic plasticity in many brain areas. While CaMKII is often involved in LTP, eCBs rather promote LTD.

The model parameter uncertainty analysis is performed through a Bayesian sampling of the region of parameter values which produced a good fit to available experimental data. Two different methods are used: data-set iterative Approximate Bayesian Computation (ABC) [4,1] and Simplified Manifold Metropolis Adjusted Langevin Algorithm (SMMALA) [5]. This estimation of posterior parameter uncertainty translates to uncertainties in predictions made from the model, and finally, a global sensitivity analysis helps in determining the importance and role of different parameters for different model outputs.

**References**

1. Eriksson O, Jauhiainen A, Sasane SM, Nair AG, Sartorius C, Kotaleski JH: Statistical uncertainty and sensitivity analysis of intracellular signaling models - through approximate Bayesian computation and variance based global sensitivity analysis, 2016, http://www.frontiersin.org/10.3389/conf.fninf.2016.20.00014/event_abstract

2. Nair AG, Gutierrez-Arenas O, Eriksson O, Jauhiainen A, Blackwell KT, Kotaleski JH: Modeling intracellular signaling underlying striatal function in health and disease. *Prog. Mol. Biol. Transl. Sci.* 2014, **123**:277–304.

3. Kim B, Hawes SL, Gillani F, Wallace LJ, Blackwell KT: Signaling Pathways Involved in Striatal Synaptic Plasticity are Sensitive to Temporal Pattern and Exhibit Spatial Specificity. *PLoS Comput. Biol.* 2013, **9(3)**: e1002953.

4. Marjoram P, Molitor J, Plagnol V, and Tavare S: Markov chain Monte Carlo without likelihoods. *Proc. Natl. Acad. Sci. U.S.A*. 2003, 100:15324–15328.

5. Girolami M, Calderhead B: Riemann manifold Langevin and Hamiltonian Monte Carlo methods. *J. R. Statist. Soc. B,* 2011, **73.2**: 123–214.

## P91 The role of striatal feedforward inhibition in propagation of cortical oscillations

### Jovana J. Belić^1,2,3^, Arvind Kumar^2,3^, Jeanette Hellgren Kotaleski^1,2,4^

#### ^1^Science for Life Laboratory, Royal Institute of Technology, Solna, 17165, Sweden; ^2^Department of Computational Science and Technology, Royal Institute of Technology, Stockholm, 11428, Sweden; ^3^Bernstein Center Freiburg, University of Freiburg, Freiburg, 79104, Germany; ^4^Department of Neuroscience, Karolinska Institute, Solna, 17177, Sweden

##### **Correspondence:** Jovana J. Belić (belic@kth.se)

*BMC Neuroscience* 2017, **18(Suppl 1)**:P91

Fast spiking interneurons (FSIs) and feedforward (FF) inhibition are a common property of neuronal networks throughout the brain and play crucial role in neural computations. For instance, in the cortex FF inhibition sets the window of temporal integration and spiking and thereby contributes to the control of firing rate and correlations [1]. In the striatum (the main input structure of the basal ganglia) despite their high firing rates and strong synapses, FSIs (comprise 1–2% of striatal neurons) do not seem to play a major role in controlling the firing of medium spiny neurons (MSNs; comprise 95% of striatal neurons) [2] and so far, it has not been possible to attribute a functional role to FSIs in the striatum. Here we use a spiking neuron network model in order to investigate how externally induced oscillations propagate through striatal circuitry. Recordings in the striatum have shown robust oscillatory activity that might be in fact cortical oscillations transmitted by the corticostriatal projections [3–5]. We propose that FSIs can perform an important role in transferring cortical oscillations to the striatum especially to those MSNs that are not directly driven by the cortical oscillations. Strong and divergent connectivity of FSIs implies that even weak oscillations in FSI population activity can be spread to the whole MSN population [6]. Further, we have identified multiple factors that influence the transfer of oscillations to MSNs. The variables such as the number of activated neurons, ongoing activity, connectivity, and synchronicity of inputs influence the transfer of oscillations by modifying the levels of feedforward and feedback inhibitions suggesting that the striatum can exploit different parameters to impact the transfer of oscillatory signals.

**References**

1. Isaacson, J. S., & Scanziani, M. (2011). How inhibition shapes cortical activity. Neuron, 72(2), 231–243.

2. Berke, J. D. (2011). Functional properties of striatal fast-spiking interneurons. Frontiers in systems neuroscience, 5.

3. Belić, J. J., Halje, P., Richter, U., Petersson, P., & Kotaleski, J. H. (2016). Untangling cortico-striatal connectivity and cross-frequency coupling in L-DOPA-induced dyskinesia. Frontiers in systems neuroscience, 10.

4. Berke, J. D. (2009). Fast oscillations in cortical‐striatal networks switch frequency following rewarding events and stimulant drugs. European Journal of Neuroscience, 30(5), 848–859.

5. Boraud, T., Brown, P., Goldberg, J. A., Graybiel, A. M., & Magill, P. J. (2005). Oscillations in the basal ganglia: the good, the bad, and the unexpected. In The basal ganglia VIII (pp. 1–24). Springer US.

6. Belić, J. J., Kumar, A., & Kotaleski, J. H. (2017). Interplay between periodic stimulation and GABAergic inhibition in striatal network oscillations. PloS one, 12(4), e0175135.

## P92 Electrical propagation on Cortical Connectome and Communicability

### Masanori Shimono^1,3^, Naomichi Hatano^2^

#### ^1^Osaka University, Toyonaka, Osaka, Japan; ^2^University of Tokyo, Bunkyo, Tokyo, Japan; ^3^Riken Brain Science Institute, Saitama, Japan

##### **Correspondence:** Masanori Shimono (smn@bpe.es.osaka-u.ac.jp)

*BMC Neuroscience* 2017, **18(Suppl 1)**:P92

The brain is macroscopically an organ transmitting electrical currents on complex networks through white matter bundles. How are the temporal dynamics, especially the temporal delays or speeds for transmitting electrical currents among brain regions, determined?

This question is currently an important issue for designing physiologically meaningful computational models of the brain. In the past, *Lamme and Roelfsema (2010)* wrote a well-organized review on electrical propagations on the brain [1]. However, because of the complexity of the network architectures, many past studies have only succeeded in showing the speeds in limited pairs of brain regions. This study highlights the connectome, the global whole-brain network, underlining electrical propagations.

We claim that the walks, the number of direct connections between independent nodes on the structural network, are crucially important for predicting whether propagation delays will reach the brain target regions, and the dependency could be captured systematically using the communicability of a well-designed measure in the network theory [2].

The mechanism is as follows: The temporal delays are mainly determined by the walks, the number of direct connections between brain regions. The relative importance, quantified as the weights of individual connections, gradually decays as the number of steps in the walks increase. Communicability systematically evaluated the relative importance depending on the number of paths.

To reach this goal, we integrated three independently developed data sets: neuronal spike data, whole-brain ECoG data, and brain structural networks, which were accumulated independently (see Figure 1). The data were partially used in our past study [3]. The last two data sets were prepared by neuroinformatic contributions from different research groups. Their contributions enabled us to reach these key findings. Our findings will provide an important new bridge between network science and neuroscience.
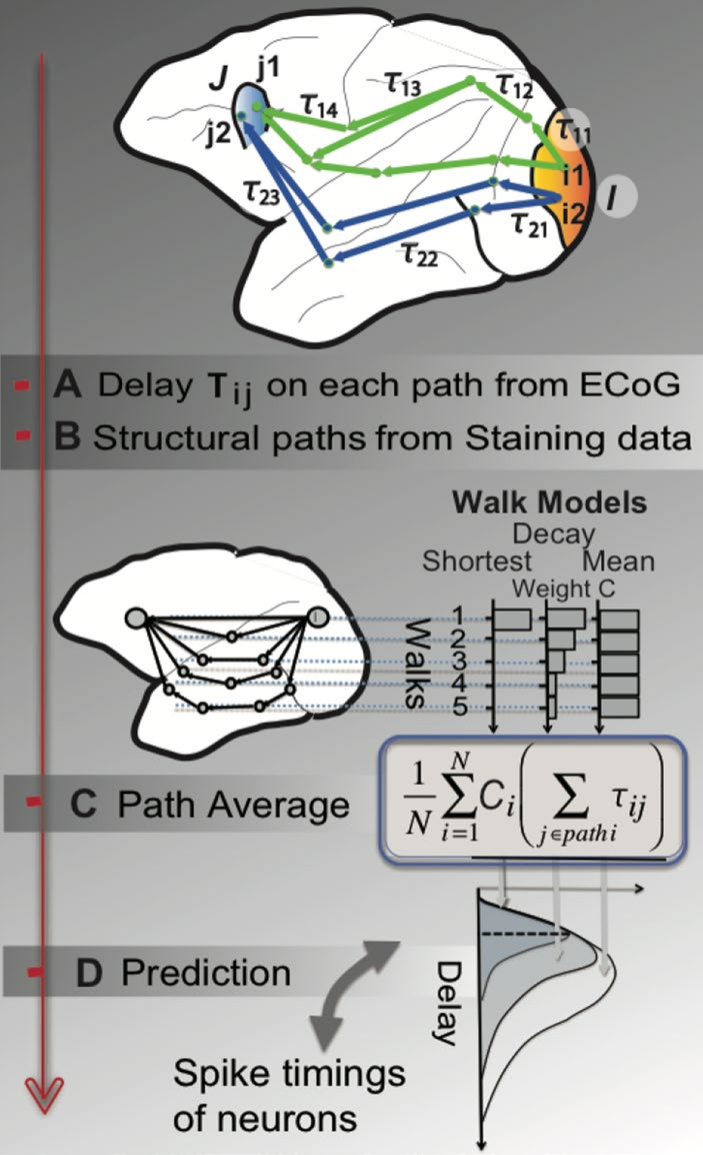


**Figure 1.** A scheme to evaluate the underlining network organization and necessary propagation time. **A.** We gave time delays to individual paths from ECoG. **B.** The underlining paths were given from structural tracer data. **C.** We prepared several models to integrate multiple paths connecting between a pair of a start node and a goal node. **D.** Finally, we evaluated the prediction performance of propagation delays extracted from neuronal spike data

**References**

1. VA Lamme, PR Roelfsema: The distinct modes of vision offered by feedforward and recurrent processing. *Trends in neurosciences*, 2000, ***23*** **(11):** 571–579.

2. E Estrada, N Hatano: Communicability in complex networks. *Physical Review E*, 2008, **77(3):** 036111.

3. M Shimono: Non-uniformity of cell density and networks in the monkey brain. *Scientific reports*, 2013, **3:** 2541.

## P93 A cortical model for learning complex temporal structure in sensory streams

### Subutai Ahmad^1^, Yuwei Cui^1^, Jeff Hawkins^1^

#### ^1^Numenta, Redwood City, CA 94063, USA

##### **Correspondence:** Subutai Ahmad (sahmad@numenta.com)

*BMC Neuroscience* 2017, **18(Suppl 1)**:P93

Extensive experimental evidence demonstrates that sequence learning occurs in multiple cortical regions, but the underlying neural mechanism remains obscure. Here we show that networks of pyramidal neurons can learn to accurately recognize complex temporal sequences in a continuous online fashion. Our neuron model contains non-linear dendrites with distinct synaptic integration zones (Figure 1). Proximal dendrites define the classical receptive field, whereas patterns recognized by distal dendrites act as predictions and have a modulatory effect. The network can learn complex non-Markovian sequences. Our model achieves accuracy on par with deep learning algorithms such as LSTM on real-world sequence prediction tasks. The model achieves superior performance in the areas of online learning, handling branching sequences, and high fault tolerance. The model relies on sparse population codes and makes several detailed predictions regarding the importance of high-order correlations and cell assemblies. We analyzed experimental calcium imaging data from awake mice learning natural sequences to verify many of the predictions. The work represents a theory of sequence memory that integrates many physiological properties of cortical neurons, is applicable to real-world applications, and makes multiple testable experimental predictions.
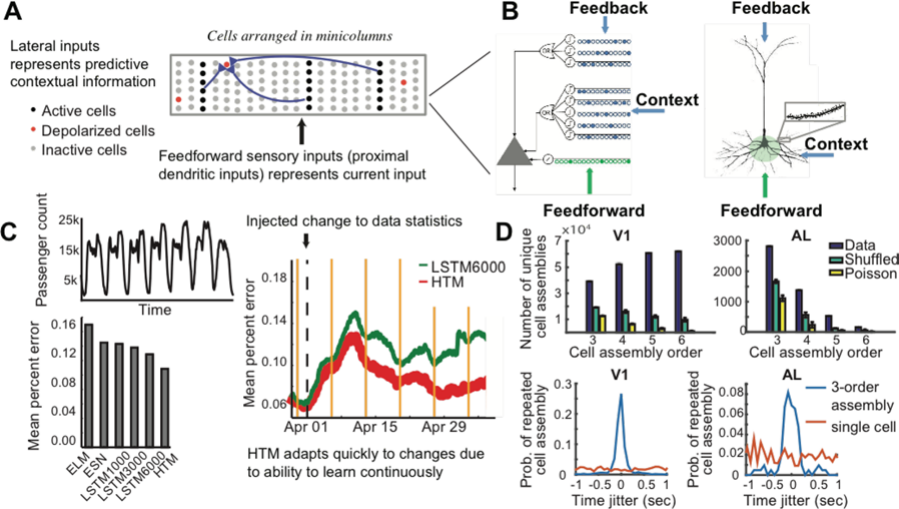


**Figure 1**. **A.** Our sequence memory model (HTM) is organized as a cellular layer with lateral recurrent connections. **B.** Our neuron model (left) has three distinct dendritic integration zones analogous to pyramidal neurons (right). The co-activation of a set of synapses on a distal dendrite have a modulatory effect on the neuron [1]. **C.** Example dataset for predicting NYC taxi passenger counts (top-left). Prediction error for various sequence prediction algorithms (bottom-left). LSTM and HTM have comparable performance [2]. The error for HTM sequence memory (red) and LSTM (green) after artificial manipulation of the data (black dashed line). **D.** Analysis of calcium imaging data shows that the number of high-order cell assemblies in areas V1 and AL is much higher than predicted by Poisson or trial-shuffled control models (top). Unlike single cell responses, high-order cell assemblies occur reliably at the same point within the sequence (bottom). These are consistent with predictions made by our model

**References**

1. Hawkins J, Ahmad S: Why Neurons Have Thousands of Synapses, a Theory of Sequence Memory in Neocortex. *Front. Neural Circuits. Frontiers* 2016 **10**:1–23.

2. Cui Y, Ahmad S, Hawkins J: Continuous online sequence learning with an unsupervised neural network model. *Neural Comput*. 2016, **28**:2474–504.

## P94 Conditions for traveling waves in spiking neural networks obtained from a rigorous mapping to a neural-field model

### Johanna Senk^1^, Karolína Korvasová^1^, Jannis Schuecker^1^, Espen Hagen^1,2^, Tom Tetzlaff^1^, Markus Diesmann^1,3,4^, Moritz Helias^1,4^

#### ^1^Institute of Neuroscience and Medicine (INM-6) and Institute for Advanced Simulation (IAS-6) and JARA BRAIN Institute I, Forschungszentrum Jülich, 52425 Jülich, Germany; ^2^Department of Physics, University of Oslo, Oslo, 0316, Norway; ^3^Department of Psychiatry, Psychotherapy and Psychosomatics, Medical Faculty, RWTH Aachen University, 52074 Aachen, Germany; ^4^Department of Physics, Faculty 1, RWTH Aachen University, 52074 Aachen, Germany

##### **Correspondence:** Johanna Senk (j.senk@fz-juelich.de), Karolína Korvasová (k.korvasova@fz-juelich.de)

*BMC Neuroscience* 2017, **18(Suppl 1)**:P94

Spatiotemporal patterns such as traveling waves are frequently observed in recordings of neural activity [1]. The mechanisms underlying the generation of such patterns are largely unknown. Previous studies addressed this problem in the framework of neural-field models, a phenomenological coarse-grained description of neural-network dynamics, and provided insights on the existence and uniqueness of traveling fronts, bumps or Turing patterns [2]. It remains unclear, however, to what extent these insights can be transferred to networks of spiking neurons. Here, we analyze the dynamics of a network of leaky integrate-and-fire (LIF) neurons positioned on a one-dimensional ring with distance-dependent connection probability. Mean-field theory [3,4] allows us to rigorously map the microscopic network model to a neural-field model in the continuum limit (see Fig. 1). In contrast to the phenomenological descriptions of the past, the neural-field model emerging from our analysis accounts for both the mean and the variance in the synaptic input. In addition, it introduces a working-point dependence in the effective coupling strength and in the shape of the temporal kernel. Using this framework, we derive conditions for the existence of periodic traveling waves in networks of spiking neurons. We show that periodic traveling waves cannot occur in a single homogeneous population of neurons with homogeneous (position independent) synaptic weights, delays and external inputs, irrespectively of the form of distance dependence of the connection probability. For two-population networks of excitatory and inhibitory neurons, in contrast, traveling waves emerge for specific types of connection-probability kernels. The predictions of the analytically tractable neural-field model are validated by means of simulations of LIF-neuron networks using NEST [5]. The rigorous mapping between the network and the neural-field model allows us to design connectivity kernels that permit the emergence of traveling waves, or, vice versa, to predict the connectivity structure from the characteristics of observed activity patterns (e.g. wavelength and frequency).
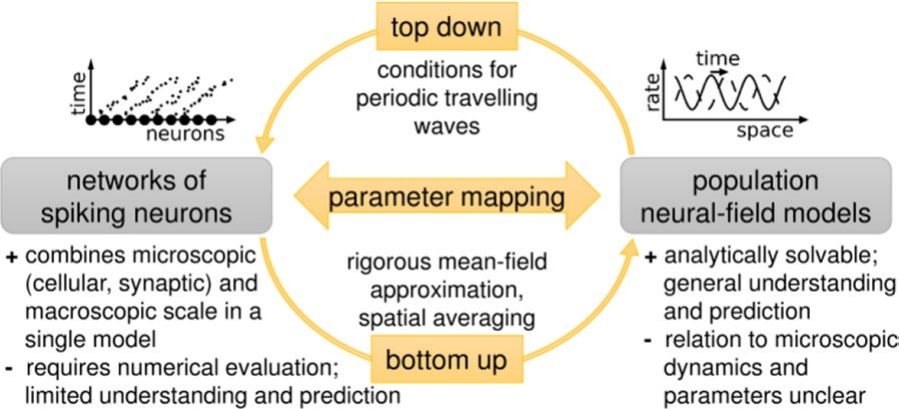


**Figure 1.** Mapping microscopic single-neuron dynamics to spatially averaged population dynamics

**Acknowledgements**

This project has received funding from the Helmholtz association: Portfolio Supercomputing and Modeling for the Human Brain (SMHB), young investigator group VH-NG-1028; from the European Union’s Horizon 2020 research and innovation programme under grant agreement No 720270 (HBP SGA1), the German Research Foundation (DFG; grant DI 1721/3-1 [KFO219-TP9]), and the Research Council of Norway (NFR through COBRA).

**References**

1. Muller L, Destexhe A: Propagating waves in thalamus, cortex and the thalamocortical system: Experiments and models. *J Physiol* 2012, **106**:222–238.

2. Coombes S: Waves, bumps, and patterns in neural field theories. *Biol Cybern* 2005, **93:**91–108.

3. Brunel N, Hakim V: Fast Global Oscillations in Networks of Integrate-and-Fire Neurons with Low Firing Rates. *Neural Comput* 1999, **11:**1621–1671.

4. Schuecker J, Diesmann M, Helias M: Modulated escape from a metastable state driven by colored noise. *Phys Rev* E 2015, **92:**052119.

5. Bos H, Morrison A, Peyser A, Hahne J, Helias M, Kunkel S, Ippen T, Eppler JM, Schmidt M, Seeholzer A et al.: NEST 2.10.0. Zenodo 2015.

## P95 Temporal structure of synchrony and Unitary Events in periodically-driven balanced networks

### Tobias Kühn^1^, Michael Denker^1^, PierGianLuca Mana^1^, Sonja Grün^1,2^, Moritz Helias^1,3^

#### ^1^Institute of Neuroscience and Medicine (INM-6) and Institute of Advanced Simulation (IAS-6) and JARA Brain Institute I, Jülich Research Centre, 52425 Jülich, Germany; ^2^Theoretical Systems Neurobiology, Faculty I, RWTH Aachen University, 52062 Aachen, Germany; ^3^Department of Physics, Faculty I, RWTH Aachen University, 52062 Aachen, Germany

##### **Correspondence:** Tobias Kühn (t.kuehn@fz-juelich.de)

*BMC Neuroscience* 2017, **18(Suppl 1)**:P95

Whether the brain employs the temporal domain for the representation of information is still a matter of ongoing debates. Theory and experiments point toward an entanglement of firing rates and correlations [1]. Moreover, in [2], it was shown by Unitary Event (UE) analysis [3] that the occurrence of excess synchronous spike events of neurons observed in parallel are more strongly locked to the phase of the local field potential (LFP)-beta-oscillations than chance synchronous events or individual spikes, which was related to the concept of cell assemblies.

We want to study the influence of oscillatory drive from remote brain areas - expressed as oscillations in the LFP - on the correlation of single neuron activities in a small cortical subnetwork. A balanced random network of homogeneously connected binary model neurons [4] receiving input from a sinusoidal perturbation [5] captures the main properties of this type of systems and illustrates mechanisms that cause time-modulated covariances. Using linear response theory, we compute the time-dependent averages and covariances of the stochastic neuronal activity in mean-field-theory, which agree with their simulated counterparts given that the perturbation is of the order of the fluctuations of the inputs. We find that the zero-time lag pairwise covariances consist of two terms, one due to the modulated susceptibility (via external input and recurrent feedback) and one due to the time-varying autocovariances. For some connectivity parameters, this leads to resonant covariances and non-resonant mean activities. The resonant behavior of the covariances occurs because the susceptibility is modulated by two terms with different signs and different dependence on the perturbing frequency: The direct drive and the recurrent feedback .

The application of the UE-analysis to data emerging from the model network shows that the probability for UEs to occur is indeed oscillatory already in an unstructured network. A locking as strong as described in [2], however, is not observed. An interesting extension of our model would therefore be to include cell assemblies as additional populations of excitatory neurons that are connected more densely amongst themselves than to the rest [6]. That would allow a closer comparison to experimental findings. However, already the results for the random network can help to answer the salient question how oscillations in mesoscopic signals and spike correlations interact.

**Acknowledgements**

Supported by the Helmholtz foundation (VH-NG-1028, SMHB); EU Grant 720270 (HBP), Simulations with NEST (nest-simulator.org).

**References**

1 J. de la Rocha, B. Doiron, E. Shea-Brown, K. Josic, and A. Reyes, Correlation between neural spike trains increases with firing rate, *Nature* 2007, **448(7155)**:802–806.

2. Denker M, Roux S, Lindén H, Diesmann M, Riehle A, Grün S: The Local Field Potential reflects surplus Spike Synchrony. *Cereb Cortex.* 2011 D, **21**:2681–2695.

3. Grün S, Diesmann M, Aertsen A. ‘Unitary Events’ in Multiple Single-Neuron Spiking Activity. II. Non-Stationary Data. Neural Comput. 2002, **14(1)**:81–119.

4. Ginzburg I, Sompolinsky H: Theory of correlations in stochastic neural networks. *Phys Rev E.* 1994, **50(4)**:3171–3191.

5. Kühn T, Helias M: Correlated activity of periodically driven binary networks. arXiv:1607.08552v2

6. Litwin-Kumar A, Chacron MJ, Doiron B: The spatial Structure of Stimuli shapes the Timescale of Correlations in Population Spiking Activity. *PLoS Comput Biol.* 2012, **8(9)**:e1002667.

## P96 Distributed correlations in motor cortex suggest virtually unstable linearized dynamics

### David Dahmen^1^, Markus Diesmann^1,2,3^, Moritz Helias^1,3^

#### ^1^Institute of Neuroscience and Medicine (INM-6) and Institute for Advanced Simulation (IAS-6) and JARA BRAIN Institute I, Jülich Research Centre, Jülich, Germany; ^2^Department of Psychiatry, Psychotherapy and Psychosomatics, Medical Faculty, RWTH Aachen University, Aachen, Germany; ^3^Department of Physics, Faculty 1, RWTH Aachen University, Aachen, Germany

##### **Correspondence:** David Dahmen (d.dahmen@fz-juelich.de)

*BMC Neuroscience* 2017, **18(Suppl 1)**:P96

Despite the large amount of shared input between nearby neurons in cortical circuits, massively parallel spiking recordings of various in vivo networks exhibit pairwise covariances in ensembles of neuronal spike trains that are on average close to zero [1]. The low average has been well understood in terms of active decorrelation by inhibitory feedback [2,3] in networks that operate far away from the critical point, which marks the onset of avalanche-like activity [4]. Experiments, however, also show large variability of covariances across pairs of neurons. An explanation for their wide distribution in relation to the static (quenched) disorder of the connectivity in recurrent networks is so far elusive. Here we combine ideas from spin-glass theory [5] with a generating function representation for the joint probability distribution of the network activity [6] to derive a finite-size mean-field theory that reduces a disordered to a highly symmetric network with fluctuating auxiliary fields (Fig. 1). The theory relates the statistics of covariances to the statistics of connections, in particular the largest eigenvalue of the connectivity matrix, and explains the experimentally observed covariance distributions [7]. The analytical expressions expose that both, average and dispersion of the latter, diverge at a critical point which has been studied in terms of a transition from regular to chaotic dynamics [8]. This critical point does not arise from net excitation, but rather from disorder in networks with balanced excitation and inhibition. Applying these results to recordings from motor cortex suggests its operation close to this breakdown of linear stability.
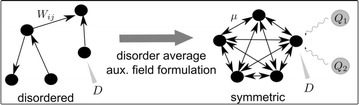


**Figure 1.** Mapping of network with frozen variability in connections to highly symmetric network with fluctuating auxiliary fields

**Acknowledgements**

Supported by HGF young investigator’s group VH-NG-1028, Helmholtz portfolio theme SMHB, and EU Grant 604102 (Human Brain Project, HBP). Data courtesy: A. Riehle and T. Brochier

**References**

1. Ecker AS, Berens P, Keliris GA, Bethge M, Logothetis NK: Decorrelated Neuronal Firing in Cortical Microcircuits. *Science* 2010, **327**:584–587.

2. Renart A, De La Rocha J, Bartho P, Hollender L, Parga N, Reyes A, Harris KD: The asynchronous State in Cortical Circuits. *Science* 2010, **327**:587–590.

3. Tetzlaff T, Helias M, Einevoll G, Diesmann M: Decorrelation of neural-network activity by inhibitory feedback. *PLOS Comput. Biol.* 2010, **8(8)**:e1002596.

4. Beggs JM, Plenz D: Neuronal avalanches in neocortical circuits. *J. Neurosci.* 2003, **23**:11167–11177.

5. Sompolinsky H, Zippelius A: Relaxational dynamics of the Edwards-Anderson model and the mean-field theory of spin-glasses. *Phys. Rev.* B 1982, **25**:6860–6875.

6. Chow C, Buice M: Path Integral Methods for Stochastic Differential Equations. J Math Neurosci. 2015, **5**:8.

7. Dahmen D, Diesmann M, Helias M: Distributions of covariances as a window into the operational regime of neuronal networks. arXiv 2016, 1605.04153 [cond-mat.dis-nn].

8. Sompolinsky H, Crisanti A, Sommers HJ: Chaos in Random Neural Networks, *Phys. Rev. Lett.* 1988, **61**:259–262.

## P97 Transition to chaos and short-term memory in driven random neural networks

### Jannis Schuecker^1^, Sven Goedeke^1^, Moritz Helias^1,2^

#### ^1^Institute of Neuroscience and Medicine (INM-6) and Institute for Advanced Simulation (IAS-6) and JARA BRAIN Institute I, Jülich Research Centre, Jülich, Germany; ^2^Department of Physics, Faculty 1, RWTH Aachen University, Aachen, Germany

##### **Correspondence:** Jannis Schuecker (j.schuecker@fz-juelich.de)

*BMC Neuroscience* 2017, **18(Suppl 1)**:P97

Recurrent networks of randomly coupled rate neurons display a transition to chaos at a critical coupling strength [1]. Their rich internal dynamics emerging near the transition has been associated with optimal information processing capabilities [2]. In particular, the dynamics becomes arbitrary slow at the onset of chaos, similar to ‘critical slowing down’. However, the interplay between time-dependent input signals, network dynamics, and the resulting consequences for information processing are poorly understood.

Here, we investigate the effect of time-varying inputs on the transition to chaos. Using dynamic mean-field theory we determine the largest Lyapunov exponent, which quantities the rate of exponential divergence or convergence of close-by trajectories. We analytically obtain the phase diagram for the transition when varying coupling strength or input amplitude (Figure 1, A). The transition is shifted to significantly larger coupling strengths than predicted by linear stability analysis of the local Jacobian matrix. This difference corresponds to the emergence of a novel dynamical regime, which combines locally expansive dynamics with asymptotic stability.

To study information processing capabilities, we evaluate the capacity to reconstruct a past input signal based on a linear readout of the present state, the so-called memory curve [3]. We find that for a given input amplitude the memory capacity peaks within the novel dynamical regime (Figure 1B). This result indicates that locally expansive while asymptotically stable dynamics is beneficial to store information about the input in the network dynamics.
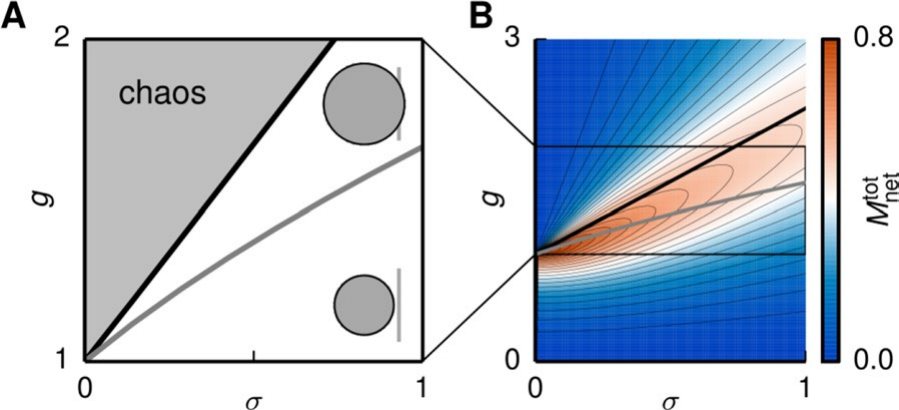


**Figure 1**. **A.** Phase diagram for signal amplitude σ and coupling strength g. Black curve: Phase transition to chaotic regime. Gray curve: condition for loss of local stability. Disk of eigenvalues of the Jacobian matrix in the locally stable (lower) and locally unstable, but asymptotically stable regime (upper). **B.** Network memory capacity encoded in color. Global and local transition curves (black and gray) as in A. Contour lines shown in black

**Acknowledgements**

This work was partially supported by Helmholtz young investigator’s group VHNG-1028, Helmholtz portfolio theme SMHB, Jülich Aachen Research Alliance (JARA). This project received funding from the European Union’s Horizon 2020 research and innovation programme under grant agreement No. 720270. S.G. and J.S. contributed equally to this work.

**References**

1. Sompolinsky H, Crisanti A, and Sommers HJ, Chaos in random neural network. *Phys Rev Lett* 1988, **61**:259.

2. Toyoizumi T and Abbott LF, Beyond the edge of chaos: Amplification and temporal integration by recurrent networks in the chaotic regime. *Phys Rev E* 2011, **84:**051908.

3. Jaeger H, Short term memory in echo state networks. *GMDForschungszentrum Informationstechnik*, 2001, **5**.

## P98 Dynamics of cell assemblies in binary neuronal networks

### Christian Keup^1,2^, Tobias Kühn^1,2^, Moritz Helias^1,2^

#### ^1^Institute of Neuroscience and Medicine (INM-6) and Institute of Advanced Simulation (IAS-6) and JARA, Jülich Research Centre, Jülich, 52425, Germany; ^2^Department of Physics, Faculty I, RWTH Aachen University, Aachen, 52062, Germany

##### **Correspondence:** Christian Keup (c.keup@fz-juelich.de)

*BMC Neuroscience* 2017, **18(Suppl 1)**:P98

Connectivity in local cortical networks is far from random: Not only are reciprocal connections over-represented [1], but there are also larger subgroups of neurons which are stronger connected among each other than to the remainder of the network [2,3]. These observations provide a growing evidence for the existence of neuronal assemblies, that is groups of neurons with stronger and/or more numerous connections between members compared to non-members. To study quantitatively the dynamics of these building blocks, we consider a single assembly of binary neurons embedded in a larger randomly connected EI-network and explore its properties by analytical methods and simulation. Extending [4] to the three population case, we obtain expressions for mean activities, auto- and cross-correlations, and response to input fluctuations using a Gaussian closure. For sufficiently strong assembly self-feedback, this mean-field theory predicts a bifurcation from a mono-stable to a bistable regime. The critical regime around the bifurcation is of interest, as input variations can drive the assembly to high or low activity states and large spontaneous fluctuations are present. These could be a source of neuronal avalanches observed in cortex [5] and the robust response to input could constitute attractor states as in [6]. In this regime however, the approximation is not accurate (Figure 1) due to large fluctuation corrections. We therefore work on a path-integral formulation of such systems built on developments in the application of statistical field theory to neuronal networks [7]. This formulation allows the derivation of an effective potential, a systematic treatment of approximations and the quantification of the response to inputs.
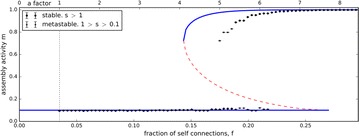


**Figure 1.** Example bifurcation diagram showing stable states of the mean assembly activity (assembly size = 70). Self-feedback is stronger for high values of f. Blue lines are predictions in Gaussian approximation, black dots are simulation results. Approximation is inaccurate in the vicinity of the two critical points

**References**

1. Song S, Sjöström P, Reigl M, Nelson S, Chklovskii DB: Highly Nonrandom Features of Synaptic Connectivity in Local Cortical Circuits. *PloS Biol* 2005, **3(3):**e68.

2. Ko H, Hofer SB, Pichler B, Buchanan KA, Sjöström PJ, Mrsic-Flogel TD: Functional specificity of local synaptic connections in neocortical networks. *Nature* 2011, **473:**87–91.

3. Perin R, Berger TK, Markram H: A synaptic organizing principle for cortical neuronal groups. *PNAS* 2011, **108:**5419–5424.

4. Helias M, Tetzlaff T, Diesmann M: The Correlation Structure of Local Neuronal Networks Intrinsically Results from Recurrent Dynamics. *PLoS Comput Biol* 2014, **10(1):**e1003428.

5. Hahn G, Petermann T, Havenith MN, Yu S, Singer W, Plenz D, Nikolic ´ D: Neuronal Avalanches in Spontaneous Activity In Vivo. *J Neurophysiol* 2010, **104:**3312–3322

6. Cossart R, Aronov D, Yuste R: Attractor dynamics of network UP states in the neocortex. *Nature* 2003, **423:**283–288

7. Schücker J, Goedeke S, Dahmen D, Helias M: Functional methods for disordered neural networks. *arXiv* 2016, **1605**:06758v2

## P99 A dynamic mean-field approach for the largest Lyapunov exponent of random neural networks

### Sven Goedeke^1^, Jannis Schuecker^1^, Moritz Helias^1,2^

#### ^1^Institute of Neuroscience and Medicine (INM-6) and Institute for Advanced Simulation (IAS-6) and JARA BRAIN Institute I, Jülich Research Centre, Jülich, Germany; ^2^Department of Physics, Faculty 1, RWTH Aachen University, Aachen, Germany

##### **Correspondence:** Sven Goedeke (s.goedeke@fz-juelich.de)

*BMC Neuroscience* 2017, **18(Suppl 1)**:P99

Recurrent neural networks often exhibit complex dynamics. It is considered important for information processing in these networks whether the dynamics is chaotic or not. The largest Lyapunov exponent quantifies the rate of exponential divergence or convergence of close-by trajectories and a positive exponent indicates chaos. For networks driven by time-dependent inputs the largest Lyapunov exponent can be used to characterize response reliability. Here, we present a dynamic mean-field approach to estimate the largest Lyapunov exponent of large networks of randomly coupled rate neurons [1,2].

Following an idea by [3,4] we express the average squared Euclidean distance between the trajectories of two identical copies of the network as a combination of time-dependent order parameters, which take the form of empirical averages over the system. The asymptotic growth rate of the Euclidean distance gives the largest Lyapunov exponent. In the limit of large networks the temporal evolution of the order parameters may be approximated using a mean-field approach.

We apply this approach to a continuous-time random neural network model [1], the prototype for a class of recurrent networks, whose dynamical properties and information processing capabilities received a lot of attention during the past years [2]. We derive the dynamic mean-field theory of the joint system and obtain a system of coupled nonlinear partial differential equations for the order parameters. Linearizing these equations about a stationary solution that corresponds to complete synchronization of the two copies results in a linear partial differential equation for the temporal evolution of the average squared Euclidean distance. Separation of variables then yields an eigenvalue problem in form of a time-independent Schrödinger equation. The lowest eigenvalue determines the asymptotic growth rate and, hence, an estimate of the largest Lyapunov exponent.

In addition to providing a mathematically transparent exposition, the method allows answering new questions. In a companion contribution, we analyzed the transition to chaos in continuous-time random neural networks driven by white noise (Schuecker J, Goedeke S, Helias M: Transition to chaos and short-term memory in driven random neural networks. See also [5]).

**Acknowledgements**

This work was partially supported by Helmholtz young investigator’s group VHNG-1028, Helmholtz portfolio theme SMHB, Jülich Aachen Research Alliance (JARA). This project received funding from the European Union’s Horizon 2020 research and innovation programme under grant agreement No. 720270. S.G. and J.S. contributed equally to this work.

**References**

1. Sompolinsky H, Crisanti A, Sommers HJ: Chaos in random neural networks. *Phys Rev Lett* 1988, **61(3)**:259–262.

2. Sussillo D, Abbott LF: Generating coherent patterns of activity from chaotic neural networks. *Neuron* 2009, **63(4):**544–557.

3. Derrida B, Pomeau Y: Random networks of automata: a simple annealed approximation. *Europhys Lett* 1986, **1(2):**45.

4. Cessac B: Increase in complexity in random neural networks. *Journal de Physique I* 1995, **5(3):**409–432.

5. Goedeke S, Schuecker J, Helias M: Noise dynamically suppresses chaos in random neural networks. *arXiv preprint* 2016, arXiv:1603.01880.

## P100 Microdraw: Online platform for the collaborative editing of cytoarchitectonic brain atlases

### Katja Heuer^1^, Rembrandt Bakker^2,3^, Paul Tiesinga^3^, Roberto Toro^4^

#### ^1^Department of Neuropsychology, Max Planck Institute for Human Cognitive and Brain Sciences, Leipzig, Germany; ^2^Neuroinformatics Department, Donders Institute for Brain, Cognition and Behaviour, Radboud University Nijmegen, Nijmegen, The Netherlands; ^3^Institute of Neuroscience and Medicine (INM-6) and Institute for Advanced Simulation (IAS-6) and JARA BRAIN Institute I, Jülich Research Centre, Jülich, Germany; ^4^Applied and Theoretical neuroanatomy group, Institut Pasteur, Paris, France

##### **Correspondence:** Rembrandt Bakker (r.bakker@donders.ru.nl)

*BMC Neuroscience* 2017, **18(Suppl 1)**:P100

Brain atlases parcellate the brain into discrete regions, which form organizational units in many computational models. Many brain atlases are derived from high resolution MR images, but to get access to the cellular organization (cytoarchitecture) of the tissue, atlases based on high resolution microscopy sections are needed. Until now, the complexity and data requirements of the atlas creation process have restricted it to the confounds of specialized laboratories. Here we present an online platform to accelerate this process by allowing it to be done publicly and collaboratively, in a web-based environment.

As the main interface of the platform we created MicroDraw (http://microdraw.pasteur.fr). With this open source application, a user can annotate DeepZoom [1] formatted tissue sections, independent of where these are hosted. It runs in the browser and offers tools to draw vectorial regions, convert polygons to smooth curves, edit/merge/delete shapes, label areas using terms from pre-loaded ontologies, and save the edits to a central database. The use of a standard ontology like the Common Upper Mammalian Brain Ontology [2] promotes the use of the same region names across species. The DeepZoom format stores sections at multiple resolutions and ensures a fast browsing experience, even for ultra-high resolution data.

Microdraw (Fig. 1) is driven by two open source libraries: OpenSeadragon (https://openseadragon.github.io/) for displaying multi-resolution data, and Paper.js (http://paperjs.org/) for vector drawing. Current development efforts focus on easy linking of external DeepZoom repositories, the organization of the central database that stores collaborative output, and integration with BrainBox (http://brainbox.pasteur.fr), a platform for segmenting MRI data.

MicroDraw is developed in the context of project ‘FIIND’, in which it is used to draw atlas regions on brains of ferrets at various stages of development. Ferrets are unique in that they are born with smooth brains, with cortical folding taking place afterwards. The ferret data is due to arrive later this year, currently hosted datasets include Macaque atlases at different postnatal stages [3], and sample sections from BigBrain [4] and other high-resolution atlasing projects.
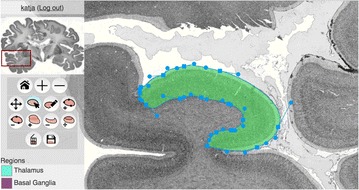


**Figure 1:** Screenshot from the application at http://microdraw.pasteur.fr

**Acknowledgments**

Supported by the FLAG-ERA joint transnational call 2015, financed by the Netherlands Organisation for Scientific Research (NWO) with project number 054-15-104, and by the French National Research Agency.

**References**

1. DeepZoom image specification [https://openseadragon.github.io/examples/tilesource-dzi/]

2. Common Upper Mammalian Brain Ontology [http://neurolex.org/wiki/Cumbo_terms]

3. NIH Blueprint Non-Human Primate Atlas [http://blueprintnhpatlas.org/]

4. Amunts K1, Lepage C, Borgeat L, Mohlberg H, Dickscheid T, et al.: BigBrain: an ultrahigh-resolution 3D human brain model, *Science* 2013, **340(6139):**1472–5. doi: 10.1126/science.1235381.

## P101 Single-compartment models of retinal ganglion cells with different morphologies

### Wei Qin, Alex Hadjinicolaou, Hamish Meffin^2,3^, David B Grayden^1^, Anthony N Burkitt^1^, Michael R Ibbotson^2,3^, Tatiana Kameneva^1^

#### ^1^Department of Biomedical Engineering, University of Melbourne, Melbourne, Victoria 3010, Australia; ^2^National Vision Research Institute, Australian College of Optometry, Melbourne, VIC 3053, Australia; ^3^Department of Optometry and Vision Sciences, University of Melbourne, Melbourne, Victoria 3010, Australia

##### **Correspondence:** Tatiana Kameneva (tkam@unimelb.edu.au)

*BMC Neuroscience* 2017, **18(Suppl 1)**:P101

Retinal prostheses deliver patterns of electrical stimulation through an array of electrodes that generate activity in retinal neurons, which ultimately lead to visual perception by the user. In order to provide the best visual experience, it is important to understand the responses of different types of retinal neurons to electrical stimulation. Previously, we have developed and constrained detailed, morphologically correct models of retinal ganglion cells (RGCs) based on published experimental data, using the same set of constraints for all morphological cell types [1]. Here, we extend this work using our recently published in vitro data for different morphological cell types [2]. To study the effects of electrophysiology on the cells’ responses separately from the effects of morphology, we employed single-compartment models.

Our electrophysiology data show that the resting membrane potential, maximum firing frequency, steady-state firing rate, frequency adaptation index, spike width, and sag amplitude are different for different morphological types. Using these parameters as constraints, we fit conductance values for sodium, potassium, calcium, hyperpolarisation-activated mixed cation, and T-type calcium currents to replicate responses of A1, A2i, A2o, C1, C2i, C2o, C3, C4o, D1, and D2 cells from experimental observations. The model parameters were constrained to within their mean ±1.5 standard deviation. Hodgkin-Huxley single-compartment models were simulated in NEURON using a high-performance computer (VLSCI facilities, approx. 800 CPU-hours). Simulation results were analyzed in MATLAB.

Results show that a single compartment model can replicate behavior of different morphological RGC types. We could replicate behavior of all the cells except C1 and C3 types; for these two cell types, experimental recordings from four and two cells, respectively, indicated only small variability between cells and resulted in very narrow constraints for the model parameters. Different combinations of ionic channels were required in order to reproduce experimentally observable maximum firing frequency, steady-state firing rate, frequency adaptation index, spike width, and sag amplitude for different morphological types. Calcium conductance varied the most between cell types, while potassium and sodium conductances were within a similar range for most of the cells. A1 and C2i cells had the smallest ranges for all conductances but this may be due to small experimentally-observed variabilities for these cells.

This research provides a more comprehensive understanding of retinal responses to electrical stimulation, and may help in the evaluation of the efficacy of different stimulation strategies and thereby accelerate the design of visual prostheses. Future work will incorporate biologically realistic multicompartment models and investigate the combined effect of morphology and electrophysiology on the responses of the RGCs.

**Acknowledgements**

ANB and TK acknowledge support through the Australian Research Council Discovery Projects funding scheme (DP140104533). This research was supported by Melbourne Bioinformatics grant number [VR0138] on its Peak Computing Facility at the University of Melbourne, an initiative of the Victorian Government, Australia

**References**

1. M Maturana, T Kameneva, AN Burkitt, H Meffin, DG Grayden. The effect of morphology upon electrophysiological responses of retinal ganglion cells: simulation results. *Journal of Computational Neuroscience,* 36: 157–175, 2014.

2. AE Hadjinicolaou, SL Cloherty, Y-S Hung, T Kameneva, MR Ibbotson. Frequency responses of rat retinal ganglion cells. *PLoS ONE,* 11(6): e0157676. doi:10.1371/journal.pone.0157676, 2016.

## P102 Credibility, Replicability, Reproducibility in Simulation for research and clinical application

### William W. Lytton^1^, Lealem Mulugeta^2^, Andrew Drach^3^, Jerry G. Myers Jr.^4^, Marc Horner^5^, Rajanikanth Vadigepalli^6^, Tina Morrison^7^, Marlei Walton^8^, Martin Steele^9^, C. Anthony Hunt^10^

#### ^1^Department Of Physiology, SUNY Downstate Medical Center, Brooklyn, NY 11203 USA; ^2^*InSilico* Labs LLC, Houston, TX, USA; ^3^Institute for Computational Engineering and Sciences, University of Texas at Austin, Austin, TX 78712, USA; ^4^John H Glenn Research Center, NASA, Houston, TX, USA; ^5^ANSYS, Inc. Canonsburg, PA 15317, USA; ^6^Thomas Jefferson University, Philadelphia, PA, USA; ^7^U.S. Food and Drug Administration, Washington DC, USA; ^8^KBRWyle, El Segundo, CA 90245, USA; ^9^Kennedy Space Center, NASA, Houston, TX, USA; ^10^Bioengineering and Therapeutic Sciences, University of California, San Francisco, CA, USA

##### **Correspondence:** William W. Lytton (bill.lytton@downstate.edu)

*BMC Neuroscience* 2017, **18(Suppl 1)**:P102

The use of mechanistic multiscale modeling and simulation (M&S) in biomedical research continues to expand, and direct application in healthcare will emerge in the not too distant future. The potential of M&S in personalized and precision medicine will require standardized data provenance and workflows to produce a credible end-product. However, there are currently no standards or guidelines to promote credible practice of M&S in academia or in industry. Lacking such standards, it will be still more difficult to promote these new techniques for direct clinical use or for regulatory approval of devices or clinical software adjuncts. The Committee on Credible Practice of Modeling & Simulation in Healthcare (CPMS) was established under the aegis of the federal Interagency Modeling and Analysis Group (IMAG) and the Multiscale Modeling (MSM) Consortium. The CPMS is developing and adapting guidelines and procedures for credible practice of M&S in conjunction with cultivating consistent terminology, and developing and demonstrating workflows for credible practice. These tasks are made more difficult by the fact that different organs and different experimental and clinical problems require very different kinds of models. In particular, study of the nervous system has a particular focus on electrical phenomenology, which necessitates unique conceptual underpinnings, computational techniques, and scaling levels (*e.g.* dendritic processing) for consideration in multiscale M&S. In addition, study of the nervous system faces a unique challenge of understanding (and modeling) cognitive and motivational processes. Related to this is the fact that experimental data for the nervous system, and the brain in particular, remains very limited compared to levels of experimental data from other organs. Despite all of these differences, it is necessary and desirable that computational neuroscience begin to meet the challenges of credibility through replicability, reproducibility, reliability and provenance, which are beginning to be the expected norm in other branches of computational systems biology. As an initial effort, the CPMS has defined 10 preliminary rules for M&S credibility as follows: 1. Define context clearly; 2. Use appropriate data; 3. Evaluate within context; 4. List limitations explicitly; 5. Use version control; 6. Document adequately; 7. Disseminate broadly; 8. Get independent reviews; 9. Test competing implementations; 10. Conform to standards. Naturally, these guidelines will need to evolve with the further development of M&S practices, especially in the context of medical devices and clinical guidance (*e.g.*, NIH SPARC program efforts, Deep Brain Stimulation, Neuroprosthetics, etc.), where numerous regulatory hurdles, as well as liability considerations, will shape credible M&S practice in the future.

**Acknowledgements**

The authors would like to acknowledge the efforts of Interagency Modeling and Analysis Group and the Multiscale Modeling Consortium, who enabled activities of the Committee.

## P103 Computational social interaction in reciprocity and empathic behavior as behavioral economics and risk tasking behavior

### Nicoladie Tam

#### Department of Biological Sciences, University of North Texas, Denton, TX 76203, USA

##### **Correspondence:** Nicoladie Tam (nicoladie.tam@unt.edu)

*BMC Neuroscience* 2017, **18(Suppl 1)**:P103

**Introduction:** The dynamics of social interaction between two individuals can be a complex behavioral phenomenon. The underlying computational methods for decision-making process can be affected by emotions and social interactions. In order to assess the neurobiological basis of decision making in social interactions, we proposed to use a computational model of the decision-making process based on behavioral economic theories. In particular, we proposed a decision-making model not just based on internal needs of the individual, but based on the factors governing the social interaction that requires reciprocity behavior. The contributing factors governing social reciprocity can result in cooperative or competitive social interactions, depending on whether empathy is used as the criteria for reciprocating such social exchange. It is particularly important to determine how decisions are made in which a conflict occurs. Conflict is defined as the condition in which given two options to choose, the choices are mutually exclusion. That is, choosing one option will nullify the other option in the decision. The most difficult task in the decision-making process is to resolve the conflict (without being forced to choose the better of two evils).

**Methods:** We propose to use the criteria for maximizing the self-gain and minimizing the self-loss as the computational criteria for the social decision-making process in reciprocity. In order to account for empathic behavior where maximizing self-gain does not necessarily impose the condition of maximizing the loss of the other individual, we propose to use the extended-self model [1] to result the conflict in which self-gain becomes other-loss if empathy is not included in the decision-making criteria. The decision is also based on the risk-taking behavior as a gambling task where the outcome is unknown, i.e., a probability rather than certainty.

In order to validate this computational model, we recruited human subjects to play the classical ultimatum game (UG), in which two experimental subjects reciprocate offering each other money to share. The brain activities of the two human subjects were also recorded simultaneously using fNIRS (functional near-infrared spectroscopy) while they perform the reciprocating UG task [2].

**Results:** Using the UG to examine the reciprocity behavior between two human subjects, we are able to determine the underlying neural decision-making process whether the two individuals optimize their self-gain/loss with or without including the other individual as a part of the extended-self. The results show that the priority for optimization is to maximize self-gain and minimize self-loss first before optimizing the extended-self gain/loss. The results also show that the reciprocity process of optimizing the extended-self gain/loss is also dependent on the previous offer by the opponent, whereby the decision for the present trial is dependent on the reciprocity of the previous trial by the opponent.

**Conclusions:** This study shows that we can create a computational model to determine the decision-making process in a social interaction that requires reciprocity based on not just the extended-self model to resolve conflict, but is also dependent on the previous experience of the opponent whether they reciprocate or not. Social decisions are not made in isolation; they involve the reciprocity of the other individuals in addition to the extended-self model of empathy.

**References**

1. Tam ND: EMOTION-III Model: A theoretical framework for social empathic emotions in autonomous control systems. *The Open Cybernetics & Systemics Journal* 2016, **10:**132–146.

2. Tam ND: Hemodynamic responses to emotions and decisions using near-infrared spectroscopy optical imaging. *25th Annual Computational Neuroscience Meeting: CNS*-*2016*, Jeju, South Korea, July 2–7, 2016. Vol. 17, Suppl. 1. pp. 54. DOI: 10.1186/s12868-016-0283-6

## P104 On the need for standardized real-time software technology in closed-loop neuroscience

### Rodrigo Amaducci, Carlos Muñiz, Manuel Reyes-Sánchez, Francisco B. Rodríguez, Pablo Varona

#### Grupo de Neurocomputación Biológica, Dpto. de Ingeniería Informática, Escuela Politécnica Superior, Universidad Autónoma de Madrid, Madrid, Spain

##### **Correspondence:** Pablo Varona (pablo.varona@uam.es)

*BMC Neuroscience* 2017, **18(Suppl 1)**:P104

Real-time software technology is required to implement many instances of closed-loop experiments in neuroscience research. Protocols implemented with such technology are needed when acquisition and stimulation have to meet precise deadlines at the millisecond time scale or lower [1]. Even though modern computers have increasing performance with multiple core architectures, time constraints from event detection to the generation of the associated response cannot be met at the millisecond timescale typically because of operating system (OS) interrupts.

To solve this problem, real-time operating systems (RTOS) and software technology come handy. In the context of electrophysiological closed-loop experiments, a few tools have been developed for this task [2–5]. However, this technology is not easy to install, to program or even to use. In this work, we emphasize the need for standardized user and setup friendly real-time technology in experimental neuroscience and describe current options to simplify the implementation of closed-loop interactions in experiments with hard time constraints.

We illustrate our approach with an example of the scheduling required to solve an inexpensive neuron model aimed to build a hybrid circuit with a living cell. We report benchmarking of this task over: 1) standard OS, 2) standard OS with core isolation, 3) different implementations of RTOS. We implemented a stand-alone Izhikevich neuron model in C, setting the sampling frequency for sending the voltage value to a DAQ board working at 10 kHz on an Intel i7 3.40 GHz multi-core computer.

Measured latencies indicate that fast multiple-core computers with standard OS cannot meet real-time constraints to implement hybrid circuits even with computational inexpensive models. Core isolation improves to some extend the performance but is not enough to implement a closed-loop with hard real-time constraints. The RTOS tested provide the solution with different performance levels but all meeting the required deadlines for physiological use. Core isolation further improved performance in RTOS.

The use of RTOS is difficult for most electrophysiology labs, which prevents the implementation of modern closed-loop protocols requiring hard real-time constraints. We argue that standardization and modularization of RT software beyond specific platforms, including our RTBiomanager, will largely contribute to the use and dissemination of this technology.

**References**

1. Muniz C, Levi R, Benkrid M, Rodriguez FB, Varona P. Real-time control of stepper motors for mechano-sensory stimulation. *J. Neurosci. Methods.* 2008, **172:**105–11.

2. Lin RJ, Bettencourt J, Ite JW, Christini DJ, Butera RJ. Real-time experiment interface for biological control applications. *Conf Proc IEEE Eng Med Biol Soc.* 2010; 2010:4160–3.

3. Muniz C, Rodriguez FB, Varona P. RTBiomanager: a software platform to expand the applications of real-time technology in neuroscience. *BMC Neurosci.* 2009, **10:**P49.

4. Linaro D, Couto J, Giugliano M. Command-line cellular electrophysiology for conventional and real-time closed-loop experiments. *J. Neurosci. Methods.* Elsevier; 2014, **230:**5–19.

5. Biró I, Giugliano M. A reconfigurable visual-programming library for real-time closed-loop cellular electrophysiology. *Front. Neuroinform. Frontiers* 2015; **9:**17.

## P105 Discovering Connectivity Changes with rescaled Energy-Based Models

### Joseph T. Cronin, Matthias H. Hennig

#### Institute for Adaptive and Neural Computation, School of Informatics, The University of Edinburgh, Edinburgh EH8 9AB, United Kingdom

##### **Correspondence:** Joseph T. Cronin (s1474586@sms.ed.ac.uk)

*BMC Neuroscience* 2017, **18(Suppl 1)**:P105

Comparison of neuronal networks between animals with differing genetic mutations, or at distinct periods in time is vital for the study of learning and plasticity in the brain. However, this comparison is often difficult due to the highly noisy and variable nature of recorded neural activity. This is partly due to limitations in recording techniques, but also due to the high number of factors influencing activity in the brain. Energy based models, such as the Ising model, have been shown to successfully capture the pairwise correlation structure and firing pattern distribution of spiking networks [1], and hence are a potentially useful tool for comparisons between networks. Often neural spiking is sparse, leading to a large dominance of the silent, or ‘0’, pattern within a network’s pattern probability distribution. We found that in the Ising model, this sparsity in turn causes the model’s field parameters (i.e. firing rates) to play a far more important role than the interaction (correlation) terms. Hence firing rate changes can strongly affect the analysis of structural changes in the functional network. In this contribution, we explore the potential of renormalising the Ising model by scaling its pattern distribution with that of a simple independent model, in order to extract the captured correlation structure of spiking data that would otherwise be masked by the over fitting of firing rates.

We evaluated the performance of this technique by fitting an Ising model to a set of simulated spike trains generated from Dichotomized Gaussian (DG) spiking models [2]. DG models offer a simple and effective way of altering the dataset’s firing rate and correlation structure, enabling a direct comparison between the Ising model parameters and the simulated spikes. Using an eigenparameter analysis of the model fits [3] and a divergence measure on their pattern probabilities, a number of cases were discovered in which the renormalised Ising model uncovered information about the correlation in the data that was previously masked by the dominant effect of the firing rates (Figure 1.). The rescaled models better described data in which correlation was the only parameter that was altered (firing rates kept fixed). These findings suggest the method has much potential for the analysis of recorded multi-neuron data, which we demonstrate with calcium imaging recordings from the mouse visual cortex.
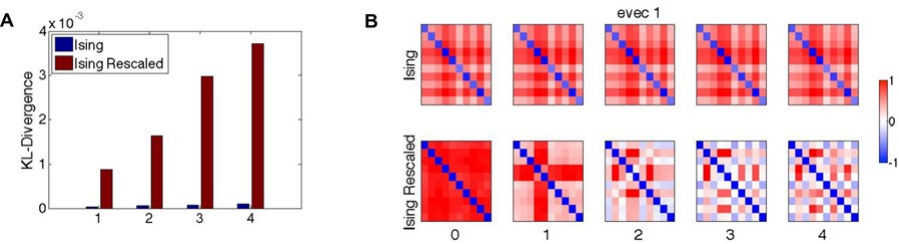


**Figure 1. S**tarting from a homogeneous correlation matrix (N = 10), the structure of the data was increased by selecting one entry at random and increasing it by a fixed amount. **A.** Kullback-Leibler divergence between the baseline distribution and the augmented distributions, for both the Ising and rescaled Ising models. The rescaled Ising model is clearly far more sensitive to this change than the original model. **B.** First eigenvectors of the Fisher information matrix for the corresponding models. The rescaled model eigenvectors reflect changes in the correlation structure while the original Ising model eigenvectors are indistinguishable from one other

**References**

1. Schneidmann GTE, Berry MJ, Segev R, Bialek W: Weak pairwise correlations imply strongly correlated network states. *Nature* 2006, **440:**1007–1012.

2. Macke JH, Berens P, Ecker AS, Tolias AS, Bethge M: Generating spike trains with specified correlation coefficients. *Neural Computation* 2009, **21(2):**397–423.

3. Panas D, Amin H, Maccione A, Muthmann O, van Rossum M, Berdondini L, Hennig MH: Sloppiness in spontaneously active neural networks. *J Neurosci* 2015, **35(22):**8480–8492.

## P106 Fitting and analysis pipeline to build data-driven models of tonic and burst firing in thalamic neurons

### Elisabetta Iavarone^1^, Christian O’Reilly^1^, Jane Yi^2^, Ying Shi^2^, Bas-Jan Zandt^1^, Werner Van Geit^3^, Christian Rössert^3^, Henry Markram^2,3^, Sean Hill^1^

#### ^1^Laboratory for the Neural Basis of Brain States, Blue Brain Project, EPFL, Geneva, GE, 1202, Switzerland; ^2^Laboratory of Neural Microcircuity, Brain Mind Institute, EPFL, Lausanne, GE, 1202, Switzerland; ^3^Blue Brain Project, EPFL, Geneva, GE, 1202, Switzerland

##### **Correspondence:** Elisabetta Iavarone (elisabetta.iavarone@epfl.ch)

*BMC Neuroscience* 2017, **18(Suppl 1)**:P106

The thalamo-cortical system constitutes a large part of the mammalian brain, comprising different thalamic nuclei and their reciprocal interactions with the neocortex. They are involved in numerous functions, such as gating of sensory information, regulating states of sleep and wakefulness, consciousness and awareness. Understanding the neural systems’ structural and functional complexity is greatly facilitated by computer simulations. Data-driven modeling pipelines have shown how sparse biological data can be used to reconstruct dense maps of neural circuits, to integrate different sources of neuroscientific data and to predict new experimental findings. These principles have been recently published in the context of the Blue Brain Project as a detailed reconstruction and simulation of a cortical microcircuit [1]. We propose to extend this approach with a model of the thalamus.

We characterized the properties of thalamic neurons (i.e., thalamo-cortical relay cells and reticular neurons) through 3D morphological reconstructions and standardized electrophysiological protocols. Features characterizing the two main firing regimes of thalamic cells (i.e. tonic firing and bursting) have been extracted from electrophysiological recordings and used to constrain the parameters of the model. The kinetics and distribution of ionic currents have been defined after systematic curation of literature in combination with an on-line encyclopedia which links multiple data and modeling resources [2]. The other free parameters, namely the maximal conductances of the ionic currents and the rate of removal of the intracellular calcium, were constrained by applying a multi-objective optimization strategy [3]. We further refined the models and the constraints by exploring the sensitivity of the responses of the model to small changes in the parameters value. We tested the generalization of the resulting models with stimuli not used during the fitting procedure, along with different morphologies that were repaired for slicing artifacts [4]. After validating the models and ensuring their robustness, they have been integrated in a large-scale digital reconstruction of thalamic microcircuitry.

Our model building, validation and analysis pipeline makes the process of creating and simulating data-driven neuron models easily reproducible and adaptable. New data can be readily integrated while biological parameters value can be predicted in order to iteratively refine the models’ reconstruction and simulation cycle.

**References**

1. Markram H, Muller E, Ramaswamy S, Reimann MW, Abdellah M, Sanchez CA, Ailamaki A, Alonso-Nanclares L, Antille N, Arsever S, et al.: Reconstruction and simulation of neocortical microcircuitry. *Cell* 2015 **163.2**: 456–492.

2. KnowledgeSpace [https://knowledge-space.org]

3. Van Geit W, Gevaert M, Chindemi G, Rössert C, Courcol JD, Muller E, Schürmann F, Segev I, Markram, H: BluePyOpt: Leveraging open source software and cloud infrastructure to optimise model parameters in neuroscience. *Front in Neuroinform* 2016, **10**

4. Anwar H, Riachi I, Hill S, Schurmann F, Markram H: An approach to capturing neuron morphological diversity. In Computational Modeling Methods for Neuroscientists. *The MIT Press* 2009, 211–231.

## P107 A data-driven pipeline for digital reconstruction of somatosensory thalamic microcircuitry

### Christian O’Reilly^1^, Elisabetta Iavarone^1^, Jane Yi^2^, Ying Shi^2^, Rodrigo Perin^2^, Huanxiang Lu^1^, Bas-Jan Zandt^1^, Henry Markram^2^, Sean Hill^1^

#### ^1^Laboratory for the Neural Basis of Brain States, Blue Brain Project, EPFL, Geneva, GE, 1202, Switzerland; ^2^Laboratory of Neural Microcircuity, Brain Mind Institute, EPFL, Lausanne, GE, 1202, Switzerland

##### **Correspondence:** Christian O’Reilly (christian.oreilly@epfl.ch)

*BMC Neuroscience* 2017, **18(Suppl 1)**:P107

The thalamo-cortical system constitutes an important part of the mammalian brain and regulates several functions, such as transition between sleep and wakefulness and gating of sensory information. Understanding this kind of complex neural system can be greatly facilitated by data-driven computer simulations. With this aim in mind, the Blue Brain Project has developed a pipeline to build and simulate large-scale biophysically detailed models of brain regions. Using the rat somatosensory microcircuit, it showed how sparse biological data can be used to reconstruct dense maps of neural circuits and predict new experimental findings [1]. This pipeline is now being extended to model a thalamic counterpart, including the ventral posterolateral (VPL), ventral posteromedial (VPM), and reticular (Rt) nuclei of the thalamus. It includes principally the following steps: optimization of single-cell models, repair and cloning of reconstructed morphologies, selection and placement of morphological/electrophysiological single-cell models in the 3D space of the thalamic nuclei (as specified in the Paxinos and Watson atlas [2] rendered in 3D using 3DBAR [3] and further processed using a hierarchical smoothing approach), modeling of synaptic contacts, and simulation. For this project, we used a comprehensive battery of stimuli to characterize the electrophysiology of tonic firing (34 thalamo-cortical (TC) cells; 2 reticular cells; see Figure 1B) and burst firing (49 TC cells; 7 reticular cells; see Figure 1C). We also reconstructed in 3D the morphology of 40 biocytin-filled TC cells (22 in VPL; 18 in VPM; see Figure 1A) and obtained 7 reconstructions of reticular cells (courtesy of Didier Pinault). This proof-of-concept allowed us to validate the technical aspects of the pipeline and to get preliminary visualization of the circuit (see Figure 1F). We are actively integrating new experimental data and systematically curated information from the literature. We are also refining the different steps of the pipeline to take into account differences in the modeling requirements compared to previous cortical modeling (e.g., modeling irregular 3D volumes of thalamic nuclei instead of stacking layered cortical columns). In future work, we will continue to refine this pipeline to study in silico thalamic functions and dysfunctions, as well as thalamo-cortical interactions.
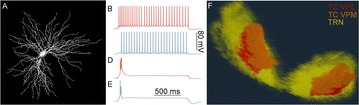


**Figure 1.** Example of a VPL TC cell morphology (**A**) and patch-clamping recording in slice for tonic (**B**) and burst (**D**) firing. Corresponding single-cell simulated tonic (**C**) and burst (**E**) firing. Reconstructed circuit (**F**)

**Acknowledgements**

The authors gratefully acknowledge the contribution of the reticular cell morphologies by Didier Pinault. They are also grateful to the various people in the Blue Brain Project whose technical support was invaluable.

**References**

1. Markram H, Muller E, Ramaswamy S, Reimann MW, Abdellah M, Sanchez CA, Ailamaki A, et al.: Reconstruction and Simulation of Neocortical Microcircuitry. *Cell* 2015, **163:**456–492.

2. Paxinos G, Watson C: *The Rat Brain in Stereotaxic Coordinates, 6th Ed*. Amsterdam: Elsevier Academic; 2007.

3. Majka P, Kublik E, Furga G, Wójcik DK: Common atlas format and 3D brain atlas reconstructor: infrastructure for constructing 3D brain atlases. *Neuroinformatics* 2012, **10(2)**, 181–197.

## P108 Sensitivity analysis of somatic and dendritic features, parameters, and fitting error of biophysically detailed neuron models

### Bas-Jan Zandt^1^, Elisabetta Iavarone^1^, Alexander Bryson^2^, Werner van Geit^1^, Christian O’Reilly^1^, Christian Rössert^1^, Sean Hill^1^

#### ^1^Blue Brain Project, École Polytechnique Fédérale de Lausanne, Geneva, GE, 1202, Switzerland; ^2^Florey Institute of Neuroscience & Mental Health, University of Melbourne, Melbourne, VIC, 3000, Australia

##### **Correspondence:** Bas-Jan Zandt (bas-jan.zandt@epfl.ch)

*BMC Neuroscience* 2017, **18(Suppl 1)**:P108

Biophysically detailed models of neurons can greatly aid investigations of single cell function, and can serve as building blocks for mesoscopic simulations of brain areas. These models simulate membrane voltage dynamics on detailed morphologies containing a range of voltage-gated currents. In the model generation pipeline of the Blue Brain Project [1], parameters such as the maximum conductances of ionic currents (g_max_) are optimized by model fitting to reproduce electrophysiological features from patch clamp data. We add to this pipeline a systematic analysis of the dependency of a model’s behavior on its parameters g_max_. The goal of this is two-fold. First, we are interested in the functional role of ionic currents and how they contribute to the neuronal dynamics. Second, we aim to improve our fitting procedure.

A pyramidal cell model [2] from rat cortex was used, containing a set of active currents (transient and persistent Na^+^ and K^+^, I_h_, I_m_, K_v_3.1, low and high voltage-activated Ca^2+^, and Ca-activated K^+^), with an optimized parameter set previously obtained from multi-objective optimization using ~ 25 features characterizing amongst others the firing rates and shape of action potentials at the soma and dendrites. Python and the BluePyOpt toolbox [3] were used to set up the model and calculate features. Dependency of the features on the currents was investigated in two ways: First, the model was simulated and features calculated with each of the currents knocked out (g_max_ set to zero). Second, the derivatives of the features with respect to each g_max_ was calculated. The resulting matrix of derivatives (Jacobian), was then used to create hierarchically clustered trees for both the currents and features, showing which currents have similar effects on the features and which features have similar dependencies (figure 1A). Furthermore, we quantified how well parameter combinations are constrained when fitting to the selected features, by investigating the local error landscape using theory from least-squares optimization [4] (figure 1B).

We will present the implications our results have for the regulation of neuronal function, and model optimization.
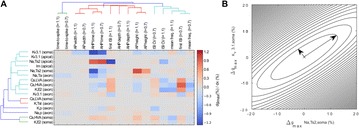


**Figure 1.** Preliminary results. **A.** Dependency of features (subset) on g_max_ of currents. **B.** Contourplot of fitting error for two selected parameters, arrows denote axes of paraboloid. Note that it allows covariation of the two parameters

**References**

1. Markram H, Muller E, Ramaswamy S, Reimann MW, Abdellah M, Sanchez CA, Ailamaki A, Alonso-Nanclares L, Antille N, Arsever S, et al.: Reconstruction and Simulation of Neocortical Microcircuitry. *Cell* 2015, **163:**456–492.

2. Hay E., Hill S., Schürmann F., Markram H., Segev I.: Models of neocortical layer 5b pyramidal cells capturing a wide range of dendritic and perisomatic active properties. *PLoS Comput Biol* 7.7 (2011): e1002107

3. Van Geit W, Gevaert M, Chindemi G, Rössert C, Courcol JD, Muller E, Schürmann F, Segev I, Markram H: BluePyOpt: Leveraging open source software and cloud infrastructure to optimise model parameters in neuroscience. *Frontiers in neuroinformatics,* 10 (2016).

4. Wright S, Nocedal J: Numerical optimization. Ch.10, Springer Science 35 (1999): 67–68.

## P109 Music gone crazy: neural oscillators to the rescue?

### Michal Hadrava^1,2,3^, Jaroslav Hlinka^2,3^

#### ^1^Department of Cybernetics, Faculty of Electrical Engineering, Czech Technical University in Prague, Prague, 166 27, Czech Republic; ^2^Department of Nonlinear Dynamics and Complex Systems, Institute of Computer Science, The Czech Academy of Sciences, Prague, 182 07, Czech Republic; ^3^National Institute of Mental Health, Klecany, 250 67, Czech Republic

##### **Correspondence:** Michal Hadrava (hadrava@cs.cas.cz)

*BMC Neuroscience* 2017, **18(Suppl 1)**:P109

Why is it so that the tickets for the Bayreuth Festival at which performances of Richard Wagner’s operas are presented annually are sold out years in advance? Wagner enthusiasts may give us a clue: when asked to describe Wagner’s music, they usually start being very emotional and such lofty descriptors as “eternal longing” are commonplace. Interestingly, both musicological and music-psychological evidence converge on the theory that the emotional power of music stems from its sophisticated play with our expectancies [1]. The extent to which a pitch/chord is “longed for” in the context of a musical sequence is closely related to its perceived ``stability” and to how much it apparently “attracts” other pitches/chords in the sequence (see [2] and the references thereof). The phenomenon wherein more stable pitches attract the less stable ones is called tonality.

It was knowledge of the laws of attraction between pitches/chords codified in musical textbooks that for centuries enabled composers to move the emotions of their audience. Surprisingly, shortly after the turn of the 20^th^ century, some of them eschewed these ``tame” musical materials and started exploring the uncharted territory of noises and, later, electronic sounds. In the second decade of the 21^st^ century, we still don’t have a textbook on tonality of general sounds. However, we might be close to having a computational model of it.

In [3], the authors derived a canonical model for networks of Wilson-Cowan-type neural oscillators. Later, a simplified model of a network of two symmetrically coupled canonical oscillators was used to predict ratings of tonal stability of pitches in both Western [4] and non-Western [5] scales with impressive results. Importantly, due to its neurodynamical origin, the model has no built-in assumptions on its input. However, there were some notable discrepancies between the model predictions and the data. As suggested by the authors in [5] based on results of a numerical simulation with the full model, these might be eliminated by considering three or more oscillators instead of just two. Consequently, we aim to derive a reduced model of a network of an arbitrary number of canonical oscillators, compute its bifurcation diagram, and, based on the latter, predict tonal stability.

We have developed a semi-analytical procedure for computing an approximate bifurcation diagram of a network of canonical neural oscillators. The procedure relies on a modified algorithm for computing the basis of non-negative solutions to a linear inhomogeneous Diophantine equation. We are currently surveying various algorithms amenable to such a modification.

**Acknowledgements**

This work was supported by the Grant Agency of the Czech Technical University in Prague, grant No. SGS17/133/OHK3/2T/13, and the Czech Health Research Council, project No. NV15-29835A.

**References**

1. Huron D: *Sweet Anticipation: Music and the Psychology of Expectation.* Cambridge: The MIT Press; 2006.

Abbott LF, van Vreeswijk C: Asynchronous states in networks of pulse-coupled oscillators. *Phys Rev E* 1993, **48:**1483–1490.

2. Woolhouse M: Modelling tonal attraction between adjacent musical elements. *J New Music Res* 2009, **38(4):** 357–379.

3. Large EW, Almonte FV, Velasco MJ: A canonical model for gradient frequency neural networks. *Physica D* 2010, **239(12):**905–911.

4. Large EW: A dynamical systems approach to musical tonality. In Huys R, Jirsa VK (Eds.): *Studies in Computational Intelligence: Nonlinear Dynamics in Human Behavior*, **328:**193–211, Berlin Heidelberg: Springer-Verlag; 2011.

5. Large EW, Kim JC, Flaig NK, Bharucha JJ, Krumhansl CL: A neurodynamic account of musical tonality. *Music Percept* 2016, **33(3):**319–331.

## P110 Bistability generates highly irregular spike trains with weakly fluctuated inputs

### Ryosuke Hosaka

#### Department of Applied Mathematics, Fukuoka University, Fukuoka 814-0180, Japan

##### **Correspondence:** Ryosuke Hosaka (hosaka@fukuoka-u.ac.jp)

*BMC Neuroscience* 2017, **18(Suppl 1)**:P110

Regarding the response to the fluctuated inputs, a strange phenomenon has been reported: “The variability of output spike trains of the Hodgkin-Huxley (HH) neuron model decreases as the input variance increases.” This inverse relationship between input and output variances is seemingly counterintuitive. The underlying mechanism of the strange responses of HH neurons may originate from the subthreshold oscillations of the membrane potential. In fact, the input-output (I-O) relationship of a leaky integrate-and-fire (LIF) neuron model, which does not contain subthreshold oscillations, is proportional.

Although the findings were important and fundamental, further analyses are required because the comparison was performed with models with dynamics that were quite different from each other. The HH and LIF models differ in a number of ways, including the complexity of their dynamics, the number of variables, and the number of parameters. Moreover, the HH model is too complicated to use to determine the origin of the strange responses. Therefore, we have been unable to verify that subthreshold oscillations are the origin of the strange responses. In addition, other components may cause or contribute to the strange responses. Thus, the purpose of this study was to reveal the origin of the strange responses.

To examine the origins of the strange responses, a neuron model that can separate the subthreshold oscillations and the bistability should be used. In the current study, we employed the Hindmarsh-Rose (HR) neuron model [1]. The HR model is a neuron model that is described by only two variables, and it has fewer parameters than the HH model. The HR model exhibits both subthreshold oscillations and bistability by controlling only two parameters.

First, we demonstrated that the HR model separated the subthreshold oscillations and bistability and that the origin of the strange responses was the bistability and not the subthreshold oscillations. We then found that the same results were obtained with map-based models. A map-based model that contained bistability reproduced the strange responses, while the map-based model that did not contain bistability did not. These results were further supported by the findings that the strange responses were reproduced by a simple mixture of two interspike interval (ISI) distributions.

**Reference**

1. Hindmarsh JL and Rose RM: A model of the nerve impulse using two first-order differential equations. *Nature* 1982, **296:**162–164.

## P111 Big bang bifurcations structure the parameter space of a two-cell inhibitory network with synaptic depression

### Mark Olenik^1^, Conor Houghton^2^

#### ^1^School of Biological Sciences, University of Bristol, Bristol, BS81TQ, UK; ^2^Department of Computer Science, University of Bristol, Bristol, BS81UB, UK

##### **Correspondence:** Mark Olenik (m.olenik@bristol.com)

*BMC Neuroscience* 2017, **18(Suppl 1)**:P111

A network of two spiking neurons mutually coupled through synaptic inhibition with short term depression can produce stable periodic anti-phase burst patterns. Numerical simulations of the network show that variations of critical synaptic parameters such as the coupling strength and the synaptic time constants can cause increasingly longer burst solutions via a series of period adding bifurcations [1]. To understand the period adding dynamics, we apply geometric singular perturbation methods and reduce the network of two neurons to a scalar map with a discontinuity border. Investigations of the map show that the period adding bifurcations are caused by co-dimension one border collision bifurcations at which period-n points coalesce with a discontinuity border thereby incrementing the period [2]. To investigate the occurrence of multi-parametric bifurcations, we further derive equations for these period-n points at the border collisions as functions of the critical parameters. We demonstrate the existence of focal points in the multidimensional parameter space at which an infinite number of bifurcation curves emerge, each curve corresponding to a border collision. These co-dimension two big bang bifurcations determine the structure of the parameter space and provide an intuition on the parameter dependence of the full two cell network.

**References**

1. Avrutin V, and Schanz. M: On multi-parametric bifurcations in a scalar piecewise-linear map. *Nonlinearity* 2006 **19(3)**: 531.

2. Bose A, and Booth V: Co-existent activity patterns in inhibitory neuronal networks with short-term synaptic depression. *Journal of theoretical biology* 2011 **272(1)**: 42–54.

## P112 Morphology and balance influences Dendritic mosaic formation

### Nicolangelo Iannella^1^, Thomas Launey^2^

#### ^1^School of Mathematical Sciences, University of Nottingham, Nottingham, NG7 2RD, UK; ^2^Lab for Synaptic Molecules of Memory Persistence, RIKEN, Brain Science Institute, 2-1 Hirosawa Wakoshi, Saitama 351-0198, Japan

##### **Correspondence:** Nicolangelo Iannella (nicolangelo.iannella@nottingham.ac.uk)

*BMC Neuroscience* 2017, **18(Suppl 1)**:P112

Neurons can simultaneously adapt their information processing capabilities, including their input and output characteristics. Their ability for such changes ultimately relies on understanding how the interplay between synaptic plasticity, the location of the synapse, and the nonlinear nature of electrical conduction of the dendrite’s membrane shapes both the strengths and spatial arrangements of convergent afferent inputs to neuronal dendrites. Recent studies support the formation of memory traces or engrams via the clustered plasticity model, a view that synaptic plasticity promotes the formation of *clusters* or hotspots of functional synapses [1–3]. Our previous studies have illustrated that spike timing-dependent plasticity (STDP) can lead to synaptic efficacies being arranged into spatially segregated clusters across the dendrite, which we have called a dendritic mosaic [4,5]. We have found that the formation and refinement of the dendritic mosaic can be influenced by both the balance between the amount of depression and potentiation admitted by the temporal learning window describing STDP, and recently dendritic morphology also has a role to play [6,7].

Here, using a biophysically detailed neuron model of a layer 2/3 cortical cell, we have shown that not only does STDP balance affects the formation and patterning of the dendritic mosaic, but also the morphology of the dendrite. We find that both dendritic morphology and STDP balance has an important role to play for this emergent mode of spatial organization, where altering the degree of balance or the shape of the dendrite leads to corresponding changes in the occurrence and patterning of efficacy clusters. Our model suggests that, over a broad range of STDP parameters, synaptic plasticity shapes the spatial arrangement of synapses in a fashion favouring the formation of clustered efficacy engrams, however the emergence of this spatial organization is also influenced by changes in dendritic morphology [6,7]. These findings suggest that under favourable conditions the branching patterns of dendrites, along with the processes that underlie synaptic plasticity, seems to permit a subdivision of dendritic space to occur at the level of dendritic branches and thus allowing a way to form near independent functional units that behave as responsive zones that contribute to neuronal responses as a function of input. This subdivision supports the hypothesis that spatially distributed storage of information is the preferred outcome of synaptic plasticity, but such subdivision of dendritic space is subjective in nature, appearing to be conditionally dependent function of the shape of the dendrite.

**Acknowledgements**

N. Iannella was supported by the People Programme (Marie Curie Actions) of the European Union Seventh Framework Programme (FP7/2007-2013) under REA grant agreement No PCOFUND-GA-2012-600181. The authors would like to thank the support of the RIKEN, Brain Science Institute and the Advanced Center for Computing and Communication (ACCC) for their supercomputing facilities.

**References**

1. De Roo M, Klauser P, and Muller D, LTP promotes a selective long-term stabilization and clustering of dendritic spines, *PLoS Biol* 2008, **6**, pp. e219:1850–1860.

2. Losonczy A, Makara JK, and Magee JC, Compartmentalized dendritic plasticity and input feature storage in neurons, *Nature* 2008, **452(7186)**, 436–441.

3. Govindarajan A, Kelleher R, and Tonegawa S. A clustered plasticity model of long-term memory engrams, *Nat Rev Neurosci* 2006, **7**:575–583.

4. Iannella N & Tanaka S: Synaptic efficacy cluster formation across the dendrite via STDP, *Neurosci Lett* 2006, **403**, 24–29.

5. Iannella N, Launey T, & Tanaka S: Spike timing-dependent plasticity as the origin of the formation of clustered synaptic efficacy engrams, *Front Comput Neurosci* 2010, **4:** 455.

6. Iannella N, Launey T: Modulating STDP balance impacts the dendritic mosaic, (under review)

7. Iannella N, Launey T: Synaptic Efficacy Mosaics and the Impact of Morphology, IEEE *IJCNN* 2017, (accepted)

## P113 Comparison between extracellular and intracellular stimulation

### Tatiana Kameneva^1^, Rebecca Kotsakidis^2^, Hamish Meffin^2,3^, Michael R Ibbotson^2,3^

#### ^1^Department of Biomedical Engineering, University of Melbourne, Melbourne, Victoria 3010, Australia; ^2^National Vision Research Institute, Australian College of Optometry, Melbourne, VIC 3053, Australia; ^3^Department of Optometry and Vision Sciences, University of Melbourne, Melbourne, Victoria 3010, Australia

##### **Correspondence:** Tatiana Kameneva (tkam@unimelb.edu.au)

*BMC Neuroscience* 2017, **18(Suppl 1)**:P113

Visual prostheses (bionic eyes) have been shown to restore rudimentary vision to people suffering from Retinitis Pigmentosa (RP) and Age-Related Macular Degeneration (AMD). RP and AMD are debilitating diseases that cause degeneration of photoreceptors in the retina. Retinal ganglion cells (RGCs) are the output neurons of the retina that send information to the visual brain via the optic nerve. Large proportions (~30%) of RGCs survive in people with RP and AMD. The challenge presented in recent research is how to best utilise residual ganglion cells to restore functional vision to patients suffering from retinal degeneration. Experiments conducted in vitro make it possible to test various stimulation strategies with less experimental commitment than in vivo experiments or clinical studies. It is often assumed that results obtained in vitro (using intracellular stimulation) directly translate to the in vivo setting and the clinic (where extracellular stimulation is used). However, no comprehensive study has been done to date that compares responses to intracellular and extracellular stimulation in the same cells and analyzes the respective underlying mechanisms that lead to cell activity.

**Experiments:** The retinal tissue from mice was mounted onto a 2.2 × 4 cm stage, held down by magnets and perfused with extracellular AMES medium at a flow of 3–5 ml/min. An extracellular stimulating electrode was placed above the inner limiting membrane approximately 100–200 μm from the cell being patched. The intracellular patch electrode administered stimulation through the internal solution to the pipette patched to the cell. Neuronal activity was collected using National Instruments LabView software and recorded with software created in MATLAB. Stimulation protocols were delivered in current clamp mode via an amplifier. The stimulation protocol consisted of sinusoidal waveforms of varying frequency (range: 0–2048 Hz) and amplitude (range: 1–256 uA for extracellular and 1–640 pA for intracellular cells). In total, 26 cells were stimulated with the extracellular electrode, 29 with the intracellular, and 19 cells were stimulated using both the intracellular and extracellular electrodes. All protocols followed ethical guidelines according to the National Health and Medical Research Council of Australia (NHMRC).

**Simulations:** Detailed morphologically correct models of retinal ganglion cells (RGCs) were simulated in the Neuron environment. Neural dynamics were described using Hodgkin-Huxley type equations. Previously constrained model parameters were used to describe ionic currents. Cell dynamics in response to extracellular and intracellular stimulation were compared and analyzed in MATLAB. The stimulation protocol consisted of sinusoidal waveforms of varying frequency and amplitude (frequency range: [0–2048] Hz; amplitude range for intracellular stimulation: 0–5 pA; for extracellular stimulation: 0–10 uA).

**Experiments and simulations:** Experimental results and simulations show that cells respond to higher frequency when extracellular stimulation is applied compared to intracellular stimulation, where no responses occurred. Simulations show that the cell membrane acts as a low-pass filter, explaining the difference between the responses to extracellular and intracellular stimulation. The power spectrum of the membrane potential signal in the soma detects high-frequency components, which is not the case for the signal recorded in the axon. In addition, not all action potentials recorded in the soma propagate into the axon. The maximum amplitude of the membrane potential and the number of spikes are lower in the axon than in the soma.

This work provides evidence that neural responses to intracellular stimulation fundamentally differ from the responses of neurons to extracellular stimulation. In particular, the optimal stimulation frequency required to elicit maximum responses is different for intracellular and extracellular stimulation. One has to be cautious when translating results obtained with intracellular stimulation to those that employ extracellular stimulation.

**Acknowledgements**

TK acknowledges support through the Australian Research Council Discovery Projects funding scheme (DP140104533). HM and MI acknowledge support from the Centre of Excellence for Integrative Brain Function (CE140100007).

## P114 Bifurcations in a temperature-dependent neural mass model reveal heterogeneous effect of focal cooling on epileptic discharges

### Jaymar Soriano^1,2^, Takatomi Kubo^1^, Takao Inoue^3^, Hiroyuki Kida^3^, Toshitaka Yamakawa^4^, Michiyasu Suzuki^3^, Kazushi Ikeda^1^

#### ^1^Nara Institute of Science and Technology, Nara, Japan; ^2^University of the Philippines - Diliman, Quezon City, Philippines; ^3^Yamaguchi University, Ube, Japan; ^4^Kumamoto University, Kumamoto, Japan

##### **Correspondence:** Takatomi Kubo (takatomi-k@is.naist.jp)

*BMC Neuroscience* 2017, **18(Suppl 1)**:P114

Experiments with anaesthetized mice have demonstrated that focal cooling with a temperature of 15 ^O^C can terminate or suppress epileptic discharges (Fig. 1A) [1], however, a mechanism of how cooling does this is still not clear. We formulated temperature dependence in a model for epileptic discharges based from experimental findings from in vitro and in vivo cooling of animal models of epilepsy. The proposed model involves temperature factors integrated into the synaptic and intrinsic firing processes in a neural mass model and is able to simulate termination or suppression of epileptic discharges based on the values of the temperature coefficients Q_10,gain_ and Q_10,sig_, respectively [2]. Fixing Q_10,gain_ at 1.8 with cooling temperature of 15^O^C, we find that the magnitude of simulated discharge activity exhibit bifurcation with respect to Q_10,sig_ (Fig. 1B squares) including a transition from terminated to suppressed discharge activity (Fig. 1B inset(i)). Surprisingly, we also find that cooling can result to seizure activity (Fig. 1B inset(ii)) when Q_10,sig_ is inside a bistable region. To gain insight on these observations, we performed a numerical continuation of a noiseless version of the model (Fig. 1B solid line). A Hopf bifurcation point is found inside a bistable region confirming the existence of low-amplitude high-frequency oscillations at intermediate values of Q_10,sig_ indicating possible seizure initiation. A limit point of cycles is also found which marks the transition of possible seizure activity back to stationary activity, indicating termination of discharge activity, until the end of the bistable region at which point the activity transitions back to limit cycles, indicating suppression of discharge activity. The bistable region vanishes at weaker cooling temperatures (25 °C above) suggesting that such possibility of seizure during cooling may be prevented.
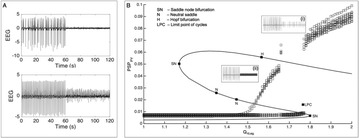


**Figure 1. A.** EEG before (0–60 s) and during 15 °C cooling (60–120 s) showing termination (top) and suppression (bottom) of epileptic discharges. **B.** Numerical continuation of the noiseless version of the model gives insight about observed bifurcation pattern of discharge activity magnitude (squares) with respect to Q_10,sig_ (Q_10,gain_ is fixed at 1.8) with 15 °C cooling. Insets show (**i**) suppressed discharges and (**ii**) seizure activity during cooling. Discharge activity before cooling is shown for reference

**Acknowledgements**

This study was supported in part by Grant-in-Aid for Scientific Research(S) Grant 496 Number 15H05719.

**References**

1. Fujii M, Inoue T, Nomura S, Maruta Y, He Y, Koizumi H, Shirao S, Owada Y, Kunitsugu I, Yamakawa T, Tokiwa T: Cooling of the epileptic focus suppresses seizures with minimal influence on neurologic functions. *Epilepsia* 2012, Mar 1;53(3):485–93.

2. Soriano J, Kubo T, Inoue T, Kida H, Yamakawa T, Suzuki M, Ikeda K: A temperature-dependent neural mass model for suppression of epileptic discharges. Program No. 407.03. 2016 Neuroscience Meeting Planner. San Diego, CA: Society for Neuroscience, 2016. Online.

## P115 Robust Transmission of Rate Coding in the Inhibitory Purkinje Cell to Cerebellar Nuclei Pathway in Awake Mice

### Samira Abbasi^2^, Amber E. Hudson^1^, Detlef H. Heck^3^, Dieter Jaeger^1^

#### ^1^Department Biology, Emory University, Atlanta, GA 30033, USA; ^2^Department of Biomedical Engineering, Hamedan University of Technology, Hamedan, 65169-13733, Iran; ^3^Department of Anatomy and Neurobiology, UT Health Science Center, Memphis, TN 38163, USA

##### **Correspondence:** Dieter Jaeger (djaeger@emory.edu)

*BMC Neuroscience* 2017, **18(Suppl 1)**:P115

An important question in Neuroscience is how populations of neurons in the brain transmit information between each other. From the single neuron perspective, this question becomes how to pick out specific information from hundreds or thousands of synaptic inputs that impinge on it each second in order to generate a single output spike train. In the answer to this question often temporal codes such as coincidence detection are contrasted with rate codes, where the output firing rate smoothly codes net changes in excitatory or inhibitory input conductance. We explored this question for the inhibitory connection between cerebellar Purkinje cells and the cerebellar nuclei. This connection provides the only output from the cerebellar cortex, which is widely thought to process fine temporal information in adaptive motor control.

Our modeling approach relied on a data set of extracellular recordings from Purkinje cells (PC) anterior vermis and cerebellar nuclei (CN) neurons in the medial nucleus in awake mice. In these structures, respiratory activity is coded through spike rate modulation [1]. Indeed, we found that in our data set the majority of PC and CN recordings showed respiratory modulation, but that the phase of modulation with respect to the respiratory cycle was highly variable. In order to simulate how respiratory coding in PCs may be transmitted to the CN we used a biophysically realistic full morphological model of a CN neuron [2]. We created artificial PC spike trains (AST) that matched the spike train statistics of recorded Purkinje cells and allowed us to manipulate the amount of respiratory co-modulation and other not behaviorally linked rate covariance shared between 50 PC ASTs that were used as inhibitory input to our model. Excitatory input was taken from a mossy fiber recording in awake mice, which did not exhibit respiratory modulation.

We found that our model could fully replicate both the baseline statistics of recorded CN spike trains from awake mice, and also the respiratory modulation seen in the CN (Fig. 1). Importantly this outcome could only be achieved if a significant proportion of PC inputs to the CN model shared the same phase of respiratory modulation, and also shared a significant amount of baseline rate changes. This result leads to the prediction that synaptic plasticity of PC inputs in the CN must functionally strengthen connections that show such rate co-variance, as it was not found in random sets of PCs recorded from the vermis.
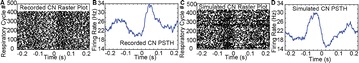


**Figure 1. A**. CN neuron spike raster centered on inspiration. **B**. Spike rate changes in CN neuron related to respiration. **C, D**. Simulated CN neuron with matching spike rate statistics (mean rate, CV, local variation) and respiratory modulation depth. To achieve this match ~50 PC ASTs were created as inputs to the model, of which 25 showed respiratory modulation

**Acknowledgements**

We acknowledge the contribution of Ying Cao to obtain experimental recordings and to Selva Maran of conducting the initial simulations leading to this study.

**References**

1. Cao, Y., S. K. Maran, et al. Behavior-Related Pauses in Simple-Spike Activity of Mouse Purkinje Cells Are Linked to Spike Rate Modulation. *J. Neurosci 2012* **32**(25): 8678–8685.

2. Steuber V, Schultheiss NW, Silver RA, De Schutter E, Jaeger D: Determinants of synaptic integration and heterogeneity in rebound firing explored with data driven models of deep cerebellar nucleus cells. *J Comput Neurosci* 2011, **30**:633–658.

## P116 A method to generate realistic artificial spike trains as inputs to biophysical neuron models: cerebellar mossy fibers as a case study

### Joel Lee^1^, Samira Abbasi^2^, Amber E. Hudson^1^, Detlef H Heck^3^, Dieter Jaeger^1^

#### ^1^Department of Biology, Emory University, Atlanta, GA 30033, USA; ^2^Department of Biomedical Engineering, Hamedan University of Technology, Hamedan, 65169-13733, Iran; ^3^Department of Anatomy and Neurobiology, University of Tennessee Health Science Center, Memphis, TN 38163, USA

##### **Correspondence:** Joel Lee (joel.lee@emory.edu)

*BMC Neuroscience* 2017, **18(Suppl 1)**:P116

The ability to create a predictive and biologically realistic model of neuronal firing provides a framework in which one can explore synaptic integration in vivo. In this study, we propose a method to generate artificial spike trains (AST) that closely resemble biological recordings. This study is based on an electrophysiological dataset of cerebellar mossy fibers (MF) related to respiratory activity from awake mice [1]. Our method is based on matching firing rate changes from recordings in a rate template constructed by a two-step adaptive Gaussian process. Creating rate templates by convolving spikes with a Gaussian minimizes the loss of spike timing information and spreads the signal power away from spike times [2]. Here, we use Gaussians with a kernel width that is adapted to slow changes in the mean firing rate. This method is capable of capturing both slow and fast firing rate fluctuations in the rate template (Fig. 1A). To replicate respiratory rate modulations, we further convolve the average peri-respiratory rate change into our adaptive Gaussian rate template (GRT) at the recorded times of inspiration. Afterwards, artificial spikes are drawn from a gamma distribution with a time-varying mean ISI following the adaptive GRT and a regularity factor kappa matching the recorded spike train variability [3]. The AST constructed using this method show close resemblance to the biological recordings. Our sample MF recording had a mean firing rate of 29.79 Hz, a local variation (LV) of 0.34, and a coefficient of variation (CV) of 0.73. The average of 20 ASTs had a mean firing rate of 29.43 Hz, a LV of 0.35, and a CV of 0.79. The ISI distribution of the AST is slightly shifted to the left compared to the biological MF recordings, but otherwise shows a close match (Fig. 1B). The results demonstrate the viability of the proposed method for generating ASTs which may be successfully implemented as inputs into biophysical neuron models.
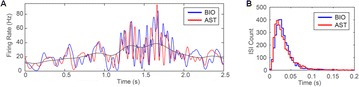


**Figure 1.** Comparison of the rate functions and ISI distribution between the recording and AST. **A.** The adaptive rate function from the biological MF recording and the AST showing a select window of 2.5 s. The black trace represents the slow rate function of the biological MF recording using a wide kernel width. The blue trace is the biological adaptive GRT created using the slow rate function to determine an adaptive kernel width. The red trace represents a GRT created using the AST spike time data. **B.** The interspike interval distributions of the recorded (blue) and artificial (red) spike trains

**References**

1. Cao Y, Maran SK, Dhamala M, Jaeger D, Heck DH: Behavior-related pauses in simple-spike activity of mouse Purkinje cells are linked to spike rate modulation. *J Neurosci* 2012, **32**(25):8678–8685.

2. Paulin MG, Hoffman LF: Optimal firing rate estimation. *Neural Netw* 2001, **14:**877–881.

3. Shinomoto S, Shima K, Tanji J: Differences in spiking patterns among cortical neurons. *Neural Comput* 2003, **15**:2823–2842.

## P117 Serotonergic fiber densities may emerge from random walks

### Skirmantas Janušonis^1^

#### ^1^Department of Psychological and Brain Sciences, University of California, Santa Barbara, CA 93106-9660, USA

##### **Correspondence:** Skirmantas Janušonis (skirmantas.janusonis@psych.ucsb.edu)

*BMC Neuroscience* 2017, **18(Suppl 1)**:P117

In addition to point-to-point projections, the brain contains numerous axons that do not have well-defined destinations. These axons (fibers) have meandering trajectories and eventually space-fill brain regions in a fractal-like fashion. Their cell bodies are typically located in the brainstem, as a component of the ascending reticular activating system, but they can also be in the forebrain (e.g., in the nucleus basalis of Meynert) or in the retina (e.g., as part of the non-image forming visual system). Macroscopically, these fibers form matrices that are typically analyzed using 2D- or 3D-density measures, disregarding the geometry of individual 1D-trajectories.

The structure of the fiber-matrix that releases serotonin (5-hydroxytryptamine, 5-HT) has particular clinical importance. These “serotonergic” fibers originate in the brainstem raphe nuclei and reach virtually all regions of the brain, including the prefrontal cortex. Altered densities of the serotonergic matrix have been associated with various mental disorders; for example, an abnormally high density of serotonergic fibers has been observed in the brains of individuals diagnosed with Autism Spectrum Disorder [1].

We have recently proposed that the brain may not actively produce or maintain region-specific fiber densities [2]. Instead, these densities may spontaneously emerge as a consequence of the behavior of individual fibers. Specifically, serotonergic fibers may perform a random walk in the heterogeneous material of brain tissue, and their density may reflect the stochastic properties of this walk. These properties, in turn, may depend on the spatial distribution of microscopic obstacles in the given volume. For example, the increased fiber density in autistic brains may be a consequence of an altered spatial distribution of cellular and subcellular elements and may not be directly associated with the cognitive symptoms of the disorder. This is supported by other studies that have demonstrated altered cell packing in autistic brains [3] and that have found surprisingly minor deficits in mice lacking brain serotonin [4].

Serotonergic fibers in the mouse forebrain were visualized with fluorescence immunohistochemistry and imaged at high resolution with confocal laser scanning microscopy. The obtained *z*-stacks of optical sections were analyzed with the Imaris (Bitplane) and BisQue (UCSB) platforms. Individual serotonergic fibers often followed very different directions in the same spatial location, suggesting that they are not guided by a gradient field and can be modeled as random walks. We are currently investigating whether these random walks can be captured by well-defined probability density functions.

**References**

1. Azmitia EC, Singh JS, Whitaker-Azmitia PM: Increased serotonin axons (immunoreactive to 5-HT transporter) in postmortem brains from young autism donors. *Neuropharmacology* 2011, **60:**1347–1354.

2. Janušonis S: Serotonin in space: Understanding single fibers. *ACS Chem Neurosci* 2017, in press.

3. Casanova MF, van Kooten IA, Switala AE, van Engeland H, Heinsen H, Steinbusch HW, Hof PR, Trippe J, Stone J, Schmitz C: Minicolumnar abnormalities in autism. *Acta Neuropathol (Berl)* 2006, **112:**287–303.

4. Mosienko V, Beis D, Pasqualetti M, Waider J, Matthes S, Qadri F, Bader M, Alenina N: Life without brain serotonin: reevaluation of serotonin function with mice deficient in brain serotonin synthesis. *Behav Brain Res* 2015, **277:**78–88.

## P118 A taxonomy of seizures based on dynamics

### Maria Luisa Saggio^1^, Andreas Spiegler^1^, William C. Stacey^2^, Christophe Bernard^1^, Viktor K. Jirsa^1^

#### ^1^INSERM UMR 1106 Institut de Neurosciences des Systèmes - Aix-Marseille Université, Marseille, 13005, France; ^2^Department of Neurology, Department of Biomedical Engineering, University of Michigan, Ann Arbor, MI 48109, USA

##### **Correspondence:** Maria Luisa Saggio (maria-luisa.saggio@etu.univ-amu.fr)

*BMC Neuroscience* 2017, **18(Suppl 1)**:P118

Epilepsy is one of the most common neurological disorders, affecting 50 million people in the world. One third of epileptic patients are drug resistant and are candidates for surgery or neurostimulation. Using a phenomenological model for seizure activity, the Epileptor [1], the patient specific whole brain connectivity obtained from DTI, and information on structural lesions and anomalies, Jirsa and colleagues created a personalized large-scale brain model, the Virtual Epileptic Patient [2]. Different treatment strategies, such as interventions on the network topology, stimulations and parameters changes, can be tested computationally using the model, providing a tool throughout the presurgical evaluation. Patients have different types of seizures, and some types are more common than others. Jirsa and colleagues [1] proposed a taxonomy of seizures based on Izhikevich’s classification of bursters [3]. Each class of the taxonomy differs in the bifurcations allowing the transition between healthy and ictal state and vice versa. The taxonomy contains sixteen classes and the Epileptor models the most predominant class.

We used the theory developed for neuronal bursters and unfolding theory [4] to build a single generic model producing bursting activity of all types, except one, within the classes of the taxonomy [5]. While a bifurcation cannot describe the unique characteristics of an individual seizure, it does capture the basic mechanism generating or suppressing a seizure. For this reason, building models for different types of seizures is not only an important step towards a further personalization of the Virtual Epileptic Patient, but can provide a conceptual framework to understand the mechanisms generating this variety. In addition, differences in the dynamics underlying seizures have an impact on features relevant for treatment, such as seizure propagation, or the reaction to stimulation. Our model can be used to tackle these issues in a seizure type dependent fashion. The model provides a ranking of the complexity of the classes in the taxonomy. The more complex a class, the less likely it is to occur in nature. It also predicts that one seizure class can switch to another class if some parameters of the model fluctuate, and only some transitions are allowed. This implies that the same patient may have seizures belonging to more than one class depending on the values of the physiological correlates of the relevant parameters of the model. In addition, in rarer cases, transitions between classes could occur within a seizure. To validate our predictions, we collected data from 85 patients (over 2100 seizures) recorded from intracranial depth and grid electrodes. We analyzed seizures’ offsets to identify the bifurcation type. We also performed a longitudinal analysis in a group of patients having intracranial electrodes implanted for several months. Results are consistent with the theoretical framework proposed, in particular the most surprising finding is that most patients have more than one class of seizures contrary to standard clinical teachings, and that transitions between classes can occur during a single seizure.

By identifying and understanding the dynamical characteristics of target seizures, interventions on drug resistant epileptic patients will improve significantly.

**References**

1. Jirsa VK, Stacey WC, Quilichini PP, Ivanov AI, Bernard C: On the nature of seizures dynamics. *Brain* 2014, **137(8):**2210–2230.

2. Jirsa VK et al.: The Virtual Epileptic Patient: Individualized whole-brain models of epilepsy spread. *NeuroImage* 2017,**145**:377–388.

3. Izhikevich EM: Neural excitability, spiking and bursting. *International Journal of Bifurcation and Chaos* 2000, **10(06):**1171–1266.

4. Golubitsky M, Josic K, Kaper TJ: An unfolding theory approach to bursting in fast-slow systems. *Global analysis of dynamical systems* 2001, **18:**277–308.

5. Saggio ML, Spiegler A, Bernard C, Jirsa VK: Fast-slow bursters in the unfolding of a high codimension singularity and the ultra-slow transitions of classes. *arXiv preprint* arXiv:1605.09353 2016.

## P119 Computational model for diffusion-induced bursting of biophysically realistic HH-type neuron: mathematical characterization

### Davide Lillo^1^, Christophe Bernard^1^, Viktor Jirsa^1^

#### ^1^Institut de Neurosciences des Systèmes, Aix Marseille Univ, Marseille, 13005, France

##### **Correspondence:** Davide Lillo (davide.lillo@etu.univ-amu.fr)

*BMC Neuroscience* 2017, **18(Suppl 1)**:P119

Ion-based neuron models implement the effects of charge displacements due to transmembrane currents on the overall concentrations of the ion species involved in the neuron’s spiking mechanism. After a spiking period, such charge displacements sum up and we can observe an evolution of ionic concentrations on a much slower timescale than spiking dynamics’. This evolution in turns affects the neuron’s firing patterns. In the repertoire of behaviors of such models, we focus on the ionic-oscillation-induced bursting patterns of a HH-type model embedded in an infinite potassium bath [1,2]. We consider a HH-type neuron with persistent K^+^ current, transient Na^+^ current, leak Cl^−^ current and ATP pump current. The conductance-based equations for this model are coupled to the equations describing the change of ionic concentrations in the intracellular and extracellular space, as well as to the equation for passive diffusion from the K^+^ bath. The total set of 11 variables is reduced to a set of 4 variables using constraints upon mass/charge, approximations of gating dynamics, and time scale separation. A 2D bifurcation diagram of the fast subsystem, comprised of membrane potential and K^+^ activation variable, is obtained for each fixed couple of values of the two slow variables (intra- and extracellular shift of K^+^ concentration from resting values). The passive diffusion from external K^+^-bath induces a slow-wave bursting behavior of the neuron for a certain range of bath concentrations. This can be visualized as a closed trajectory in the slow-variables’ plane, which crosses a SNIC line in both onset and offset bifurcations of the fast subsystem. Such SNIC/SNIC burster appears to be robust to slight changes of the metabolic parameters in the model, but bursters of other classes are observed as transients before stabilization to the aforementioned class.

Our model is able to combine physiological realism and computational convenience to reproduce a K^+^-elevation-induced bursting. This might serve as starting point for a further extension to neuro-glial biochemical coupling: we might observe different trajectories in the slow-variable’s plane and thus an automatic mathematical characterization of different physiological situations. Another valuable promise for the future is the translation of this local model for a neuro-glial system to a population level formalism, through mean-field techniques.

**References**

1. Cressman JR, Ullah G, Ziburkus J, Schiff SJ: The influence of sodium and potassium dynamics on excitability, seizures, and the stability of persistent states: I. Single neuron dynamics. *J Comput Neurosci* 2009, **26:** 159–170.

2. Hübel N, Dahlem MA: Dynamics from seconds to hours in Hodgkin-Huxley model with time-dependent ion concentrations and buffer reservoirs. *PLoS Comput Biol* 2014, **10(12):** e1003941.

## P120 Phase-lags in large scale brain synchronization

### Spase Petkoski, Viktor K. Jirsa

#### INSERM UMR 1106 Institut de Neurosciences des Systèmes - Aix-Marseille Université, Marseille, France

##### **Correspondence:** Spase Petkoski (spase.petkoski@univ-amu.fr)

*BMC Neuroscience* 2017, **18(Suppl 1)**:P120

Network couplings of oscillatory large-scale systems, such as the brain, have a space-time structure composed of connection strengths and signal transmission delays and both contribute to the system’s spatiotemporal organization [1]. Rhythms and their synchronization, as one of the key mechanisms of brain function are particularly sensitive to delays, which become notably long in large-scale brain models with biologically realistic connectivity [2].

We show theoretical and in silico numerical results for phase coherence between signals from different brain regions. For this we build on the Kuramoto model with spatially distributed time delays [3], where the network connectivity strengths and distances are defined by the connectome.

For all-to-all networks with delay-imposed structure based on the connectome [3], we analytically derive and numerically confirm the statistics for the phase shifts in relation to the strength and spatial position of the nodes (Figure 1A). These depend on the frequency and the coupling strengths, and the most realistic scenario for the brain signals is for weaker nodes to lead over stronger for in-phase, and vice versa for anti-phase synchronization. In-silico simulations of brain activity (Figure 1B-C) confirm those findings for the connectome-derived brain network model and unveil the same in- and anti-phase motifs. In addition, the simulations capture the realistic patterns for the phase-locking values, which are commonly used for empirical description of the phase dynamics. In this way, we quantify the impact of the connectome to the synchronization patterns between distant brain regions.
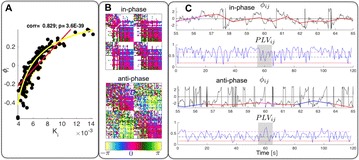


**Figure 1.** Coupled phase oscillators with (**A**) log-normally distributed and (**B-C**) connectome-derived couplings. **A.** Phase shifts for different node strengths (numerical –circles, theoretical – yellow, linear correlation - red). **B.** In- and anti-phase synchronization between significantly coherent links, with nodes in each hemisphere sorted by in-strength. **C.** (upper plots) Instantaneous (black) and time-averaged phase differences (red and magenta for different levels of significance). These correspond to the shaded part of the phase locking values (PLV) (blie) in the lower plots, shown with the significance levels (red and magenta). Parameters: D = 1, f = 20 Hz, v = 5 m/s. (A) τ = [0.05,0.2] s, N = 300 (B-C) N = 68, K = 1

**References**

1. Sanz-Leon P et al.: Mathematical framework for large-scale brain network modeling in the virtual brain. *Neuroimage* 2015, **111**, 385–430

2. Deco G, Jirsa V, McIntosh AR, Sporns O, Kötter R: Key role of coupling, delay, and noise in resting brain fluctuations. *Proc Natl Acad Sci USA* 2009, **106 (25):** 10302–10307

3. Petkoski S et al.: Heterogeneity of time delays determines synchronization of coupled oscillators. *Phys Rev E* 2016, **94**, 012209

## P121 Network dynamics after focal stimulation in a connectome-based network model of the mouse brain

### Andreas Spiegler, Viktor Jirsa

#### Institut de la Santé et de la Recherche Médical, Institut de Neurosciences des Systèmes UMR_S 1106, Aix Marseille Université, 13005 Marseille, France

##### **Correspondence:** Andreas Spiegler (andreas.spiegler@univ-amu.fr)

*BMC Neuroscience* 2017, **18(Suppl 1)**:P121

Systematic exploration via stimulation of all cortical and subcortical brain areas can only be performed in silico. The pattern formation of brain activity after stimulation is constrained by the structural connectivity of the brain. The extent to which information is processed over short- and long-range connections is unclear. Here we use a whole-brain model of the mouse to explain the brain activity following stimulation.

The model bases on the structural brain connectivity data [1]. The mouse brain is modeled as a network of 14400 nodes, each can oscillate at 42 Hz [2]. The short-range connectivity links the 13972 nodes of the isocortical surface, which is divided into 84 areas. The remaining 428 nodes represent areas such as cerebral and thalamic nuclei. The long-range connectivity links the 512 areas with a delay. Brain areas are stimulated, and the effect of short- and long-range connectivity is investigated. The responsive networks are extracted and analyzed.

The in silico exploration provides consistent activation patterns and a map (see Figure 1) indicating which brain areas need to be stimulated to place the brain in a particular state. Stimulations specifically bias widespread functional networks. Spatial proximity of stimulation does not necessarily predict the similarity of induced activities. The same pattern of activity can be induced by stimulation of several sites scattered all over the brain. Slow time scales characterize the responsive networks. Activity patterns were then compared to the voltage-sensitive dye (VSD) imaging [3].

The resulting catalogue of responsive networks and their stimulation sites can be used for future experiments. The model can integrate and evaluate diverse experimental data such as VSD, which measures activity in wide parts of the isocortex, and such electrophysiological signals as local field potentials, which measure small volumes that can lie deeper in the brain. This approach can also be used for the human brain.
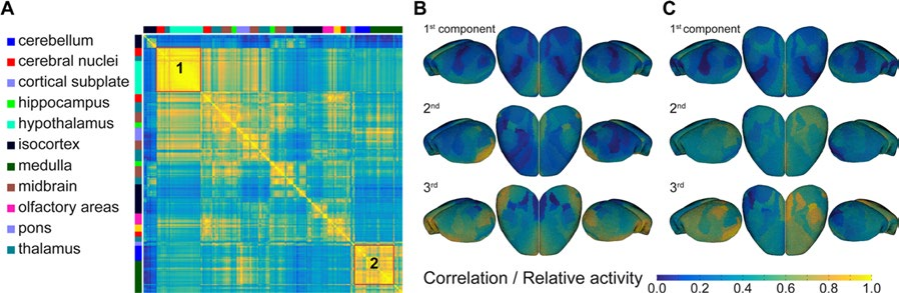


**Figure 1.** Specific focal stimulations activate similar networks. Panel **A** is the similarity matrix of stimulation with the clusters 1 and 2. Panels **B** and **C** show the responsive networks (isocortex) of cluster 1 and of cluster 2

**References**

1. Allen Brain Atlas [http://connectivity.brain-map.org]

2. Spiegler A, Hansen ECA, Bernard C, McIntosh AR, Jirsa VK: Selective activation of resting state

networks following focal stimulation in a connectome-based network model of the human brain. *eNeuro* 2016, **3:**ENEURO.0068-16.2016.

3. Mohajerani MH, Chan AW, Mohsenvand M, LeDue J, Liu R, McVea DA, Boyd JD, Wang YT, Reimers M, Murphy TH: Spontaneous cortical activity alternates between motifs defined by regional axonal projections. *Nat Neurosci* 2013, **16(10):**1426–35.

## P122 What is the feasibility of estimating axonal conduction delays from micro-structural MRI?

### Mark Drakesmith^1,2^, Derek K Jones^1,2^

#### ^1^Cardiff University Brain Research Imaging Centre, Cardiff University, Cardiff, UK; ^2^Neuroscience and Mental Health Research Institute, Cardiff University, Cardiff, UK

##### **Correspondence:** Mark Drakesmith (drakesmithm@cardiff.ac.uk)

*BMC Neuroscience* 2017, **18(Suppl 1)**:P122

Recent developments in MRI have promised to provide non-invasive, in vivo estimates of specific microstructural indices of white matter e.g. axon diameter [1] and g-ratios [2], which are known to contribute to conduction speed. This opens the possibility of generating in vivo conduction delay maps. However, there are many other parameters that impact on conduction speed but are not possible to image non-invasively (e.g. morphology of nodes of Ranvier, inter-nodal spacing, electrical membrane properties). Some of these parameters may have high variability across fibre populations and individuals. Here we quantify the effects of variability in these parameters on estimated conduction speed. The effects of 17 parameters (figure 1) in the axon model of Richardson et al. [3] on conduction speed were tested using Fourier amplitude sensitivity testing (FAST) [4]. Model parameters for the optic nerve [5] were used as an approximation for CNS white matter axons. Each parameter was varied sinusoidally at frequencies specific to each parameter, across 13,741 model runs. Bounds for parameter variation were defined within ±1 s.d. from experimental observations [5,6], or ±20% for parameters where no variance data is available. Conditional variances were estimated from the coefficients of the Fourier series from the model outputs. Sensitivity indices were obtained from the summed conditional variances for each parameter normalised to the total variance. Variance in conduction speed is dominated by variance in axon diameter, but with also some contribution from g-ratio (figure 1), which are parameters that can be estimated from MRI. Together they constitute 75.7% of the total output variance. This suggests it is feasible to obtain reliable estimates of axonal conduction delay from micro-structural parameters. Further analysis is required to determine the impact of imaging noise on these estimates. In addition, further analysis is will determine if number of myelin wraps or due to overall myelin thickness underlie the strong effects of these parameters.
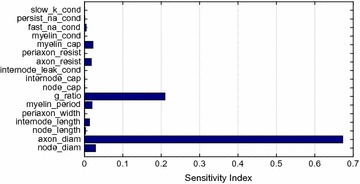


**Figure 1.** FAST sensitivity indices for the 17 tested parameters

**Acknowledgements**

This work was funded by a Wellcome Trust New Investigator Award to DKJ. We thank Lee Cossell & David Attwell for providing the simulation programme used [5].

**References**

1. Assaf Y, Basser PJ. Composite hindered and restricted model of diffusion (CHARMED) MR imaging of the human brain. *Neuroimage* 2005; **27**:48–58.

2. Stikov N, Campbell JSW, Stroh T, Lavelée M, Frey S, Novek J, et al. In vivo histology of the myelin g-ratio with magnetic resonance imaging. *Neuroimage* 2015; **118**:397–405.

3. Richardson AG, Mclntyre CC, Grill WM. Modelling the effects of electric fields on nerve fibres: influence of the myelin sheath. *Med. Biol. Eng. Comput.* 2000; **38**:438–46.

4. McRae GJ, Tilden JW, Seinfeld JH. Global sensitivity analysis-a computational implementation of the Fourier Amplitude Sensitivity Test (FAST). *Comput. Chem. Eng.* 1982; **6**:15–25.

5. Arancibia-Cárcamo IL, Ford MC, Cossell L, Ishida K, Tohyama K, Attwell D. Node of Ranvier length as a potential regulator of myelinated axon conduction speed. *Elife,* 2017; **6**:1–15.

6. Brinkmann BG, Agarwal A, Sereda MW, Garratt AN, Müller T, Wende H, et al. Neuregulin-1/ErbB Signaling Serves Distinct Functions in Myelination of the Peripheral and Central Nervous System. *Neuron.* 2008; **59**:581–95.

## P123 Analysing the impact of sodium channels in Alzheimer’s disease using a computational model

### Ali Sadegh Zadeh, Chandra Kambhampati

#### Department of Computer Science, University of Hull, Hull, HU6 7RX, UK

##### **Correspondence:** Ali Sadegh Zadeh (S.A.Sadegh-Zadeh@2014.hull.ac.uk)

*BMC Neuroscience* 2017, **18(Suppl 1)**:P123

Research indicates that a significant perturbation of sodium ion homoeostasis in the brain could be important for the pathophysiology of brain dysfunction in Alzheimer’s disease (AD) [1]. AD is a progressive disease that is often characterised by the abnormal proteolytic processing of the Amyloid precursor protein [2]. The process of creating the protein which results in the production of Amyloid Beta Peptide (Aβ). Research shows that high levels of amyloid beta peptide has an impact on the normal functioning of the nervous system [3]. This increase in Aβ results in the creation of micro pores which act as additional ion channels on the membrane of neurons. These additional channels cause an imbalance in the ions of the neuron and hence its response to stimulates. As a result, it has been proposed that AD progresses with an increasing level of Aβ. In extreme this production of Aβ results with an initial damage to the neurons, eventually leading to neuronal death.

The creation of additional pores results in the channel hypothesis which states that the forming of additional ion channels, results in leakage of cations across the membranes of neurons [3]. The leakage of the ions results in a change in the expected response of the neuron to stimulus (as it can be seen in the Eq. 1).

$$ \frac{1}{C}\dot{V} = I_{inj} - \mathop \sum \limits_{i = 1}^{\infty } I_{\text{i}} ,\quad I_{i} = {\text{leakage current due to the ions}} \hfill (1)$$

The increase in leakages which results in a change in neuronal activity, are often reflected in clusters of neurons which exhibit excessive levels of inhibition [1,4,5]. This increased inhibition is often considered to be a first sign of AD [1]. Although specific cellular mechanisms leading to this interneuronal dysfunction are not completely understood, recent studies suggest that the formation of ion channels results in an upregulated sodium (Na^+^) leak [3]. Na^+^ is an essential electrolyte plays an important role in generating nerve impulses. This is because Na^+^ channels control the depolarisation phase of the membrane potential, and thus extreme high or low Na^+^ changes the magnitudes of the spikes in membrane potential [6,7]. This incorrect depolarisation means that there is a failure to reliably produce the right action potentials [8].

This paper investigates these mechanisms by simulating neurons for different levels of Aβ. The response of a neuron is examined for different sodium ion leakages. This vitro computational study confirms the earlier in vivo studies, and it is possible to develop bounds on the changes in Na^+^ for a given neuron. Further this analysis also explains the observations in the relationship between increased sodium leakage and increased inhibitory activity of inhibitory neurons.

**References**

1. Burkitt AN, & Clark GM: Synchronization of the Neural Response to Noisy Periodic Synaptic Input. *Neural Computation* 2001, **13(12):** 2639–2672.

2. Ding SL, Royall JJ, Sunkin SM, Ng L, Facer BA, Lesnar P, Christopher: Comprehensive cellular-resolution atlas of the adult human brain. *Journal of Comparative Neurology* 2017, **525(2)**.

3. Perez C, Ziburkus J, Ullah G: Analyzing and Modeling the Dysfunction of Inhibitory Neurons in Alzheimer’s Disease. *PloS one* 2017, **11(12).**

4. Rybak IA, Jasinski PE, Molkov YI, Smith JC: Modeling Na^+^- and Ca^2+^-dependent mechanisms of rhythmic bursting in excitatory neural networks. *BMC Neuroscience* 2012,13(1), P38.

5. Sharpee T, Rust N, Bialek W: Analyzing neural responses to natural signals : maximally informative dimensions. *Neural computation* 2004, **16(2):**223–250.

6. Sukbin L, Mark SG: Balanced Cortical Microcircuitry for Spatial Working Memory Based on Corrective Feedback Control. *Journal of Neuroscience* 2014, **34(20):**6790–6806.

7. Tchumatchenko T, Clopath C: 2015. The contribution of subthreshold preference in inhibitory neurons to network response. Prague, Czech Republic: 24th Annual Computational Neuroscience Meeting: CNS*2015.

8. Victor M, Sanjay KG, Richard FK, Roger LA, Ruma B: Na + and K + ion imbalances in Alzheimer’s disease. *Biochim Biophys Acta* 2013, **1822(11):**1671–1681.

## P124 Models of brain design: is physics more important than evolutionary optimization?

### Jan Karbowski

#### University of Warsaw, 02-097 Warsaw, Poland

##### **Correspondence:** Jan Karbowski (jkarbowski@mimuw.edu.pl)

*BMC Neuroscience* 2017, **18(Suppl 1)**:P124

Empirical data indicate that there are some structural [1] and energetic [2] regularities in the organization of mammalian brains. These include scaling relations among various brain parameters (e.g. scaling of gray matter vs. white matter [3]) and invariant parameters (e.g. constancy of synaptic density [1,4]). The existence of invariants, i.e. conserved parameters across species with brain sizes differing by 3-4 orders of magnitude, suggests that (i) there are certain universal features in brain design, and (ii) the observed brain design has been selected (preferred) by evolution over other possible designs [4]. We might think about the actual design as optimal in some sense. The question is: What sets this optimality? Is this the laws of physics or some kind of “laws of functionality”?

It is amazing that the term “functionality” essentially does not appear in physics textbooks, which reflects the fact that we do not need such terms to successfully describe non-living matter. On the contrary, in biology textbooks the word “functionality” appears frequently and seems to be fundamental for our understanding of evolution. The main difficulty in constructing a theory of brain design is to know what “functionality” for the brain would mean. Can the laws of non-equilibrium thermodynamics (e.g. minimum of entropy production) be useful in formulating such functional principles of grand scale brain architecture? We do not know for sure, but as for now, the answer seems to be negative. There exist a few models of brain structural design and none of them invokes physical laws as founding principles.

The most popular model of brain design is based on efficient neural connectivity and it is called the principle of neural “wire volume/length minimization” [3,5], which relates to savings in local timing and material. However, data analysis of neuronal connections revealed that it cannot be exactly right [6], and it was proposed that a better principle would be a compromise between saving wire and enabling fast long-distance communication [6–8]. All these models are based on optimization of a certain “fitness” function, in accord with ideas of evolutionary optimization, but they are indifferent to physical laws.

However, the above design principles cannot explain other non-neuronal features of brain tissue (brain consists also of supporting elements such as glia and capillaries, which provide metabolic energy). As an alternative, a new principle was proposed recently, which can be called “spine economy maximization” that maximizes the fraction of synaptic connections among neurons and simultaneously minimizes the energy use by them. This principle is motivated by two empirical observations: long-term information in the brain is stored in synapses, and synapses are energetically costly. Thus, maximizing the fraction of synapses maximizes also information with energetic constraint. This new principle takes to some degree thermodynamics into account, at least indirectly, and can explain a hierarchical organization of cortical tissue.

Recently, the “spine economy maximization” principle was used to derive from a first principle the scaling between gray and white matter (i.e. relation between local and global brain architecture), and it agreed with the empirical data.

To answer the question from the title. Physics has not contributed much to our understanding of global and local brain design. However, there are still many mysteries concerning brain organization, and with more involvement of physicists one might hope to reveal some deep connections between brain structure and function.

**References**

1. Braitenberg V, Schuz A: Cortex: Statistics and Geometry of Neuronal Connectivity, Springer, Berlin, 1998.

2. Karbowski J: Global and regional brain metabolic scaling and its functional consequences. *BMC Biology* 2007**, 5**:18.

3. Wen Q, Chklovskii DB: Segregation of the brain into gray and white matter. *PloS Comput Biol* 2005, **1**: e78.

4. Karbowski J: Constancy and trade-offs in the neuroanatomical and metabolic design of the cerebral cortex. *Front. Neural Circuits* 2014, **8**:9.

5. Chklovskii DB, Sikorski T, Stevens CF: Wiring optimization in cortical circuits. *Neuron* 2002, **34**:341.

6. Kaiser M, Hilgetag CC: Nonoptimal component placement, but short precessing paths, due to long-distance projections in neural systems. *PLoS Comput Biol* 2006, **2**: e95.

7. Karbowski J: Optimal wiring principle and plateaus in the degree of separation for cortical neurons. *Phys. Rev. Lett.* 2001, **86**: 3674.

## P125 Perceptual Attractors and Neural Confusions in Phoneme Manifolds

### Zeynep Gokcen Kaya^1^, Yair Lakretz^2^, Alessandro Treves^1,3^

#### ^1^Cognitive Neuroscience Sector, SISSA, Trieste, 34136, Italy; ^2^Sagol School of Neuroscience, Tel Aviv University, Tel Aviv, 6997801, Israel; ^3^Kavli Institute, Norwegian University of Science and Technology, Trondheim, 7491, Norway

##### **Correspondence:** Zeynep Gokcen Kaya (zgkaya@sissa.it)

*BMC Neuroscience* 2017, **18(Suppl 1)**:P125

Traditionally, phoneme representation in the brain has been studied within the simple notion of a tree-like structure of hierarchically clustered discrete items [1]. Our aim is to understand the neural representation of phonemes without any prior assumption on its organization, but allowing for the continuous and the multifactorial aspects that defy hierarchies and discreteness. Further, we want to focus on its short and long-term dynamics, how perceptual clues change over the time course of phoneme production and how the “phoneme manifold” is shaped by the memory templates set up by the mother tongue.

We use both behavioral (phoneme confusion frequencies) and neural measures (the spatio-temporal distribution of phoneme-evoked neural activation). Using EEG, we replicate and extend a classical study on sub-phonemic features underlying perceptual differences between phonemes [3]. Comparing the responses of native listeners to that of Italian, Turkish, Hebrew, and (Argentinian) Spanish listeners to a range of American English vowels and consonants, we confirm the superiority of native listeners at identifying the phonemes of their mother tongue [2].

Beyond the analysis of percent correct scores, and transmitted information, we frame the problem in terms of attractors, in analogy to those which have been well studied in spatial memory. Do different phoneme attractors exist in different languages? We find that at the consonant feature level, for these five languages, the corresponding large basins of attraction appear at different places of articulation. Our preliminary analysis suggests listeners set up deeper attractors at phonemes that have higher frequencies in their mother tongue. In order to further investigate language specific place attractors in conditions mimicking real world situations, we look at confusions among meaningful English words differing only in the initial consonant sound.

Moreover, we look at the brain signals to see if neural discriminations match perceptual confusions. We are interested in learning which sub-phonemic features are informative about the identity of a phoneme and when, and which features are perceived differently by non-native listeners, to produce, over and beyond the effects of semantics, context, etc., suboptimal speech comprehension.

**References**

1. Bouchard KE, Mesgarani N, Johnson K, Chang EF: Functional organization of human sensorimotor cortex for speech articulation. *Nat. Neurosci* 2013, **495:**327–332.

2. Cutler A, Weber A, Smits R, Cooper N: Patterns of English phoneme confusions by native and non-native listeners. *J. Acoust. Soc. Am.* 2004, **116(6):**3668–3678.

3. Miller GA, Nicely PE: An Analysis of Perceptual Confusions Among Some English Consonants. *J. Acoust. Soc. Am.* 1955, **27(2):**339–352.

## P126 Network analysis of task-oriented neuroimaging data via multivariate information-theoretic measures

### Lily W. Li^1^, Joseph Lizier^2^, Paula Sanz-Leon^1,3^, Cliff C. Kerr^1,3^

#### ^1^School of Physics, University of Sydney, NSW 2006, Australia; ^2^School of Civil Engineering, University of Sydney, NSW 2006, Australia; ^3^Centre for Integrative Brain Function, University of Sydney, NSW 2006, Australia

##### **Correspondence:** Cliff C. Kerr (cliff@thekerrlab.com)

*BMC Neuroscience* 2017, **18(Suppl 1)**:P126

Human motor behaviors are modulated by the dynamic interactions between motor and sensory regions of the cerebral cortex. When motor tasks are cued by visual or aural stimuli, a functional network describing the causal relations between different brain areas yields insights into the underlying neurophysiological mechanisms responsible for simple movement executions. However, capturing the nonlinear causal interactions between brain regions using limited neurophysiological data requires a model-free approach and a way of formalizing the concept of causality. In this study, we use transfer entropy (TE) [1] to explore functional connectivity patterns between brain regions using two sets of neuroimaging data, electrocortographic (ECoG) and magnetoencephalographic (MEG) data. TE is an information-theoretic reformulation of the concept of predictive information flow, and can be interpreted as the improvement in the prediction of the future of a given time series conditioned on its own past by incorporating the past of a second time series [2]. The spatial and temporal resolution of the ECoG recordings, as well as the definition of individual electrodes as nodes in the connectivity network, are particularly valuable and suitable for our information-theoretic approach. With MEG recordings, whilst the spatial accuracy of reconstructed activity at source level is reduced, its non-invasive nature means that experimental data is comparatively abundant. ECoG data were taken from a visually cued simple hand movement task, performed during a study on 14 pre-surgery epileptic human patients [3]. MEG data were taken from a cohort of over 600 adult participants in the 2014 Cambridge Centre for Ageing and Neuroscience (Cam-CAN) study [4], and consisted of a similar motor task as was used in the ECoG study, except that it was cued both visually and aurally.

TE estimation for quantifying internodal (or interregional) connectivity for both datasets was achieved using the open-source software package TRENTOOL [5]. The statistical significance of the connectivity between each electrode pair was determined using the nonparametric approach of permutation testing. In both datasets, we found a number of significant causal relationships between electrodes, including from the primary visual cortex to other areas, indicating complex patterns of information flow between brain regions. These patterns differed both between studies and, to a lesser extent, between individuals. These results suggest that TE (i) captures the inter-subject similarities between functional brain networks involved in executing simple motor tasks, and (ii) reflects the differences in functional connectivity due to different sensory modalities.

**References**

1. Schreiber, T: Measuring information transfer. *Phys Rev Lett* 2000, **85**:461–464.

2. Lizier JT, Heinzle J, Horstmann A, Haynes JD, Prokopenko M: Multivariate information-theoretic measures reveal directed information structure and task relevant changes in fMRI connectivity. *J Comp Neurosci* 2011, **30(1):**85–107.

3. Miller KJ, Hermes D, Honey CJ, Hebb AO, Ramsey NF, Knight RT, Ojemann JG, Fetz EE: Human motor cortical activity is selectively phase-entrained on underlying rhythms. *PLoS Comput Biol* 2012, **8(9)**:e1002655.

4. Shafto MA, Tyler LK, Dixon M, Taylor JR, Rowe JB, Cusack R, Calder AJ, Marslen-Wilson WD, Duncan J, Dalgleish T, Henson RN: The Cambridge Centre for Ageing and Neuroscience (Cam-CAN) study protocol: a cross-sectional, lifespan, multidisciplinary examination of healthy cognitive ageing. *BMC Neurology* 2014, **14(1)**:204.

5. Lindner M, Vicente R, Priesemann V, Wibral M: TRENTOOL: A Matlab open source toolbox to analyse information flow in time series data with transfer entropy. *BMC Neuroscience* 2011, **12(1)**:119.

## P127 Optimal localist and distributed coding of spatiotemporal spike patterns through STDP and coincidence-detection

### Timothée Masquelier^1^, Saeed Reza Kheradpisheh^2^

#### ^1^CERCO UMR 5549, CNRS – Université de Toulouse 3, Toulouse, F-31300, France; ^2^Department of Computer Sc., School of Mathematics, Statistics, and Computer Science, Univ. of Tehran, Tehran, Iran

##### **Correspondence:** Timothée Masquelier (timothee.masquelier@cnrs.fr)

*BMC Neuroscience* 2017, **18(Suppl 1)**:P127

Repeating spatiotemporal spike patterns exist and carry information. Here we investigate how a single neuron can optimally signal the presence of one given pattern (localist coding), or of either one of several patterns. In the latter case, the response of the neuron is ambiguous, but the identity of the pattern could be inferred from the response of multiple neurons, a scheme known as distributed coding. Intuitively, we should connect the detector neuron to the neurons that fire during the patterns, or during subsections of them (we note Δ*t* their duration, cf. Fig. 1). Using a threshold-free leaky integrate-and-fire (LIF) neuron with non-plastic unitary synapses and homogeneous Poisson inputs, we derived analytically the signal-to-noise ratio $SNR = (V_{ \hbox{max} } - V_{\text{noise}} )/\sigma_{\text{noise}}$ of the resulting pattern detector, even in the presence of jitter. In most cases, this *SNR* turned out to be optimal for relatively short membrane time constant τ (at most a few tens of ms), and for Δ*t* ~ *τ*. In other words, long patterns are optimally detected by coincidence detectors working at a much shorter timescale, although these ignore most of the pattern [1]. When increasing the number of patterns *P*, the optimal *τ* and Δ*t* decrease. Unsurprisingly, the resulting *SNR* also decreases, but remains acceptable for several tens of independent patterns (that is with chance-level overlaps).

Next, we wondered if spike-timing-dependent plasticity (STDP) could enable a neuron to reach the theoretical optimum. We simulated a LIF equipped with additive STDP, and repeatedly exposed it to multiple input spike patterns. The LIF progressively became selective to every repeating pattern with no supervision, even when the patterns were embedded in Poisson activity. Furthermore, using certain STDP parameters, the resulting pattern detectors were optimal. Up to ~30 independent patterns could be learned by a single neuron using a relatively low depression rate, in contrast with our previous studies, in which higher depressing rates led to localist coding only [2].

Taken together these results suggest that coincidence-detection is a powerful mechanism, even in detecting multiple long spike sequences, and that the required connectivity can spontaneously emerge with STDP.
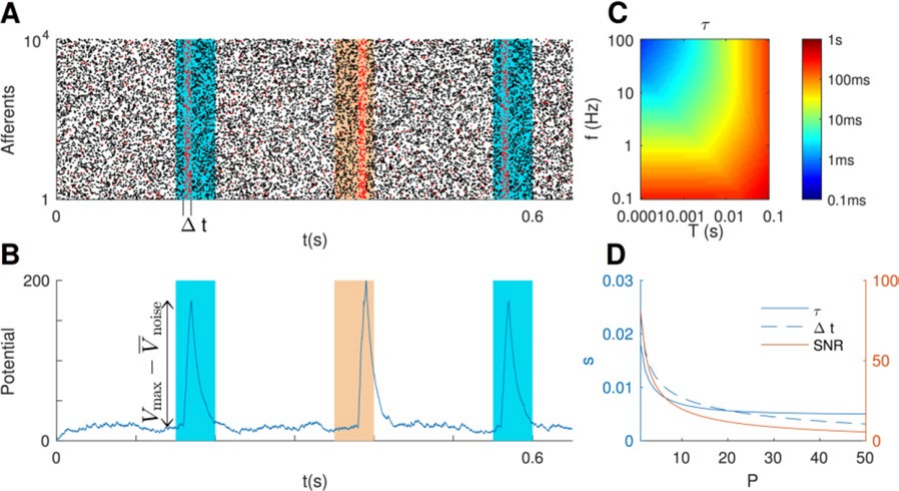


**Figure 1. A.** *P* = 2 repeating spike patterns (colored rectangles), embedded in Poisson noise. The LIF is connected to the neurons that fire in subsections of the patterns with duration Δ*t* (these emit red spikes). **B.** The LIF potential peaks for patterns, and we want to optimize the *SNR.* **C.** Optimal *τ* as a function of the mean input firing rate *f* and the jitter *T.* **D.** Optimal *τ* and Δ*t* (for *f* = 3 ms, *T* = 3 ms) and resulting *SNR* as a function of *P*

**Acknowledgements**

This research received funding from the European Research Council under the European Union’s 7^th^ Framework Program (FP/2007-2013)/ERC Grant Agreement no. 323711 (M4 project)

**References**

1. Masquelier T: STDP allows close-to-optimal spatiotemporal spike pattern detection by single coincidence detector neurons. *arXiv* 2016.

2. Masquelier T, Guyonneau R, Thorpe SJ: Competitive STDP-Based Spike Pattern Learning. *Neural Comput* 2009, **21**:1259–1276.

## P128 Muscle force potentiation induced by active dendrites of spinal motoneuron during locomotor-like movement

### Hojeong Kim

#### Convergence Research Institute, DGIST, Daegu, 42988, Korea

##### **Correspondence:** Hojeong Kim (hojeong.kim03@gmail.com)

*BMC Neuroscience* 2017, **18(Suppl 1)**:P128

Voltage-gated calcium channels generating persistent inward currents (Ca-PICs) in the dendrites of spinal motoneurons are actively involved for generation and control of normal movement [1]. However, the Ca-PIC effects on muscle force have been implied based solely on the input-output properties of the motoneurons. This study is focused on how the Ca-PIC activation influences the force output of motor units during locomotor-like movements. A model of motor unit was constructed combining two physiologically realistic models: one for the motoneuron including active dendrites and the other for the muscle unit reflecting movement dependent force production. The long-lasting step current with alternating excitatory and inhibitory pulses was injected at the soma of the motoneuron model and resulting force output was calculated from the muscle unit model while dynamically varying the muscle length. Force production without Ca-PIC activation was simulated by excluding the excitatory pulses from the current step protocol. Figure 1 shows the comparison of the force production with and without Ca-PIC activation during the movement. Ca-PIC induced force production was not apparent when the motoneuron transitioned into the stable firing state from the quiescent state by Ca-PIC activation (see the first half of the simulation in Figure 1). However, when the motoneuron switched from the low- to the high-frequency firing state by Ca-PIC activation, the force was increased by an average of almost 30% (see the second half of the simulation in Figure 1). The simulation results suggest that the Ca-PIC activation in the motoneuron dendrites may potentiate force production of the motor unit depending on the firing history of the motoneuron during the movement.
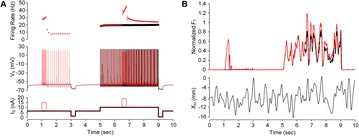


**Figure 1.** The motoneuron spiking and muscle force without (black) and with (red) Ca-PIC activation in the motoneuron dendrites. **A.** Instantaneous firing frequency (upper) of action potentials (middle) at the soma of the motoneuron model under the current step stimulation protocol (bottom). **B.** Normalized muscle force (upper) generated by electrical activities (V_S_ in A) of the motoneuron during locomotor-like movement (bottom)

**Acknowledgements**

This study was supported by the DGIST R&D Program of the Ministry of Science, ICT and Future Planning of Korea (17-BT-01).

**References**

1. Heckman CJ, Lee RH and Brownstone RM: Hyperexcitable dendrites in motoneurons and their neuromodulatory control during motor behavior. *Trens Neurosci* 2003, **26:**688–695.

## P129 Perceptual Mechanics of the Brain and the Free Energy Principle

### Chang Sub Kim

#### Department of Physics, Chonnam National University, Gwangju, 61186, Republic of Korea

##### **Correspondence:** Chang Sub Kim (cskim@jnu.ac.kr)

*BMC Neuroscience* 2017, **18(Suppl 1)**:P129

The free-energy principle (FEP) in neuroscience endeavors to understand how all living organisms perceive and act on their external world to maintain their physiological bounds and how they implement the operation of perception, learning, and action in the gray matter [1]. The brain’s maneuvering of organism’s internal states given sensory input is framed in the FEP by means of the mathematical measure of the informational free-energy (IFE) in a unified way.

In this work, we borrow the IFE as an informational Lagrangian (IL) of the brain and subsequently define the informational action (IA) as a continuous accumulation of the IFE over a temporal horizon of an agent. Then, by invoking the principle of least action [2], we derive a Hamilton-Jacobi-Bellman-type equation, which we call the perceptual mechanics. In this formulation, we do not need to employ the gradient descent scheme in optimal theory to execute minimization of the IFE. The resulting perceptual mechanics is statistically deterministic as Erwin Schrödinger phrased [3], emerging from the inherently stochastic equations of motion of states and sensory data.

We conceive of the IL as a function of the brain variables and their first-order time derivatives, admitting the position and velocity as only relevant dynamical variables. Our approach reveals that the non-Newtonian physical construct of the generalized motion in the FEP is not necessary to secure a fixed point of the perceptual dynamics and thus can be removed in formulating the FEP. According to our formulation, the brain’s Helmholtzian perception corresponds to finding an optimal trajectory in the hierarchical cortical network by minimizing the IA. When the brain fulfills the perceptual mechanics of reaching a desired fixed-point, it remains resting, i.e. being spontaneous, until another sensory stimulus will come in.

To manifest the utility of our formulation we take up the Hodgkin-Huxley (H-H) neurons as biophysical neural correlates of the basic perceptual units in the brain [4]. In the scope of the H-H model the membrane potential, ionic concentrations, and gating variables are the relevant brain variables. We formulate first how the sensory perception proceeds at the basic unit, a cellular level. Subsequently, we scale up the single-node formulation to form a cortical network with incorporating the functional hierarchical architecture of the brain. The hierarchical intertwining is assumed to be mediated by the connection variables, which describe synaptic interconnection and gap junction. Then, the perceptual state of the brain is specified by instant values of connected neural nodes, which define the perceptual neural network. When the initial default state is stimulated by an incoming sensory perturbation, the brain performs the perceptual dynamics by activating its internal variables. At the end of the perceptual computation, the brain finds an optimal trajectory in its functional network which minimizes the IA.

We propose here a hybrid theory of the biologically plausible information-theoretic FEP and the mechanical formulation complying with the physical principle of least action. As Hopfield once puts in words, ``it lies somewhere between a model of neurobiology and a metaphor for how the brain computes’’ [5].

**References**

1. Friston K The free-energy principle: a unified brain theory? *Nature Review Neurosci* 2010, **11**: 127–138.

2. Landau LP: *Classical Mechanics, 2nd Edition.* New York: Springer-Verlag; 1998.

3. Schrödinger E: What is Life? Mind and Matter, Cambridge: Cambridge Univ. Press: 1967.

4. Hodgkin A, Huxley A: A quantitative description of membrane current and its application to conduction and excitation in nerve. *J Physiol* 1952, **117**:500–544.

5. Hopfield JJ: Brain, neural network, and computation. *Rev Mod Phys* 1999, **71**:S431–S437.

## P130 Influence of handedness on the response inhibition in Stroop task: ERP study

### Julia A Marakshina^1,2^, Alexander V Vartanov^1^, Anastasia A. Neklyudova^1^, Stanislav A Kozlovskiy^1^, Andrey A Kiselnikov^1^

#### ^1^Lomonosov Moscow State University, Moscow, Russia; ^2^Psychological Institute of Russian Academy of Education, Moscow, Russia

##### **Correspondence:** Anastasia A. Neklyudova (elirafromthesea@gmail.com)

*BMC Neuroscience* 2017, **18(Suppl 1)**:P130

ERPs is an informative method for research and modeling of cognitive control functions. Early studies showed that response inhibition is one of the control functions related to the right brain hemisphere [1]. It should be noted that lateralization of inhibition could be related to functional lateral asymmetry in humans. Studies revealed behavioral differences between left- and right-handers during Stroop task performance [2]. The aim of this research is to find the differences in response inhibition in individuals with different lateral asymmetry by using ERPs method. The Russian words “red” and “green” written in red or green font were used in two Stroop tasks. The participants (N = 26, 18 right-handers and 8 left-handers) had to press right or left button as response to presentation of words written in red or green font, respectively (task 1). The same participants inhibited response to the word “red” and responded only to the word “green” (task 2). ERPs on these stimuli were recorded. We found significant differences in the amplitude of ERPs at the latency after 400 ms (P300) in all conditions using Student’s *t* test (see table 1). We analyzed the frontal leads due to crucial involvement of the frontal brain regions in the response inhibition.

**Table:** **Table 1.** The differences in P300 amplitude between groups of right- and left-handers (*p < 0.05)

Lead	Condition							
	“green” in green font		“green” in red font		“red” in green font		“red” in red font	
	Task 1	Task 2	Task 1	Task 2	Task 1	Task 2	Task 1	Task 2
F3	–	–	–	–	–	–	–	–
F4	*	–	*	–	*	–	–	*
F7	*	–	*	*	–	–	–	–
F8	*	–	–	–	*	–	–	*
Fz	–	–	–	*	*	–	–	*

**Conclusions:** We revealed significant differences between groups of right- and left-handers. Effect of handedness on response inhibition is observed in task 2 in which participants suppress reactions on word “red”. This effect is expressed only on congruent stimuli (“red” in red font) in right leads F4, F8, Fz. Individuals with different types of lateral asymmetry use various cognitive resources for response inhibition that related to the right brain hemisphere.

**Acknowledgements**

The research was supported by the Russian Science Foundation (project #16-18-00066).

**References**

1.Criaud M, Boulinguez P: Have we been asking the right questions when assessing response inhibition in go/no-go tasks with fMRI? A meta-analysis and critical review. *Neurosci Biobehav Rev* 2013, **37 (1)**: 11–23.

2. Beratis IN, Rabavilas A, Papadimitriou GN, Papageorgiou C: Effect of handedness on the Stroop Colour Word task. *Laterality* 2010, **15(6)**: 597–609.

## P131 Contribution of short-term plasticity of the bipolar-ganglion synapse to the activity both in the normal and the degenerating rd1 retina

### Kanako Taniguchi^1^, Katsunori Kitano^2^

#### ^1^Graduate School of Information Science and Engineering, Ritsumeikan University, Kusatsu, Shiga 5258577, Japan; ^2^Department of Human and Computer Intelligence, Ritsumeikan University, Kusatsu, Shiga 5258577, Japan

##### **Correspondence:** Katsunori Kitano (kitano@ci.ritsumei.ac.jp)

*BMC Neuroscience* 2017, **18(Suppl 1)**:P131

The normal retina responds to the onset or offset of light stimuli, which are called ON or OFF responses, respectively. While the retina of the retinal degeneration rd1 mouse fails to respond to light stimuli due to loss of photoreceptors, the abnormal retina is found to exhibit spontaneous rhythmic activity at a low frequency (<10 Hz) even without light stimuli that is not observed in the normal retina. Experimental results thus far suggest that the spontaneous rhythmic activity could be attributed not to ganglion cells (GCs) responsible for retinal outputs, but to the upstream neuronal network including bipolar cells (BCs) and AII amacrine cells (AII-ACs) [1]. Based on the results, two potential mechanisms have been proposed; one arises from the property of a gap junction network not only between BCs and AII-ACs but also between AII-ACs [2] whereas the other does from the intrinsic property of AII-ACs [3]. In either case, the oscillatory activity is enhanced when the AII-ACs are hyperpolarized, suggesting that both AII-ACs and BCs would be more hyperpolarized in the rd1 retina than in the normal retina. As the presynaptic neuron (BC) is more depolarized, the synapse from the BC to the postsynaptic neuron (GC) is more activated, which implies that GC should be more activated in the normal retina than in the rd1 retina. Therefore, It should be solved why the normal retina does not show such an activity as well as how such spontaneous rhythmic activity is generated in the abnormal retina. In the present study, we studied the mechanism for the spontaneous rhythmic activity using a computational model of AII-AC, BC, and GC network. In particular, to solve the paradoxical phenomenon mentioned above, we incorporated a modified short-term plasticity model as the BC-GC synapse [4,5] because BCs do not generate action potentials. Even at a depolarized state, the synapse model was not　activated sufficiently because of short-term depression, suggesting that it would be one of the required properties to account for both the normal and abnormal neural activities in the retina. Our results imply that the retina would utilize rate and/or temporal coding for neural information processing of the retina.

**References**

1. Euler T, Schubert T: Multiple independent oscillatory networks in the degenerating retina. *Front Cell Neurosci* 2015, **9**:444.

2. Trenholm S Borowska J, Zhang J, Hoggarth A, Johnson K, Barnes S, Lewis TJ, Awatramani GB: Intrinsic oscillatory activity arising within the electrically coupled AII amacrine-ON cone bipolar cell network is driven by voltage-gated Na^+^ channels. *J Physiol* 2012, **590**:2501–2517.

3. Choi H, Zhang L, Cembrowski MS, Sabottke CF, Markowitz AL, Butts DA, Kath WL, Singer JH, Riecke H: Intrinsic bursting of AII amacrine cells underlies oscillations in the rd1 mouse retina. *J Neurophysiol* 2014, 112:1491–1504.

4. Sagdullaev BT, Eggers E, Purgert R, Lukasiewicz PD: Nonlinear interactions between excitatory and inhibitory retinal synapses control visual output. *J Neurosci* 2011, **31**:15102–15112.

5. Tsodyks MV, Markram H: The neural code between neocortical pyramidal neurons depends on neurotransmitter release probability. *Proc Natl Acad Sci* 1997, **94**:719–723.

## P132 Differential connectomics of the rat thalamus

### Oliver Schmitt^1^, Felix Lessmann^1^, Sebastian Schwanke^1^, Peter Eipert^1^, Jennifer Meinhardt^1^, Julia Beier^1^, Kanar Kadir^1^, Adrian Karnitzki^1^, Linda Sellner^1^, Ann-Christin Klünker^1^, Lena Kuch^1^, Frauke Ruß^1^, Jörg Jenssen^1^, Andreas Wree^1^

#### ^1^Department of Anatomy, University of Rostock, Rostock, 18055, Germany

##### **Correspondence:** Oliver Schmitt (schmitt@med.uni-rostock.de)

*BMC Neuroscience* 2017, **18(Suppl 1)**:P132

The directed and weighted rat connectome is based on a metastudy of **all** published tract-tracing experiments. The connectivity data and relevant experimental data have been collated from 7351 papers until march 2017. Data were collated from experiments of normal postnatal rats of both sexes without lesions. Trans- or multisynaptic as well as collateral connectivity has been collated, too. The connectivity data were imported into the rat connectome project in the neuroVIISAS framework [1,3]. The stereotaxic atlas of Paxinos and Watson [2] in combination with a neuro-ontology represents the backbone of the connectome. All regions of the peripheral and central nervous system are organized in a 22 level hierarchy down to different types of neurons. The connectome is a multidimensional graph with regard to multiple weights that were found in the literature for particular connections. The total number of observations of connections is 955,195. 581,954 connections are singular (rejecting multiple observation). The connections are linking ipsilateral and contralateral regions. The regions of the thalamus of the stereotaxic atlas were used to filter all connections between them.

Any connectome that is derived from the whole 6*10^5^ rat connectome project is a *partial connectome*. The thalamic connectome is a partial connectome which consist of regions that are mapped in the stereotaxic atlas (see Figure 1b-c). The extrinsic regions of the thalamus have direct connections to the thalamic nuclei. They were filtered if they are located under hierarchy level 13 (the mean hierarchical level of thalamic regions is 11) and if the extrinsic regions have a degree larger 9.

The bilateral extrinsic thalamic connectome consists of 608 regions linked by 43079 connections. 9465 connections are reciprocal. The average degree is 142 and the line density 12%. The mean path length is 1.98 the average clustering coefficient is 0.42 and the small-worldness of 3.42 suggests a small-world architecture. The adjacency matrix (see Figure 1a) shows the bihemispheric frequency of connections. The stripe pattern belongs to ascending spinal projections.
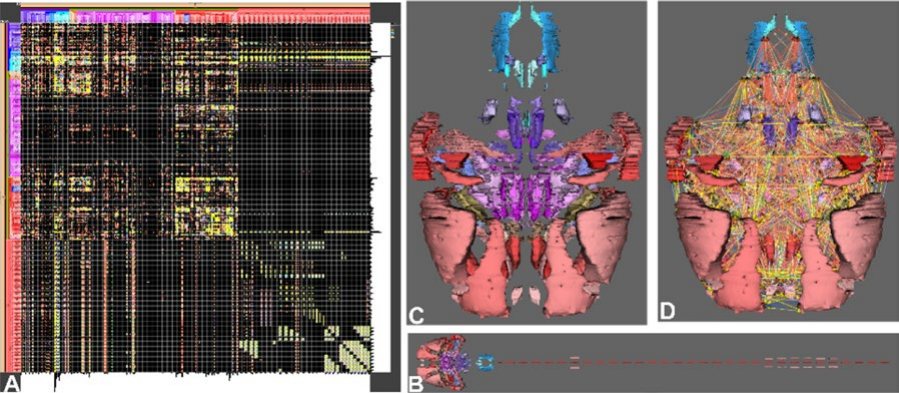


**Figure 1. A.** Adjacency matrix with frequency of connections. **B.** Regions with connections to the thalamic connectome. **C.** Expansion view of intracranial regions connected with the thalamus. **D.** 3D connectivity view

**Conclusions:** The rat intrinsic and extrinsic thalamic connectome has been characterized. The thalamic connectome is a multi-level–multidimensional directed and weighted network. Connectivity data show regular patterns at the level of the spinal cord (spinothalamic tract). The thalamic connectome was integrated into the stereotaxic atlas. The connectome has a small-world architecture and a relatively strong reciprocal connectivity whereas the intrinsic contralateral connectivity is sparse.

**References**

1. Schmitt O, Eipert P: neuroVIISAS: approaching multiscale simulation of the rat connectome. Neuroinformatics 2012, **10:** 243–267.

2. Paxinos G, Watson C: *The Rat Brain in Stereotaxic Coordinates, 7th Edition.* San Diego: Academic-Press; 2014.

3. neuroVIISAS framework [http://neuroviisas.med.uni-rostock.de/neuroviisas.shtml]

## P133 Nonuniform neural field modeling of nonlinear dynamics spreading

### Paula Sanz-Leon^1,2^, Stuart A. Knock^1,2^

#### ^1^School of Physics, University of Sydney, Sydney, New South Wales, Australia; ^2^Center for Integrative Brain Function, University of Sydney, Sydney, New South Wales, Australia

##### **Correspondence:** Paula Sanz-Leon (paula.sanz-leon@sydney.edu.au)

*BMC Neuroscience* 2017, **18(Suppl 1)**:P133

A neural field model of the corticothalamic system [1] is used to simulate neural activity on a curved surface as shown in the diagram in Fig. 1. An understanding of physically plausible mechanisms involved in the evolution and spreading of nonlinear activity requires the incorporation of realistic anatomical geometry to approximate the continuum medium on which neural activity propagates [2, 3]. Furthermore, the introduction of spatial support allows for the modeling of non-homogenous media. These two features are particularly important to understand epilepsy, for instance, where seizures may originate from isolated regions of restricted spatial extent. Previous work [2] showed that for spatially uniform parameters, the model presented here reproduces the results from [4] where an abstract approximation of the cortical sheet had been used (e.g., a square grid). We also showed that for the Laplacian operator acted as a spatial averaging filter and prevented the onset of seizures, despite all other parameters being consistent with an absence seizure state in the original model. Here, we study the evolution of nonlinear dynamics for different sizes of a patch of tissue that may exhibit oscillatory activity while the surrounding cortex is set to a linearly stable state (i.e., fixed point dynamics). We conclude that the interaction the area of the “anomalous” patch, in conjunction with the wave propagation velocity, determines whether (a) a seizure occurs and (b) whether it generalizes or not. These preliminary results are consistent with previous nonuniform neural field approaches [5]. However, unlike their work we study propagation on the convoluted geometry of the cortex, which can be directly extracted from subjects’ structural data and adds unmatched biophysical realism to the model.
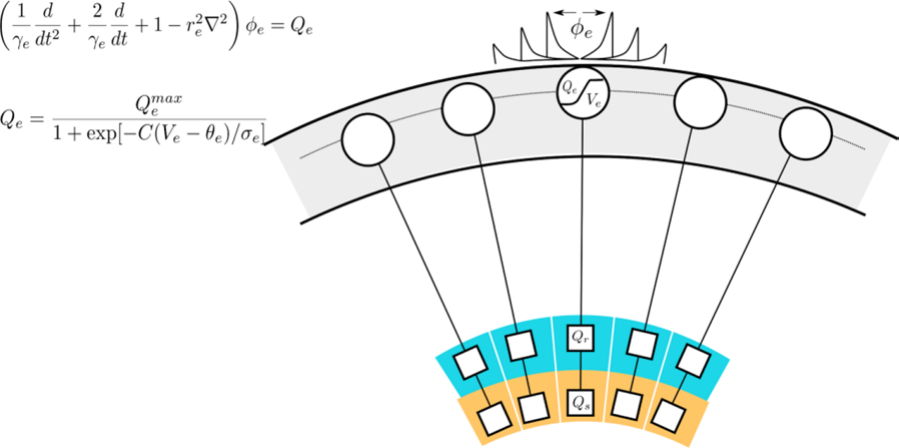


**Figure 1.** Corticothalamic model and propagation of nonlinear activity on the curved surface

**References**

1. Robinson PA, Rennie CJ and Rowe DL. Dynamics of large-scale brain activity in normal arousal states and epileptic seizures, *Physical Review E* 2002, vol. **65**, no. 4, pp. 041924 (1-9).

2. Sanz-Leon P and Knock SA. An Implementation Of Neural Fields On The Cortical Surface. *IWSP7* 2015 Melbourne - Conference paper.

3. Sanz-Leon P, Knock SA, Spiegler A, Jirsa, V. K. Mathematical framework for large-scale brain network modeling in The Virtual Brain, *NeuroImage* 2015, vol. **111**, no. May 2015, pp. 385–430

4. Breakspear M, Roberts JA, Terry JT, Rodrigues S, Mahant N, Robinson, PA. A unifying explanation of primary generalized seizures through nonlinear brain modeling and bifurcation analysis, *Cerebral Cortex* 2006, vol. **16**, no., 9, pp. 1296–1313

5. Kim JW, Roberts JA, Robinson PA. Dynamics of epileptic seizures: evolution, spreading and suppression. *J. Theor. Biol. 2009*. vol **257**, pp. 527–532

## P134 Excitatory-to-inhibitory plasticity for sequence learning

### Shih-Cheng Chien^1^, Burkhard Maess^1^, Thomas R. Knösche^1^

#### ^1^Max Planck Institute for Human Cognitive and Brain Sciences, Leipzig, 04103, Germany

##### **Correspondence:** Shih-Cheng Chien (vchien@cbs.mpg.de)

*BMC Neuroscience* 2017, **18(Suppl 1)**:P134

The generation of sequential neural activities takes place across different levels of sensory, motor, and cognitive systems. The stable heteroclinic channel (SHC) [1] provides a possible sequence generation mechanism, which relies on the existence of unbalanced inhibitory connections in the network. For learning and recalling a new sequence, the inhibitory connections in an SHC network should be able to alter with respect to the input stimuli. In this study, we investigated the role of the excitatory-to-inhibitory (EI) plasticity on SHC formation for sequence learning.

For our simulations, several nodes (Wilson-Cowan models [2]) were initially fully connected with excitatory-to-excitatory (EE) and excitatory-to-inhibitory (EI) connections. The input (e.g., tones of different pitch plus background activity) were fed to the E population of different nodes. We examined the effect of EI plasticity on SHC formation by keeping the EE connections fixed. The learning rule for EI plasticity was adapted from the differential anti-Hebbian learning rule [3] which was based on the observations that the spike-time dependent plasticity (STDP) of EI connections follows an opposite STDP window as compared to EE connections. For instance in the case of excitatory synapses onto inhibitory neurons, a pre-to-post pairing induces long-term depression (LTD) and a post-to-pre pairing causes long-term potentiation (LTP) [4].

In the simulation (Figure 1), the network with initially symmetric EI connections showed no sequential activities under background activity. After receiving a couple of repeated sequences, the matrix of EI connections altered and became asymmetric and an SHC emerged. The network activities then persisted in the same order as the learned sequence for a short moment in time. The network was able to reorganize and learn new sequences. This result showed that the EI plasticity based on the differential anti-Hebbian learning rule facilitates SHC formation in the network of connected E and I populations. Future study will include both EI and EE plasticity and examine their interactive effect on SHC formation for sequence learning.
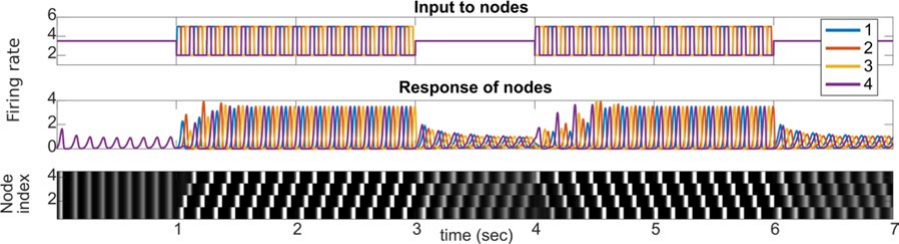


**Figure 1.** Example of a four-node network with EI plasticity during sequence learning. The network learned a first sequence (repeated 1-2-3-4) and recalled the sequences what the input was turned off at t = 3 s. A second sequence (repeated 4-3-2-1) was then learned and recalled at t = 6 s

**References**

1. Rabinovich MI, Huerta R, Varona P, Afraimovich VS: Generation and reshaping of sequences in neural systems. *Biol Cybern* 2006, **95**(6):519–536.

2. Wilson HR, Cowan JD: Excitatory and inhibitory interactions in localized populations of model neurons. *Biophys J* 1972, **12**(1):1–24.

3. Xie X, Seung HS: Spike-based learning rules and stabilization of persistent neural activity. 2000.

4. Bell CC, Han VZ, Sugawara Y, Grant K: Synaptic plasticity in a cerebellum-like structure depends on temporal order. *Nature* 1997, **387**(6630):278–281.

## P135 Experimental and modelling evidence for coaxial conduction in myelinated axons

### Charles C. Cohen^1,2^, Marko A. Popovic^1^, Jan Klooster^3^, Maarten H.P. Kole^1,2^

#### ^1^Axonal Signalling Group, Netherlands Institute for Neuroscience, Royal Netherlands Academy for Arts and Sciences (KNAW), Meibergdreef 47, 1105 BA Amsterdam, the Netherlands; ^2^Cell Biology, Faculty of Science, Utrecht University, Padualaan 8, 3584 CH, Utrecht, The Netherlands

##### **Correspondence:** Charles C. Cohen (c.cohen@nin.knaw.nl)

*BMC Neuroscience* 2017, **18(Suppl 1)**:P135

The development of discretized cable models constrained by cellular morphology and electrophysiology has revolutionized our understanding of neural computation, in particular for dendritic integration. In comparison, action potential (AP) propagation in the primary axon beyond initiation at the axon initial segment remains poorly understood. The common explanation for the rapid saltatory conduction of APs in myelinated axons is the single cable model, in which axon and myelin combine to form a single insulating membrane between nodes of Ranvier to rapidly propagate internodal potentials. Voltage responses at nodes, consequently, are expected to occur after preceding internodal ones [1]. In contrast, more recent optical voltage recordings of APs in myelinated axons show nodal potentials occurring before preceding or proceeding internodal ones [2,3], indicating saltation also occurs temporally. We hypothesized these results could be explained by the addition of a longitudinal resistance around internodal axon membrane, decelerating internodal potentials with respect to nodal ones by forming a local submyelin current return pathway. Thus, submyelin internodal current would flow more slowly with this additional resistance in its return path, whereas nodal current would flow faster, due to the near-absence of resistance in its return pathway through the extracellular space. Computational models including periaxonal conduction, known as double cable, show a better representation for certain AP features, such as the afterdepolarization [4]. However, whether submyelin conduction occurs, or is negligible, remains to be explored. By combining simultaneous whole-cell dual axo-somatic electrophysiological recordings and cable modelling with optical voltage recordings and electron microscopy (EM) of neocortical myelinated axons, we show that submyelin periaxonal conduction is necessary and sufficient for the saltatory conduction of APs. We leveraged the NEURON platform with a genetic algorithm we developed to robustly model unique solutions for our electrophysiological recordings with corresponding morphological constraints. The results showed that a double cable internodal circuit accounted for the observed axonal response, including temporal saltation, with near-absolute minimum error. We further developed equations for predicting the ultrastructure of the double cable internode, such as number of myelin membranes and periaxonal dimension, which we verified with the EM of recorded axons. Additional modelling revealed that a narrow submyelin space generates the temporal saltation of nodal potentials, as well as the attenuation of internodal ones throughout the internode. Taken together, we propose that coaxial conduction in the axon core and periaxonal space is imperative for accurately representing the fundamental axo-myelin circuit, and forms the basis for the rapid saltatory propagation of APs in myelinated axons.

**References**

1. Huxley AF, Stämpfli R: Evidence for saltatory conduction in peripheral myelinated nerve fibres. *J Physiol* 1949, **108:**315–39.

2. Palmer LM, Stuart GJ: Site of Action Potential Initiation in Layer 5 Pyramidal Neurons. *J Neurosci* 2006, **26:**1854–63.

3. Popovic MA, Foust AJ, McCormick DA, Zecevic D: The spatio-temporal characteristics of action potential initiation in layer 5 pyramidal neurons: a voltage imaging study. *J Physiol* 2011, **589:**4167–87.

4. McIntyre CC, Richardson AG, Grill WM: Modeling the excitability of mammalian nerve fibers: influence of afterpotentials on the recovery cycle. *J Neurophysiol* 2002, **87**:995–1006.

## P136 GIMBL-Vis: A GUI-Based Interactive Multidimensional Visualization Toolbox for Matlab

### Erik A. Roberts^1^, Nancy J Kopell^2^

#### ^1^Department of Biomedical Engineering, Boston University, Boston, MA 02215, USA; ^2^Department of Mathematics and Statistics, Boston University, Boston, MA 02215, USA

##### **Correspondence:** Erik A. Roberts (erob@bu.edu)

*BMC Neuroscience* 2017, **18(Suppl 1)**:P136

Computational models of neural circuits exhibit diverse behaviors, including many that are unexpected or difficult to predict [1]. Numerical simulations are useful for characterizing the dynamics of these high-dimensional systems. An increasingly vexing issue is the difficulty in visualizing and analyzing how properties of these models change as multiple independent parameters are varied in simulations. Here we introduce GIMBL-Vis, a Matlab toolbox for visualization of such multidimensional datasets [2]. This toolbox simplifies the process of exploring multidimensional data in Matlab by providing a graphical interface for visualization and analysis. It implements two different approaches for visualization: (1) dimensionality reduction provides useful information about the overall structure of the data [3]; (2) the ability to scroll through two-dimensional slices of a dataset reveals the effects of varying specific parameters. The toolbox is a useful complement for neural simulators, including our group’s recently developed Dynamic Neural Simulator (DynaSim) [4,5]. Furthermore, since it supports datasets in table form, it may be used for diverse sources of data.

**Acknowledgements**

We are grateful for the helpful feedback from lab members Michelle McCarthy, Ben Polletta, Austin Soplata, Dave Stanley, and Jason Sherfey.

**References**

1. Izhikevich EM. *Dynamical systems in neuroscience*. MIT Press; 2007.

2. GIMBL-Vis. [https://github.com/erik-roberts/GIMBL-Vis].

3. Van Der Maaten L, Postma E, Van den Herik J. Dimensionality reduction: a comparative review. *J Mach Learn Res* 2009, 10:66–71.

4. Sherfey JS, Kopell N. Dynamic Neural Simulator - a simple tool for rapidly building and sharing large neural models. *SFN* 2014.

5. DynaSim. [https://github.com/DynaSim/DynaSim].

## P137 Neural relativity principle

### Daniel Kepple^1^, Hamza Giaffar^1^, Dima Rinberg^2^, and Alex Koulakov^1^

#### ^1^Cold Spring Harbor Laboratory, Cold Spring Harbor, NY 11724, USA; ^2^New York University, New York, NY 10012, USA

##### **Correspondence:** Alex Koulakov (koulakov@cshl.edu)

*BMC Neuroscience* 2017, **18(Suppl 1)**:P137

Sensory systems are constantly facing the problem of computing the stimulus identity that is invariant with respect to several features. In the olfactory system, for example, odorant percepts have to retain their identity despite substantial variations in concentration, timing, and background. This computation is necessary for us to be able to navigate in chemical gradients or within variable odorant plumes. How can the olfactory system robustly represent odorant identity despite variable stimulus intensity? We propose a novel strategy for the encoding of intensity-invariant stimulus identity that is based on representing relative rather than absolute values of the stimulus features. We propose that, once stimulus features are extracted at the lowest levels of the sensory system, the stimulus identity is inferred on the basis of their relative amplitudes. Because, in this scheme, stimulus identity depends on relative amplitudes of features, identity becomes invariant with respect to variations in intensity and monotonous non-linearities of neuronal responses. For example, in the olfactory system, stimulus identity can be represented by the identities of p strongest responding odorant receptor types out of 1000. We show that this information is sufficient to ensure the robust recovery of a sparse stimulus (odorant) via l1 norm or elastic net loss minimization. Such a minimization has to be performed under the constraints imposed by the relationships between stimulus features. We map this problem onto a dual problem of minimization of a functional of Lagrange multipliers. The dual problem, in turn, can be solved by a neural network whose Lyapunov function represents the dual Lagrangian. We thus propose that the networks in the piriform cortex computing odorant identity implement dual computations with the sparse activities of individual neurons representing the Lagrange multipliers. Our theory yields predictions for the structure of olfactory connectivity.

## P138 Disrupted cerebrum-cerebellum network in Schizophrenia revealed by network-based statist and graph theory

### Caroline Garcia Forlim^1^, Leonie Klock^1,2^,Johanna Bächle^2^, Laura Stoll^2^, Patrick Giemsa^2^, Marie Fuchs^2^, Nikola Schoofs^2^, Christiane Montag^2^, Jürgen Gallinat^1^, Simone Kühn^1^

#### ^1^Clinic and Policlinic for Psychiatry and Psychotherapy, University Medical Center Hamburg-Eppendorf, Hamburg, 20246, Germany; ^2^Charité University Medicine and St. Hedwig-Krankenhaus, Department of Psychiatry and Psychotherapy, Berlin, 10115, Germany

##### **Correspondence:** Caroline Garcia Forlim (c.garcia-forlim@uke.de)

*BMC Neuroscience* 2017, **18(Suppl 1)**:P138

Schizophrenia is a mental illness that is characterized by heterogeneous symptoms ranging from positive symptoms such as delusions and hallucination to negative symptoms such as lack of motivation and reduced emotional expression among others. It has been suggested that schizophrenia is related to disrupted brain connectivity. Graph theoretical studies have found abnormal structural and functional in multiple brain networks supporting the theory of dysconnectivity syndrome [1] and suggesting wide-range of connectivity disturbances [2]. Nevertheless, there is no consensus regarding localized mechanisms and their associated symptoms. The cerebellum, an often overlooked region, might play a key role in schizophrenia. It has been suggested that a misconnection between cerebellum and cortex can lead to a misinterpretation of the information arriving from the cortex, resulting in, for example, experiences of delusion and auditory hallucinations [3].

Here, we first extract a functional subnetwork from resting state fMRI of schizophrenia patients and healthy controls using network-statistic approach and then applied graph measures. Network-based statistic method is a new nonparametric cluster statistic, to look for differences in the subnetwork brain wiring. In order to relate functional connectivity and graph measures to psychopathology, we correlated these measures with clinical symptoms scales. Furthermore, we sought to relate these measures to state aspects of clinical symptoms and presented participants eleven questions concerning their subjective experience during the resting state immediately after the measurement.

We found a single disrupted subnetwork in cerebrum-cerebellum mainly comprising brain regions related to visual processing and the cerebellum. More precisely: inferior and superior occipital, lingual, cuneus, fusiform, thalamus and Vermis. The density of the connections was higher in the right hemisphere. Schizophrenia patients presented higher functional connectivity, strength, global efficiency and betweenness than the healthy control participants, showing increased information processing. The regions that mainly control the information flow in the subnetwork are vermis 9 and inferior occipital, followed by vermis 7 in healthy controls whereas by superior occipital controlled the information flow in patients with schizophrenia. All disrupted connections were in cerebrum-cerebellum, especially occipital lobe-vermis, except those from inferior/superior occipital-thalamus.

Regarding the relationship of these measures with psychopathology, we did not find an association with any of the symptom scales but observed that all graph measures of this subnetwork were anti-correlated with reports of being externally influenced during the resting state measurement.

These findings reinforce the role of the occipital lobe and cerebellum in schizophrenia and it is consistent with structural imaging studies that showed alteration in the cerebellum [4,5] and in the occipital lobe[4].

**Acknowledgements**

This work was funded by SFB 936.

**References**

1. Hadley JA, Kraguljac NV, White DM, Ver Hoef L, Tabora J, Lahti AC, et al.: Change in brain network topology as a function of treatment response in schizophrenia: a longitudinal resting-state fMRI study using graph theory. *Schizophr* 2016, **2**:16014.

2. van den Heuvel MP, Fornito A: Brain Networks in Schizophrenia. *Neuropsychol Rev* 2014, **24**:32–48.

3. Andreasen NC, Pierson R: The Role of the Cerebellum in Schizophrenia. *Biol Psychiatry* 2008, **64**:81–8.

4. Chuang J-Y, Murray GK, Metastasio A, Segarra N, Tait R, Spencer J, et al.: Brain Structural Signatures of Negative Symptoms in Depression and Schizophrenia. *Front Psychiatry* 2014, **5**:116.

5. Okugawa G, Nobuhara K, Sugimoto T, Kinoshita T. Diffusion tensor imaging study of the middle cerebellar peduncles in patients with schizophrenia. *Cerebellum* 2005, **4**:123–7.

## P139 Computational behavioral analysis of acute psychosocial trauma reveals gradually increasing stress reactions in adult mice

### Ray X. Lee^1,2^, Greg J. Stephens^2,3^, Bernd Kuhn^1^

#### ^1^Optical Neuroimaging Unit, Okinawa Institute of Science and Technology Graduate University (OIST), Okinawa 904-0495, Japan; ^2^Biological Physics Theory Unit, Okinawa Institute of Science and Technology Graduate University (OIST), Okinawa 904-0495, Japan; ^3^Department of Physics and Astronomy, Vrije Universiteit Amsterdam, NL-1081 HV, Amsterdam, The Netherlands

##### **Correspondence:** Ray X. Lee (rayxin.lee@oist.jp)

*BMC Neuroscience* 2017, **18(Suppl 1)**:P139

Acute psychological trauma, particularly for a subjectively painful event involving interpersonal relationship, has been known to be a potent resource of mental disorders which develop through a temporal delay under neglect [1], yet our understanding of its neural substrate is limited to retrospective reports and case studies where informative behavioral details were poorly captured [2, 3]. Here we developed a novel and general computational tool to directly analyze the probability distributions of spontaneous behavior in a steady environment. We used this tool as a readout of an animal’s internal states such as emotion, and investigated the psychological development after an acute psychosocial trauma in laboratory mice (Fig. 1). The focal mice were isolated after observing a traumatic event that happened to their pair-housed partners. A group of control mice isolated without the traumatic experience were compared. Based on the special symmetry of the designed experimental environments, we analyzed the cumulative probability distribution of the time spent at locations along a one-dimensional stressor-included to stressor-free axis. Instead of merely analyzing the distances between cumulative probability distributions (e.g. two-sample Kolmogorov-Smirnov test), we considered individual variances and analyzed the statistically significant levels of the distances. While behavioral variables in classical measures showed delayed differences between control and traumatized mice, long-term and gradually increasing differences in the spatial probability distributions were initiated right after the traumatic event. The increasing behavioral differences also showed up in the independent behavioral index of locomotor speed under the statistical analysis of probability distributions, but not in the mean speeds. Moreover, these developing patterns were not correlated with those of acute stress reactions which gradually decreased and recovered in late post-traumatic period, together supporting an independent internal process after acute psychosocial trauma. These results suggest a potential neural mechanism of post-traumatic stress incubation through the slow dynamics of neural plasticity arisen spontaneously from intrinsic network activity.
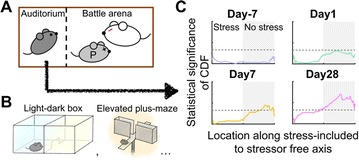


**Figure 1.** Schematic of computing internal effects from emotion on behavioral probability distributions that revealed psychological development before delayed stress reactions in traumatized mice. **A.** Witnessing trauma induction. Focal mouse (dark gray), partner (light gray P), aggressor (white). **B.** Behavioral tests of anxiety. **C.** Fine-scale analysis along subjective stressor gradient on days before/after trauma; CDF, cumulative distribution function

**Acknowledgements**

We acknowledge JSPS KAKENHI Grant (JP 16J10077) to R.X.L. and OIST internal funding to B.K.

**References**

1. Association AP: Diagnostic and statistical manual of mental disorders (DSM-5^®^): American Psychiatric Pub; 2013.

2. Gilbertson MW, Shenton ME, Ciszewski A, Kasai K, Lasko NB, Orr SP, Pitman RK: Smaller hippocampal volume predicts pathologic vulnerability to psychological trauma. *Nature neuroscience* 2002, **5**(11):1242–1247.

3. Reynaud E, Guedj E, Trousselard M, El Khoury-Malhame M, Zendjidjian X, Fakra E, Souville M, Nazarian B, Blin O, Canini F: Acute stress disorder modifies cerebral activity of amygdala and prefrontal cortex. *Cognitive neuroscience* 2015, **6**(1):39–43.

## P140 Relationship between population sparse coding and short-term synaptic plasticity

### Luiz Tauffer^1,2^, Philippe Isope^3^, Arvind Kumar^2^

#### ^1^Bernstein Center Freiburg, University of Freiburg, Freiburg, Germany; ^2^School of Computer Science and Communication, KTH Royal Institute of Technology, Stockholm, Sweden; ^3^CNRS, University of Strasbourg, Strasbourg, France

##### **Correspondence:** Luiz Tauffer (luiz.tauffer@bcf.uni-freiburg.de)

*BMC Neuroscience* 2017, **18(Suppl 1)**:P140

Short-term synaptic plasticity (STP) refers to an increase (facilitation) or decrease (depression) in the synaptic efficacy depending on the history of presynaptic spiking activity [1]. STP is ubiquitous in the brain and is believed to play an important role in information transfer [1]. Furthermore, computational models have suggested that STP may also underlie population synchronization [4], working memory [5] and the cortical up and down states [6].

Here, using numerical simulations, we investigated how STP imposes constrains on distribution of spiking activity over a group of presynaptic neurons to maximize the information transmission. To this end, we searched for how a certain number of extra spikes (occurring in a small time window) carrying an information should be distributed over the population of presynaptic neurons in order to maximize the Proportion of Released Vesicles (PRV). We found that for a fixed number of extra spikes, PRV changed in a non-monotonic fashion as a function of number of recruited presynaptic neurons (Fig 1A). Furthermore, the number of input neurons required to maximize PRV increased linearly as a function of number of extra spikes (Fig. 1B). Finally, PRV was maximized for a fewer number of presynaptic neurons when synapses exhibited short-term-facilitation as compared to when synapses exhibited short-term-depression (Fig. 1B).

These results suggest that STP of the synapses sets an optimum firing rate for each presynaptic neuron, in a given ensemble, to maximize the PRV. Therefore, an optimum spatial distribution of extra spikes is the one that brings the firing rate of each presynaptic neuron to its optimum firing rate. Our model predicts that in brain areas where synapses predominantly show short-term-facilitation stimulus related spiking activity should be confined to a few high firing rate neurons and vice versa when synapses predominantly show short-term-depression. In summary, we show that there is a close relationship between sparse representation in population coding and STP features.
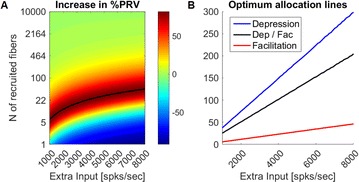


**Figure 1. A.** the PRV of a population changes according to the number of recruited fibers. Black line marks the optimum number of fibers to be recruited in a Facilitation dominated regime. **B.** Optimum allocation lines for three STP regimes, Depression (blue, 37 Hz), Depression/Facilitation (black, 48 Hz) and Facilitation (red, 100 Hz)

**References**

1. Rotman Z, Deng PY, Klyachko VA: Short-term plasticity optimizes synaptic information transmission. *J Neurosci* 2011; **31:** 14800–14809.

2. Tsodyks M, Markram H: Neural networks with dynamic synapses. *Neural Comput* 1998; **10:**821–835.

3. Hennig MH: Theoretical models of synaptic short term plasticity. *Front. Comput. Neurosci*. 2013; **7:**45.

4. Tsodyks, M., Uziel, A., and Markram, H: Synchrony generation in recurrent networks with frequency-dependent synapses. *J. Neurosci.* 2000; **20**, RC50.

5. Mongillo G, Barak O, Tsodyks M: Synaptic theory of working memory. *Science.* 2008; **319(5869):**1543–1546.

6. Holcman D, Tsodyks M: The emergence of up and down states in cortical networks. *PLoS Computational Biology.* 2006; **2(3):**e23.

## P141 The contribution of topology for inclusion of feedforward network and biased synaptic strength to the long-term memory effect in a cortical microcircuit

### Katsuma Inoue^1^, Yoshiyuki Ohmura^1^, Shogo Yonekura^1^, Yasuo Kuniyoshi^1^

#### ^1^Department of Mechano-Informatics, University of Tokyo, Bunkyo, Tokyo, Japan, 113-8656

##### **Correspondence:** Katsuma Inoue (k-inoue@isi.imi.i.u-tokyo.ac.jp)

*BMC Neuroscience* 2017, **18(Suppl 1)**:P141

The neural network in a cortical microcircuit is inherently structured [1, 2]. These structures may contribute to environmental adaptability and perceptual motor learning. However, little is known about the effect of these structures of network topology and synaptic rule on information processing. In this study, to clarify the influence of cortical structures on network function, we constructed microcircuit model based on the cortical organization rule in a juvenile rodent and analyzed its activity. Moreover, we evaluated the memory performance with a liquid state machine (LSM) [3], which is a framework for computation that utilizes a spiking neural network.

**Materials and Methods:** *Network topology* There are four main features pertaining to network topology in a cortical microcircuit [1, 2]. (a) Degree distribution has long tail. (b) Connection probability between any two neurons increases with the number of common neighbors (CNs). (c) More feedforward sub-graphs are contained compared to random network. (d) Higher reciprocity is observed compared to random networks. We determined the model in which the number of degree follow a lognormal distribution independently satisfies these four features the most. Therefore, we adopted a “lognormal” model and compared this model to a random network. *(2) Synaptic rule:* It has also been reported that the amplitudes of excitatory postsynaptic synaptic potentials (EPSPs) in any two neurons increase with the number of CNs [2]. To make the network satisfy this requirement, we generated EPSPs randomly following the Gamma distribution as well as arranged EPSPs with the number of CNs. Hence, we created an arranged condition and a randomly shuffled condition and there are four conditions (2*2) in our experiments (Table 1).

**Table:** **Table 1.** Experimental conditions

conditions	topology	EPSPs
logn_sorted	lognormal	arranged
logn_shuffle	lognormal	shuffled
random_sorted	random	arranged
random_shuffle	random	shuffled

**Results:** First, we measured the activity of each neuron in the network for 10 s. We gave constant input current to one neuron with the largest out-degree. The distribution in the “long_sorted” condition, which satisfies all requirements described above, is bimodal, whereas the distributions in other conditions are unimodal. This means that though the activities of some neurons increase, most neurons become inactive in “logn_sorted” condition. Secondly, we evaluated the performance of memory task with LSM (Figure 1). We gave non-periodic input to 10 neurons and trained the LMS to output average of past ΔT [ms] input signal. The mean square error of the “logn_sorted” is less than that of the other conditions when ΔT is larger than 90 [ms].
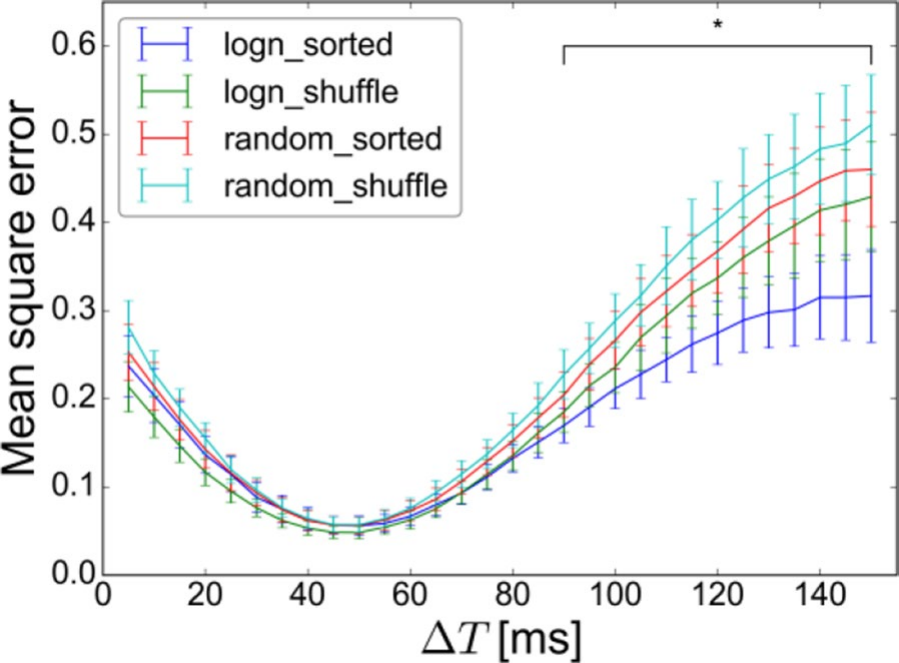


**Figure 1.** Performance of memory task. The “logn_sorted” condition has minimum value when ΔT > 90 [ms] (p < 10^−10^; Welch’s t-test)

**Conclusions:** We observed that neuron activities become bipolarized and some neurons with larger degree have long-term memories only when both network topology and synaptic are structured. These results suggest that both network topology and synaptic rules play important roles in the long-term memory effect and only active neurons contribute towards it.

**References**

1. Song S, Sjöström PJ, Reigl M, Nelson S, Chklovskii DB. Highly nonrandom features of synaptic connectivity in local cortical circuits. *PLoS Biol* 2005;**3:**0507–0519.

2. Perin R, Berger TK, Markram H. A synaptic organizing principle for cortical neuronal groups. *PNAS* 2011;**108:**5419–5424.

3. Maass W, Natschläger T, Markram H. Real-Time Computing Without Stable States: A New Framework for Neural Computation Based on Perturbations. *Neural Comput* 2002;**14:**2531–2560.

## P142 Propagation of spatio-temporally complex spike pattern in feedforward network of the barrel cortex: in vitro multi-electrode array and in silico neural network model study

### Hyun Jae Jang and Jeehyun Kwag

#### Department of Brain and Cognitive Engineering, Korea University, Seoul, Korea

##### **Correspondence:** Jeehyun Kwag (jkwag@korea.ac.kr)

*BMC Neuroscience* 2017, **18(Suppl 1)**:P142

Somatosensory information of the external world detected by whiskers of rodents is translated into spatio-temporally complex neuronal spikes in the vibrissal primary somatosensory cortex (barrel cortex) [1]. For effective somatosensory information processing, the information contained within such complex spike pattern should be preserved in downstream neurons in the feedforward network (FFN) of the barrel cortex [2]. Through multi-electrode array (MEA) recording of barrel cortical slice in vitro, we first investigated whether spatio-temporally complex spike patterns can reliably propagate in the feedforward network of the barrel cortex by stimulating layer 4 of the barrel cortex, the main recipient of thalamic sensory inputs [3]. We found that spatio-temporally complex spike pattern was preserved in downstream layers 2/3, 5 and 6. However, application of GABA_A_ receptor antagonist (gabazine, 5 μM) disrupted the spike pattern propagation, indicating that inhibition gates the propagation of spatio-temporally complex spike pattern. To further investigate the inhibitory network mechanism underlying spike pattern propagation, we constructed a four-layer in silico FFN model composed of 200 Hodgkin-Huxley excitatory neurons and 50 interneurons in each layer and found that differential recruitment of distinct inhibitory network structures is required for the reliable propagation of spatio-temporally complex spike pattern. Our in vitro and in silico study provide evidence for the roles of inhibition in somatosensory information propagation in the barrel cortex.

**Acknowledgements**

This study was supported by Human Frontier Science Program (RGY0073/2015), the Basic Science Research Program (NRF-2016R1A1A05921614) and the Brain Research Program (NRF-2015M3C7A1028790) through the National Research Foundation (NRF) of Korea funded by the Ministry of Science, ICT and Future Planning.

**References**

1. Ramirez A, Pnevmatikakis EA, Merel J, Paninski L, Miller KD, Bruno RM: Spatiotemporal receptive fields of barrel cortex revealed by reverse correlation of synaptic input. *Nat Neurosci* 2014, **17**(6):866–875.

2. Kumar A, Rotter S, Aertsen A: Spiking activity propagation in neuronal networks: reconciling different perspectives on neural coding. *Nature reviews Neuroscience* 2010, **11**(9):615–627.

3. Felleman DJ, Van Essen DC: Distributed hierarchical processing in the primate cerebral cortex. *Cereb Cortex* 1991, **1**(1):1–47.

## P143 Computational Geometry for the Simulation of Neural Circuits with Population Density Techniques

### Marc de Kamps^1^, Yi Ming Lai

#### School of Computing, University of Leeds, Leeds, LS2 9JT, UK

##### **Correspondence:** Marc de Kamps (M.deKamps@leeds.ac.uk)

*BMC Neuroscience* 2017, **18(Suppl 1)**:P143

Large-scale neuronal networks are often modeled at the population level, rather than by generating a large number of individual neural model instances. Using techniques from statistical physics it is possible to model population level quantities such as firing rate and membrane potential distributions in terms of the dynamics of individual neurons. Unlike rate based models, as long as the populations are homogeneous, the results will be comparable to those of spiking neuron simulators. In the past, most work has been done on populations of one-dimensional point model neurons. Here, we present a method for two-dimensional neuronal models, a generalization from [1,2], which makes it possible to model populations of conductance based (single channel), adaptive-exponential-integrate-and-fire (AdExp) neurons, or Izhikevich neurons. The method is universal in the following sense: prior to simulation the modeler creates a state space grid, which is presented to a simulator that we developed (MIIND; http://miind.sf.net). The simulator calculates transition matrices for a given set of synaptic efficacies, and allows the creation and simulation of networks of neuronal populations described by a state space grid, and then to simulate network dynamics. This makes it possible to incorporate neuronal models that are completely novel, or to extend existing models to a new dynamical range. Since the method is agnostic about the neuronal dynamics and only operates on the grid, it is universal in the sense that if a good grid can be made, the method will work. An example for AdExp neurons is given in Figure 1.

We will show that models that contain strong non-linearities often end up with small regions of state space where the mesh is distorted or breaks up, but that in general the movement of neural mass in small problematic areas can be inferred from the movement of mass outside of them, and that very little bias is produced in the overall simulation results. We will demonstrate this on populations of AdExp and Fitzhugh-Nagumo (FN) neurons. We will show that in AdExp neurons, for the right parameters, strong resonances will be produced if the right combination of excitatory and inhibitory stimuli is presented. For FN neurons, we will demonstrate that within one mesh one can model the disappearance and emergence of a limit cycle, and study the effect of diffusion –with and without bias- on the limit cycle. This is an important demonstration of the flexibility of the method. We believe these examples show that the method is broadly applicable and a good alternative to rate based models when the structure of the population state cannot be captured in a single variable.
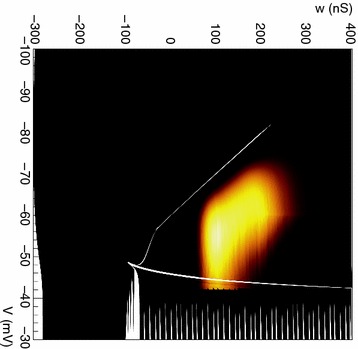


**Figure 1.** A heat plot of the probability density function of a population of AdExp neurons receiving Poisson distributed input spikes. The horizontal axis represents the membrane potential in mV, the vertical axis the adaptation values w in nS. Some neurons have just spiked and reappear at high values of w

**Acknowledgements**

This project has received funding from the European Union’s Horizon 2020 research and innovation programme under grant agreement No 720270 (HBP SGA1).

**References**

1. de Kamps M: A Generic Approach to Solving Jump Diffusion Equations with Applications to Neural Populations, https://arxiv.org/abs/1309.1654

2. Iyer R, Menon V, Buice M, Koch C, Mihalas S: The Influence of Synaptic Weight Distribution on Neuronal Population Dynamics, *Plos Comp Bio* 2013, http://dx.doi.org/10.1371/journal.pcbi.1003248

## P144 Neuron classification in the Stomatogastric ganglion using voltage-sensitive dye imaging and signal processing tools

### Filipa dos Santos, K. P. Lam^1^, Peter Andras

#### School of Computing and Mathematics, Keele University, Newcastle-under-Lyme, ST5 5BG, UK

##### **Correspondence:** Filipa dos Santos (f.dos.santos@keele.ac.uk)

*BMC Neuroscience* 2017, **18(Suppl 1)**:P144

Voltage sensitive dye (VSD) imaging has been used in the past few decades in both in vivo and in vitro studies. Fast response VSD have been used to analyse small invertebrate neural networks [1,2] including the crustacean Stomatogastric Ganglion (STG). The STG has two central patter generator neural networks and consists of just 26 neurons in the *Cancer pagurus* [3]. Earlier work implemented the so-called *event*-*triggering average* procedure for neuron identification, which removes sharp changes associated with spikes making the classification of neurons harder [4,5]. Here we present the use of advanced signal processing tools to classify neurons on the basis of recorded VSD imaging data. *C. pagurus* were anaesthetized prior dissection in 20–30 min ice bucket; dissection was carried out following the procedures described by [6], including the desheating of the STG for better dye exposure. The dye (Di-4 ANEPPS) was applied inside the petroleum jelly well around the STG and left for incubation for 20 min; after this time, the well was removed and the dye washed out. A high-resolution (HR) image (376 × 252) was first taken to identify the cells followed by low-resolution (LR) image (40 × 28) (see figure 1A) series at time gap of 1.5 ms, for a total of 21,840 frames (32,76 s). The pixels representing each cell were chosen in the HR image and mapped to the LR image stack where the temporal time sequences were extracted. The multi-resolution procedure of the Sequential Single Spectrum Analysis was performed on the extracted time sequences for each cell, from which the pyloric rhythm (PR) was extracted where applicable [7]. The extracted PR was then compared with the latero-ventral nerve (lvn) pyloric rhythm signal obtained via extracellular recording. The extracted PR from each cell (slow wave) was studied and matched with the lvn signal, This enables the detection of the individual phase, and thus the classification of the respective neuron. Specifically, we looked for neurons matching the distinct phases of the tri-phasic pattern produced by the pyloric neurons, i.e. LP (lateral pyloric), PY (pyloric constrictor) and PD (pyloric dilator) phases. Figure 1B shows the tri-phasic pattern of the pyloric rhythm, illustrating how an individual cell’s activity is correlated with the lvn signal.
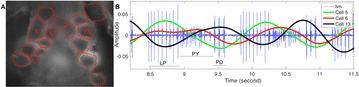


**Figure 1. A.** High-resolution image with cell identification. **B.** lvn recording (blue) with two pyloric cycles. Pyloric rhythm extracted from three different cells

**References**

1. S. Chemla and F. Chavane: Voltage-sensitive dye imaging: Technique review and models. *J Physiol Paris* 2010, **104:**40–50.

2. E. S. Hill, A. M. Bruno, and W. N. Frost: Recent developments in VSD imaging of small neuronal networks. *Learn Mem* 2014, **21:**499–505.

3. E. Marder and D. Bucher: Understanding circuit dynamics using the stomatogastric nervous system of lobsters and crabs. *Annu Rev Physiol*, 2007, **69:**1–12.

4. W. Stein, C. Städele, and P. Andras: Single-sweep voltage-sensitive dye imaging of interacting identified neurons. *J Neurosci Methods*, 2011, **194:**224–234.

5. C. Städele, P. Andras, and W. Stein: Simultaneous measurement of membrane potential changes in multiple pattern generating neurons using voltage sensitive dye imaging. *J Neurosci Methods*, 2012, **203:**78–88.

6. G. J. Gutierrez and R. G. Grashow: Cancer borealis Stomatogastric Nervous System dissection. *J Vis Exp*, 2009, 1–5.

7. F. dos Santos, P. Andras, and K. Lam: *A Multiresolution Approach to the Extraction of the Pyloric Rhythm from voltage*-*sensitive dye imaging data in the Stomatogastric Ganglion*. to present in *TSP/IEEE conference*, 2017.

## P145 Reconstructed attractors from optogenetics experiments

### Sorinel A. Oprisan^1^, Julia Imperatore^1^, Jessica Helms^1^, Tamas Tompa^2^, Antonieta Lavin^2^

#### ^1^Department of Physics and Astronomy, College of Charleston, Charleston, SC 29424, USA; ^2^Department of Neuroscience, Medical University of South Carolina, Charleston, SC 29425, USA

##### **Correspondence:** Sorinel A. Oprisan (oprisans@cofc.edu)

*BMC Neuroscience* 2017, **18(Suppl 1)**:P145

**Introduction:** Gamma oscillations show strong coherence across different areas of the brain during associative learning or during successful recollection. Gamma rhythm involves the reciprocal interaction between interneurons, mainly parvalbumin (PV+) fast spiking interneurons (FS PV+) and principal cells. The predominant mechanism for neuronal synchronization is the synergistic excitation of glutamatergic pyramidal cells and GABAergic interneurons. Nonlinear time series was previously used for investigating large-scale synchronization of activity that leads to epilepsy was diagnosed using nonlinear dynamics [1] and for disrupting synchronization based on phase response curve [2].

**Method:** We used optogenetic mice to record the local field potentials (LFPs) from medial prefrontal cortex (mPFC) in response to 473 nm laser light stimuli. We used a single, 10 ms duration, light pulse applied every 2 s and recorded with a sampling time ∆t = 10^−4^ s. Each trial was repeated 100 times and we only retained and analyzed data from six animals that showed stable and repeatable response to optical stimulations. For each trail, surrogate data sets were generated and both time reversal asymmetry and false nearest neighbor (FNN) were used as discriminating statistics for the null hypothesis. We used the delay embedding method to investigating the possibility of recovering phase resetting from single-cell recordings [3, 4].

**Results:** We used average mutual information (Fig. 1A) to determine the delay time and FNN (Fig. 1B) to estimate the embedding dimension. Although the shape of the attractors for control group were relatively stable and similar (Fig. 1C), we found significant changes under systemic amphetamine administration. Under amphetamines, the autocorrelation function gave a significantly smaller delay time for attractor embedding. This also correlates with a decrease in LFP noise under amphetamine.
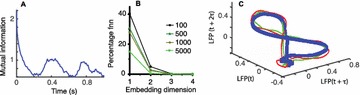


**Figure 1. A.** The first minimum of the average mutual information gives the delay time τ ≈ 2000∆t; **B.** The percentage of FNN estimated with variable Theiler window; **C.** Reconstructed 3D attractors

**Acknowledgements**

SAO acknowledges support for this research from NSF-CAREER award IOS 1054914.

**References**

1. Päivinen N, Lammi S, Pitkänen A, Nissinen J, Penttonen M, Grönfors T: Epileptic seizure detection: A nonlinear viewpoint. *Computer Methods and Programs in Biomedicine* 2005, **79**(2):151–159.

2. Greenberg BD, Gabriels LA, Malone DA, Jr., Rezai AR, Friehs GM, Okun MS, Shapira NA, Foote KD, Cosyns PR, Kubu CS et al.: Deep brain stimulation of the ventral internal capsule/ventral striatum for obsessive–compulsive disorder: worldwide experience. *Mol Psychiatry* 2010, **15**(1):64–79.

3. Oprisan SA, Thirumalai V, Canavier CC: Dynamics from a Time Series: Can We Extract the Phase Resetting Curve from a Time Series? *Biophysical Journal* 2003, **84**(5):2919–2928.

4. Oprisan SA, Lynn PE, Tompa T, Lavin A: Low-dimensional attractor for neural activity from local field potentials in optogenetic mice. *Frontiers in Computational Neuroscience* 2015, **9**(125).

## P146 A Simple Parameter Landscape for Optimisation of Conductance-Based Models of Neurons

### Felicity H. Inkpen^1^, Michael C Ashby^1^, Nathan F Lepora^2^

#### ^1^School of Physiology, Pharmacology and Neuroscience, University of Bristol, Bristol, BS8 1TD, UK; ^2^Department of Engineering Mathematics, University of Bristol, Bristol, BS8 1UB, UK

##### **Correspondence:** Felicity H. Inkpen (fi14240@bristol.ac.uk)

*BMC Neuroscience* 2017, **18(Suppl 1)**:P146

The action potential is fundamental to neural communication, instrumental to the release of neurotransmitters, and implicated in the formation of functional cell networks. The shape of the action potential and the properties of a neuronal spike train contain a wealth of information, being dependent on cell type, functionality, and developmental stage. By fitting computational conductance-based Hodgkin-Huxley style models to electrophysiological data, we can attempt to reveal the underlying biophysics behind the subtleties of the action potential shape, returning predictions of ion conductance channel populations and cell membrane properties. But, with multiple parameters to consider when modelling the action potential, accurate optimisation techniques are needed, and successful fitting requires not only a good model, but effective evaluation of the goodness of fit [1–3].

Voltage-based fitting methods, which compare the neuronal membrane potential from electrophysiology experiments to that modelled via the Hodgkin-Huxley equation, can produce complex parameter landscapes with local or narrow minima, requiring computationally expensive algorithms, such as stochastic or evolutionary optimisation, to return the conductances associated with different active and passive neuronal dynamics. By alternatively considering computation of the difference between passive and active neuronal currents, the optimisation can be reduced to a simple linear sum, in which a residual current error is minimised [4]. This results in a smooth parameter landscape, in which finding a single minimum is a trivial and computationally inexpensive problem to solve [5].

However, we find upon close examination of the residual current error for simulated neuron data that in previous methods [5] the minimum is displaced from the true value. This appears to be a consequence of numerically solving the differential equations for the channel kinetics. Here, we take a different approach, algebraically solving the differential equations that determine the gating variables behind the Hodgkin-Huxley conductances, computing the resulting passive and active currents via simple integration using trapezium rule. We demonstrate that these current-based methods make the error between model and target neuron simulation a trivial function with a single minimum, finding target conductance values with a high degree of accuracy, whilst voltage-based methods are less successful.

The minimisation of residual current method can be presented graphically for the different conductances of the Hodgkin-Huxley model in multidimensional yet simple parameter landscapes that allow for intuitive interpretation and fast model optimisation. Currently built on the basis of smooth artificial target data, we expect this technique to have increased robustness to noise, and to be effective for model neurons with multiple channels. Furthermore, we expect this method can be used as a simple probe of the changing biophysical properties of the developing neuron and help to quantify the biophysical heterogeneity of neuronal populations.

**References**

1. Van Geit W, De Schutter E, Achard P, Automated neuron model optimization techniques: a review. *Biological Cybernetics* 2008, **99**: 241–251.

2. Druckmann S, Banitt Y, Gidon A, Schürmann F, Markram H, Segev I, A novel multiple objective optimization framework for constraining conductance-based neuron models by experimental data. *Frontiers in Neuroscience* 2007, **1(1)**:7–18

3. Gerstner W, Naud R, How Good Are Neuron Models? *Science* 2009, **326(5951)**:379–380.

4. Morse, TM, Davidson AP, Hines M, Parameter space reduction in neuron model optimization through minimization of residual voltage clamp current, *31* ^*st*^ *Annual Meeting of the Society for Neuroscience, San Diego, CA, USA, 10*–*15 November 2001. Society for Neuroscience Abstracts* 2001, **27**

5. Lepora NF, Overton PG, Gurney K, Efficient fitting of conductance-based model neurons from somatic current clamp. *Journal of Computational Neuroscience* 2012, **32(1)**:1–24.

## P147 Neural Synchronization Through Electric Field Effects

### Aaron R. Shifman^1,2,3^, John E. Lewis^1,2,3^

#### ^1^Department of Biology, University of Ottawa, Ottawa, Ontario, K1N 6N5, Canada; ^2^Center for Neural Dynamics, University of Ottawa, Ottawa, Ontario K1N 6N5, Canada; ^3^Ottawa Brain and Mind Research Institute, University of Ottawa, Ottawa, Ontario K1N 6N5, Canada

##### **Correspondence:** Aaron R. Shifman (ashifman@uottawa.ca)

*BMC Neuroscience* 2017, **18(Suppl 1)**:P147

The ionic currents underlying the action potentials generated during regular brain activity give rise to an electric field. These electric fields permeate space and can create a voltage at a distance. In the context of the brain, these induced voltages reflect a change in the transmembrane potential of other nearby neurons, effectively coupling the neurons without any form of synaptic connection. Identified in the early 1940s, and known as ephaptic coupling, this electric field effect is nonspecific and diffuse; which is in direct contrast to traditional synaptic coupling (electrical or chemical). Thus, (with exception of a few well described circuits [1]), ephaptic coupling is generally assumed to play a negligible role in the brain. This is justified by the fact that the induced voltage scales with the resistance of the surrounding tissue, which has been estimated as being quite small [2,3]. However, we have shown that despite the low resistance, ephaptic coupling can influence neural dynamics in a significant way.

To avoid experimental complexity, ephaptic coupling is traditionally studied through the use of computational methods (for example see [4,5]). These methods require computationally expensive techniques which limit the type and scope of questions which can be studied. To reduce this complexity, we have created a novel, semi-analytic computational approach for modeling ephaptic coupling. By eliminating the (relative) bulk of the computational expense, we can ask more detailed questions about the role that ephaptic coupling plays in the brain. We use this new approach to study synchronization and phase locking in small (2–3) model neuron networks. We found that synchronization and phase locking was robust in these small networks, but the specific dynamics differed from what is typically seen with similar networks involving synaptic coupling. Synchrony in neural networks is of interest in many contexts, from pathologies (e.g. epilepsy) to learning and memory; our results suggest that ephaptic coupling adds richness to such dynamics and may be involved in their generation and control.

**References**

1. Furukawa T, Furshpan E: Two Inhibitory Mechanisms in the Neurons of Goldfish. *J. Neurophysiol*. 1963, **26(1)**:140–176.

2. Jefferys JGR: Nonsynaptic modulation of neuronal activity in the brain: electric currents and extracellular ions. *Physiol. Rev.* 1995, **75(4)**:689–723.

3. Miceli S, Ness TV, Einevoll GT, Schubert D: Impedance Spectrum in Cortical Tissue: Implications for Propagation of LFP Signals on the Microscopic Level. *eNeuro.* 2017, **4**:1.

4. Lin J, Keener JP: Modeling electrical activity of myocardial cells incorporating the effects of ephaptic coupling. *Proc. Natl. Acad. Sci. U.S.A.* 2010, **107(49)**:20935–20940

5. Anastassiou CA, Montgomery SM, Barahona M, Buzsaki G, Koch C: The Effect of Spatially Inhomogeneous Extracellular Electric Fields on Neurons. *J. Neurosci.*2010, **30(5)**:1925–1936

## P148 How Spines Cross-talk: Compartmental Model of Heterosynaptic Plasticity

### Zhong Zhang^1^, Yeqian Feng^1^, Christian Tetzlaff^3^, Tomas Kulvicius^4^, Yinyun Li^2^

#### ^1^Department of Management Science, Beijing Normal University, Beijing, 100875, China; ^2^School of Systems Science, Beijing Normal University, Beijing, 100875, China; ^3^Institute of Physics-Biophysics, Georg-August-University, Goettingen, 37077, Germany; ^4^Maersk Mc-Kinney Moller Institute, University of Southern Denmark, 5230 Odense, Denmark

##### **Correspondence:** Yinyun Li (yinyun@bnu.edu.cn)

*BMC Neuroscience* 2017, **18(Suppl 1)**:P148

Synaptic plasticity as the physiological fundamental basis for functions in nervous system has two forms of expression, homosynaptic and heterosynaptic plasticity [2,6,7]. Synapses that receive presynaptic stimulation could change synaptic transmission, which is referred to homosynaptic plasticity or Hebbian plasticity. Experimental evidences [1,8] demonstrate that synapses without any presynaptic stimulation could also induce changes, which is referred to heterosynaptic plasticity.

Given the functional significance [2,5–7], the biophysical mechanism of heterosynaptic plasticity induction is still an unsolved question. We hypothesize that calcium signal may mediate the ‘cross talk’ between neighboring synapses, and calcium from stimulated spine could diffuse into neighboring spines through dendritic shaft, and the coincidence detection of diffusive calcium signal with calcium influx triggered by back propagated action potentials (bAPs) may result in heterosynaptic potentiation or depression.

By extracting kinetic parameters of calcium decay in spine from experimental observation [3,4], our results are able to reproduce the calcium dynamics in spine and dendrites comparable to experimental observations [3,4]. Importantly, our model could predict a “Mexican hat” profile by high frequency stimulation, and a clustered Long Term Depression (LTD) region by low frequency stimulation. In addition, our model illustrates coordinated interactions of synaptic competition and cooperation, by stimulating two synapses simultaneously at differential locations along a common dendrite. The simulation results suggest that the compartmental model of heterosynaptic plasticity provides a promising mechanism for interpretation of cooperative and competitive interactions between synapses, and reveals a variety of abundance for synaptic function in neural circuits.

**Acknowledgements**

We would like to thank Prof. Florentin Woergoetter for helpful discussions. This work is supported by National Science foundation of China, Yong Scientist Funding Number: 31601145, and supported by

Federal Ministry of Education and Research (BMBF) Germany to the Goettingen Bernstein Center for Computational Neuroscience Project D1.

**References**

1. Yang SN, Tang YG, Zucker RS. Selective induction of LTP and LTD by postsynaptic [Ca^2+^] elevation. *J. Neurophysiol*. 1999, **81**:781–187.

2. Chistiakova M, Bannon NM, Bazhenov M, Volgushev M. Heterosynaptic plasticity: multiple mechanisms and multiple roles. *Neuroscientist* 2014, **20(5)**:483–498.

3. Graupner M, Brunel N. Calcium-based plasticity model explains sensitivity of synaptic changes to spike pattern, rate, and dendritic location. *Proc. Natl. Acad. Sci.* USA 2012, **109(10)**:3991–3996.

4. Ania Majewska, Edward Brown, Jonathan Ross, and Rafael Yuste. Mechanisms of calcium decay kinetics in hippocampal spines: role of spine calcium pumps and calcium diffusion through the spine neck in biochemical compartmentalization. *J. Neurosci.* 2000, **20(5)**: 1722–1734.

5. Higley MJ, Sabatini BL. Calcium signaling in dendrites and spines: practical and functional considerations. *Neuron* 2008, **59**: 902–913.

6. Larkum ME, Nevian T. Synaptic clustering by dendritic signaling mechanism. *Curr. Opin. Neurobiol*. 2008, **18**:321–331.

7. Harvey CD, Svoboda K. Locally dynamic synaptic learning rules in pyramidal neuron dendrites. *Nature* 2007, **450**:1195–1200.

## P149 Determination of the spike-train power spectrum statistics in modular networks with mixtures of different excitatory and inhibitory populations

### Rodrigo F. O. Pena^1^, Davide Bernardi^2,3^, Antonio C Roque^1^, Benjamin Lindner^2,3^

#### ^1^Department of Physics, University of Sao Paulo, Ribeirao Preto, Sao Paulo, Brazil; ^2^Theory of Complex Systems and Neurophysics, Bernstein Center for Computational Neuroscience, Berlin, Germany; ^3^Department of Physics, Humboldt University of Berlin, Berlin, Germany

##### **Correspondence:** Rodrigo F. O. Pena (rodrigo.pena@usp.br)

*BMC Neuroscience* 2017, **18(Suppl 1)**:P149

Spike-trains produced in recurrent neural networks are in general of non-Poissonian nature as revealed by their non-white (colored) power spectrum. Nevertheless, most theoretical studies are based on the assumption that the stimulus delivered to a single-neuron has a Poisson nature, consequently missing temporal correlations. For instance, the standard Fokker–Planck approach in its simplest version assumes that single neurons are stimulated by white Gaussian noise. If we assume that the major source of noise received by any neuron in the network comes from the quasi-random input from other cells, we encounter the following problem of self-consistency: for neurons arbitrarily picked in every population of the network, the input spike-trains should have the same second-order statistics of the output spike-train per population. By self-consistency, temporal correlations should be preserved. We investigate self-consistency using an extended version of an iterative scheme proposed by Lerchner et al. [1] and extended by Dummer et al. [2]: instead of simulating a network, we simulate one single neuron for each population over several generations injecting surrogate noise input with the same second-order statistics of the output of the previous generation. We show that the power spectrum converges self-consistently when the mean input is close to balance. Neurons were modeled as leaky integrate-and-fire neurons. We compare our results with large modular networks of leaky integrate-and-fire neurons and with different mixtures of excitatory and inhibitory populations [3]. In Figure 1A we show an example of a modular network. In Figure 1B and 1C we show the agreement between power spectra from network and scheme extracted from the same configuration.
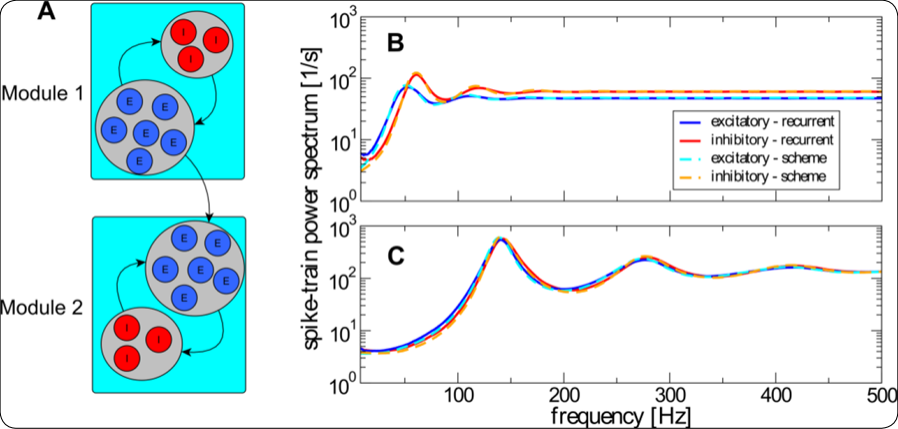


**Figure 1.** Modular network with different populations. **A.** network sketch, populations E and I are modeled with different membrane time constant and are connected via C_E_ and C_I_ links. Module 1 sends 0.01C_E_ links to module 2. Each module contains 10^4^ cells with E:I ratio 4:1. **B** and **C**. scheme and network power spectra for modules 1 and 2

**Acknowledgements**

Research supported by FAPESP 2015/50122-0 and DFG-GRTK 1740/2. RP and AR are also part of the Research, Innovation and Dissemination Center for Neuromathematics FAPESP grant (2013/07699-0). RP is supported by a FAPESP scholarship (2013/25667-8). ACR is partially supported by a CNPq fellowship (grant 306251/2014-0).

**References**

1. Lerchner A, Ursta C, Hertz J, Ahmadi M, Ruffiot P, Enemark S: Response variability in balanced cortical networks. *Neural Comput 2006*, 18, 634.

2. Dummer B, Wieland S, Lindner B: Self-consistent determination of the spike-train power spectrum in a neural network with sparse connectivity. *Front in Comp Neur* 2014, 8:104.

3. Brunel N: Dynamics of sparsely connected networks of excitatory and inhibitory spiking neurons. *J Comput Neurosci* 2000, 8:183–208.

## P150 Optimal detection of single-cell stimulation in large random networks of integrate-and-fire neurons

### Davide Bernardi^1,2^, Benjamin Lindner^1,2^

#### ^1^Bernstein Center for Computational Neuroscience - Berlin, Berlin, 10115, Germany; ^2^Department of Physics, Humboldt University, Berlin, 12489, Germany

##### **Correspondence:** Davide Bernardi (davide.bernardi@bccn-berlin.de)

*BMC Neuroscience* 2017, **18(Suppl 1)**:P150

It has been proposed that the cerebral cortex operates in a noisy chaotic regime, in which only averages over large populations carry information [1]. In this picture, the experimental evidence that the stimulation of a single cell in the barrel cortex can influence the behavior of an awake rat [2,3] is highly surprising and still calls for a theoretical explanation.

We investigate the effects of the stimulation of a single cell in a realistically-sized random network of integrate-and-fire neurons [4] with exponentially distributed synaptic weights [5] and propose a simple readout mechanism to detect the occurrence of the stimulus. Our numerical simulations and analytical estimates yield detection rates that are comparable to the experimental results if the readout is slightly biased toward specific neurons, a proxy for the training of the experimental subjects. These findings are robust for a wide range of intermediate values of the recurrent coupling strength.

**Acknowledgements**

This work was funded by the BMBF (FKZ: 01GQ1001A) and by the DFG (GRK1589/2).

**References**

1. London M, Roth A, Beeren L, Häusser M, Latham P: Sensitivity to perturbations in vivo implies high noise and suggests rate coding in cortex. *Nature* 2010, **466:** 123–127.

2. Houweling A, Brecht M: Behavioural report of single neuron stimulation in somatosensory cortex. *Nature* 2008, **451:** 65–68

3. Doron G, von Heimendahl M, Schlattmann P, Houweling A, Brecht M: Spiking irregularity and frequency modulate the behavioral report of single-neuron stimulation. *Neuron* 2014, **81:** 653–663

4. Brunel N: Dynamics of sparsely connected networks of excitatory and inhibitory spiking neurons. *J Comput Neurosci* 2000, **8:** 183–208

5. Richardson M, Swarbrick R: Firing-rate response of a neuron receiving excitatory and inhibitory synaptic shot noise. *Phys Rev Lett* 2010, **105:** 178102.

## P151 Self-consistent power spectra from an iterative scheme for recurrent heterogeneous networks

### Sebastian Vellmer^1,2^ and Benjamin Lindner^1,2^

#### ^1^Theory of Complex Systems and Neurophysics, Bernstein Center for Computational Neuroscience, Berlin, Germany; ^2^Department of Physics, Humboldt Universität zu Berlin, Berlin, Germany

##### **Correspondence:** Sebastian Vellmer (sebastian.vellmer@bccn-berlin.de)

*BMC Neuroscience* 2017, **18(Suppl 1)**:P151

Dynamics of random networks of excitatory and inhibitory spiking neurons with sparse connectivity have been studied for a long time [1,2]. Spike-trains of single neurons in these networks display temporal correlation and a non-flat power spectrum. Instead of simulating a whole homogeneous network, where neurons are driven by a large number of spikes with small amplitude, a self-consistent power spectrum can be determined by an iterative scheme [2]. In this scheme, a single neuron is driven by Gaussian noise, initially with white Gaussian noise, whereby the noise for the next iteration is generated with the same power-spectrum as the one from output spike trains. In some cases, it converges to a self-consistent spectrum, that does not change through another iteration. Here we extend this scheme to determine self-consistent spectra for heterogeneous networks, in particular, we use binomial distributed numbers of presynaptic neurons and additionally exponentially distributed synaptic weights. Figure 1A presents a sketch of the used iterative scheme. To include heterogeneity in the iterative scheme, we drive few neurons with Gaussian noise instead of a single one. Initially we use white noise as in [2], but we increase the mean µ of the noise for each neuron and determine their power spectra separately. Subsequently we calculate the average spectrum for the next iteration as a sum of the individual power spectra weighted with the probability to find a neuron in a heterogeneous network with mean input in an interval around the used µ. We assume that the distribution of µ is Gaussian in heterogeneous networks. The variance of this distribution depends on the considered heterogeneity and can be approximated analytically in our cases. In the next iteration, we generate Gaussian noise from the average spectrum and drive the few neurons with increasing mean for each again. After only 2 iterations the average spectrum as well as the individual neuron spectra converge to self-consistent spectra that also appear in network simulations. Figure 1B presents the average spectrum of the heterogeneous network. We found self-consistent power spectra in heterogeneous networks which can be determined by extending the iterative scheme in [2] to few neurons, instead of a single one, that are driven with different mean input for each. The scheme is a powerful tool and might be helpful for a better understanding of network dynamics in future.
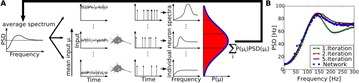


**Figure 1. A.** Sketch of the iterative scheme: We drive few neurons with Gaussian noise, initially white noise (dashed line) with different mean for each and determine their individual power spectra. Through a weighted sum, we determine the average spectrum for the next iteration and drive the few neurons with Gaussian noise with the determined power spectrum and different mean. **B.** Resulting input spectrum for a heterogeneous network with binomial distributed numbers of presynaptic neurons and exponential distributed synaptic weights compared to the iterative scheme

**References**

1. Brunel, N: Dynamics of sparsely connected networks of excitatory and inhibitory spiking neurons. *J. Comput. Neurosci* 2000, **8:**183–208.

2. Dummer B, Wieland S and Lindner B: Self-consistent determination of the spike-train power spectrum in a neural network with sparse connectivity. *Front. Comput. Neurosci* 2014, **8:**104

## P152 Astrocyte-modulated synaptic plasticity in sensory cortex: a computational study

### Ausra Saudargiene^1,2†^, Tiina Maninen^3†^, Riikka Havela^3^, Marja-Leena Linne^3^

#### ^1^Neuroscience Institute, Lithuanian University of Health Sciences, Kaunas, LT-50161, Lithuania; ^2^Department of Informatics, Vytautas Magnus University, Kaunas, LT-44404, Lithuania; ^3^Computational Neuroscience Group, BioMediTech Institute and Faculty of Biomedical Sciences and Engineering, Tampere University of Technology, Tampere, Finland

##### **Correspondence:** Marja-Leena Linne (marja-leena.linne@tut.fi)

^†^Equal contribution

*BMC Neuroscience* 2017, **18(Suppl 1)**:P152

Astrocytes have been historically regarded as glial cells responsible for the homeostasis and metabolic support for neurons. Based on current knowledge astrocytes can use different mechanisms to interact with neurons and express an overwhelming complexity of molecular and cell-level signaling [1]. The mechanisms involved seem to depend not only on the developmental stage of an animal but also on the brain area, neural circuitry, as well as on the experimental technique used to characterize the phenomena. Recent evidence suggests that astrocytes play a key role in modulating synapses, in a variety of cortical areas in vitro [2], [3].

In this study, we aim to identify the key cellular and molecular mechanisms involved in astrocyte modulation of synaptic long-term plasticity in sensory cortex by means of computational modeling techniques. We have previously evaluated more than 80 published computational models of astrocytes and astrocyte-neuron interactions and, additionally, analyzed the reproducibility and comparability of some of these models [4]. Based on these earlier studies we developed a new detailed biophysico-chemical model of a tripartite synapse. The tripartite synapse model consists of a single cortical presynaptic compartment and two postsynaptic compartments of a cortical neuron as well as an adjacent cortical astrocyte process. The model includes the well-established biophysical mechanisms for neuronal sodium and neuronal and astrocytic calcium excitability, as well as the biochemical signaling pathways putatively important in plasticity for all the three tripartite synapse elements. Classical pre- and postsynaptic stimulation protocols for long-term plasticity were applied as synaptic inputs to activate the model.

Our simulations show that changes in the temporal patterns of synaptic input affect endocannabinoid production and release at the postsynaptic site, elevate endocannabinoid-induced calcium levels in the astrocyte process, induce gliotransmission from the astrocytic process, and modulate the probability of the vesicle release at the presynaptic terminal. The long-term goal of our work is to develop extended synapse models, including other mechanisms possibly contributing to plasticity, to address the controversies [5] present in the field. The biophysically and biochemically detailed models developed by us may be used to address the involvement of astrocytes in learning in cortical networks interconnected by synapses.

**Acknowledgements**

This project received funding from the European Union Seventh Framework Programme (FP7) under grant agreement No. 604102 (HBP), European Union’s Horizon 2020 research and innovation programme under grant agreement No. 720270, and Academy of Finland (decision No. 297893). The authors wish to thank Tampere University of Technology Graduate School, Emil Aaltonen Foundation, The Finnish Concordia Fund, and Ulla Tuominen Foundation for support for RH.

**References**

1. Volterra A, Liaudet N, Savtchouk I: Astrocyte Ca^2+^ signalling: an unexpected complexity. *Nat Rev Neurosci* 2014, **15**(5):327–335.

2. Perea G, Araque A: Astrocytes potentiate transmitter release at single hippocampal synapses. *Science* 2007, **317**(5841):108–-6.

3. De Pitta M, Brunel N, Volterra A: Astrocytes: Orchestrating synaptic plasticity? *Neuroscience* 2016, **323**:43–61.

4. Manninen T, Havela R, Linne M-L. Reproducibility and comparability of computational models for astrocyte calcium excitability. *Front Neuroinform* 2017, **11**:11.

5. Agulhon C, Fiacco TA, McCarthy KD: Hippocampal short- and long-term plasticity are not modulated by astrocyte Ca^2+^ signaling. *Science* 2010, **327**(5970):1250–4.

## P153 Up and down states statistics for Gamma oscillations

### Arthur Powanwe, Andre Longtin

#### Department of Physics and Centre for Neural Dynamics, University of Ottawa, Ottawa, Canada

##### **Correspondence:** Arthur Powanwe (apowa074@uottawa.ca)

*BMC Neuroscience* 2017, **18(Suppl 1)**:P153

Gamma oscillations are ubiquitous in the brain and are believed to be useful for perceptual and cognitive behaviour, coding properties or communication between brain areas. Usually, recorded LFP or EEG show self-sustained oscillations at gamma frequency which exhibit epochs of high amplitude oscillations (up states) alternating with epochs of low amplitude (down states). Such spontaneous up and down states are believed to be involved in working memory, modulation of neuronal excitability with attention or generation of spontaneous activity during sleep. Moreover, they have been showed to arise due to local cortical circuits which operate through a balance of excitation and inhibition [1,2]. But statistics of these up and down states like transition rates between the two states, probability densities or even Serial Correlation Coefficients between states are poorly understood. The goal is to give a novel characterisation of up and down states by deriving exact analytics expressions for the transitions cited above.

To this end, we consider a network of stochastic spiking neurons that can self-generate avalanche dynamics or oscillations in gamma frequency [3,4]. The activities of the excitatory and inhibitory populations can be described by stochastic Wilson-Cowan equations. When the network exhibits oscillations, the stochastic Wilson-Cowan equations possess noisy limit cycles or “quasi-cycles”. In the case of quasi-cycles, the corresponding deterministic Wilson-Cowan equations possess a fixed point with complex conjugate eigenvalues with negative real part. Oscillations are then the consequence of the stochastic spiking of each single neuron and the finite size of the system. Moreover, such oscillations show up and down states. To describe the dynamics of the system around its corresponding fixed point, we can express the activities of the populations as the sums of fixed-points activities plus some deviations, a technique called Linear-Noise-Approximation (LNA). The equations obtained from LNA are coupled stochastic linear equations describing noisy gamma oscillations. Going further in the analysis, we are able to describe gamma oscillations by uncoupled phase and amplitude equations. The corresponding amplitude equation is related to the parameters of the system and allow us to have a clear understanding of gamma oscillations up and down states and transitions between them. We can then assume that up and down states are the consequence of the transitions of the amplitude of gamma oscillations above and below a fixed threshold. Using the amplitude equation derived from the LNA we can derive analytic expressions for the stationary probability densities as well as the transition rate from down to up state and the converse. Moreover, we can also derive analytic expressions for higher order statistics like Serial Correlation Coefficients (SCC) between states. Numerical simulations are also performed and confirm the analytic expressions obtained.

**References**

1. Y.Shu, A.Hasenstaub, and D.A.McCormick. Turning on and off recurrent balanced cortical activity. *Nature*, **423:**288–293, 2003.

2. D.Holcman and M. Tsodyks. The emergence of up and down states in cortical networks. *Plos Comput. Biol*. **2:e23**, 2006.

3. M.Benayoun, J.Cowan,W.van Drongelen and E.Wallace. Avalanches in a stochastic model of spiking neurons. *Plos Comput. Biol*. **6(7):** e1000846.

4. E.wallace, M.Benayoun, W.van Drongelen and J.Cowan. Emergent Oscillations in networks of stochastic Spiking Neurons. *Plos Comput.* Biol. **5(6):**e14804, 2011

## P154 EDLUT: a real-time spiking neural network simulator for embodiment experiments

### Francisco Naveros^1^, Jesús A. Garrido^1^, Richard R. Carrillo^1^, Eduardo Ros^1^, Niceto R. Luque^2,3,4^

#### ^1^Department of Computer Architecture and Technology, University of Granada (CITIC), Granada, Spain; ^2^INSERM, U968, Paris, France; ^3^Sorbonne Universités, UPMC University Paris 06, UMR_S 968, Institut de la Vision, Paris, France; ^4^CNRS, UMR_7210, Paris, France

##### **Correspondence:** Francisco Naveros (fnaveros@ugr.es)

*BMC Neuroscience* 2017, **18(Suppl 1)**:P154

EDLUT is a spiking neural simulator mostly oriented towards the efficient simulation of medium-scale neural networks connected to a body (embodiment). This body can be a virtual or real robot (this last option requires real-time (RT) simulations). Embodiment helps to better understand how certain capabilities of the nervous system (e.g. the role of the cerebellum in coordinated movements and object manipulation) emerge based on cellular characteristics, network topology, or particular synaptic adaptation mechanisms.

EDLUT incorporates a hybrid event- and time-driven simulation scheme in multi-core CPU-GPU co-processing platforms. This scheme uses point neurons (LIF, AdEx, Izhikevich and HH) and optimized STDP mechanisms that allow EDLUT to operate in a single general-purpose computer in RT. EDLUT natively implements event-driven methods in CPU to cope with RT requirements. These event-driven methods pre-compute the neural dynamic evolution in look-up tables which are used later during simulation [1, 2]. It also implements time-driven methods in CPU and GPU [3, 4] that are mainly focused on RT performance using fixed-step and bi-fixed-step integration methods to compute the neural dynamic evolution [2]. Event- and time-driven methods use OpenMP in CPU and CUDA in GPU to parallelize the simulation. Thus, EDLUT takes advantage of the strengths and mitigate the weaknesses of each simulation method and parallelization technique. EDLUT also incorporates STDP mechanisms that are RT oriented. EDLUT uses a synaptic-redistribution mechanism in RAM together with a two-step updating mechanism that minimizes the number of cache failures when updating synaptic weights during simulation.

EDLUT uses a RT supervisor to control the timing of the neural-network-body interaction. This supervisor adapts the neural processing to facilitate the communication between the sensory/actuator body systems and the neural simulation dynamically. EDLUT allows the RT supervisor to momentarily disable several neural processes (spikes generation and propagation, plasticity mechanism, neural dynamic evolution, etc.) to cope with RT constraint when operating a connected body [5].

EDLUT is one among the few tools that allow the simulation of biologically-plausible spiking neural networks in RT for embodiment experimentation.

**Acknowledgements**

This study was supported by the Spanish National Grant PhD scholarship (AP2012-0906).

**References**

1. Ros E, Carrillo R, Ortigosa EM, Barbour B, Agís R: Event-driven simulation scheme for spiking neural networks using lookup tables to characterize neuronal dynamics. *Neural computation* 2006, **18(12):**2959–2993.

2. Naveros F, Garrido JA, Carrillo RR, Ros E, Luque NR: Event-and Time-Driven Techniques Using Parallel CPU-GPU Co-Processing for Spiking Neural Networks. *Frontiers in Neuroinformatics* 2017, **11**:7.

3. Garrido JA, Carrillo RR, Luque NR, Ros E: Event and time driven hybrid simulation of spiking neural networks. In: *International Work*-*Conference on Artificial Neural Networks: Springer* 2011, 554–561.

4. Naveros F, Luque NR, Garrido JA, Carrillo RR, Anguita M, Ros E: A spiking neural simulator integrating event-driven and time-driven computation schemes using parallel CPU-GPU co-processing: a case study. *IEEE transactions on neural networks and learning systems* 2015, **26(7):**1567–1574.

5. Luque NR, Carrillo RR, Naveros F, Garrido JA, Sáez-Lara M: Integrated neural and robotic simulations. Simulation of cerebellar neurobiological substrate for an object-oriented dynamic model abstraction process. *Robotics and Autonomous Systems* 2014, **62(12):**1702–1716.

## P155 Embedded Ensemble Encoding — a hypothesis for reconciling cortical coding strategies

### Joe W. Graham^1^, Salvador Dura-Bernal^1^, Sergio L Angulo^1^, Samuel A Neymotin^1,2^, Srdjan D Antic^3^, William W Lytton^1,4^

#### ^1^Department of Physiology and Pharmacology, SUNY Downstate Medical Center, Brooklyn, NY, 11203, USA; ^2^Department of Neuroscience, Brown University, Providence, RI 02912, USA; ^3^Institute for Systems Genomics, University of Connecticut Health Center, Farmington, CT, 06030, USA; ^4^Department of Neurology, Kings County Hospital, Brooklyn NY 11203, USA

##### **Correspondence:** Joe W. Graham (joe.w.graham@gmail.com)

*BMC Neuroscience* 2017, **18(Suppl 1)**:P155

As well as synaptically-induced NMDA spikes, basal dendrites of cortical pyramidal neurons can produce distinct plateaus of longer duration which depolarize the soma as well [1]. During these periods of sustained depolarization (comparable to Up states observed in sleep and anesthesia), the neuron is readily able to follow external inputs. Cells in this activated or “standby” state show higher rates of firing, interpretable as rate coding. In addition, fortune and synaptic inputs will favor these prepared neurons, so that subsets of the standby ensembles could be readily recruited into sub-ensembles based on simultaneous firing. This could happen in coordination with beta or gamma activity, which would be identifiable as evidence for temporal coding by firing synchrony.

Thus, our embedded-ensemble encoding (EEE) theory allows seemingly distinct coding strategies to coexist.

The synchronized spiking of the sub-ensembles would provide the possibility for formation of broad distributed ensemble-by-synchrony across areas that has been hypothesized to allow the binding of multimodal features into coherent object perception (binding-by-synchrony theory). EEE also has implications for Bayesian predictive coding theory — we propose the outer ensemble (plateau ensemble) as a predictor for codings in the nested ensemble.

We have begun to explore the phenomenology of dendritic plateau potentials at the cellular level, demonstrating that we are able to obtain dendritic and somatic plateaus in a morphologically-simplified model pyramidal neuron model which are comparable to those seen physiologically in vitro during focal glutamate application (Figure 1). Sufficient weight of input to a synapse modeled with NMDA and AMPA receptors located in a basal dendrite can bring the soma into a prolonged depolarized plateau. We are now placing these cells into simulated cortical columns in order to explore how plateau potentials affect network behavior.
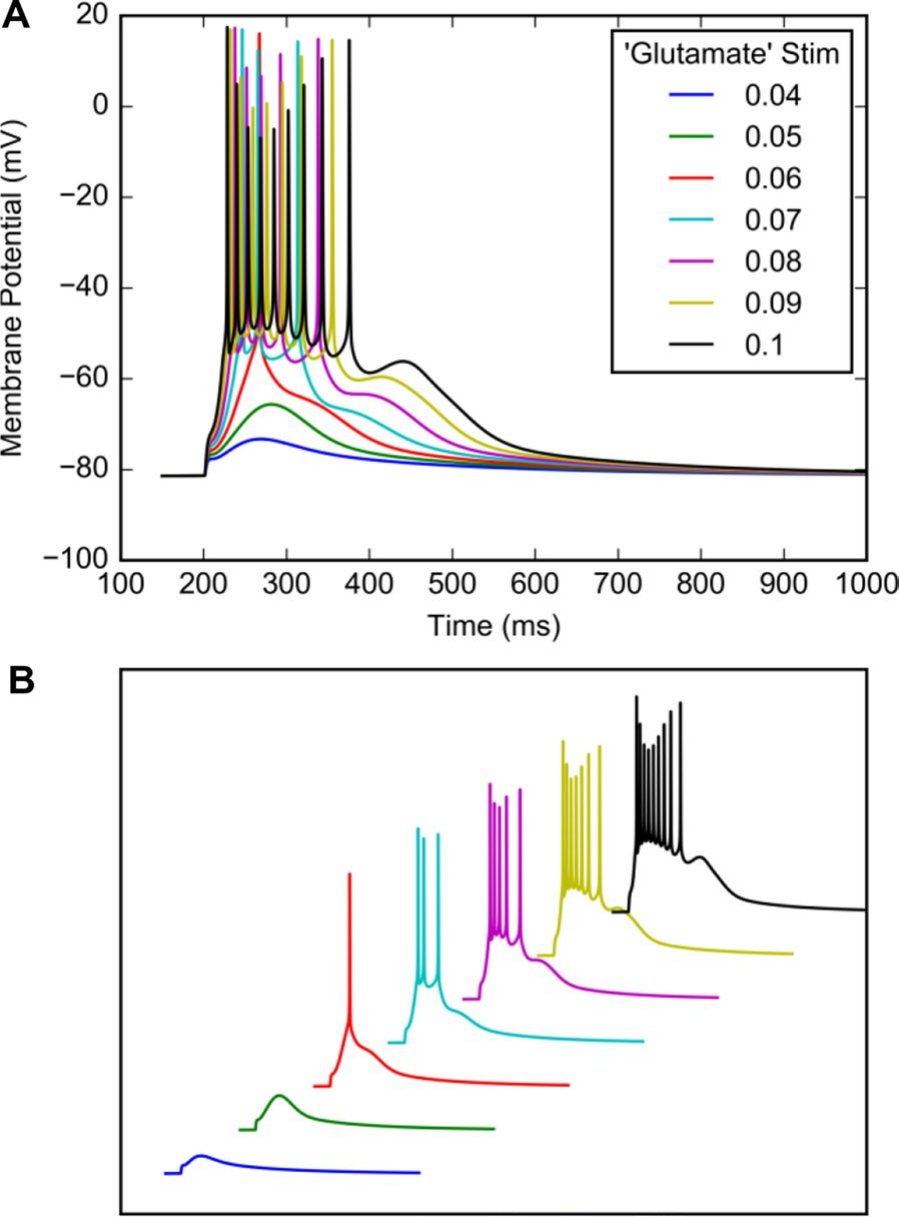


**Figure 1.** Model plateaus. **A.** Somatic membrane potential (mV) versus time (ms) for a range of simulated glutamate stimulation amplitudes. **B.** Same traces separated for clarity. As seen experimentally, increasing the stimulation amplitude increases plateau height to a saturated value on the range of 25 mV, with increasing stimulation amplitude only increasing the duration of the plateau eds of milliseconds)

**Reference**

1. Oikonomou KD, Singh MB, Rich MT, Short SM, Antic SD: Contribution of extrasynaptic N-methyl-D-aspartate and adenosine A1 receptors in the generation of dendritic glutamate-mediated plateau potentials. *Philos Trans R Soc Lond B Biol Sci* 2015, **370**. Available from: http://dx.doi.org/10.1098/rstb.2014.0193

**Publisher’s Note**

Springer Nature remains neutral with regard to jurisdictional claims in published maps and institutional affiliations.

